# Sulphide as a leaving group: highly stereoselective bromination of alkyl phenyl sulphides†
†Electronic supplementary information (ESI) available: Experimental procedures and spectroscopic characterization for all new compounds, including details of NMR experiments and racemisation study. See DOI: 10.1039/c9sc03560e


**DOI:** 10.1039/c9sc03560e

**Published:** 2019-08-16

**Authors:** Daniele Canestrari, Caterina Cioffi, Ilaria Biancofiore, Stefano Lancianesi, Lorenza Ghisu, Manuel Ruether, John O'Brien, Mauro F. A. Adamo, Hasim Ibrahim

**Affiliations:** a Centre for Synthesis and Chemical Biology (CSCB) , Department of Chemistry , Royal College of Surgeons in Ireland , 123 St. Stephen's Green , Dublin 2 , Ireland . Email: madamo@rcsi.ie ; Email: hasimibrahim@rcsi.ie; b IRBM Science Park S.p.A , Department of Medicinal Chemistry , Via Pontina, 30.600 , 00071 Pomezia RM , Italy; c Trinity Biomedical Sciences Institute , School of Chemistry , The University of Dublin , Trinity College , Dublin 2 , Ireland

## Abstract

A conceptionally novel nucleophilic substitution approach to synthetically important alkyl bromides is presented. Using molecular bromine (Br_2_), readily available secondary benzyl and tertiary alkyl phenyl sulphides are converted into the corresponding bromides under exceptionally mild, acid- and base-free reaction conditions. This simple transformation allows the isolation of elimination sensitive benzylic β-bromo carbonyl and nitrile compounds in mostly high yields and purities. Remarkably, protic functionalities such as acids and alcohols are tolerated. Enantioenriched benzylic β-sulphido esters, readily prepared by asymmetric sulpha-Michael addition, produce the corresponding inverted bromides with high stereoselectivities, approaching complete enantiospecificity at –40 °C. Significantly, the reported benzylic β-bromo esters can be stored without racemisation for prolonged periods at –20 °C. Their synthetic potential was demonstrated by the one-pot preparation of γ-azido alcohol (*S*)-**5** in 90% ee. NMR studies revealed an initial formation of a sulphide bromine adduct, which in turn is in equilibrium with a postulated dibromosulphurane intermediate that undergoes C–Br bond formation.

## Abstract 2

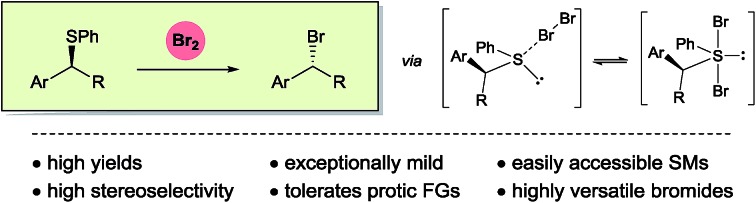
Molecular bromine is shown to stereoselectively brominate readily available alkyl phenyl sulphides under exceptionally mild reaction conditions.

## Introduction

**Scheme 1:**
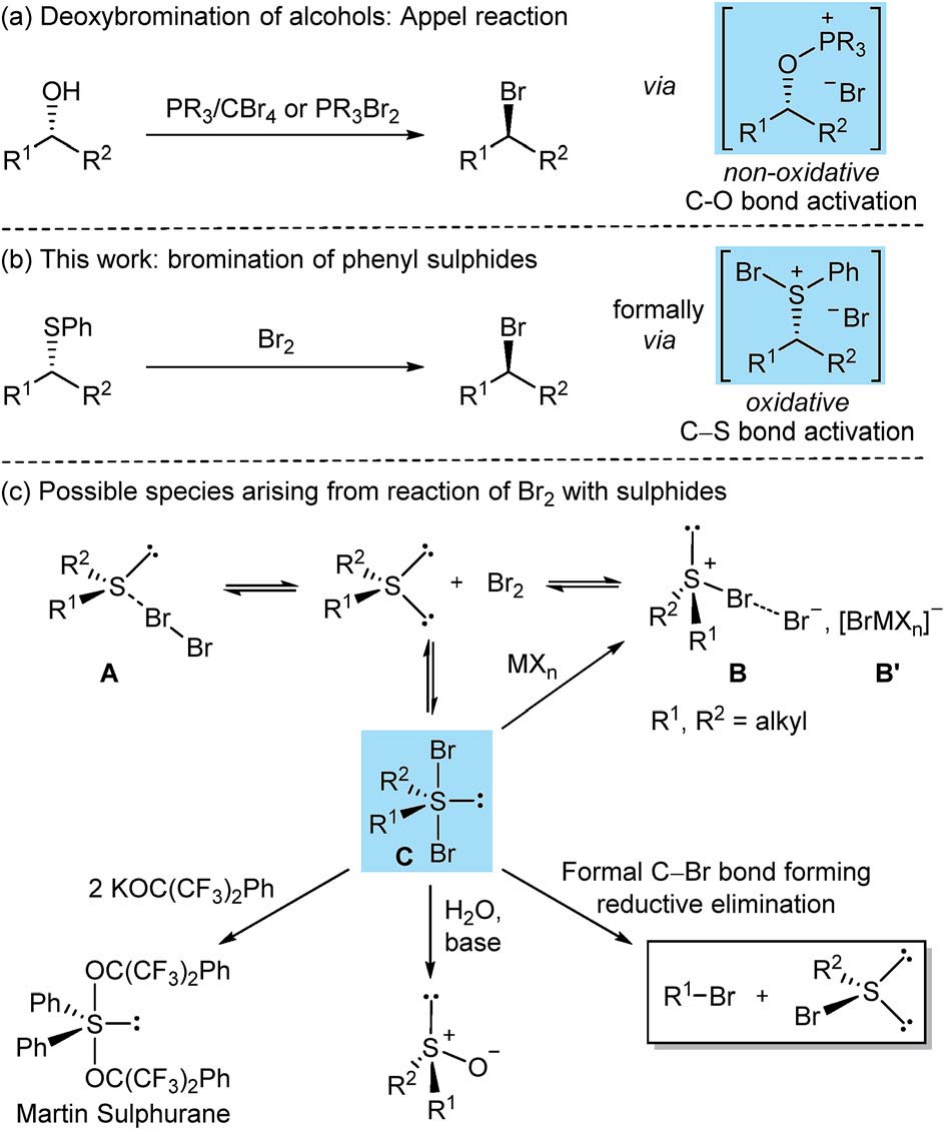
Nucleophilic bromination of alkyl phenyl sulphides in context.

Alkyl bromides are versatile and extensively utilised synthetic intermediates, constitute precursors for a wide range of C–C^1^ and C–heteroatom^2^ bond forming reactions, and are motifs found in biologically active natural products.^3^ Given their importance, numerous methods have been developed for their preparation,^4^ with protocols relying on the nucleophilic substitution of alkyl alcohols with bromide ions remaining the most widely applied and studied.^5^,^6^ Among them, the use of phosphorus(v) or phosphorus(iii) reagents is particularly widespread (Scheme 1a).^7^ Catalytic variants of the phosphorus(v) based Appel reaction have been developed recently,^8^ including a catalytic system for the *direct* deoxybromination of alcohols.8*b* Other innovative modes for catalytic activation of alkyl alcohols towards bromide ion substitution have recently emerged;6b,^9^ however, these protocols proceed through *in situ* substitution of catalytically formed alkyl chloride intermediates by added exogenous bromide ions (Finkelstein reaction).6b,9d

The above methods showcase the recent progress made in the deoxybromination of alkyl alcohols; however, challenges remain such as alkene side product formation from elimination sensitive bromides, limited scope and functional group tolerance, difficulties in removing by-products such as phosphine oxides, requirement of multiple reagents and bromide racemisation from optically active alkyl alcohols.6a,^10^

We have recently reported a novel nucleophilic chlorination approach to obtaining alkyl chlorides from readily available alkyl phenyl sulphides.^11^ This mild and rapid (dichloroiodo)benzene (PhICl_2_) promoted transformation proceeded formally *via* a S_N_2 chloride ion attack on a proposed, oxidatively generated chlorosulphonium chloride intermediate.

Considering the above-mentioned limitations in the preparation of alkyl bromides from alcohols, we posed the question whether sulphides could be used as precursors for alkyl bromides. Sulphides can be accessed by a number of methods, especially *via* thiol addition to widely available and inexpensive Michael acceptors. However, it was unclear if this was a viable approach, since Br_2_ and other Br^+^ equivalents are weaker oxidising agents than PhICl_2_ (*vide infra*).

Herein, we delineate how the concept of ‘sulphide as leaving group’ was exploited for the bromination of alkyl phenyl sulphides using Br_2_, an inexpensive laboratory commodity (Scheme 1b). This study culminated in the development of an exceptionally mild, chemoselective and highly stereoselective nucleophilic bromination reaction.

The reaction of diorganosulphides with Br_2_ has been known for over 100 years to oxidise the sulphur(ii) centre;^12^ however, conflicting reports on the nature of the formed species have been disclosed. Depending on sulphide substitution, temperature, methods of generation, isolation and structural analysis (*i.e.* in the solid state or in solution),^13^ it has been suggested that the reaction could, *via* an equilibrium, generate tetrahedral sulphur(ii) molecular complexes **A** (MCs), trigonal pyramidal sulphur(iv) bromosulphonium bromides **B** or trigonal bipyramidal (TB) sulphur(iv) dibromosulphurane adducts **C** (Scheme 1c).^13^,^14^ NMR spectroscopic studies in support of adducts **A** and/or **C** in solution have been reported.^15^

In the case of dialkyl substitution, the treatment of bromine adducts with Lewis acidic metal halides (MX_*n*_) was reported to form bromosulphonium [BrMX_*n*_] salts **B′** by irreversible bromide ion complexation.13b,14c,^16^ These salts have found widespread application in organic synthesis as, for example, bromonium ion (Br^+^) equivalents and/or oxidants.^16^,^17^ Dibromosulphuranes **C** have been proposed as intermediates in several transformations. For instance, in his seminal work on organosulphuranes, Martin employed dibromosulphuranes as intermediates en route to alkoxysulphuranes,^18^ such as the versatile Martin sulphurane reagent.^19^ These reactions proceed *via* alkoxide displacement of the apical bromide ligands. In a related transformation, sulphuranes **C** were converted into the corresponding sulphoxides upon treatment with water and a base (Scheme 1c).^20^ However, that dibromosulphurane intermediates could reductively collapse to form concurrently alkyl bromides and organosulphenyl bromides has to the best of our knowledge, not been reported. Herein, we show that, with the right choice of the substituents on the sulphur, such a transformation is indeed feasible, and in doing so uncover that aryl sulphides can be considered a valuable, heretofore unexplored leaving group for highly stereoselective, non-neighbouring group assisted, nucleophilic bromination.

## Results and discussion

**Scheme 2:**
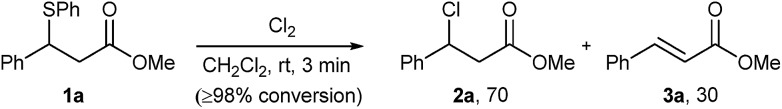
Reaction of β-sulphido ester **1a** with Cl_2_.

As part of our studies on desulphurative chlorination with PhICl_2_, we examined the ability of other electrophilic chlorinating reagents, and specifically that of elemental chlorine, to promote chlorination of β-sulphido esters. In a preliminary experiment, we treated β-sulphido ester **1a** with Cl_2_ gas and observed a rapid consumption of the starting material within 3 minutes. ^1^H NMR analysis of the crude material showed the formation of β-chloro ester **2a** as the major product, accompanied by dehydrochlorination-derived methyl cinnamate (**3a**) in a ratio of 70 : 30 (Scheme 2). In spite of significant alkene side-product formation, this experiment clearly demonstrated that molecular chlorine could promote desulphurative chlorination. This reactivity formed the basis for our efforts to examine elemental bromine in the desulphurative bromination of β-sulphido esters.

In an initial experiment, adding 1.0 equivalent of neat Br_2_ to our test substrate β-sulphido ester **1a** in dry dichloromethane and monitoring the progress of the reaction by ^1^H NMR spectroscopy showed, within 15 minutes, complete conversion to β-bromo ester **4a**, accompanied by small amounts of dehydrobromination-derived methyl cinnamate (**3a**) (Table 1, entry 1). This experiment clearly indicated that Br_2_ could not only promote bromination of sulphide **1a**, but also, gratifyingly, with very little alkene side-product formation. Moreover, this surprising outcome was even more profound when considering the large difference in oxidising power between Cl_2_ and Br_2_.

**Table 1:** Optimisation of reaction conditions with sulphide **1a**^*a*^ ^,^^*b*^

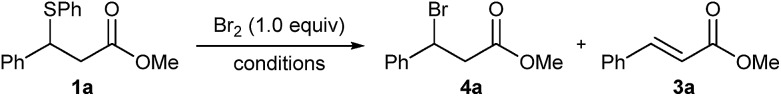				
Entry	Solvent	Temp (°C)	Time^*c*^ (min)	Ratio^*d*^ **4a** : **3a**
1^*e*^	CH_2_Cl_2_	rt	15	98 : 2
2	CH_2_Cl_2_	rt	25	100 : 0 (95)^*f*^
3	CH_2_Cl_2_^*g*^	rt	15	98 : 2
4	Toluene	rt	30	100 : 0
5	DCE	rt	30	98 : 2
6	THF	rt	90	CM
7	MeCN	rt	5	99 : 1 (72)^*f*^
8	CH_2_Cl_2_	0	60	100 : 0
9	CH_2_Cl_2_	–20	180	100 : 0
10	CH_2_Cl_2_	–40	900	100 : 0

^*a*^Conditions: **1a** (0.5 mmol), Br_2_ (0.5 mmol) as 1.0 M solution in reaction solvent, and dry solvent (3.0 mL, 0.17 M); all reactions proceeded to complete conversion (≥98%).

^*b*^Styrene (0.65 mmol, 1.3 equiv.) was added to quench the reaction.

^*c*^Reaction progress was monitored by ^1^H NMR spectroscopy using a stock solution of styrene in CDCl_3_ (0.03 M).

^*d*^Determined by ^1^H NMR spectroscopy of the crude material.

^*e*^Neat Br_2_ (0.5 mmol) used.

^*f*^Isolated yield after SiO_2_ chromatography.

^*g*^Non-purified CH_2_Cl_2_. CM = complex mixture.

To simplify the procedure and ensure accurate addition of Br_2_, we repeated this reaction with a 1.0 M solution of Br_2_ in dry DCM, which gave complete conversion to the brominated product in 25 minutes, and significantly, without the formation of alkene **3a** (entry 2). We were delighted to isolate β-bromo ester **4a** in an excellent yield of 95% after SiO_2_ chromatography. Performing the same reaction in non-purified DCM gave complete conversion in a shorter reaction time of 15 min but with small amounts of alkene **3a** (entry 3). The reaction proceeded equally well in toluene or dichloroethane (DCE), albeit with slightly longer reaction times (entries 4 and 5), whereas THF as solvent gave a complex mixture (entry 6). A marked increase in the reaction rate was observed in MeCN; however, workup and purification gave bromo ester **4a** in a lower yield of 72% (entry 7).

Conducting the reaction in DCM at lower temperatures resulted in longer reaction times but gave clean conversions to bromide **4a**, with the reaction at –40 °C requiring 15 hours for completion (entries 8–10). This is in stark contrast to desulphurative chlorination with PhICl_2_, which showed an increase in alkene side-product formation upon lowering the reaction temperature to 0 °C.^11^ Moreover, in contrast with the limited solubility of PhICl_2_ in chlorinated solvents at lower temperatures,^21^ reactions with Br_2_ remained homogeneous throughout. Crucially, in all of the above reactions only small amounts or no dehydrobrominated alkene **3a** was observed,^22^ which is a testament to the exceptionally mild, base- and acid-free reaction conditions.

Using conditions from entry 2 in Table 1 we proceeded to examine the scope of our bromination with various β-sulphido carbonyl compounds, which generally gave good to excellent yields of the corresponding bromide products (Table 2, **4a–4p**). Reaction times varied with substitution on the aryl ring, with aryl groups having deactivating substituents requiring longer reaction times (4 hours for **1f**). Bromination of slow-reacting sulphides could be accelerated by running the reaction at double the concentration in DCM (**1e**), or in DCE as solvent at 50 °C (**1g** and **1i**). Both heating at 50 °C and excess of Br_2_ were needed for the bromination of sulphides **1h** and **1o**.

**Table 2:** Scope for the bromination of phenyl sulphides **1**^*a*^ ^,^^*b*^

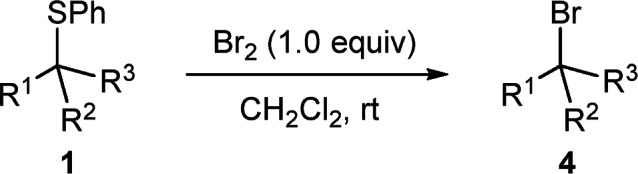
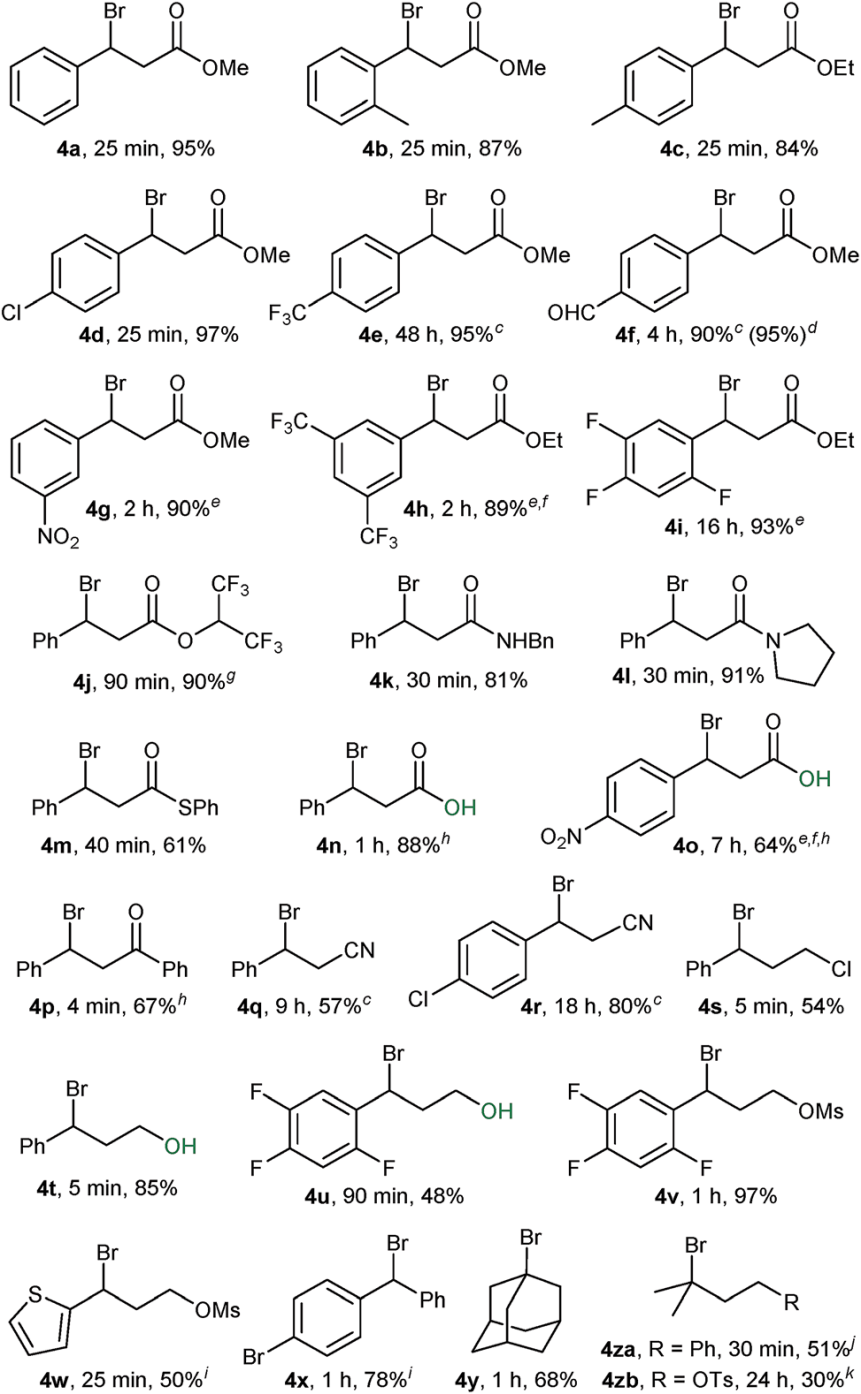

^*a*^Unless otherwise stated, reactions were performed on a 0.5 mmol scale at 0.17 M (3 mL) using 0.5 mmol of Br_2_ as 1.0 M solution in CH_2_Cl_2_; reaction progress was monitored by ^1^H NMR using a stock solution of styrene in CDCl_3_ (0.03 M).

^*b*^Isolated yield after SiO_2_ chromatography.

^*c*^Run at 0.33 M (1.5 mL).

^*d*^5.0 mmol scale.

^*e*^Reaction performed in DCE at 50 °C.

^*f*^Run with 1.0 mmol (2.0 equiv.) of Br_2_.

^*g*^Contains 8% alkene.

^*h*^Isolated yield after trituration or recrystallisation.

^*i*^Yield of the corresponding trifluoroethyl ether derivative.

^*j*^ ^1^H NMR yield using CH_2_Br_2_ as the internal standard.

^*k*^Isolated yield contains 26% 3,4-dibromide side-product.

Remarkably, desulphurative bromination also occurred with substrates having protic functionalities such as β-sulphido acids **1n** and **1o** and sulphido alcohols **1t** and **1u**, a transformation that would be incompatible with the use of the aforementioned phosphorus(v) or phosphorus(iii) based deoxybrominating reagents. The chemoselectivity for sulphido alcohols **1t** and **1u** is even more striking, when considering that the productive intermediate is formally a bromosulphonium ion analogous to the one generated in Swern oxidation. These brominations proceeded to complete conversion, with bromo alcohol **4t** obtained in a high 85% yield. Bromo alcohol **4u** was isolated in a moderate yield after purification; nevertheless, it could be generated *in situ* and taken further in a follow-up reaction. For instance, a one-pot bromination/mesylation sequence of sulphido alcohol **1u** gave bromo mesylate **4v** in 45% yield (97% yield from sulphide **1v**).

Performing the bromination on easily accessible β-sulphido nitriles **1q** and **1r** gave rise to the corresponding β-bromonitriles in fair to good yields. Our method provides a direct and simple route to these novel and potentially versatile compounds,^23^ especially when considering that the only direct literature-known method relies on the mostly low-yielding halodehydration of the corresponding alcohol precursors.^24^

Scale-up of the above reactions proceeded without problems. For instance, a ten-fold scale-up, at 5 mmol, of the bromination of sulphide **1f** gave bromide **4f** in an excellent 95% yield, thus underlining the practicality of the herein reported bromination protocol.

Bromination of sulphides containing electron-rich heteroarenes such as sulphido thiophene **1w** proceeded to complete conversion; however, attempts to purify the corresponding bromide **4w** by chromatography resulted in decomposition. Ultimately, 2,2,2-trifluoroethanol solvolysis afforded the 2,2,2-trifluoroethoxy derivative **4wa** in 50% yield (see the ESI†). Likewise, bromide **4x** was sensitive towards purification by chromatography and was isolated as the 2,2,2-trifluoroethoxy derivative **4xa** in 78% yield.

Initial experiments with adamantyl sulphide **1y** indicated that tertiary alkyl sulphides were suitable substrates. Bromination of sulphide **1y** proceeded with clean complete conversion as indicated by the ^1^H NMR spectrum of the crude material. However, the moderate yield of 68% for bromide **4y** (as well as for **4s**) was due to the chromatographic separation from (PhS)_2_, which possessed similar polarity. Bromination of tertiary dimethylalkyl-derived sulphides proceeded to complete conversion, but was accompanied by elimination side-products. Thus, sulphide **1za** gave within 30 min the corresponding bromide in 51% ^1^H NMR yield together with minor elimination side-products, whereas bromo tosylate **4zb** could be isolated in 30% yield as an inseparable 3 : 1 mixture together with its corresponding Br_2_ alkene addition side-product (see the ESI†).

### Optically active bromides

There are relatively few methods to access optically active, racemisation-sensitive benzylic bromides. By far the most widely applied methods rely on nucleophilic S_N_2 bromination of optically active benzylic alcohols; however, these reactions require accurate monitoring of reaction conditions and usually yield partially racemised bromide products. Potentially very useful, but rare, asymmetric protocols for obtaining benzylic bromides are emerging. These include the recently reported Rh-catalysed asymmetric Kharasch addition to styrenes,^25^ and the Cu-catalysed formal asymmetric hydrobromination of styrenes.^26^ However, these methods require the use of precious metal-chiral phosphine catalysis and display limited substrate scope.

**Scheme 3:**
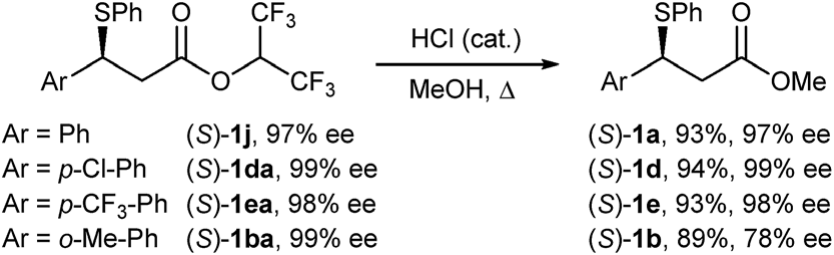
Synthesis of enantiomerically enriched β-sulphido methyl esters.

Given the exceptionally mild reaction conditions to access elimination-sensitive bromides, we proceeded to examine the suitability of our method for the synthesis of highly versatile optically active benzylic β-bromo esters from enantiomerically enriched benzylic β-sulphido esters. Sulphide substrates were prepared according to Wang's asymmetric sulpha-Michael addition of thiophenol to hexafluoroisopropyl cinnamates.^27^ As the bromination of β-sulphido hexafluoroisopropyl esters **1j** and **1ea** gave, after purification by chromatography, bromides containing 8% and 25% alkene, respectively, we decided to investigate the corresponding β-sulphido methyl esters. Thus, acid-catalysed methanolysis was achieved without erosion of the enantiomeric purity for sulphides (*S*)-**1j**, (*S*)-**1da** and (*S*)-**1ea** (Scheme 3). However, methanolysis of sulphido ester (*S*)-**1ba** having 99% ee gave, under the studied conditions, partially racemised (*S*)-**1b** in 78% ee.

Subjecting sulphide (*S*)-**1a** to the optimised bromination at room temperature (Table 1, entry 2) gave inverted bromide (*R*)-**4a** in 83% ee and 86% es (Table 3, entry 1), which was subsequently confirmed to have an *R* absolute configuration (*vide infra*). We were delighted to find that lowering the reaction temperature had a significant effect on the enantioselectivity of the reaction (Table 3, entries 2–4), with the bromination ran at –40 °C affording, after 15 hours, bromide (*R*)-**4a** in an excellent 93% ee and 96% es. Similarly, bromination of sulphide (*S*)-**1d** at –40 °C gave bromide (*R*)-**4d** in 93% ee and 94% es (entry 5).^28^ The slow reacting sulphide (*S*)-**1e** gave at room temperature bromide (*R*)-**4e** in high 86% ee and 88% es (entry 6). Lowering the reaction temperature to 0 °C gave bromide (*R*)-**4e** with a slightly improved enantiomeric excess of 87%, but at the expense of a significant drop in the reaction rate (entry 7). Finally, running sulphide (*S*)-**1b** having 78% ee at –40 °C gave bromide (*R*)-**4b** in 66% ee, which still corresponded to 85% es, showing that the reaction tolerated sterically encumbered *ortho*-substituted aryl groups (entry 8).

**Table 3:** Bromination of enantiomerically enriched β-sulphido esters^*a*^ ^,^^*b*^

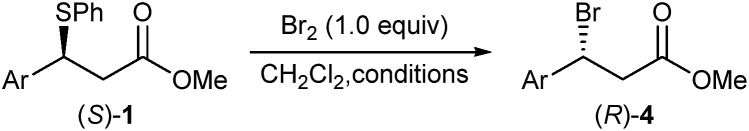						
Ent.	(*S*)-**1**	Temp. (°C)	Time^*c*^	(*R*)-**4** yield^*d*^ (%)	ee^*e*^ (%)	es (%)
1	(*S*)-**1a**	rt	25 min	82	83	86
2	(*S*)-**1a**	0	1 h	94	87	90
3	(*S*)-**1a**	–20	3 h	87	92	95
4	(*S*)-**1a**	–40	15 h	89	93	96
5	(*S*)-**1d**	–40	24 h	97	93	94
6	(*S*)-**1e**	rt	48 h	95	86	88
7	(*S*)-**1e**	0	48 h	36	87	89
8	(*S*)-**1b**	–40	15 h	99	66	85

^*a*^Conditions: (*S*)-**1** (0.25 mmol), Br_2_ (0.25 mmol) as 1.0 M solution in CH_2_Cl_2_, and dry CH_2_Cl_2_ (1.5 mL, 0.17 M).

^*b*^Styrene (0.3 mmol, 1.3 equiv.) was added to quench the reaction.

^*c*^Reaction progress was monitored by ^1^H NMR spectroscopy using a stock solution of styrene in CDCl_3_ (0.03 M).

^*d*^Isolated yield after SiO_2_ chromatography.

^*e*^ee values were determined by HPLC analysis on the chiral stationary phase.

Optically active benzylic bromides have been reported to be prone to racemisation.1c,e,^10^ For instance, a sample of (*R*)-1-(bromoethyl)benzene having 88% ee kept at 0 °C was shown to fully racemise within 8 hours.1*c* We therefore monitored the configurational stability of a sample of β-bromo ester (*R*)-**4a** having 93% ee over a three-month period at –20 °C and room temperature, and uncovered that when stored at –20 °C, no racemisation was detectable. In contrast, the sample stored at room temperature showed partial racemisation with a drop in enantiomeric excess to 78% after the same period (see the ESI† for further information). This is a very significant finding as it shows that optically active benzylic β-bromo esters can be conveniently prepared using our method and stored for prolonged periods at –20 °C (for at least three months) without measurable racemisation.

**Scheme 4:**
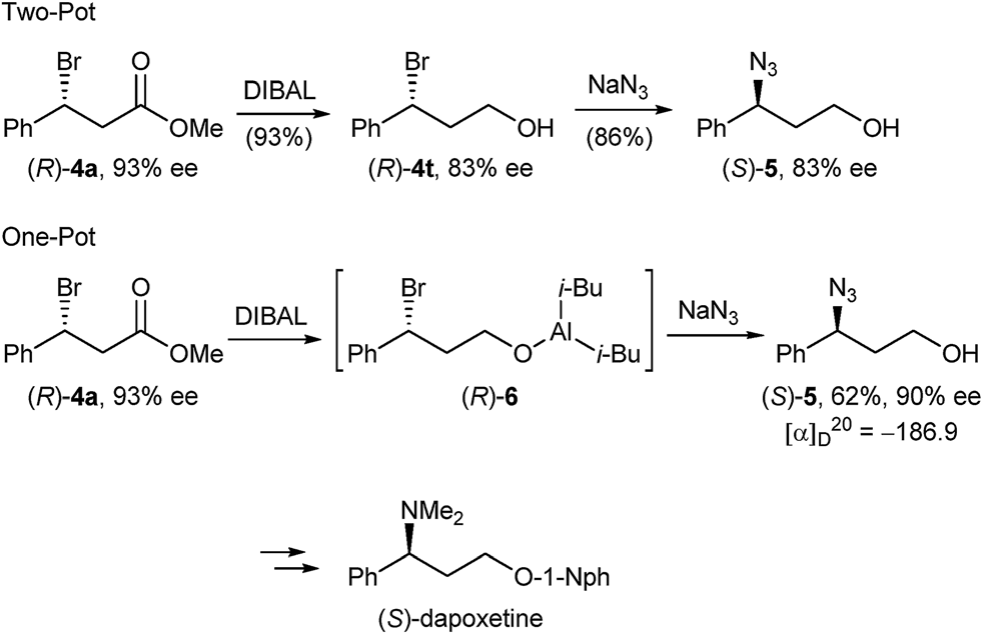
Preparation of enantiomerically enriched azido alcohol (*S*)-**5**.

In order to demonstrate the versatility of our enantiomerically enriched β-bromo esters and to corroborate that the desulphurative bromination occurred with the proposed inversion of configuration, we converted bromo ester (*R*)-**4a** in a reduction/azidation sequence into azido alcohol (*S*)-**5** (Scheme 4). After examining several reducing reagents, we discovered that DIBAL readily reduced the ester functionality in the presence of a benzylic bromide to afford bromo alcohol (*R*)-**4t** in an excellent 93% yield, but with a partially eroded optical purity of 83% ee (Scheme 4). We believe that this is due to the propensity of optically active bromo alcohol (*R*)-**4t** towards racemisation.^29^ Subsequent treatment of the crude product with sodium azide in DMF gave azido alcohol (*S*)-**5** in 86% yield and 83% ee.

Given that bromo alcohol (*R*)-**4t** is racemisation sensitive, we devised an alternative one-pot protocol by submitting the *in situ* generated bromo aluminium alcoholate (*R*)-**6** to sodium azide substitution and were delighted to obtain azido alcohol (*S*)-**5** in 62% yield and 90% ee. Comparing the sign of the specific optical rotation with that of the literature-known azido alcohol (*R*)-**5**^30^ confirmed that bromide **4a** was formed with an *R* absolute configuration and S_N_2 inversion from sulphide (*S*)-**1a**. Azido alcohol (*S*)-**5** can be considered an advanced intermediate en route to (*S*)-dapoxetine,^31^ thus showcasing that the herein described optically active β-bromo esters can serve as valuable intermediates in the transition metal free synthesis of a host of APIs having a benzylic chiral centre.

### NMR study and mechanism

As outlined in the Introduction, there is uncertainty surrounding the nature of the interaction of Br_2_ with sulphides, with very few studies reported in solution. In order to gain insight into the mechanism operating in the presented desulphurative bromination, we conducted a series of NMR experiments. They were undertaken in light of the fact that the reaction of both PhICl_2_ 32 and Cl_2_ 15b,^33^ with sulphides leads to the oxidation of the sulphur(ii) centre to sulphur(iv) forming dichlorosulphuranes.

**Fig. 1:**
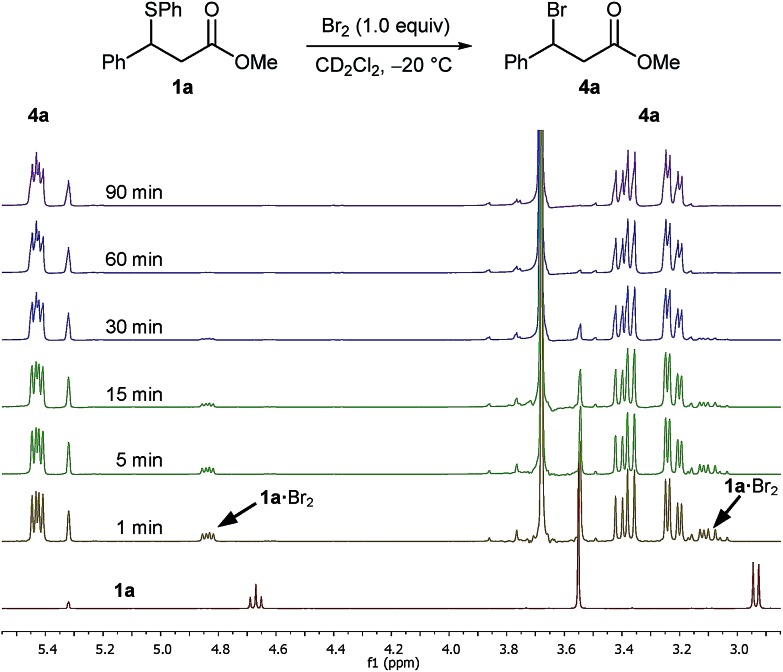
^1^H NMR spectra (400 MHz) of the reaction of Br_2_ (1.0 M) with sulphide **1a** (0.17 M) in CD_2_Cl_2_ at –20 °C over a period of 90 min.

In an initial experiment ran at an identical concentration to the actual reaction, we treated a 0.17 M solution of sulphide **1a** in CD_2_Cl_2_ pre-cooled to –20 °C with 1.0 equivalent of a 1.0 M solution of Br_2_ in CD_2_Cl_2_ and monitored the progress of the reaction by ^1^H NMR spectroscopy at –20 °C (Fig. 1). After one minute of Br_2_ addition, and focusing on the benzylic proton, the recorded spectrum showed the total absence of the benzylic signal of sulphide **1a** at 4.67 ppm with two new species appearing as double doublets at 5.43 ppm and 4.84 ppm in a ratio of 84 : 16. The major species was identified as bromo ester **4a**, with the minor unknown species converting to bromide **4a** upon further monitoring (Fig. 1). It was apparent that the new unknown species – postulated as being a sulphide bromine adduct intermediate **1a**·Br_2_ (*vide infra*) – converted rapidly to bromo ester **4a**, even at –20 °C.

**Fig. 2:**
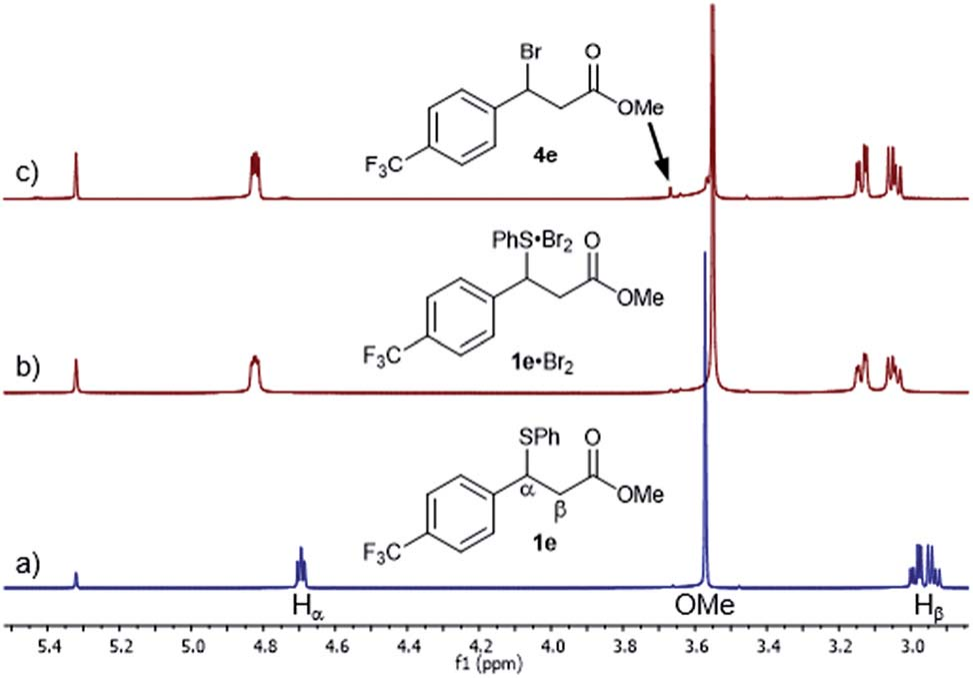
^1^H NMR spectra (800 MHz) of the Br_2_ (1.0 M) addition to sulphide **1e** (0.17 M) in CD_2_Cl_2_ at –20 °C: (a) sulphide **1e** at –20 °C before Br_2_ addition, (b) 2 minutes after Br_2_ addition and formation of the **1e**·Br_2_ adduct and (c) 1 hour after Br_2_ addition showing the appearance of bromide **4e**.

Given the high rate of bromination for **1a**, we turned our attention to the slow-reacting sulphide **1e** (Fig. 2). Thus, adding a 1.0 M solution of Br_2_ in CD_2_Cl_2_ to a pre-cooled 0.17 M solution of sulphide **1e** and recording a ^1^H NMR spectrum at –20 °C showed, after 2 min, the complete disappearance of **1e** and its conversion to a single new species, identified by the complete disappearance of the benzylic proton signal at 4.69 ppm and the appearance of a new double doublet at 4.82 ppm (Fig. 2b). Other significant shifts included downfield shifts of the diastereotopic H_β_ hydrogen atoms at 2.94 and 2.99 ppm to 3.05 and 3.14 ppm, respectively, and a downfield shift of the signal of hydrogens *ortho* to the sulphur by 0.14 ppm (not shown).

**Fig. 3:**
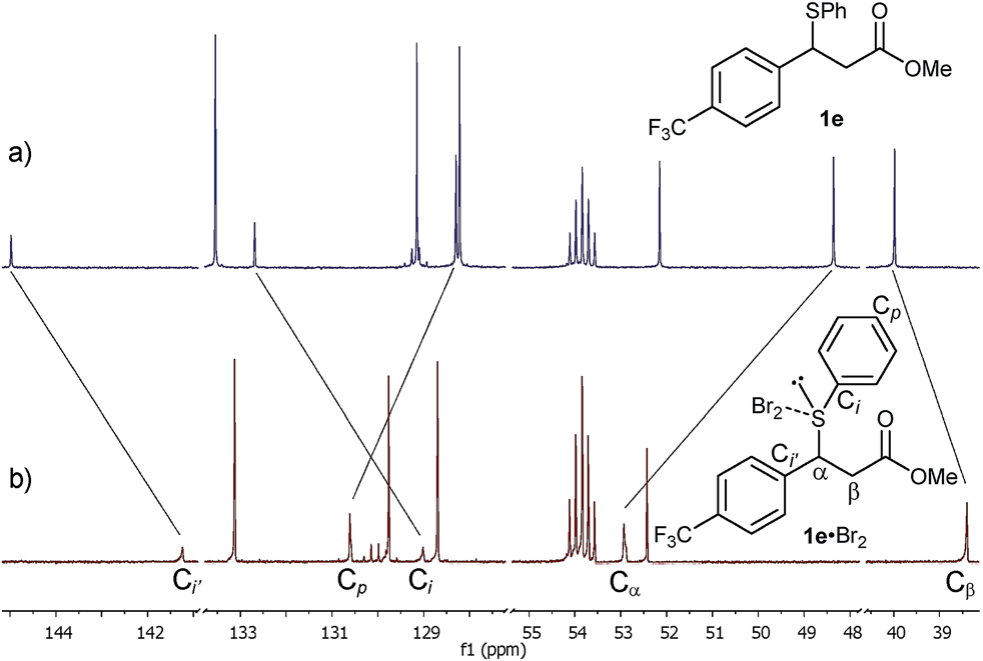
^13^C NMR spectra (201 MHz) of the Br_2_ (1.0 M) addition to sulphide **1e** (0.17 M) in CD_2_Cl_2_ at –20 °C: (a) sulphide **1e** at –20 °C before Br_2_ addition and (b) after Br_2_ addition, displaying significant shifts of the formed **1e**·Br_2_ adduct.

Gratifyingly, the new species was sufficiently stable at –20 °C to allow its full characterisation by 1D and 2D NMR spectroscopy. The ^13^C NMR spectrum showed significant shifts of signals compared to **1e** (Fig. 3). These included large shifts for carbons flanking the sulphur atom corresponding to an upfield shift of the resonance of the C_i_ of **1e** to 129.0 ppm (Δ*δ* = –3.7 ppm) and a downfield shift of the benzylic carbon C_α_ to 52.9 ppm (Δ*δ* = +4.6 ppm), as well as an upfield shift for C_i′_ to 141.2 ppm (Δ*δ* = –3.7 ppm) (Fig. 3b). In addition, notable shifts were observed for C_p_ of **1e** to 130.6 ppm (Δ*δ* = +2.3 ppm) and C_β_ to 38.4 ppm (Δ*δ* = –1.6 ppm).

All of the above ^1^H and ^13^C NMR Δ*δ*s are less pronounced at 20 °C.15*a* Hence, taking into account the NMR spectroscopic criteria proposed by Nakanishi to distinguish between MCs and TBs of R^1^R^2^S·X_2_ adducts in solution,15*b* the observed shifts – most notably the upfield shift of C_i_ of the SPh group – are generally consistent in terms of magnitude, direction and temperature behaviour with the presence of a MC structure for the **1e**·Br_2_ adduct.15*a* These findings provide, to the best of our knowledge, strong evidence for the first observation of a benzylic sulphide Br_2_ adduct in solution.

**Fig. 4:**
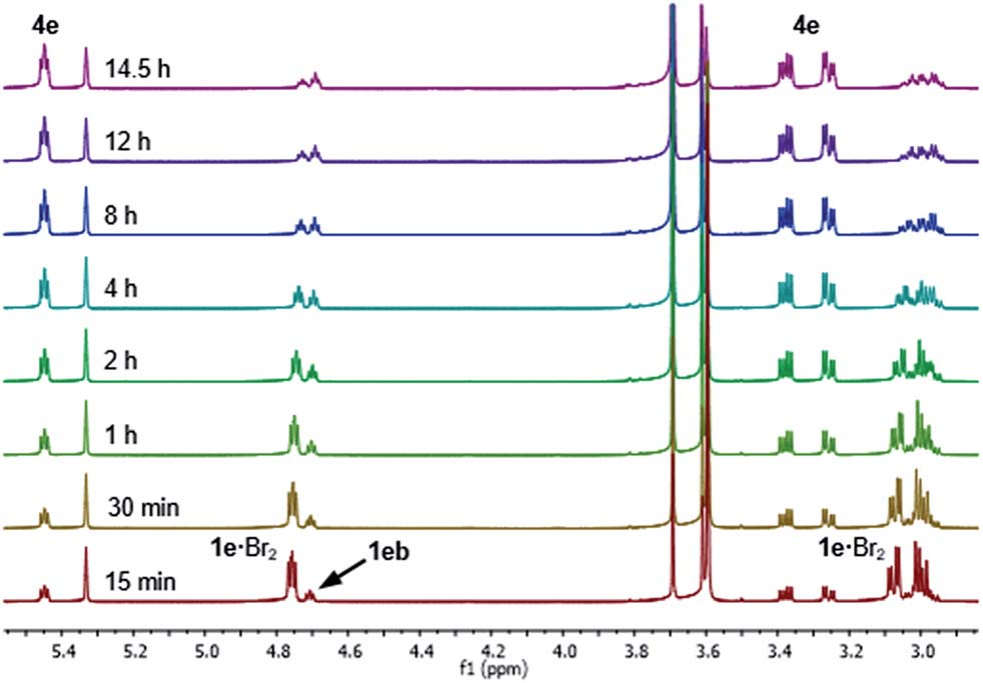
^1^H NMR spectra (800 MHz) of the Br_2_ (1.0 M) addition to sulphide **1e** (0.17 M) in CD_2_Cl_2_ after raising the temperature to 20 °C, showing the progressive conversion of the **1e**·Br_2_ adduct into bromide **4e** and the presence of side product sulphide **1eb**.

After 1 hour, signals assigned to bromide **4e** were visible, with further conversion to **4e** being very slow at –20 °C.^34^ We thus raised the temperature of the experiment to 20 °C and continued monitoring the progress of the reaction for a further 14.5 hours, and observed that **1e**·Br_2_ progressively converted to bromide **4e** (Fig. 4). Repeating this experiment at 20 °C for 60 h showed conversion of **1e**·Br_2_ to **4e** slowing markedly down to 87% after approximately 48 hours (see the ESI† for spectra). It is important to note that the actual bromination of **1e** was carried out at double the concentration (0.33 M) requiring 48 h for complete conversion to **4e**.

The reaction of **1e** also showed the formation of a side product observed with experiments at –20 °C and 20 °C, which started to form after the appearance of bromide **4e**. This side product was proposed as being sulphide **1eb** derived from the *para* bromination of the SPh group in **1e** (Fig. 4), which was confirmed after comparison with an authentic sample. In an NMR experiment conducted at 20 °C, sulphide **1eb** converted cleanly to bromide **4e** upon addition of 1.0 equivalent of Br_2_ (see the ESI† for spectra). However, the bromination was slower than that with sulphide **1e**, stopping at 82% conversion after 60 h, with complete conversion reached only after a further addition of 0.2 equivalents of Br_2_.

**Scheme 5:**
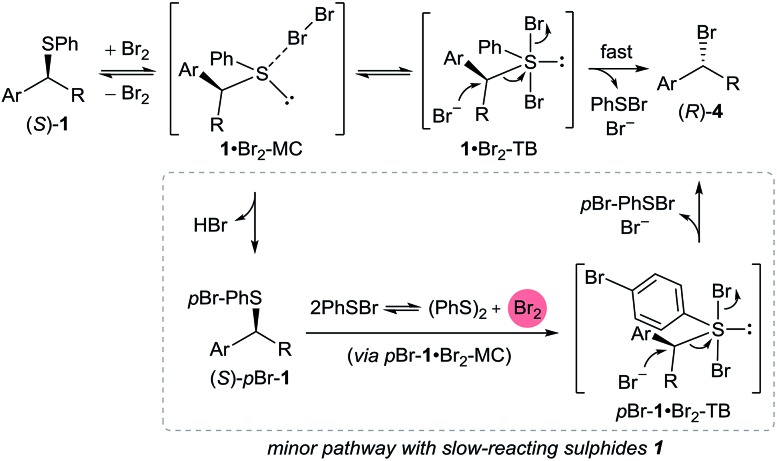
Proposed mechanism taking into account stereochemical and NMR evidence, as well as side-product formation.

A preliminary mechanistic picture is emerging from the above NMR experiments (Scheme 5). We propose that the addition of Br_2_ to sulphide (*S*)-**1** results in the formation of sulphur(ii) adduct **1**·Br_2_-MC. Based on the facts that (i) PhSBr is formed as a by-product and (ii) 1 equivalent of bromide ions must be generated from Br_2_ for S_N_2 C–Br bond formation to occur, we propose that **1**·Br_2_-MC must be in equilibrium with trigonal bipyramidal sulphur(iv) dibromosulphurane intermediate **1**·Br_2_-TB.^15^ However, due to its high reactivity, it is very likely that **1**·Br_2_-TB is present in very low concentrations. Invertive bromide ion attack on **1**·Br_2_-TB produces bromide (*R*)-**4** with concomitant release of PhSBr and one equivalent of bromide ions. Therefore, only catalytic amounts of bromide ions are required to initiate the reaction. Bromide ions could be derived from the ionisation of **1**·Br_2_-TB to form, in equilibrium, trigonal pyramidal bromosulphonium bromide [**1**-Br]Br. The fact that the reaction is significantly faster in coordinating MeCN (Table 1, entry 7) supports such a hypothesis.^35^

As seen with sulphide **1e**, slow-reacting sulphides can form the *para*-brominated side product (*S*)-*p*Br-**1**, presumably *via* reaction with generated **1**·Br_2_-TB or possibly PhSBr_3_ formed *in situ* (in equilibrium) from the generated PhSBr and Br_2_ from **1**·Br_2_-MC.^36^ The exclusive *para* bromination of activated aromatics with bromodimethylsulphonium bromide has been reported.17*b* Under optimised reaction conditions, the reductive coupling of two PhSBr molecules generates one molecule of Br_2_ that could oxidatively brominate (*S*)-*p*Br-**1** in an analogous manner to (*S*)-**1** (Scheme 5).^37^

Finally, a pathway involving n → σ* type activation of the C–S bond in **1**·Br_2_-MC towards bromide ion attack cannot be ruled out at present,^38^ and will be the subject of a detailed mechanistic analysis in the future.

## Conclusions

We have disclosed a novel nucleophilic bromination reaction that employs easily accessible alkyl aryl sulphides as starting materials and basic elemental Br_2_ as an oxidative brominating agent. Reaction conditions are exceptionally mild, allowing the isolation of otherwise difficult to access and highly versatile benzylic β-bromo esters and nitriles in generally good to excellent yields. The reaction tolerates various functionalities and, remarkably, proceeds in the presence of protic functionalities such as alkyl acids and alcohols; a transformation incompatible with the vast majority of deoxybromination procedures. Optically active benzylic β-sulphido esters could be converted into the corresponding inverted β-bromo esters with high stereoselectivities. These bromides are configurationally stable at –20 °C, which should pave the way for their exploitation as highly useful chiral synthons in organic and medicinal chemistry. Their utility was demonstrated by the preparation of γ-azido alcohol (*S*)-**5**, an advanced intermediate en route to dapoxetine, in 90% ee. The developed one-pot sequence from bromo ester (*R*)-**4a**, consisting of a DIBAL ester reduction in the presence of a benzylic bromide and subsequent invertive nucleophilic azidation, proceeded with high stereochemical fidelity. Significantly, the required stereochemistry was introduced into sulphide precursor (*S*)-**1a***via* an asymmetric sulpha-Michael reaction, thus bypassing the dependency on optically active benzylic alcohols.

Low temperature NMR spectroscopic studies pointed to an initial MC adduct formation between the starting sulphide and Br_2_ en route to the bromide product. This was observed for adducts **1a**·Br_2_ and **1e**·Br_2_, with the latter, derived from slow-reacting sulphide **1e**, being sufficiently stable at –20 °C to allow its full characterisation. Subsequent stereoinvertive C–Br bond formation is postulated to occur from the highly reactive isomeric dibromosulphurane TB adduct, proposed to be present in equilibrium with the MC adduct.

Given the very recent progress made in the synthesis of enantiomerically enriched benzylic aryl sulphides,^39^ we anticipate that the nucleophilic desulphurative bromination reported herein will find wide utility in the synthesis of optically active benzylic bromides relevant to drug discovery, agrochemicals, and materials.

Conceptually, the *in situ* oxidative activation of sulphides towards S_N_2 nucleophilic substitution should provide a platform for the development of other C–X and C–C bond forming reactions, and could prove to be a general, practical and viable alternative to oxygen based leaving groups.

## Conflicts of interest

There are no conflicts to declare.

## Supplementary Material

Supplementary information
