# Supplementary material for: Sulphide as a leaving group: highly stereoselective bromination of alkyl phenyl sulphides
Source: Chem Sci. 2019 Aug 16;10(39):9042–50. doi: 10.1039/c9sc03560e (PMC6889150; doi:10.1039/c9sc03560e)
Supplement: Supplementary file 1 [file SC-010-C9SC03560E-s001.pdf]

Electronic Supplementary Material (ESI) for Chemical Science.
This journal is © The Royal Society of Chemistry 2019

Sulphide as Leaving Group: Highly Stereoselective Bromination of Alkyl Phenyl Sulphides

Daniele Canestrari,[a] Caterina Cioffi,[a] Ilaria Biancofiore,[a,b] Stefano Lancianesi,[a] Lorenza Ghisu,[a] Manuel Ruether,[c] John O'Brien,[c] Mauro F. A. Adamo*[a] and Hasim Ibrahim*[a]

[a] *Centre for Synthesis and Chemical Biology (CSCB), Department of Chemistry, Royal College of Surgeons in Ireland, 123 St. Stephen's Green, Dublin 2, Ireland*

[b] *IRBM Science Park S.p.A., Department of Medicinal Chemistry, Via Pontina, 30.600, 00071 Pomezia RM, Italy*

[c] *Trinity Biomedical Sciences Institute, School of Chemistry, The University of Dublin, Trinity College, Dublin 2, Ireland*

email: madamo@rcsi.ie, hasimibrahim@rcsi.ie

Supporting Information

Table of Contents

1. General remarks 4

2. Materials 4

3. Reaction of sulphide (**1a**) with Cl_2 gas 5

4. Deoxybromination of β-hydroxyl esters 5

4.1 Dehydroxybromination of methyl 3-hydroxy-3-phenylpropanoate under Appel conditions 5

4.2 Deoxybromination of ethyl 3-(4-chlorophenyl)-3-hydroxypropanoate with PBr_3 6

5. Preparation of sulphides 8

5.1 General procedure for the reduction of β-sulphido esters (**GP1**) 8

5.2 General procedure for the preparation of β-sulphido mesylates (**GP2**) 9

5.3 General procedure for the sulpha-Michael addition to cinnamate esters (**GP3**) 10

5.4 Synthesis of 3-(4-chlorophenyl)-3-(phenylthio)propanenitrile (**1r**) 12

6. Bromination of sulphides with Br_2 13

6.1 General procedure for bromination of sulphides 13

6.2 Bromination followed by solvolysis with 2,2,2-trifluoroethanol 22

6.3 Bromination followed by mesylation of sulphide (**1u**) 23

6.4 Large scale bromination of β-sulphido ester (**1f**) 23

7. General procedure for the transesterification of enantiomerically enriched hexafluoroisopropyl esters to cinnamate methyl esters (**GP4**) 24

8. Optically active bromides 25

8.1. Bromination of enantiomerically enriched sulphides 25

8.2. Monitoring the racemisation of bromide (*R*)-**4a** 27

9. Synthesis of (–)-(*S*)-3-azido-3-phenylpropan-1-ol 27

9.1 Two-pot procedure 27

9.2 One-pot procedure 28

10. NMR studies 29

10.1. NMR monitoring of the bromination of (**1e**) 29

10.2. NMR monitoring of the bromination of sulphide (**1eb**) 37

11. References 42

12. HPLC traces of optically active compounds..44

13. Copies of ^{1}H NMR and ^{13}C NMR spectra 54

1. General remarks

^{1}H, ^{19}F and ^{13}C NMR spectra were recorded on a Bruker Avance 400 MHz spectrometer. Low-temperature 1D and 2D NMR spectra were recorded on an Agilent Technologies Ultra High Field (UHF) 800 MHz spectrometer or Agilent 400-MR Long Hold Mag. Res. spectrometer. Chemical shifts (δ) are reported in ppm relative to residual solvent signals for ^{1}H and ^{13}C NMR (^{1}H NMR: 7.26 ppm for $CDCl_3$, 1.94 ppm for CD_3CN, 5.32 ppm for CD_2Cl_2; ^{13}C NMR: 77.00 ppm for $CDCl_3$, 1.32 ppm and 118.26 ppm for CD_3CN, 53.84 ppm for CD_2Cl_2). ^{13}C NMR spectra were acquired with ^{1}H broad band decoupled mode. Coupling constants (*J*) are in Hz. Melting points were measured using a Stuart scientific melting point apparatus and are uncorrected. Infrared spectra (IR) were recorded using a Nicolet iS 10 FT-IR spectrometer. Low resolution mass spectra were acquired with an Advion Expression CMS instrument. High resolution mass spectra were obtained on a Waters Micromass GCT Premier MS spectrometer or on a Bruker micrOTOF-Q III LC-MS spectrometer (APCI method). Elemental analysis was carried out using an Exeter Analytical CE 440 elemental analyser. Optical rotations were measured on a Perkin-Elmer 343 polarimeter. The enantiomeric excess (ee) was determined by chiral stationary phase HPLC using a Shimadzu SIL-20AHT HPLC instrument.

2. Materials

Analytical grade solvents and commercially available reagents were used as received. Dry CH_2Cl_2, DCE, toluene, MeCN and Et_2O were purchased from commercial sources. Dry THF was obtained from an Inert Pure Solv Micro drying solvent system. Reactions were monitored by TLC analysis (Merck, aluminum plates, silica gel 60 F_{254}) and/or ^{1}H NMR spectroscopy. Flash column chromatography was performed using silica gel 60 (0.040-0.063 mm, 230-400 mesh). Cinnamate esters,[1] 1,1,1,3,3,3-hexafluoropropan-2-yl cinnamates and optically active sulphides (*S*)-**1aa**, (*S*)-**1ba**, (*S*)-**1da** and (*S*)-**1ea** were prepared according to published procedures.[2] Methyl 3-hydroxy-3-phenylpropanoate and ethyl 3-(4-chlorophenyl)-3-hydroxypropanoate,[3] sulphides **1a**, **1c**, **1d**, **1i**, **1m**, **1s**, **1t,** **1x**, **1za** and **1zb**,[4] **1j** and **1k**,[2] **1l**,[5] **1n** and **1o**,[6] **1p**,[7] **1q**[8] and **1y**[9] were synthesized according to literature procedures.

3. Reaction of sulphide (1a) with Cl_2 gas

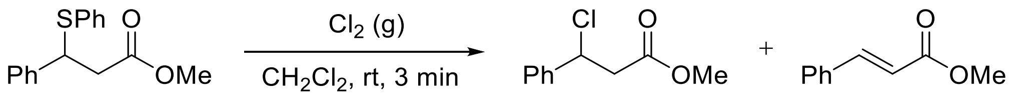

Cl_2 gas was bubbled into a stirred solution of **1a** (136 mg, 0.5 mmol) in dry CH_2Cl_2 (3 mL) (ca. 5 bubbles/sec) under N_2 atmosphere. The reaction was stirred for 3 minutes, quenched with styrene (86 µL, 78 mg, 0.75 mmol, 1.5 equiv) and the solvent removed *in vacuo*. A ^{1}H NMR spectrum of the residue was recorded to determine the composition of the crude material, which indicated the complete consumption of **1a** and the presence of chloride **2a** and dehydrochlorinated **3a** in a ratio of 70:30.

4. Deoxybromination of β-hydroxyl esters

4.1 Dehydroxybromination of methyl 3-hydroxy-3-phenylpropanoate under Appel conditions

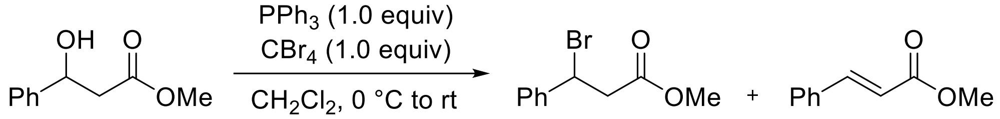

Standard conditions for the Appel reaction were followed.[10] A solution of methyl 3-hydroxy-3-phenylpropanoate (90 mg, 0.5 mmol) and tetrabromomethane (166 mg, 0.5 mmol, 1.0 equiv) in dry CH_2Cl_2 (5.0 mL) was cooled to 0 °C, and triphenylphosphine (131 mg, 0.5 mmol, 1.0 equiv) was added portion-wise. The reaction mixture was allowed to reach rt and stirred for 24 h (TLC analysis), then the reaction mixture was evaporated *in vacuo*. A ^{1}H NMR spectrum of the residue showed the formation of **4a** and **3a** in a ratio of 82:18.

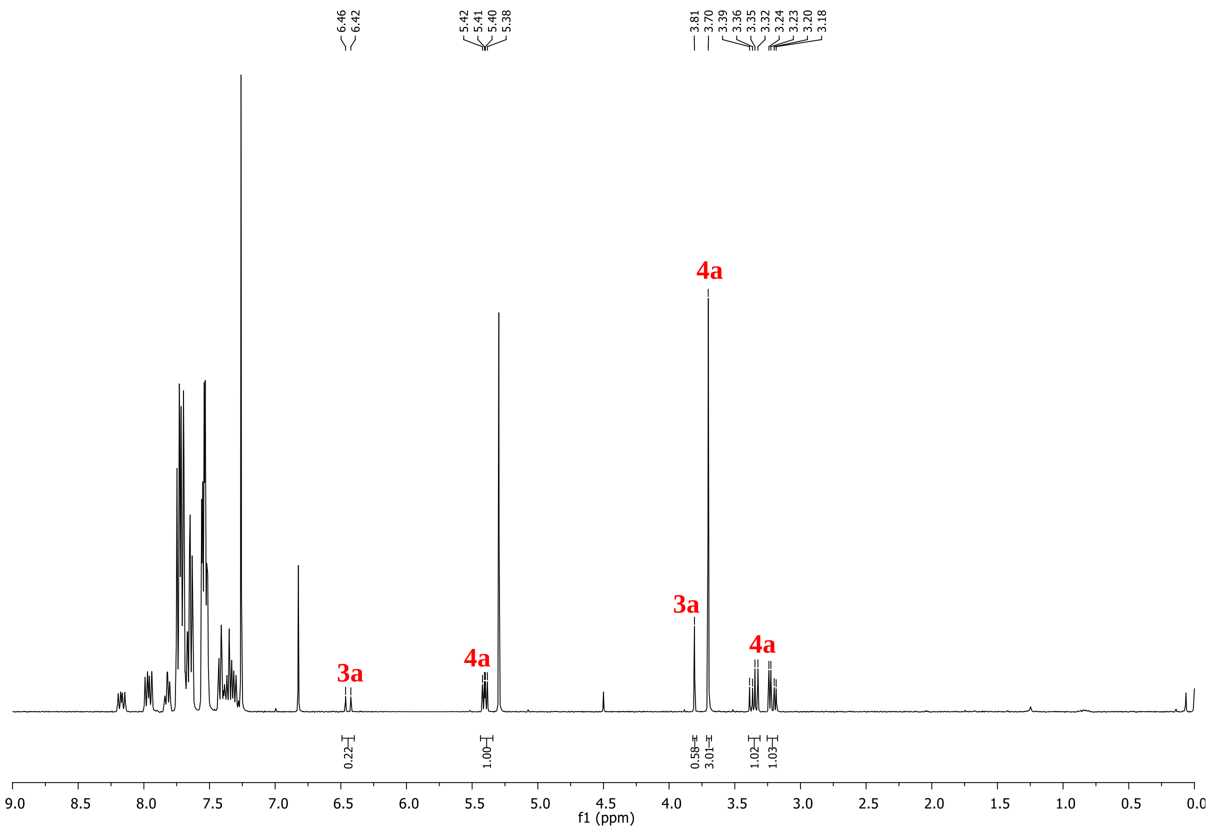

4.2 Deoxybromination of ethyl 3-(4-chlorophenyl)-3-hydroxypropanoate with PBr_3

The deoxybromination procedure with PBr_3 was repeated according to A. Sudalai *et al.*[11] In our hands, the ^{1}H NMR spectrum of the crude material showed the formation of a product mixture containing around 42% of bromide **4da**.

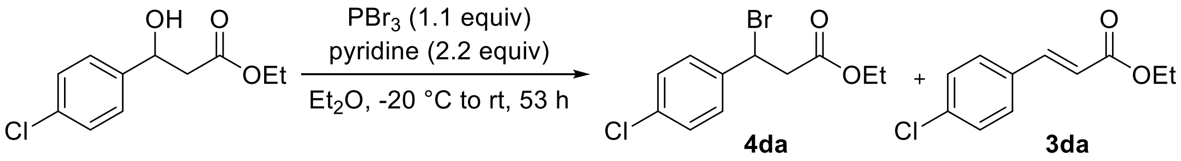

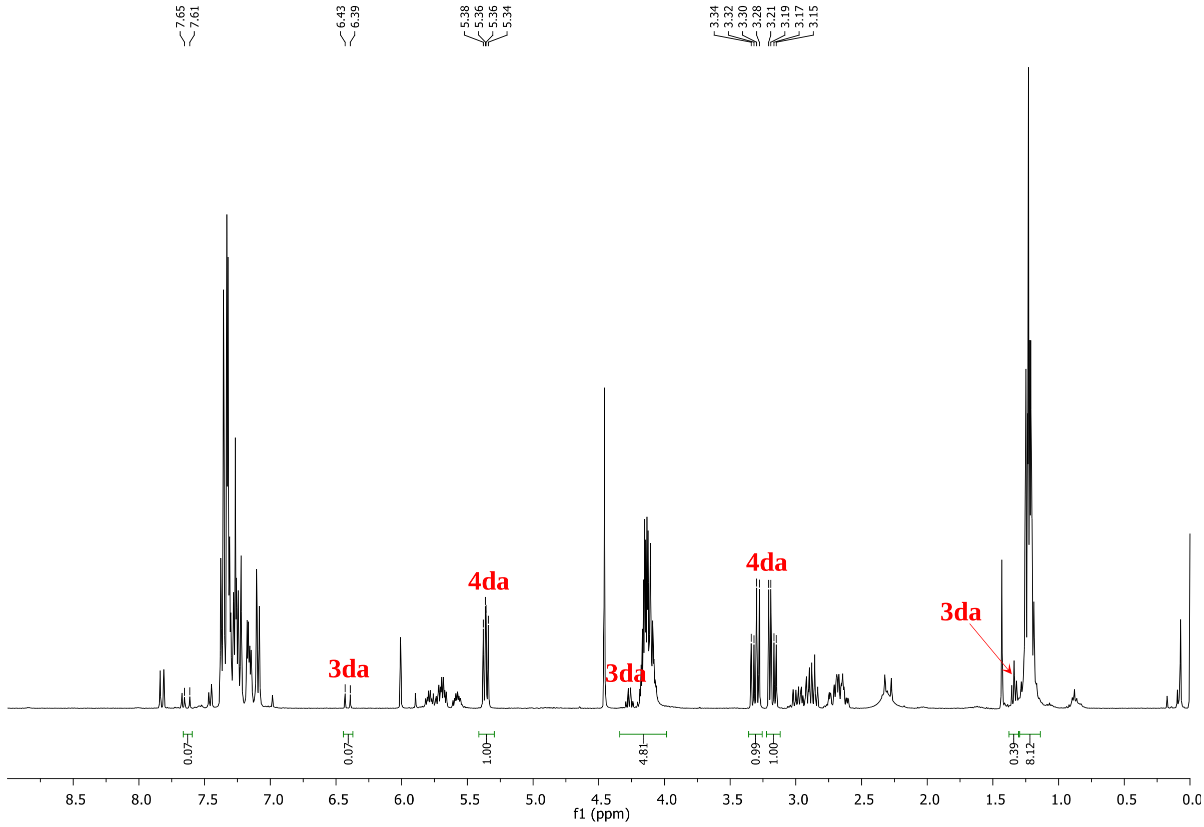
7.65
7.61
6.43
6.39
5.38
5.36
5.36
5.34
3.34
3.32
3.30
3.28
3.21
3.19
3.17
3.15
4da
3da
3da
4da
3da
0.07
0.07
1.00
4.81
0.99
1.00
0.39
8.12
8.5
8.0
7.5
7.0
6.5
6.0
5.5
5.0
4.5
4.0
3.5
3.0
2.5
2.0
1.5
1.0
0.5
f1 (ppm)

5. Preparation of sulphides

5.1 General procedure for the reduction of β-sulphido esters (GP1)

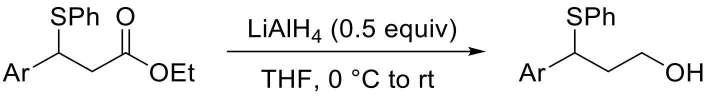

To a stirred solution of sulphide (2.0 mmol) in dry THF (4.0 mL) was added dropwise *via* syringe $LiAlH_4$ (1.0 mL, 1.0 M solution in Et_2O, 1.0 mmol, 0.5 equiv) at 0 °C. The reaction mixture was allowed to reach rt and stirred until consumption of the starting material was observed by TLC analysis, then carefully quenched with sat. aq. soln of NH_4Cl (10 mL) and extracted with Et_2O (3 x 10 mL). The combined organic layers were dried over Na_2SO_4 and the solvent removed *in vacuo* to obtain the corresponding alcohol, which was used without further purification.

3-(Phenylthio)-3-(thiophen-2-yl)propan-1-ol

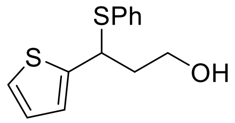

Prepared according to GP1. The reaction was completed in 2 h. The title compound was obtained as a yellow oil (425 mg, 85% yield). IR (neat, cm^{-1}): *ν* 3331, 3071, 2930, 1473, 1125, 1042, 954. ^{1}H NMR (400 MHz, $CDCl_3$): δ 1.79 (br s, 1H), 2.15–2.25 (m, 2H), 3.62–3.68 (m, 1H), 3.80 (dt, *J* = 11.3, 5.8 Hz, 1H), 4.65 (virt t, *J* = 7.6 Hz, 1H), 6.75 (d, *J* = 3.5 Hz, 1H), 6.82 (dd, *J* = 5.1, 3.5 Hz, 1H), 7.16 (dd, *J* = 5.1, 0.9 Hz, 1H), 7.19–7.26 (m, 2H), 7.27–7.32 (m, 3H). ^{13}C NMR (101 MHz, $CDCl_3$): δ 39.6, 45.4, 60.1, 124.5, 125.3, 126.3, 127.5, 128.7, 132.8, 134.0, 146.0. HRMS (EI): $C_{13}H_{14}OS_2Na$ $[M + Na]^+$ calculated: 273.0384, found: 273.0390.

3-(2,4,5-Trifluorophenyl)-3-(phenylthio)propan-1-ol (1u)

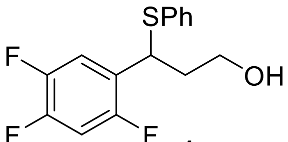

1u

Prepared according to GP1. The reaction was completed in 16 h. The title compound was isolated as a pale yellow oil (528 mg, 89% yield). IR (neat, cm^{-1}): ν 3487, 3362, 3100, 2856, 1469, 1123, 978. ^{1}H NMR (400 MHz, $CDCl_3$): δ 1.46 (br s, 1H), 2.02–2.10 (m, 1H), 2.16–2.24 (m, 1H), 3.65 (virt dt, *J* = 11.3, 6.3 Hz, 1H), 3.81 (virt dt, *J* = 11.5, 5.9 Hz, 1H), 4.69 (virt t, *J* = 7.6 Hz, 1H), 6.82 (virt dt, *J* = 9.7, 6.6 Hz, 1H), 7.15 (ddd, *J* = 10.8, 8.7, 6.9 Hz, 1H), 7.20–7.30 (m, 5H). ^{19}F NMR (376 MHz, $CDCl_3$): δ –119.6 (dd, *J* = 15.2, 3.8 Hz), –134.7 (dd, *J* = 21.7, 3.8 Hz), –141.9 (dd, *J* = 21.7, 15.1 Hz). ^{13}C NMR (101 MHz, $CDCl_3$): δ 37.9, 41.8, 60.1,

105.3 (dd, J = 28.8, 20.7 Hz), 116.6 (dd, J = 19.9, 5.2 Hz), 125.7 (dt, J = 16.0, 4.6 Hz), 127.8, 128.9, 132.6, 133.3, 146.9 (ddd, J = 244.9, 12.6, 3.4 Hz), 148.8 (ddd, J = 251.2, 14.5, 12.6 Hz), 155.2 (ddd, J = 245.3, 9.3, 2.7 Hz). HRMS (EI): $C_{15}H_{13}F_3OSNa$ $[M + Na]^+$ calculated: 321.0537, found: 321.0525.

5.2 General procedure for the preparation of β-sulphido mesylates (GP2)[4]

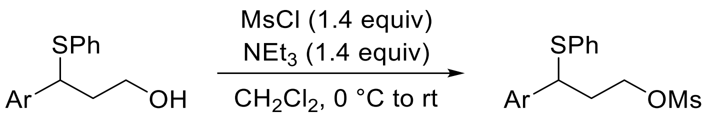

To a stirred solution of the sulphido alcohol (2.0 mmol) and methanesulphonyl chloride (260 μL, 321 mg, 2.8 mmol, 1.4 equiv) in CH_2Cl_2 (5.0 mL) was added dropwise triethylamine (467 μL, 339 mg, 3.4 mmol, 1.4 equiv) at 0 °C. The reaction mixture was allowed to reach rt and stirred until consumption of the starting material was observed by TLC analysis, then diluted with H_2O (10 mL) and extracted with CH_2Cl_2 (3 x 10 mL). The combined organic layers were washed with H_2O (3 x 10 mL), brine and dried over Na_2SO_4. The solvent was removed *in vacuo* and the crude mixture was purified by flash column chromatography on silica gel to afford the corresponding methanesulphonate.

(2,4,5-Trifluorophenyl)-3-(phenylthio)propyl methansulphonate (1v)

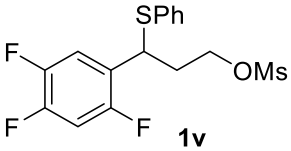

1v

Prepared according to GP2. The reaction was completed in 16 h. The title compound was isolated by flash column chromatography (silica gel; petroleum ether/EtOAc, 80:20) as a brown oil (568 mg, 91% yield). IR (neat, cm^{-1}): ν 3029, 3015, 2659, 1629, 1510, 1353, 1331, 1170, 797, 525. ^{1}H NMR (400 MHz, $CDCl_3$): δ 2.21–2.30 (m, 1H), 2.32–2.41 (m, 1H), 2.96 (s, 3H), 4.20 (ddd, J = 10.3, 6.4, 5.7 Hz, 1H), 4.39 (ddd, J = 10.3, 6.7, 5.6 Hz, 1H), 4.58 (virt t, J = 7.6, 1H), 6.86 (virt dt, J = 9.7, 6.6 Hz, 1H), 7.09 (ddd, J = 10.7, 8.6, 6.8 Hz, 1H), 7.21–7.31 (m, 5H). ^{19}F NMR (376 MHz, $CDCl_3$): δ –119.1 (dd, J = 15.2, 3.2 Hz), –133.8 (dd, J = 21.6, 2.0 Hz), –141.4 (dd, J = 26.3, 10.5 Hz). ^{13}C NMR (101 MHz, $CDCl_3$): δ 34.4, 37.3, 41.5, 66.7, 105.6 (dd, J = 28.7, 20.8 Hz), 116.5 (dd, J = 20.0, 5.0 Hz), 124.6 (dd, J = 15.8, 4.6 Hz), 128.2, 129.1, 132.4, 133.1, 146.9 (ddd, J = 245.6, 12.6, 3.5 Hz), 149.1 (ddd, J = 252.0, 14.5, 12.6 Hz), 155.2 (ddd, J = 246.0, 9.3, 2.6 Hz). HRMS (EI): $C_{16}H_{15}F_3O_3S_2Na$ $[M + Na]^+$ calculated: 399.0312, found: 399.0309.

3-(Phenylthio)-3-(thiophen-2-yl)propyl methanesulphonate (1w)

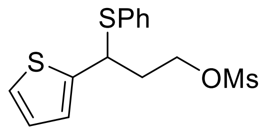

1w

Prepared according to GP-2. The reaction was completed in 3 h. The title compound was isolated by flash column chromatography (silica gel; CH_2Cl_2) as a yellow oil (636 mg, 97% yield). IR (neat, cm^{-1}): *v* 3020, 2935, 1566, 1351, 1169, 961. ^{1}H NMR (400 MHz, $CDCl_3$): δ 2.28-2.49 (m, 2H), 2.94 (s, 3H), 4.22 (ddd, *J* = 10.1, 7.3, 5.2 Hz, 1H), 4.40 (virt dt, *J* = 10.2, 5.8 Hz, 1H), 4.59 (virt t, *J* = 7.2 Hz, 1H), 6.78 (d, *J* = 3.2 Hz, 1H), 6.86 (dd, *J* = 5.1, 3.5 Hz, 1H), 7.21 (dd, *J* = 5.1, 0.8 Hz, 1H), 7.23–7.28 (m, 3H), 7.28–7.38 (m, 2H). ^{13}C NMR (101 MHz, $CDCl_3$): δ 36.2, 37.2, 44.9, 67.2, 125.0, 125.8, 126.6, 128.1, 128.9, 133.2, 133.3, 144.5. HRMS (EI): $C_{14}H_{16}O_3S_3Na$ $[M + Na]^+$ calculated: 351.0156, found: 351.0151.

5.3 General procedure for the sulpha-Michael addition to cinnamate esters (GP3)

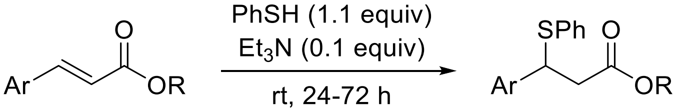

Thiophenol (337 μL, 364 mg, 3.3 mmol, 1.1 equiv) and triethylamine (42 μL, 30 mg, 0.3 mmol, 0.1 equiv) were added to the cinnamate ester (3.0 mmol) and the reaction mixture was stirred at rt until complete consumption of the starting material was observed by TLC analysis (9-48 h). The reaction mixture was purified directly by flash column chromatography on silica gel to afford the sulpha-Michael adduct.

Methyl 3-(phenylthio)-3-(*o*-tolyl)propanoate (1b)

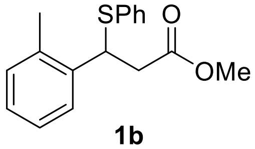

1b

Prepared according GP3. The reaction was completed in 16 h. The title compound was isolated by flash chromatography (silica gel; petroleum ether/EtOAc, 95:5) as a pale yellow oil (566 mg, 66% yield). IR (neat, cm^{-1}): *v* 3025, 2925, 2910, 1787, 1599, 1503, 1271, 759, 651. ^{1}H NMR (400 MHz, $CDCl_3$): δ 2.40 (s, 3H), 2.98 (m, 2H), 3.55 (s, 3H), 4.87 (virt t, *J* = 7.7 Hz, 1H), 7.09-7.18 (m, 4H), 7.22-7.27 (m, 3H), 7.28-7.35 (m, 2H). ^{13}C NMR (101 MHz, $CDCl_3$): δ 19.4, 40.2, 44.6, 51.8, 126.2, 126.3, 127.4, 128.0, 128.8, 130.5, 133.4, 133.9, 136.1, 138.1, 171.4. HRMS (EI): $C_{17}H_{18}O_2SNa$ $[M + Na]^+$ calculated: 309.0931, found: 309.0925.

Methyl 3-(phenylthio)-3-[4-(trifluoromethyl)phenyl]propanoate (1e)

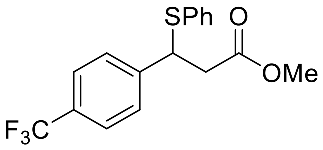

1e

Prepared according to GP3. Reaction was completed in 16 h. The title compound was isolated by flash column chromatography (silica gel; petroleum ether/EtOAc, 95:5) as a white solid (989 mg, 97% yield). Mp: 91–93 °C. IR (neat, cm^{-1}): ν 3019, 2854, 1710, 1564, 1389, 1354, 1015, 758. ^{1}H NMR (400 MHz, $CDCl_3$) δ 2.92 (dd, *J* = 16.0, 8.6 Hz, 1H), 3.00 (dd, *J* = 16.0, 6.9 Hz, 1H), 3.61 (s, 3H), 4.66 (dd, *J* = 8.3, 7.2 Hz, 1H), 7.21-7.31 (m, 5H), 7.34 (d, *J* = 8.1 Hz, 2H), 7.51 (d, *J* = 8.2 Hz, 2H). ^{19}F NMR (376 MHz, $CDCl_3$): δ –62.5. ^{13}C NMR (101 MHz, $CDCl_3$): δ 40.3, 48.7, 51.9, 124.02 (q, *J* = 272.0 Hz), 125.4 (q, *J* = 3.8 Hz), 128.0, 128.2, 129.0, 129.6 (q, *J* = 32.4 Hz), 132.7, 133.6, 144.7, 170.8. HRMS (EI): $C_{17}H_{15}F_3O_2SNa$ [M + Na]$^+$: calculated: 363.0643, found: 363.0658.

Methyl 3-(4-formylphenyl)-3-(phenylthio)propanoate (1f)

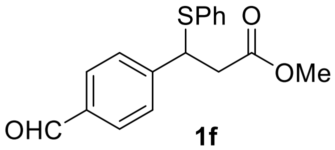

Prepared according to GP3. The reaction was completed in 24 h. The title compound was isolated by flash column chromatography (silica gel; CH_2Cl_2) as a pale yellow oil (792 mg, 88% yield). IR (neat, cm^{-1}): ν 3019, 2979, 2359, 1730, 1211, 1674, 689. ^{1}H NMR (400 MHz, $CDCl_3$): δ 2.94 (dd, *J* = 16.0, 8.5 Hz, 1H), 3.02 (dd, *J* = 16.0, 7.0 Hz, 1H), 3.61 (s, 3H), 4.67 (dd, *J* = 8.3, 7.2 Hz, 1H), 7.19–7.31 (m, 5H), 7.37 (d, *J* = 8.2 Hz, 2H), 7.77 (d, *J* = 8.2 Hz, 2H), 9.95 (s, 1H). ^{13}C NMR (101 MHz, $CDCl_3$): δ 40.0, 48.9, 51.9, 128.2, 128.3, 128.9, 129.8, 132.5, 133.8, 135.5, 147.5, 170.7, 191.6. HRMS (EI): $C_{17}H_{16}O_3SNa$ [M + Na]$^+$: calculated: 323.0718, found: 323.0720.

Methyl 3-(3-nitrophenyl)-3-(phenylthio)propanoate (1g)

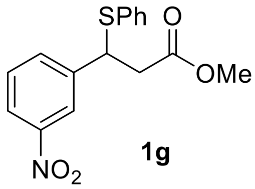

Prepared according to GP3. The reaction was completed in 22 h. The title compound was isolated by flash column chromatography (silica gel; petroleum ether/EtOAc 90:10) as a white solid (718 mg, 84% yield). Mp: 67–69 °C. IR (neat, cm^{-1}): ν 3067, 2961, 2924, 1722, 1578, 1470, 1352, 1218, 1147, 987, 753. ^{1}H NMR (400 MHz, $CDCl_3$): δ 2.95 (dd, *J* = 16.2, 8.8 Hz, 1H), 3.04 (dd, *J* = 16.2, 6.7 Hz, 1H), 3.62 (s, 3H), 4.70 (dd, *J* = 8.7, 6.8 Hz, 1H), 7.20–7.34 (m, 5H), 7.41–7.45 (m, 1H), 7.54–7.56 (m, 1H), 8.05–8.10 (m, 2H). ^{13}C NMR (101 MHz, $CDCl_3$): δ 40.1, 48.5, 52.0, 122.5, 128.5, 129.1, 129.3, 132.2, 133.9, 133.9, 142.9, 170.6. HRMS (EI): $C_{16}H_{15}NO_4SNa$ [M + Na]$^+$ calculated: 340.0612, found: 340.0619.

Ethyl 3-[(3,5-bis(trifluoromethyl)phenyl)]-3-(phenylthio)propanoate (1h)

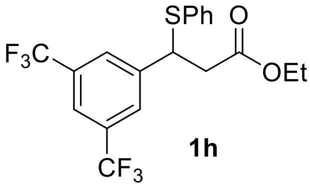

1h

Prepared according to GP3. Reaction was completed in 48 h. The title compound was isolated by flash column chromatography (silica gel; petroleum ether/CH_2Cl_2, 80:20) as a pale yellow oil (974 mg, 77% yield). IR (neat, cm^{-1}): *ν* 3001, 2983, 1732, 1275, 1168, 1125, 681. ^1H NMR (400 MHz, $CDCl_3$): δ 1.17 (t, *J* = 7.1 Hz, 3H), 2.92 (dd, *J* = 16.0, 8.8 Hz, 1H), 3.04 (dd, *J* = 16.0, 6.8 Hz, 1H), 4.02–4.13 (m, 2H), 4.68 (dd, *J* = 8.8, 6.8 Hz, 1H), 7.20–7.34 (m, 5H), 7.57 (s, 2H), 7.71 (s, 1H). ^{19}F NMR (376 MHz, $CDCl_3$): δ –62.9. ^{13}C NMR (101 MHz, $CDCl_3$): δ 13.9, 40.0, 48.6, 61.1, 121.3 (hept, *J* = 3.7 Hz), 123.1 (q, *J* = 272.8 Hz), 127.8 (q, *J* = 2.8 Hz), 128.8, 129.1, 131.5 (q, *J* = 33.4 Hz), 131.6, 134.4, 143.4, 169.9. HRMS (EI): $C_{19}H_{16}F_6O_2SNa$ $[M + Na]^+$ calculated: 445.0673, found: 445.0670.

Ethyl 3-(phenylthio)-3-(thiophen-2-yl)propanoate

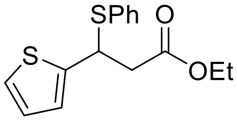

Prepared according to GP3. The reaction was completed in 9 h. The title compound was isolated by flash column chromatography (silica gel; petroleum ether/EtOAc, 95:5) as a yellow oil (900 mg, 56% yield). IR (neat, cm^{-1}): *ν* 3010, 2979, 1730, 1211, 1146, 1024, 689. ^1H NMR (400 MHz, $CDCl_3$): δ 1.20 (t, *J* = 7.1 Hz, 3H), 2.94 (dd, *J* = 16.2, 8.0 Hz, 1H), 2.98 (dd, *J* = 16.5, 7.6 Hz, 1H), 4.01-4.19 (m, 2H), 4.92 (virt t, *J* = 7.6 Hz, 1H), 6.78 (d, *J* = 3.3 Hz, 1H), 6.83–6.85 (m, 1H), 7.18 (dd, *J* = 5.1, 1.0 Hz, 1H), 7.23–7.30 (m, 3H), 7.31–7.39 (m, 2H). ^{13}C NMR (101 MHz, $CDCl_3$): δ 14.1, 42.0, 44.6, 60.9, 124.7, 125.3, 126.4, 128.1, 128.9, 133.1, 133.6, 144.6, 170.4. HRMS (EI): $C_{15}H_{16}O_2S_2Na$ $[M + Na]^+$ calculated: 315.0481, found: 315.0489.

5.4 Synthesis of 3-(4-chlorophenyl)-3-(phenylthio)propanenitrile (1r)

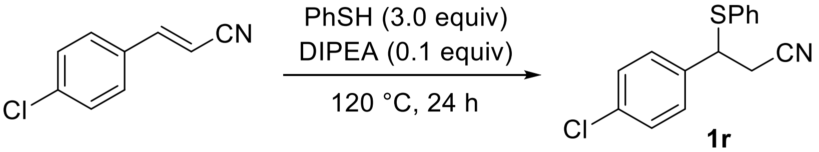

1r

Thiophenol (919 μL, 990 mg, 9.0 mmol, 3.0 equiv) and DIPEA (52 μL, 39 mg, 0.3 mmol, 0.1 equiv) were added to 3-(4-chlorophenyl)acrylonitrile (489 mg, 3.0 mmol) and the reaction mixture was stirred at 120 °C until complete consumption of the starting material was observed by TLC analysis (24 h). The reaction mixture was directly purified by flash chromatography (silica gel; petroleum ether/EtOAc 95:5) to afford the title compound as a white solid (779 mg,

95% yield). Mp: 80–81 °C. IR (neat, cm^{-1}): *ν* 2979, 1730, 1489, 1211, 1146, 1024, 689. ^1H NMR (400 MHz, $CDCl_3$) δ 2.84 (dd, *J* = 16.6, 8.1 Hz, 1H), 2.90 (dd, *J* = 16.6, 5.9 Hz, 1H), 4.38 (dd, *J* = 8.1, 5.9 Hz, 1H), 7.24–7.39 (m, 9H). ^{13}C NMR (101 MHz, $CDCl_3$) δ 24.9, 48.6, 116.9, 128.8, 128.9, 129.1, 129.3, 131.9, 133.9, 134.3, 136.8. HRMS (EI): $C_{15}H_{12}ClNSNa$ $[M + Na]^+$ calculated: 296.0277, found: 296.0275.

6. Bromination of sulphides with Br_2

6.1 General procedure for bromination of sulphides

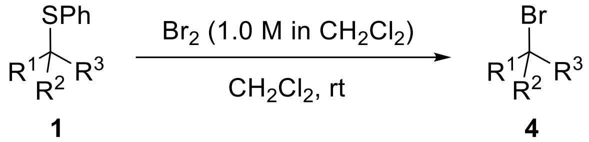

To a stirred solution of sulphide **1** (0.5 mmol) in dry CH_2Cl_2 (3.0 mL) was added Br_2 (500 μL, 1.0 M in CH_2Cl_2, 0.5 mmol, 1.0 equiv) at rt under N_2 atmosphere and the reaction progress was monitored by ^1H NMR analysis using a stock solution of styrene in $CDCl_3$ (0.03 M). After the consumption of the starting material (4 min-48 h), the reaction mixture was quenched by either the addition of styrene (68 μL, 0.6 mmol, 1.2 equiv) or a sat. aq. soln of $Na_2S_2O_3$, which led to an immediate fading of the red colour. After the evaporation of the solvents or aqueous work up, the residue was purified by flash column chromatography to afford the corresponding bromide.

Products that precipitated from the reaction mixture were purified by either recrystallization or trituration.

An analogous procedure was followed for slow-reacting substrates (sulphides **1g-i**, **1o**) using a 1.0 M Br_2 solution in DCE, DCE as solvent and a reaction temperature of 50 °C.

Methyl 3-bromo-3-phenylpropanoate (4a)

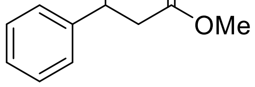

4a

Reaction was quenched with styrene after 25 min. The title compound was isolated by flash column chromatography (silica gel; petroleum ether/EtOAc 95:5) as a yellow oil (115 mg, 95% yield). All analytical data are consistent with those reported in the literature.[12]

Methyl 3-bromo-3-(*o*-tolyl)propanoate (4b)

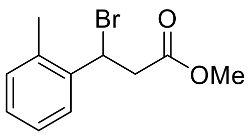

4b

Reaction was quenched with styrene after 25 min. The title compound was isolated by flash column chromatography (silica gel; petroleum ether/EtOAc, 96:4) as a yellow oil (112 mg, 87% yield). IR (neat, cm^{-1}): *ν* 3011, 2951, 1736, 1435, 1200, 761, 627. ^{1}H NMR (400 MHz, $CDCl_3$): δ 2.45 (s, 3H), 3.26 (dd, *J* = 16.4, 6.0 Hz, 1H), 3.43 (dd, *J* = 16.4, 9.0 Hz, 1H), 3.70 (s, 3H), 5.64 (dd, *J* = 9.0, 6.0 Hz, 1H), 7.13–7.27 (m, 3H), 7.39–7.45 (m, 1H). ^{13}C NMR (101 MHz, $CDCl_3$): δ 19.1, 43.5, 44.3, 52.1, 126.1, 126.7, 128.6, 130.8, 135.7, 138.5, 170.3. HRMS (APCI): $C_{11}H_{14}{}^{79}BrO_2$ $[M + H]^+$ calculated: 257.0177, found: 257.0179. Anal. Calcd for $C_{11}H_{13}BrO_2$: C, 51.38; H, 5.10. Found: C, 51.31; H, 5.13.

Ethyl 3-bromo-3-(*p*-tolyl)propanoate (4c)

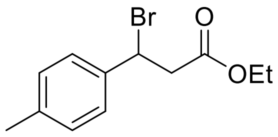

4c

Reaction was quenched with styrene after 25 min. The title compound was isolated by flash column chromatography (silica gel; petroleum ether/EtOAc, 97:3) as a yellow oil (115 mg, 84% yield). IR (neat, cm^{-1}): *ν* 2924, 1734, 1374, 1184, 1018, 734, 515. ^{1}H NMR (400 MHz, $CDCl_3$): δ 1.24 (t, *J* = 7.1 Hz, 3H), 2.34 (s, 3H), 3.19 (dd, *J* = 16.1, 6.2 Hz, 1H), 3.34 (dd, *J* = 16.1, 9.0 Hz, 1H), 4.06–4.25 (m, 2H), 5.41 (dd, *J* = 9.0, 6.2 Hz, 1H), 7.16 (d, *J* = 8.1 Hz, 2H), 7.32 (d, *J* = 8.1 Hz, 2H). ^{13}C NMR (101 MHz, $CDCl_3$): δ 14.1, 21.1, 44.8, 48.2, 60.1, 127.0, 129.4, 137.8, 138.7, 169.7. HRMS (APCI): $C_{12}H_{16}{}^{79}BrO_2$ $[M + H]^+$ calculated: 271.0334, found: 271.0347. Anal. Calcd for $C_{12}H_{15}BrO_2$: C, 53.16; H, 5.58. Found: C, 53.11; H, 5.50.

Methyl 3-bromo-3-(4-chlorophenyl)propanoate (4d)

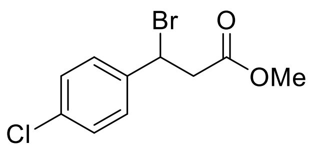

4d

Reaction was quenched with styrene after 25 min. The title compound was isolated by flash column chromatography (silica gel; petroleum ether/EtOAc, 96:4) as a yellow oil (135 mg, 97% yield). All analytical data are consistent with those reported in the literature.[13]

Methyl 3-bromo-3-(4-(trifluoromethyl)phenyl)propanoate (4e)

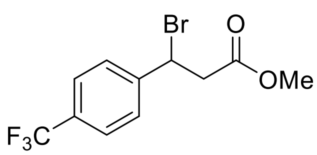

4e

The reaction was performed at double concentration (1.5 mL of dry CH_2Cl_2) and quenched with styrene after 48 h. The title compound was isolated by flash chromatography (silica gel; petroleum ether/CH_2Cl_2, 60:40) as a yellow oil (148 mg, 95% yield). IR (neat,

cm^{-1}): *v* 2955, 1736, 1320, 1110, 1068, 630. ^{1}H NMR (400 MHz, $CDCl_3$): δ 3.22 (dd, *J* = 16.4, 6.7 Hz, 1H), 3.35 (dd, *J* = 16.4, 8.5 Hz, 1H), 3.70 (s, 3H), 5.41 (dd, *J* = 8.5, 6.7 Hz, 1H), 7.55 (d, *J* = 8.3 Hz, 2H), 7.62 (d, *J* = 8.3 Hz, 2H). ^{19}F NMR (376 MHz, $CDCl_3$): δ –62.6. ^{13}C NMR (101 MHz, $CDCl_3$): δ 44.3, 46.0, 52.1, 123.7 (q, *J* = 272.2 Hz), 125.8 (q, *J* = 3.8 Hz), 127.6, 130.7 (q, *J* = 32.7 Hz), 144.5, 169.7. HRMS (APCI): $C_{11}H_{11}{}^{79}BrFO_2$ [M + H]$^+$ calculated: 310.9883, found: 310.9889.

Methyl 3-bromo-3-(4-formylphenyl)propanoate (4f)

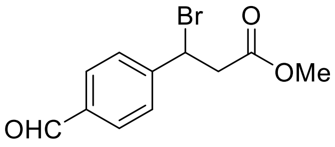

4f

The reaction was performed at double concentration (1.5 mL of dry CH_2Cl_2) and quenched with styrene after 4 h. The title compound was isolated by flash column chromatography (silica gel; CH_2Cl_2) as a yellow oil (123 mg, 90% yield). IR (neat, cm^{-1}): *v* 2922, 1721, 1690, 1211, 1156, 855, 527. ^{1}H NMR (400 MHz, $CDCl_3$): δ 3.25 (dd, *J* = 16.4, 6.7 Hz, 1H), 3.36 (dd, *J* = 16.4, 8.5 Hz, 1H), 3.70 (s, 3H), 5.42 (dd, *J* = 8.3, 6.9 Hz, 1H), 7.60 (d, *J* = 8.2 Hz, 2H), 7.87 (d, *J* = 8.2 Hz, 2H), 10.01 (s, 1H). ^{13}C NMR (101 MHz, $CDCl_3$): δ 44.1, 46.1, 52.1, 127.9, 130.1, 136.3, 146.9, 169.6, 191.3. HRMS (APCI): $C_{11}H_{12}{}^{79}BrO_3$ [M + H]$^+$ calculated: 270.9972, found: 270.9964. Anal. Calcd for $C_{11}H_{11}BrO_3$: C, 48.73; H, 4.22. Found: C, 48.60; H, 4.05.

Methyl 3-bromo-3-(3-nitrophenyl)propanoate (4g)

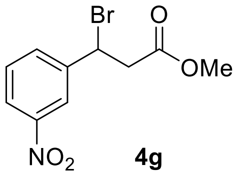

4g

The reaction was performed in dry DCE (3.0 mL) at 50 °C, using Br_2 (0.5 mL, 1.0 M in DCE, 0.5 mmol, 1.0 equiv) and quenched with styrene after 2 h. The title compound was isolated by flash column chromatography (silica gel; petroleum ether/CH_2Cl_2, 70:30) as a yellow solid (129 mg, 90% yield). Mp: 84-86 °C. IR (neat, cm^{-1}): *v* 3012, 2953, 1732, 1527, 1347, 1024, 683. ^{1}H NMR (400 MHz, $CDCl_3$): δ 3.26 (dd, *J* = 16.5, 7.0 Hz, 1H), 3.38 (dd, *J* = 16.5, 8.2 Hz, 1H), 3.71 (s, 3H), 5.43 (dd, *J* = 8.2, 7.0 Hz, 1H), 7.55 (t, *J* = 8.0 Hz, 1H), 7.76 (d, *J* = 7.8 Hz, 1H), 8.10–8.21 (m, 1H), 8.29 (t, *J* = 1.8 Hz, 1H). ^{13}C NMR (101 MHz, $CDCl_3$): δ 44.2, 45.1, 52.3, 122.2, 123.6, 129.9, 133.4, 142.8, 148.3, 169.5. HRMS (APCI): $C_{10}H_{11}{}^{79}BrNO_4$ [M + H]$^+$ calculated: 287.9874, found: 287.9866. Anal. Calcd for $C_{10}H_{10}BrNO_4$: C, 41.69; H, 3.53; N, 4.86. Found: C, 41.54; H, 3.53; N, 4.53.

Ethyl 3-[3,5-bis(trifluoromethyl)phenyl]-3-bromopropanoate (4h)

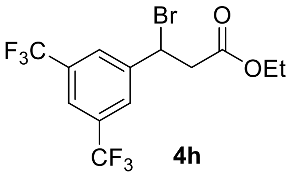

4h

The reaction was performed in dry DCE (3.0 mL) at 50 °C, using Br_2 (1.0 mL, 1.0 M in DCE, 1.0 mmol, 2.0 equiv) and quenched with styrene after 2 h. The title compound was isolated by flash column chromatography (silica gel; petroleum ether/CH_2Cl_2 70:30) as a colourless oil (175 mg, 89% yield). IR (neat, cm^{-1}): *ν* 3011, 2958, 2545, 1734, 1373, 1275, 1066, 727, 591. ^1H NMR (400 MHz, $CDCl_3$): δ 1.23 (t, *J* = 7.1 Hz, 3H), 3.23 (dd, *J* = 16.4, 7.1 Hz, 1H), 3.36 (dd, *J* = 16.4, 8.2 Hz, 1H), 4.08–4.23 (m, 2H), 5.43 (virt t, *J* = 7.6 Hz, 1H), 7.83 (s, 1H), 7.88 (s, 2H). ^{19}F NMR (376 MHz, $CDCl_3$): δ –62.9. ^{13}C NMR (101 MHz, $CDCl_3$): δ 14.0, 44.5, 44.7, 61.4, 122.6 (hept, *J* = 3.7 Hz), 122.9 (q, *J* = 272.9 Hz), 127.6, 132.3 (q, *J* = 33.6 Hz), 143.3, 168.8. LRMS (ESI): *m/z* (%) 393 (100) $[M]^+$. Anal. Calcd for $C_{13}H_{11}BrF_6O_2$: C, 39.72; H, 2.82. Found: C, 39.68; H, 2.89.

Ethyl 3-bromo-3-(2,4,5-trifluorophenyl)propanoate (4i)

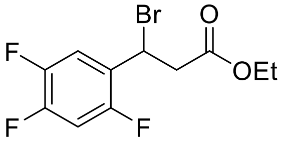

4i

The reaction was performed in dry DCE (3.0 mL) at 50 °C, using Br_2 (0.6 mL, 1.0 M in DCE, 0.6 mmol, 1.2 equiv), stirred for 16 h and then quenched with styrene. The title compound was isolated by flash column chromatography (silica gel; petroleum ether/CH_2Cl_2 70:30) as a pale yellow oil (578 mg, 93% yield). IR (neat, cm^{-1}): *ν* 2921, 2852, 1734, 1514, 1336, 1110, 887, 527. ^1H NMR (400 MHz, $CDCl_3$): δ 1.24 (t, *J* = 7.1 Hz, 3H), 3.19 (dd, *J* = 16.4, 7.0 Hz, 1 H), 3.31 (dd, *J* = 16.4, 8.3 Hz, 1H), 4.10-4.23 (m, 2H), 5.56 (virt t, *J* = 7.7 Hz, 1H), 6.94 (virt dt, *J* = 9.7, 6.5 Hz, 1H), 7.29 (ddd, *J* = 10.3, 8.4, 6.8 Hz, 1H). ^{19}F NMR (376 MHz, $CDCl_3$): δ –117.1 (dd, *J* = 15.1, 4.8 Hz), –131.8 (dd, *J* = 21.4, 4.8 Hz), –140.9 (dd, *J* = 21.4, 15.1 Hz). ^{13}C NMR (101 MHz, $CDCl_3$): δ 14.0, 38.1 (d, *J* = 2.9 Hz), 43.6, 61.3, 106.1 (dd, *J* = 28.0, 20.9 Hz), 116.6 (ddd, *J* = 20.2, 4.4, 1.5 Hz), 124.6 (ddd, *J* = 15.2, 7.1, 2.5 Hz), 146.9 (ddd, *J* = 246.3, 12.8, 3.6 Hz), 150.1 (ddd, *J* = 253.9, 14.3, 12.5 Hz), 154.6 (ddd, *J* = 249.2, 9.4, 2.7 Hz), 169.0. LRMS (ESI): *m/z* (%) 311 (50) $[M]^+$. Anal. Calcd for $C_{11}H_{10}BrF_3O_2$: C, 42.47; H, 3.24. Found: C, 42.39; H, 3.20.

1,1,1,3,3,3-Hexafluoropropan-2-yl 3-bromo-3-phenylpropanoate (4j)

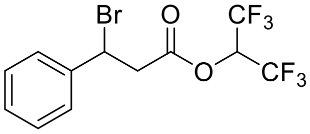

4j

Reaction was quenched with styrene after 90 min. The title compound was isolated as an inseparable mixture with 1,1,1,3,3,3-hexafluoropropan-2-yl cinnamate (8%) by flash column chromatography (silica gel; petroleum ether/CH_2Cl_2 90:10) as a pale

yellow oil (174 mg, 90%). ^{1}H NMR (400 MHz, $CDCl_3$): δ 3.44 (dd, *J* = 16.5, 6.3 Hz, 1H), 3.56 (dd, *J* = 16.5, 9.0 Hz, 1H), 5.38 (dd, *J* = 9.0, 6.3 Hz, 1H), 5.74 (hept, *J* = 6.0 Hz, 1H), 7.31–7.39 (m, 3H), 7.40–7.46 (m, 2H). ^{19}F NMR (376 MHz, $CDCl_3$): δ –73.2. ^{13}C NMR (101 MHz, $CDCl_3$): δ 43.9, 45.9, 66.7 (hept, *J* = 34.9 Hz), 120.2 (q, *J* = 281.9 Hz), 127.0, 129.0, 129.2, 139.7, 166.5.

***N*-Benzyl-3-bromo-3-phenylpropanamide (4k)**

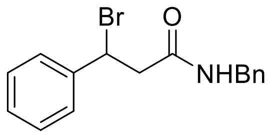

4k

Reaction was quenched with styrene after 30 min. The title compound was isolated by flash column chromatography (silica gel; CH_2Cl_2/EtOAc, 90:10) as a white solid (130 mg, 81% yield). Mp: 127-129 °C. IR (neat, cm^{-1}): *ν* 3263, 3089, 2965, 1635, 1567, 691, 582. ^{1}H NMR (400 MHz, $CDCl_3$): δ 3.07 (dd, *J* = 14.7, 5.9 Hz, 1H), 3.16 (dd, *J* = 14.7, 8.9 Hz, 1H), 4.43 (d, *J* = 5.7 Hz, 2H), 5.52 (dd, *J* = 8.9, 5.9 Hz, 1H), 5.81 (br s, 1H), 7.12–7.19 (m, 2H), 7.29–7.38 (m, 6H), 7.39–7.47 (m, 2H). ^{13}C NMR (101 MHz, $CDCl_3$): δ 43.7, 47.4, 49.4, 127.2, 127.6, 127.7, 128.7, 128.9, 129.0, 137.7, 141.0, 168.7. HRMS (APCI): $C_{16}H_{17}{}^{79}BrNO$ [M + H]$^+$ calculated: 318.0483, found: 318.0488. Anal. Calcd for $C_{16}H_{16}BrNO$: C, 60.39; H, 5.07; N, 4.40. Found: C, 60.41; H, 5.00; N, 4.38.

3-Bromo-3-phenyl-1-(pyrrolidin-1-yl)propan-1-one (4l)

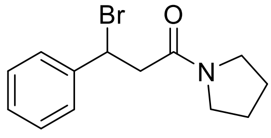

4l

Reaction was quenched with styrene after 30 min. The title compound was isolated by flash column chromatography (silica gel; CH_2Cl_2/EtOAc, 80:20) as a white solid (129 mg, 91% yield). Mp: 93-95 °C. IR (neat, cm^{-1}): *ν* 2989, 2870, 1626, 1440, 713, 605, 529. ^{1}H NMR (400 MHz, $CDCl_3$): δ 1.78–2.02 (m, 4H), 3.08 (dd, *J* = 15.7, 5.6 Hz, 1H), 3.36 (dd, *J* = 15.5, 8.8 Hz, 2H), 3.33–3.46 (m, 3H), 3.47–3.56 (m, 1H), 3.58 (dt, *J* = 9.9, 6.8 Hz, 1H), 5.58 (dd, *J* = 8.7, 5.6 Hz, 1H), 7.24–7.31 (m, 1H), 7.32–7.36 (m, 2H), 7.46 (d, *J* = 7.3 Hz, 2H). ^{13}C NMR (101 MHz, $CDCl_3$): δ 24.3, 26.0, 44.9, 45.8, 46.7, 49.7, 127.3, 128.4, 128.7, 141.6, 167.4. HRMS (APCI): $C_{13}H_{17}{}^{79}BrNO$ [M + H]$^+$ calculated: 282.0493, found: 282.0488.

***S*-Phenyl 3-bromo-3-phenylpropanethioate (4m)**

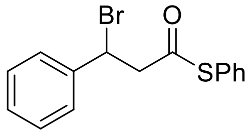

4m

Reaction was quenched with styrene after 40 min. The title compound was isolated by flash chromatography column (silica gel; petroleum ether/CH_2Cl_2 70:30) as a colourless oil (100 mg, 61% yield). IR (neat, cm^{-1}): *ν* 3189, 1856, 1667, 1485, 1423, 1148, 978, 699, 587. ^{1}H NMR

(400 MHz, $CDCl_3$): δ 3.49 (dd, *J* = 15.9, 6.4 Hz, 1H), 3.66 (dd, *J* = 15.9, 8.6 Hz, 1H), 5.44 (dd, *J* = 8.5, 6.4 Hz, 1H), 7.28–7.46 (m, 10H). ^{13}C (101 MHz, $CDCl_3$) δ 47.3, 52.6, 126.8, 127.3, 128.9, 128.9, 129.3, 129.7, 134.4, 140.3, 193.7. LRMS (ESI): *m/z* (%) 263 (15) $[M + Na - Br]^+$. Anal. Calcd for $C_{15}H_{13}BrOS$: C, 56.09; H, 4.08. Found: C, 56.01; H, 4.05.

3-Bromo-3-phenylpropanoic acid (4n)

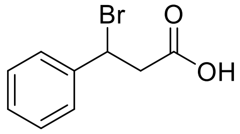

4n

Reaction was quenched with styrene after 1 h. The title compound was obtained by trituration with cold *n*-hexane (2 x 5 mL) as an off-white solid (101 mg, 88% yield). All analytical data are consistent with those reported in the literature.[14]

3-Bromo-3-(4-nitrophenyl)propanoic acid (4o)

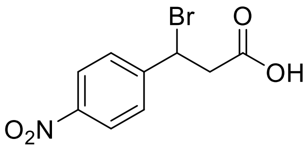

4o

The reaction was performed in dry DCE (3.0 mL) at 50 °C, using Br_2 (1.0 mL, 1.0 M in DCE, 1.0 mmol, 2.0 equiv) and stirred for 7 h. Then the solvent was removed *in vacuo*. The title compound was obtained by trituration with *n*-hexane (2 x 5 mL) as an off-white solid (110 mg, 64% yield). Compound has been previously described in the literature without NMR data. Mp: 167-169 °C; lit.[15]: 169-171 °C. ^1H NMR (400 MHz, CD_3CN): δ 3.27–3.43 (m, 2H), 5.50 (virt t, *J* = 7.4 Hz, 1H), 7.71 (d, *J* = 8.5 Hz, 2H), 8.20 (d, *J* = 8.4 Hz, 2H), 9.42 (s, 1H). ^{13}C NMR (101 MHz, CD_3CN): δ 43.8, 46.9, 124.8, 129.4, 148.6, 149.1, 170.9.

3-Bromo-1,3-diphenyl-1-propanone (4p)

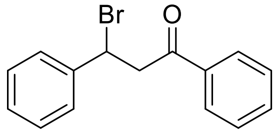

4p

Reaction was quenched with sat. aq. soln of $Na_2S_2O_3$ (ca. 4 mL) after 4 min and stirred for a further 5 min. The aqueous layer was extracted with CH_2Cl_2 (3 x 10 mL) and the combined organic phases were washed with H_2O, brine, dried over Na_2SO_4 and the solvent removed *in vacuo*. The title compound was obtained by recrystallization from Et_2O (ca. 6 mL) as a white solid (96 mg, 67% yield). All analytical data are consistent with those reported in the literature.[16]

3-Bromo-3-phenylpropanenitrile (4q)

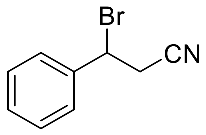

4q

The reaction was performed at double concentration (1.5 mL in dry CH_2Cl_2), using Br_2 (0.6 mL, 1.0 M in CH_2Cl_2, 0.6 mmol, 1.2 equiv), quenched with sat. aq. soln of $Na_2S_2O_3$ (ca. 4 mL) after 9 h and stirred for a further 5 min. The aqueous layer was extracted with CH_2Cl_2 (3 x 10 mL) and the combined organic phases were washed with H_2O, brine, dried over Na_2SO_4 and the solvent removed *in vacuo*. The residue was purified by flash column chromatography (silica gel; petroleum ether/EtOAc 95:5) to give the title compound as a pale yellow oil (60 mg, 57% yield). IR (neat, cm^{-1}): ν 3011, 2984, 2564, 1495, 1410, 1157, 942, 525. ^1H NMR (400 MHz, $CDCl_3$): δ 3.24 (dd, J = 16.7, 6.6, 1H), 3.30 (dd, J = 16.7, 7.0, 1H), 5.15 (virt t, J = 7.1, 1H), 7.32-7.49 (m, 5H). ^{13}C NMR (101 MHz, $CDCl_3$): δ 29.4, 45.3, 116.5, 127.0, 129.1, 129.5, 138.7. HRMS (APCI): $C_9H_9{}^{79}BrN$ $[M + H]^+$ calculated: 209.9912, found: 209.9913. Anal. Calcd for C_9H_8BrN: C, 51.46; H, 3.86; N, 6.67. Found: C, 51.46; H, 3.84; N, 6.67.

3-Bromo-3-(4-chlorophenyl)propanenitrile (4r)

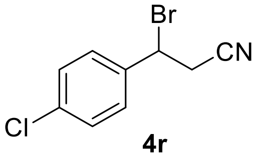

4r

The reaction was performed at double concentration (1.5 mL in dry CH_2Cl_2), using Br_2 (0.6 mL, 1.0 M in CH_2Cl_2, 0.6 mmol, 1.2 equiv), quenched with sat. aq. soln of $Na_2S_2O_3$ (ca. 4 mL) after 18 h and stirred for a further 5 min. The aqueous layer was extracted with CH_2Cl_2 (3 x 10 mL) and the combined organic phases were washed with H_2O, brine, dried over Na_2SO_4 and the solvent removed *in vacuo*. The residue was purified by flash column chromatography (silica gel; petroleum ether/EtOAc 90:10) to give the title compound as a brown oil (101 mg, 80% yield). IR (neat, cm^{-1}): ν 3001, 2974, 2442, 1589, 1321, 1010, 950, 501. ^1H NMR (400 MHz, $CDCl_3$): δ 3.22 (dd, J = 16.3, 6.5 Hz, 1H), 3.28 (dd, J = 16.3, 6.4 Hz, 1H), 5.11 (virt t, J = 7.1 Hz, 1H), 7.33–7.44 (m, 4H). ^{13}C NMR (101 MHz, $CDCl_3$): δ 29.4, 44.1, 116.2, 128.3, 129.4, 135.5, 137.2. LRMS (ESI): *m/z* (%) 279 (20) $[M + Cl]^+$. Anal. Calcd for C_9H_7BrClN: C, 44.21; H, 2.89; N 5.73. Found: C, 44.18; H, 2.91; N, 2.80.

(1-Bromo-3-chloropropyl)benzene (4s)

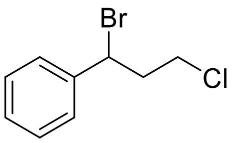

4s

Reaction was quenched with sat. aq. soln of $Na_2S_2O_3$ (ca. 4 mL) after 5 min and stirred for a further 5 min. The aqueous layer was extracted with CH_2Cl_2 (3 x 10 mL) and the combined organic phases were washed with H_2O, brine, dried over Na_2SO_4 and the solvent removed *in vacuo*. The title compound was isolated by flash column chromatography (silica gel; petroleum ether) as

a colourless oil (63 mg, 54% yield). All analytical data are consistent with those reported in the literature.[17]

3-Bromo-3-phenylpropan-1-ol (4t)

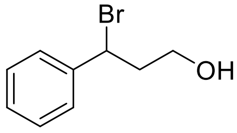

4t

Reaction was stirred for 5 min and then directly evaporated *in vacuo*. The residue was purified by flash column chromatography (silica gel; petroleum ether/EtOAc 80:20) to afford the title compound as a colourless oil (91 mg, 85% yield). All analytical data are consistent with those reported in the literature.[18] The title compound was stored at –20 °C as it was prone to degradation.

3-Bromo-3-(2,4,5-trifluorophenyl)propan-1-ol (4u)

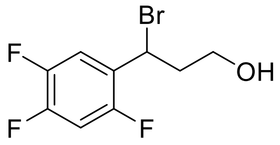

4u

Reaction was quenched with styrene after 90 min. The title compound was isolated by flash chromatography (silica gel; *n*-hexane/Et_2O 70:30) as a colourless oil (63 mg, 48% yield). The title compound was stored at –20 °C as it was prone to degradation. IR (neat, cm^{-1}): *ν* 3015, 2824, 1789, 1535, 1125, 1115, 984, 568. ^1H NMR (400 MHz, $CDCl_3$): δ 1.55 (br s, 1H), 2.22–2.32 (m, 1H), 2.44 (virt ddt, *J* = 14.5, 9.7, 4.9 Hz, 1H), 3.79 (virt dt, *J* = 10.8, 5.3 Hz, 1H), 3.87 (ddd, *J* = 11.0, 8.0, 4.6 Hz, 1H), 5.43 (dd, *J* = 9.5, 5.4 Hz, 1H), 6.93 (virt dt, *J* = 9.7, 6.5 Hz, 1H), 7.31 (ddd, *J* = 10.5, 8.5, 6.8 Hz, 1H). ^{19}F NMR (376 MHz, $CDCl_3$): δ –117.9 (dd, *J* = 15.1, 4.6 Hz), –132.5 (dd, *J* = 21.6, 4.6 Hz), –141.1 (dd, *J* = 21.5, 15.1 Hz). ^{13}C NMR (101 MHz, $CDCl_3$): δ 40.9, 41.7 (d, *J* = 2.9 Hz), 60.1, 105.9 (dd, *J* = 28.2, 20.9 Hz), 116.8 (ddd, *J* = 20.1, 4.5, 1.4 Hz), 125.6 (ddd, *J* = 15.4, 5.1, 4.5 Hz), 147.0 (ddd, *J* = 245.9, 12.8, 3.6 Hz), 149.9 (ddd, *J* = 253.4, 14.5, 12.5 Hz), 154.5 (ddd, *J* = 248.3, 9.4, 2.7 Hz). LRMS (ESI): *m/z* (%) 535 (61) $[2 M]^+$. Anal. Calcd for $C_9H_8BrF_3O$: C, 40.18; H, 3.00. Found: C, 40.14; H, 2.97.

3-Bromo-3-(2,4,5-trifluorophenyl)propyl methanesulphonate (4v)

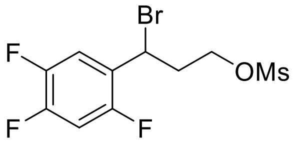

4v

Reaction was quenched with sat. aq. soln of $Na_2S_2O_3$ (ca. 4 mL) after 1 h and stirred for a further 5 min. The aqueous layer was extracted with CH_2Cl_2 (3 x 10 mL) and the combined organic phases were washed with H_2O, brine, dried over Na_2SO_4 and the solvent removed *in vacuo*. The title compound was isolated by flash column chromatography (silica gel; petroleum ether/EtOAc 80:20) as a pale yellow oil (169 mg, 97% yield). IR (neat, cm^{-1}): *ν* 2921, 1513, 1353, 1134, 1170, 992, 575. ^1H NMR (400 MHz, $CDCl_3$): δ 2.44–2.55 (m, 1H),

2.61 (virt ddt, *J* = 14.6, 9.6, 4.7 Hz, 1H), 3.06 (s, 3H), 4.32–4.49 (m, 2H), 5.31 (dd, 8.9, 5.0 Hz, 1H), 6.97 (virt dt, *J* = 9.7, 6.5 Hz, 1H), 7.30 (ddd, *J* = 10.3, 8.4, 6.9 Hz, 1H). ^{19}F NMR (376 MHz, $CDCl_3$): δ –117.4 (dd, *J* = 15.1, 4.8 Hz), –131.4 (dd, *J* = 21.5, 4.9 Hz), –140.5 (dd, *J* = 21.6, 15.1 Hz). ^{13}C NMR (101 MHz, $CDCl_3$): δ 37.4, 37.9, 40.3 (d, *J* = 2.8 Hz), 66.9, 106.2 (dd, *J* = 28.1, 21.0 Hz), 116.7 (ddd, *J* = 20.2, 4.3, 1.4 Hz), 124.5 (ddd, *J* = 15.1, 4.8, 4.4 Hz), 147.0 (ddd, *J* = 246.6, 12.8, 3.6 Hz), 150.2 (ddd, *J* = 254.3, 14.4, 12.5 Hz), 154.6 (ddd, *J* = 248.9, 9.5, 2.7 Hz). HMRS (APCI): $C_{10}H_{10}BrF_3O_3S$ $[M + H]^+$ calculated: 345.9502, found: 345.9492.

1-Bromoadamantane (4y)

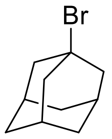

4y

Reaction was quenched with sat. aq. soln of $Na_2S_2O_3$ (ca. 4 mL) after 1 h and stirred for further 5 min. The aqueous layer was extracted with CH_2Cl_2 (3 x 10 mL) and the combined organic phases were washed with H_2O, brine, dried over Na_2SO_4 and the solvent removed *in vacuo*. The title compound was obtained by flash column chromatography (silica gel; petroleum ether) as a white solid (76 mg, 68% yield). All analytical data are consistent with those reported in the literature.[19]

(3-Bromo-3-methylbutyl)benzene (4za)

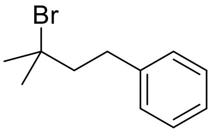

4za

Reaction was stirred for 30 min and then directly evaporated *in vacuo*. The ^{1}H NMR yield (51%) was determined by adding dibromomethane (35 μL, 87 mg, 0.5 mmol, 1.0 equiv) as internal standard to the crude product re-dissolved in $CDCl_3$. All analytical data are consistent with those reported in the literature.[20]

3-Bromo-3-methylbutyl 4-methylbenzenesulfonate (4zb)

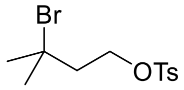

4zb

Reaction was quenched with styrene after 24 h. The title compound was isolated as an inseparable 3:1 mixture with 3,4-dibromo-3-methylbutyl 4-methylbenzenesulfonate by flash column chromatography (silica gel; petroleum ether 100%) as a colourless oil (48 mg, 30% yield). All analytical data are consistent with those reported in the literature.[21]

6.2 Bromination followed by solvolysis with 2,2,2-trifluoroethanol

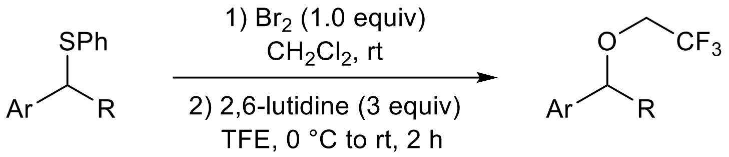

3-(Thiophen-2-yl)-3-(2,2,2-trifluoroethoxy)propyl methanesulphonate (4wa)

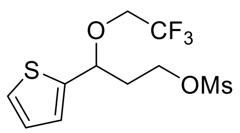

4wa

To a stirred solution of the sulphide (0.5 mmol) in dry CH_2Cl_2 (3.0 mL) was added Br_2 (0.5 mL, 1.0 M solution in CH_2Cl_2, 0.5 mmol, 1.0 equiv) under N_2 atmosphere. The reaction was stirred at rt for 25 min and then quenched by the addition of sat. aq. soln of $Na_2S_2O_3$ (ca. 4 mL) and stirred for a further 5 min. The aqueous layer was extracted with CH_2Cl_2 (3 x 10 mL) and the combined organic phases were washed with H_2O, brine, dried over Na_2SO_4 and the solvent removed *in vacuo.* The residue was re-dissolved in TFE (5.0 mL) and 2,6-lutidine (175 μL, 161 mg, 3.0 equiv) was added at 0 °C. The mixture was stirred for 2 h, then 1.0 M HCl (5 mL) and petroleum ether (20 mL) were added. The aqueous layer was extracted with petroleum ether (2 x 20 mL) and the combined organic layers were washed with 1.0 M HCl (2 x 5 mL), brine, dried over Na_2SO_4 and concentrated *in vacuo*. The residue was purified by flash column chromatography (silica gel; petroleum ether/CH_2Cl_2, 70:30 → 0:100) to afford the title compound as a yellow oil (80 mg, 50% yield). IR (neat, cm^{-1}): *ν* 2938, 1353, 1274, 1158, 964, 526. ^1H NMR (400 MHz, $CDCl_3$): δ 2.22 (ddd, *J* = 19.6, 9.3, 5.0 Hz, 1H), 2.36 (ddd, *J* = 19.1, 9.4, 4.8 Hz, 1H), 3.02 (s, 3H), 3.64–3.82 (m, 2H), 4.29 (virt dt, *J* = 10.2, 5.2 Hz, 1H), 4.45 (ddd, *J* = 9.8, 8.8, 4.5 Hz, 1H), 4.91 (dd, *J* = 9.1, 4.6 Hz, 1H), 7.01 (dd, *J* = 5.0, 3.5 Hz, 1H), 7.06 (d, *J* = 2.8 Hz, 1H), 7.36 (d, *J* = 4.7 Hz, 1H). ^{19}F NMR (376 MHz, $CDCl_3$): δ –77.2. ^{13}C NMR (101 MHz, $CDCl_3$): δ 37.1, 37.8, 65.4 (q, *J* = 34.3 Hz), 66.3, 74.6, 122.5, 125.3, 126.5, 126.9, 142.3. HMRS (EI): $C_{10}H_{13}F_3O_4S_2Na$ $[M + Na]^+$ calculated: 341.0105, found: 341.0089.

1-Bromo-4-[phenyl(2,2,2-trifluoroethoxy)methyl]benzene (4xa)

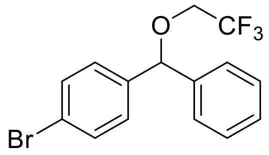

4xa

The procedure was similar to **4wa**. The reaction was stirred at rt for 1 h and concentrated *in vacuo* without any quench before solvolysis step. The title compound was obtained by flash column chromatography (silica gel; petroleum ether/EtOAc 98:2) as a pale yellow oil (134 mg, 78% yield). All analytical data are consistent with those reported in the literature.[4]

6.3 Bromination followed by mesylation with sulphide (1u)

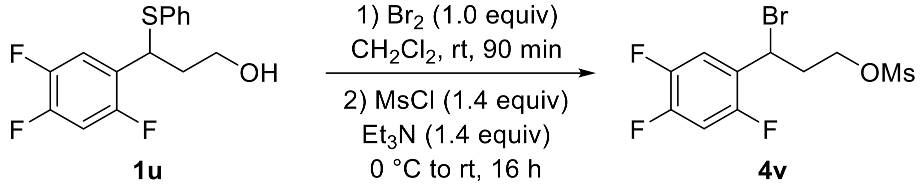

To a stirred solution of sulphide **1u** (144 mg, 0.5 mmol) in dry CH_2Cl_2 (3.0 mL) was added Br_2 (500 µL, 1.0 M in CH_2Cl_2, 0.5 mmol, 1.0 equiv) at rt under N_2 atmosphere and stirred for 90 min. The reaction mixture was quenched by the addition of styrene (68 µL, 62 mg, 0.6 mmol, 1.2 equiv). To the resulting colourless solution were added dropwise methanesulphonyl chloride (54 µL, 79 mg, 0.7 mmol, 1.4 equiv) and triethylamine (97 µL, 70 mg, 0.7 mmol, 1.4 equiv) at 0 °C. The reaction mixture was allowed to reach rt and stirred for 16 h, then diluted with H_2O (10 mL) and extracted with CH_2Cl_2 (3 x 10 mL). The combined organic layers were washed with H_2O, brine and dried over Na_2SO_4. The solvent was removed *in vacuo* and the crude mixture was purified by flash column chromatography (silica gel; petroleum ether/EtOAc, 90:10) to afford bromide **4v** as a brown oil (65 mg, 45% yield).

6.4 Large scale bromination of β-sulphido ester (1f)

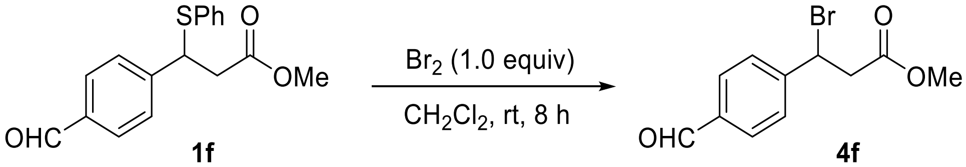

To a stirred solution of sulphide **1f** (1.50 g, 5.0 mmol) in dry CH_2Cl_2 (15.0 mL) was added *via* syringe Br_2 (5.0 mL, 1.0 M in CH_2Cl_2, 5.0 mmol, 1.0 equiv) under N_2 atmosphere. The reaction was stirred at rt for 8 h, then quenched by the addition of styrene (743 µL, 676 mg, 6.5 mmol, 1.3 equiv). The mixture was concentrated *in vacuo* and the residue was purified by flash column chromatography (silica gel; CH_2Cl_2/petroleum ether 90:10) to afford the corresponding bromide (1.29 g, 95% yield).

7. General procedure for the transesterification of enantiomerically enriched hexafluoroisopropyl esters to cinnamate methyl esters (GP4)

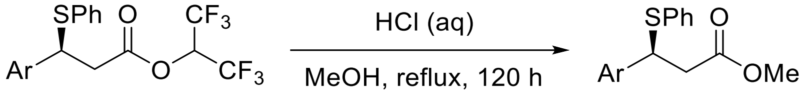

To a solution of (*S*)-1,1,1,3,3,3-hexafluoropropan-2-yl 3-aryl-3-(phenylthio)propanoate (1.0 mmol) in methanol (7.0 mL) was added a few drops of concentrated HCl and the resulting mixture was stirred under reflux for 120 h. After the completion of the reaction, as judged by TLC analysis, the solvent was removed *in vacuo* and the residue was purified by flash column chromatography on silica gel to give the corresponding (*S*)-methyl 3-aryl-3-(phenylthio)propanoate.

(–)-(*S*)-Methyl-3-phenyl-3-(phenylthio)propanoate (–)-(*S*)-1a

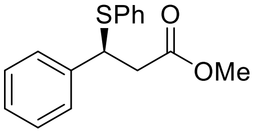

(–)-(*S*)-**1a**

Prepared according to GP4. The title compound was isolated by flash column chromatography (silica gel; petroleum ether/EtOAc, 95:5) as a colourless oil (253 mg, 93% yield). Enantiomeric excess was determined by HPLC analysis (CHIRALPAK IB-H, *n*-hexane/*i*-PrOH, 99:1, 1.0 mL/min, 210 nm): t_r (minor) = 8.6 min; t_r (major) = 11.3 min: 97% ee. $[\alpha]_D^{20}$ = –150.2 (*c* 1.27, $CHCl_3$). Enantioenriched (–)-(S)-**1a** has previously been described.[2]

(–)-(*S*)-Methyl-3-(phenylthio)-3-(*o*-tolyl)propanoate (–)-(*S*)-1b

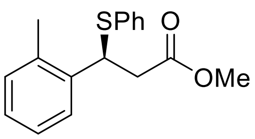

(–)-(*S*)-**1b**

Prepared according to GP4. The title compound was isolated by flash column chromatography (silica gel; petroleum ether/EtOAc, 95:5) as a colourless oil (255 mg, 89% yield). Enantiomeric excess was determined by HPLC analysis (CHIRALPAK OD-H, *n*-hexane/*i*-PrOH, 95:5, 1.0 mL/min, 210 nm): t_r (minor) = 6.0 min; t_r (major) = 10.4 min: 78% ee. $[\alpha]_D^{20}$ = –62.3 (*c* 1.41, $CHCl_3$).

(–)-(*S*)-Methyl-3-(4-chlorophenyl)-3-(phenylthio)propanoate (–)-(*S*)-1d

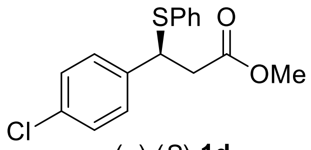

(–)-(*S*)-**1d**

Prepared according to GP4. The title compound was isolated by flash column chromatography (silica gel; petroleum ether/EtOAc, 95:5) as a yellow oil (288 mg, 94% yield). Enantiomeric excess was determined by HPLC analysis (CHIRALPAK OD-H, *n*-hexane/*i*-PrOH, 95:5, 0.5 mL/min, 210 nm): t_r (major) = 10.9 min; t_r (minor) = 14.0 min: 99% ee. $[\alpha]_D^{20}$ = –169.0 (*c* 1.18, $CHCl_3$).

(–)-(*S*)-Methyl-3-(phenylthio)-3-(4-(trifluoromethyl)phenyl)propanoate (–)-(*S*)-1e

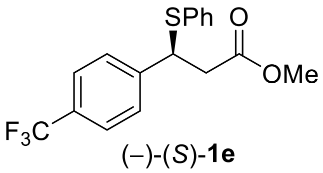

(–)-(*S*)-**1e**

Prepared according to GP4. The title compound was isolated by flash column chromatography (silica gel; petroleum ether/EtOAc, 95:5) as a white solid (316 mg, 93% yield). Mp: 91–93 °C. Enantiomeric excess was determined by HPLC analysis (CHIRALPAK OD-H, *n*-hexane/*i*-PrOH, 95:5, 0.5 mL/min, 210 nm): t_r (major) = 10.2 min; t_r (minor) = 13.0 min: 98% ee. $[\alpha]_D^{20}$ = –139.0 (*c* 1.51, $CHCl_3$).

8. Optically active bromides

8.1. Bromination of enantiomerically enriched sulphides

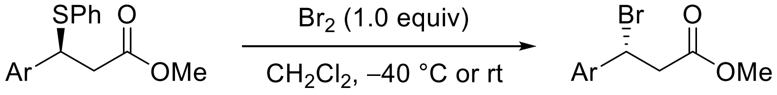

(*S*)-**1** (*R*)-**4** }

(+)-(*R*)-Methyl 3-bromo-3-phenylpropanoate (+)-(*R*)-4a

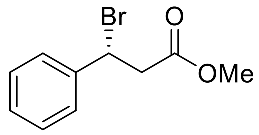

(+)-(*R*)-**4a**

To an oven-dried 10 mL Schlenk tube containing a stirred solution of sulphide (–)-(*S*)-**1a** (68 mg, 0.25 mmol, 97% *ee*) in dry CH_2Cl_2 (1.5 mL) cooled to –40 °C was added *via* syringe Br_2 (250 µL, 1M in CH_2Cl_2, 0.25 mmol, 1.0 equiv) under N_2 atmosphere. The reaction mixture was quenched *via* syringe with styrene (34 µL, 31 mg, 0.3 mmol, 1.3 equiv) after 15 h, which led to an immediate fading of the red colour. Evaporation of the solvent *in vacuo* and purification by flash column chromatography (silica gel; petroleum ether/EtOAc, 95:5) gave the title compound as a yellow oil (54 mg, 89% yield). Enantiomeric excess was determined by HPLC

analysis (CHIRALPAK IB-H, *n*-hexane/*i*-PrOH, 95:5, 0.75 mL/min, 210 nm): t_r (major) = 7.3 min; t_r (minor) = 9.3 min: 93% ee. $[\alpha]_D^{20}$ = +114.9 (*c* 1.01, $CHCl_3$).

(–)-(*R*)-Methyl 3-bromo-3-(o-tolyl)propanoate (–)-(*R*)-4b

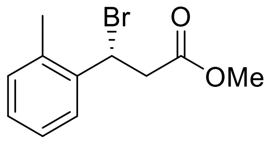

(-)-(*R*)-**4b**

To an oven-dried 10 mL Schlenk tube containing a stirred solution of sulphide (–)-(*S*)-**1b** (72 mg, 0.25 mmol, 78% *ee*) in dry CH_2Cl_2 (1.5 mL) cooled to –40 °C was added *via* syringe Br_2 (250 μL, 1M in CH_2Cl_2, 0.25 mmol, 1 equiv) under N_2 atmosphere. The reaction mixture was quenched *via* syringe with styrene (34 μL, 31 mg, 0.3 mmol, 1.3 equiv) after 15 h, which led to an immediate fading of the red colour. Evaporation of the solvent *in vacuo* and purification by flash column chromatography (silica gel; petroleum ether/EtOAc, 97:3) gave the title compound as a yellow oil (64 mg, 99% yield). Enantiomeric excess was determined by HPLC analysis (CHIRALPAK IB-H, *n*-hexane/*i*-PrOH, 95:5, 0.75 mL/min, 210 nm): t_r (major) = 6.9 min; t_r (minor) = 10.5 min: 66% ee. $[\alpha]_D^{20}$ = –13.6 (*c* 1.03, $CHCl_3$).

(+)-(*R*)-Methyl 3-bromo-3-(4-chlorophenyl)propanoate (+)-(*R*)-4d

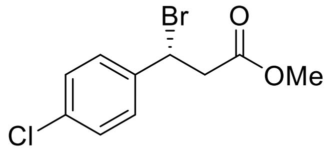

(+)-(*R*)-**4d**

To an oven-dried 10 mL Schlenk tube containing a stirred solution of sulphide (–)-(*S*)-**1d** (77 mg, 0.25 mmol, 99% *ee*) in dry CH_2Cl_2 (1.5 mL) cooled to –40 °C was added *via* syringe Br_2 (250 μL, 1M in CH_2Cl_2, 0.25 mmol, 1.0 equiv) under N_2 atmosphere. The reaction mixture was quenched *via* syringe with styrene (34 μL, 31 mg, 0.3 mmol, 1.3 equiv) after 24 h, which led to an immediate fading of the red colour. Evaporation of the solvent *in vacuo* and purification by flash column chromatography (silica gel; petroleum ether/EtOAc, 95:5) gave the title compound as a yellow oil (67 mg, 97% yield). Enantiomeric excess was determined by HPLC analysis (CHIRALPAK IB-H, *n*-hexane/*i*-PrOH, 98:2, 0.5 mL/min, 210 nm): t_r (major) = 14.8 min; t_r (minor) = 15.6 min: 93% ee. $[\alpha]_D^{20}$ = +85.2 (*c* 1.09, $CHCl_3$).

(+)-(*R*)-Methyl 3-bromo-3-(4-(trifluoromethyl)phenyl)propanoate (+)-(*R*)-4e

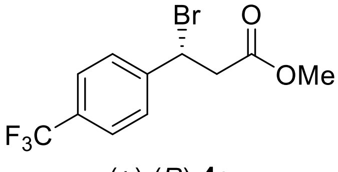

(+)-(*R*)-**4e**

To an oven-dried 10 mL Schlenk tube containing a stirred solution of sulphide (–)-(*S*)-**1e** (85 mg, 0.25 mmol, 98% *ee*) in dry CH_2Cl_2 (750 μL) was added *via* syringe Br_2 (275 μL, 1M in CH_2Cl_2, 0.275 mmol, 1.1 equiv) at rt under N_2 atmosphere. The reaction mixture was quenched *via* syringe with styrene (34 μL, 31 mg, 0.3 mmol, 1.3 equiv) after 48 h at rt, which led to an immediate fading of the red colour. Evaporation of the solvent *in vacuo* and

purification by flash column chromatography (silica gel; petroleum ether/CH_2Cl_2, 70:30) gave the title compound as a yellow oil (74 mg, 95% yield). Enantiomeric excess was determined by HPLC analysis (CHIRALPAK IB-H, *n*-hexane/*i*-PrOH, 98:2, 0.5 mL/min, 210 nm): t_r (major) = 14.3 min; t_r (minor) = 15.3 min: 86% ee. $[\alpha]_D^{20}$ = +73.9 (*c* 1.68, $CHCl_3$).

8.2. Monitoring the racemisation of bromide (*R*)-4a

Enantiomeric excess of β-bromo ester (*R*)-**4a** (93% ee), stored at **–20** °C or rt, was measured over a 14-week period.

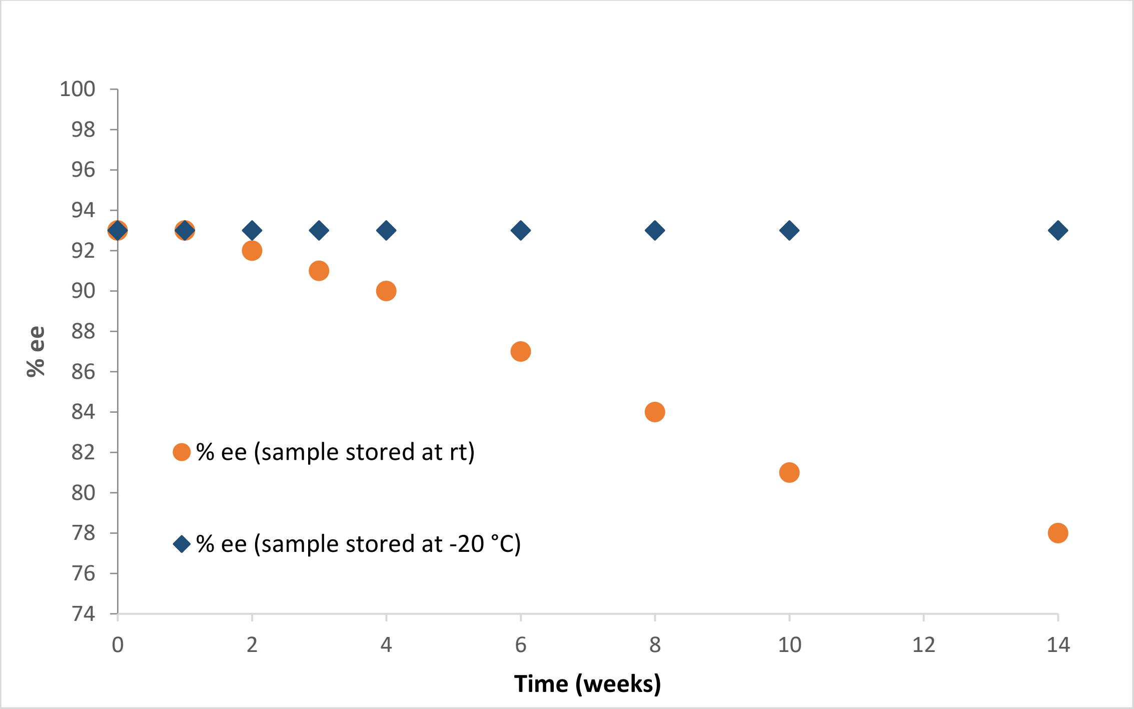

9. Synthesis of (–)-(*S*)-3-azido-3-phenylpropan-1-ol

9.1 Two-pot procedure

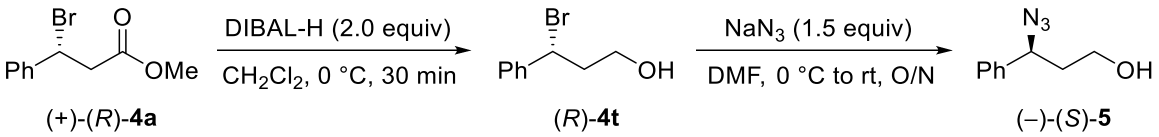

To a stirred solution of (+)-(*R*)-**4a** (61 mg, 0.25 mmol) in dry CH_2Cl_2 (2.0 mL) was added *via* syringe DIBAL-H (0.4 mL, 1.0 M in CH_2Cl_2, 0.4 mmol, 2.0 equiv) at 0 °C under N_2 atmosphere. After stirring for 30 min (TLC analysis), the mixture was carefully quenched with H_2O, filtered over a Celite pad, which was washed with CH_2Cl_2 (3 x 10 mL). The combined

organic layers were washed with H_2O, brine, dried over Na_2SO_4 and concentrated *in vacuo* to give the corresponding alcohol (*R*)-**4t** as a brown oil (50 mg, 93% yield). Enantiomeric excess was determined by HPLC analysis (CHIRALPAK IB-H, *n*-hexane/*i*-PrOH, 90:10, 0.75 mL/min, 210 nm): t_r (major) = 9.3 min; t_r (minor) = 10.3 min: 83% *ee*. All analytical data are consistent with those reported in the literature.[22]

Alcohol (*R*)-**4t** was dissolved in DMF (1.0 mL) and sodium azide (23 mg, 0.35 mmol, 1.5 equiv) was added at 0 °C under N_2 atmosphere. The reaction was allowed to reach rt and stirred overnight. H_2O (3 mL) was added and the aqueous layer was extracted with Et_2O (3 x 10 mL) and the combined organic layers were washed with H_2O, brine, dried over Na_2SO_4 and concentrated *in vacuo*. The residue was purified by flash column chromatography (silica gel; petroleum ether/EtOAc, 80:20) to afford the azido alcohol (–)-(*S*)-**5** as a colourless oil (35 mg, 86% yield). Enantiomeric excess was determined by HPLC analysis (CHIRALPAK IB-H, *n*-hexane/*i*-PrOH, 90:10, 0.75 mL/min, 210 nm): t_r (minor) = 8.6 min; t_r (major) = 9.4 min: 83% ee. The enantiomerically enriched (+)-(*R*)-**5** enantiomer has been previously described.[23]

9.2 One-pot procedure

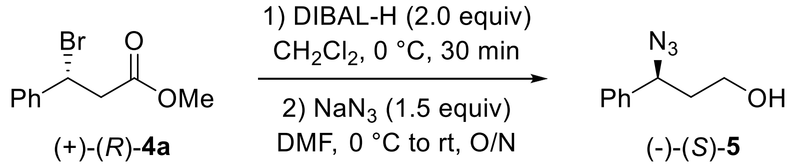

To a stirred solution of (+)-(*R*)-**4a** (61 mg, 0.25 mmol) in dry CH_2Cl_2 (2.0 mL) was added *via* syringe DIBAL-H (0.4 mL, 1.0 M in CH_2Cl_2, 0.4 mmol, 2.0 equiv) at 0 °C under N_2 atmosphere. After stirring for 30 min (TLC analysis), dry DMF (2.0 mL) was added, followed by sodium azide (25 mg, 0.38 mmol, 1.5 equiv) and the reaction was allowed to reach rt and stirred overnight. The mixture was carefully quenched with H_2O and filtered over a Celite pad, which was washed with CH_2Cl_2 (3 x 10 mL). The combined organic layers were washed with H_2O, brine, dried over Na_2SO_4 and concentrated *in vacuo*. The residue was purified by flash column chromatography (silica gel; petroleum ether/EtOAc, 80:20) to afford the title compound as a colourless oil (27 mg, 62% yield). Enantiomeric excess was determined by HPLC analysis (CHIRALPAK IB-H, *n*-hexane/*i*-PrOH, 90:10, 0.75 mL/min, 210 nm): t_r (minor) = 8.6 min; t_r (major) = 9.4 min: 90% ee. $[\alpha]_D^{20}$ = –186.9 (*c* 1.0, $CHCl_3$); lit.[23]: (+)-(*R*)-**5**: $[\alpha]_D^{20}$ = +192.7 (*c* 0.95, $CHCl_3$). No literature ee value was disclosed, however, an ee of >90% is deduced based on the synthetic sequence.

10. NMR studies

10.1. NMR monitoring of the bromination of (1e)

10.1.1. Low-temperature NMR spectroscopic characterization of adduct (1e·Br_2)

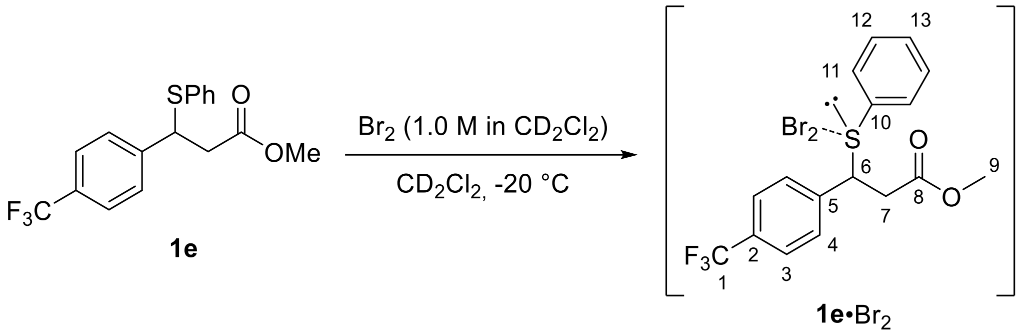

A solution of sulphide **1e** (34 mg, 0.1 mmol) in CD_2Cl_2 (0.6 mL), containing 1,1,2,2-tetrachloroethane (11 µL,17 mg, 0.1 mmol, 1.0 equiv) as internal standard, was prepared under N_2 atmosphere. The solution was transferred *via* syringe to a 5 mm NMR tube placed in a suba-seal capped Schlenk tube. The NMR tube was sealed with a precision seal rubber septum, which was covered with parafilm. The NMR tube was allowed to equilibrate to –20 °C in the NMR spectrometer, before a Br_2 solution (100 µL, 1.0 M in CD_2Cl_2, 0.1 mmol, 1.0 equiv), precooled to ca –20 °C in a freezer, was added *via* syringe. The NMR tube was placed immediately back into the NMR spectrometer and a series of 1D and 2D NMR experiments were recorded.

^{1}H NMR (800 MHz, CD_2Cl_2, –20 °C): δ 3.05 (dd, *J* = 16.5, 10.1 Hz, 1H, H7), 3.14 (dd, *J* = 16.6, 4.8 Hz, 1H, H7), 3.55 (s, 3H, H9), 4.82 (dd, *J* = 9.8, 5.0 Hz, 1H, H6), 7.33-7.35 (m, 4H, H4, H12), 7.40 (t, *J* = 7.2 Hz, 1H, H13), 7.43 (d, *J* = 7.5 Hz, 2H, H11), 7.53 (d, *J* = 7.8 Hz, 2H, H3).

^{19}F NMR (376 MHz, CD_2Cl_2, 20 °C): δ –62.9.

^{13}C NMR (201 MHz, CD_2Cl_2, –20 °C): δ 38.4 (C7), 52.4 (C9), 52.9 (br s, C6), 124.0 (q, *J* = 272.3 Hz, C1), 125.7 (q, *J* = 3.5 Hz, C3), 128.7 (C4), 129.0 (br s, C10), 129.8 (C12), 130.1 (q, *J* = 32.4 Hz, C2), 130.6 (C13), 133.1 (C11), 141.2 (br s, C5), 169.9 (C8).

^{1}H NMR (800 MHz, CD_2Cl_2, –20 °C) of **1e** *vs* **1e**•Br_2 (2 min after Br_2 addition)

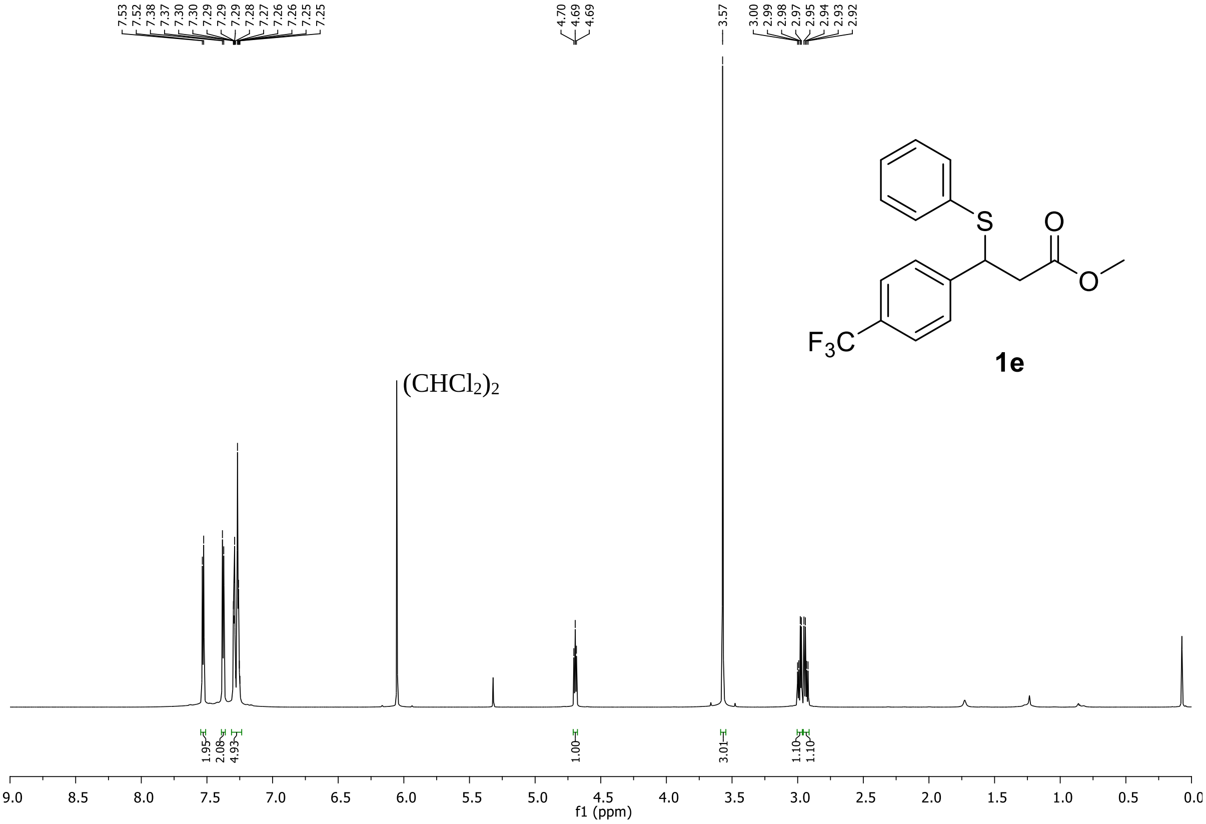

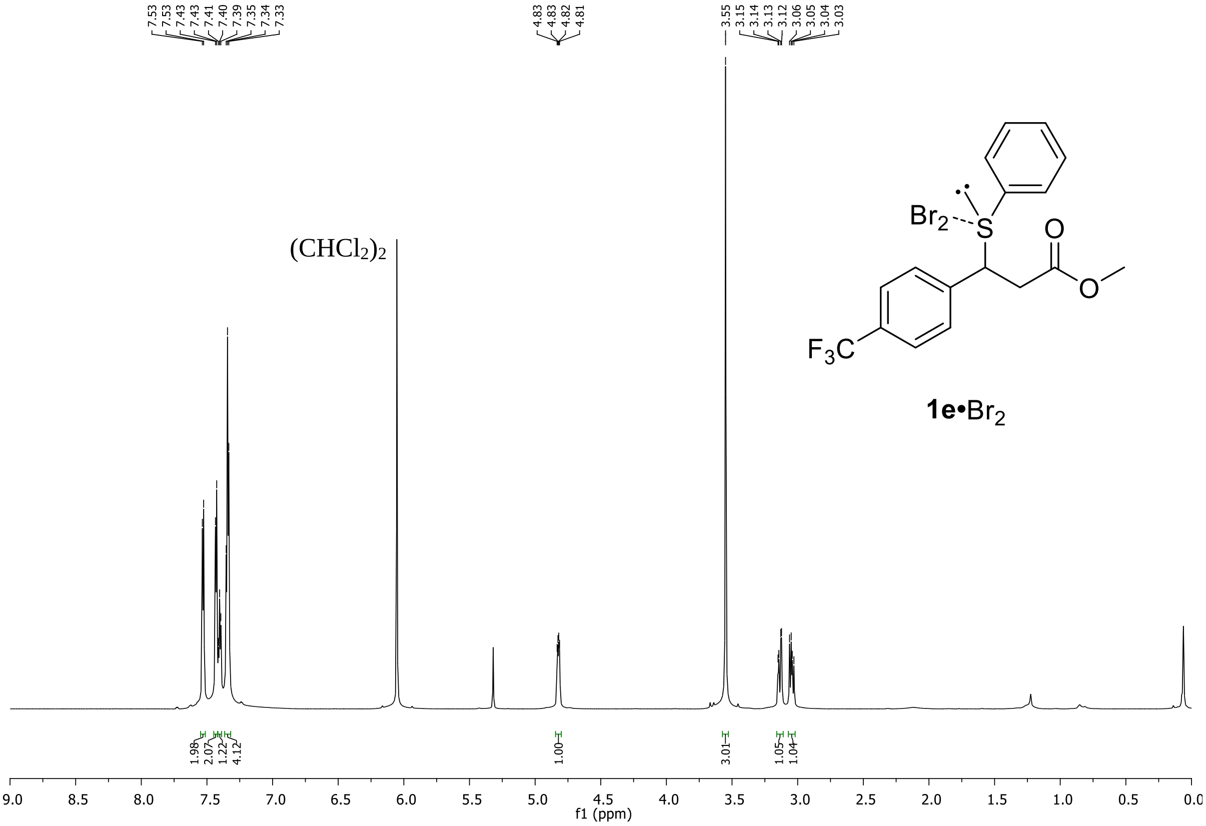

^{13}C NMR (201 MHz, CD_2Cl_2, –20 °C) of **1e** *vs* **1e**·Br_2 (15 min after Br_2 addition)

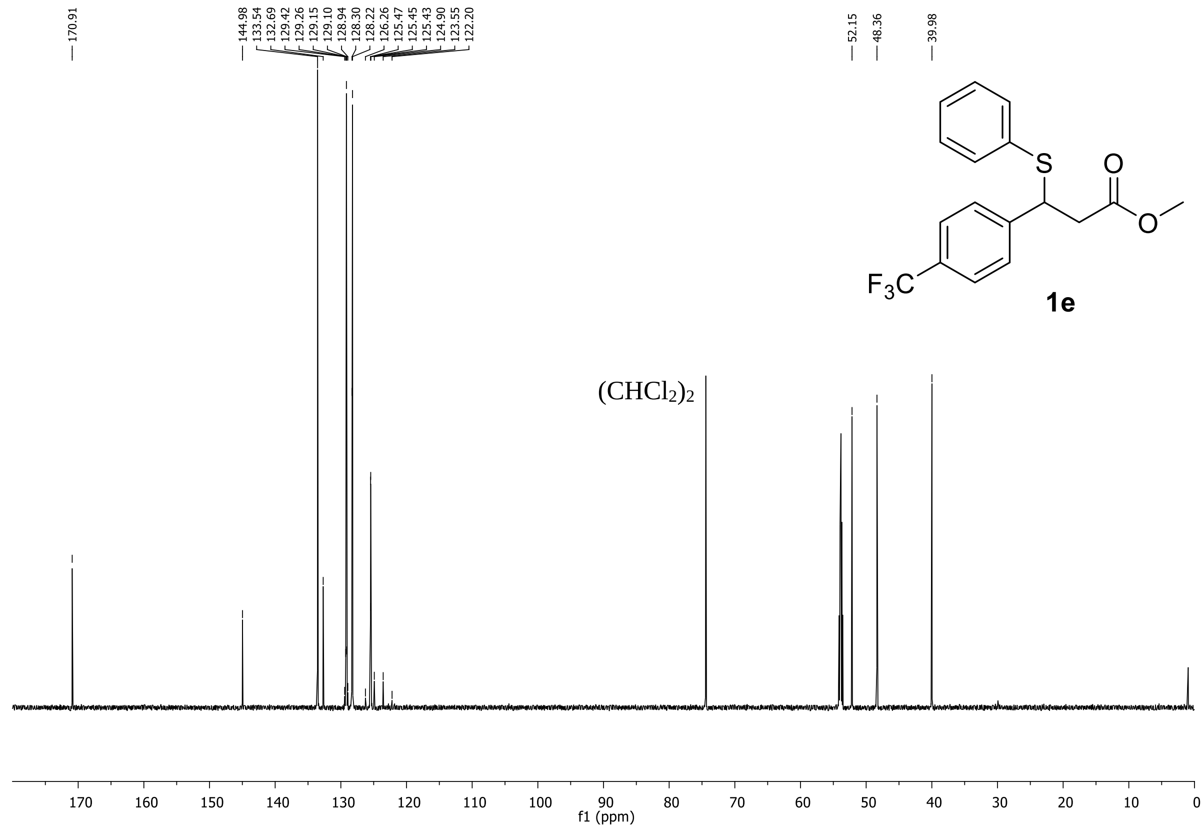

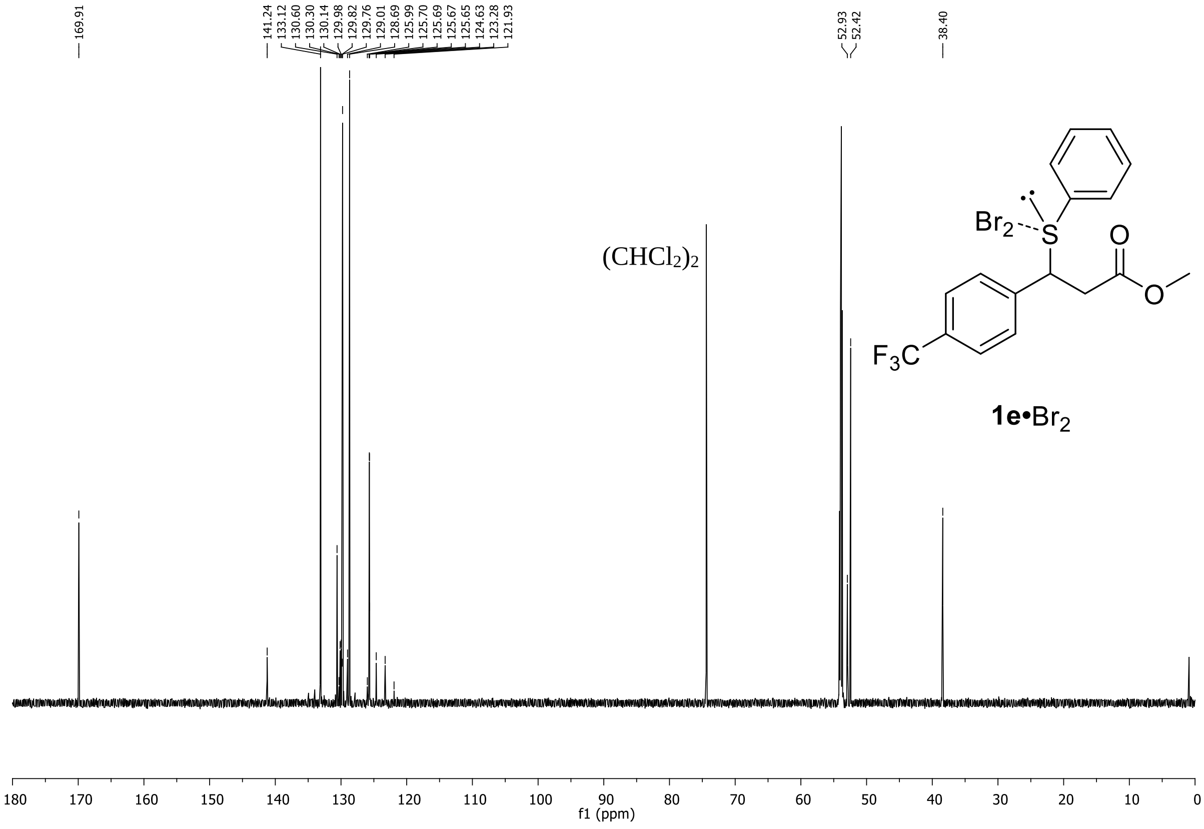

HSQC (800 MHz, CD_2Cl_2, –20 °C)

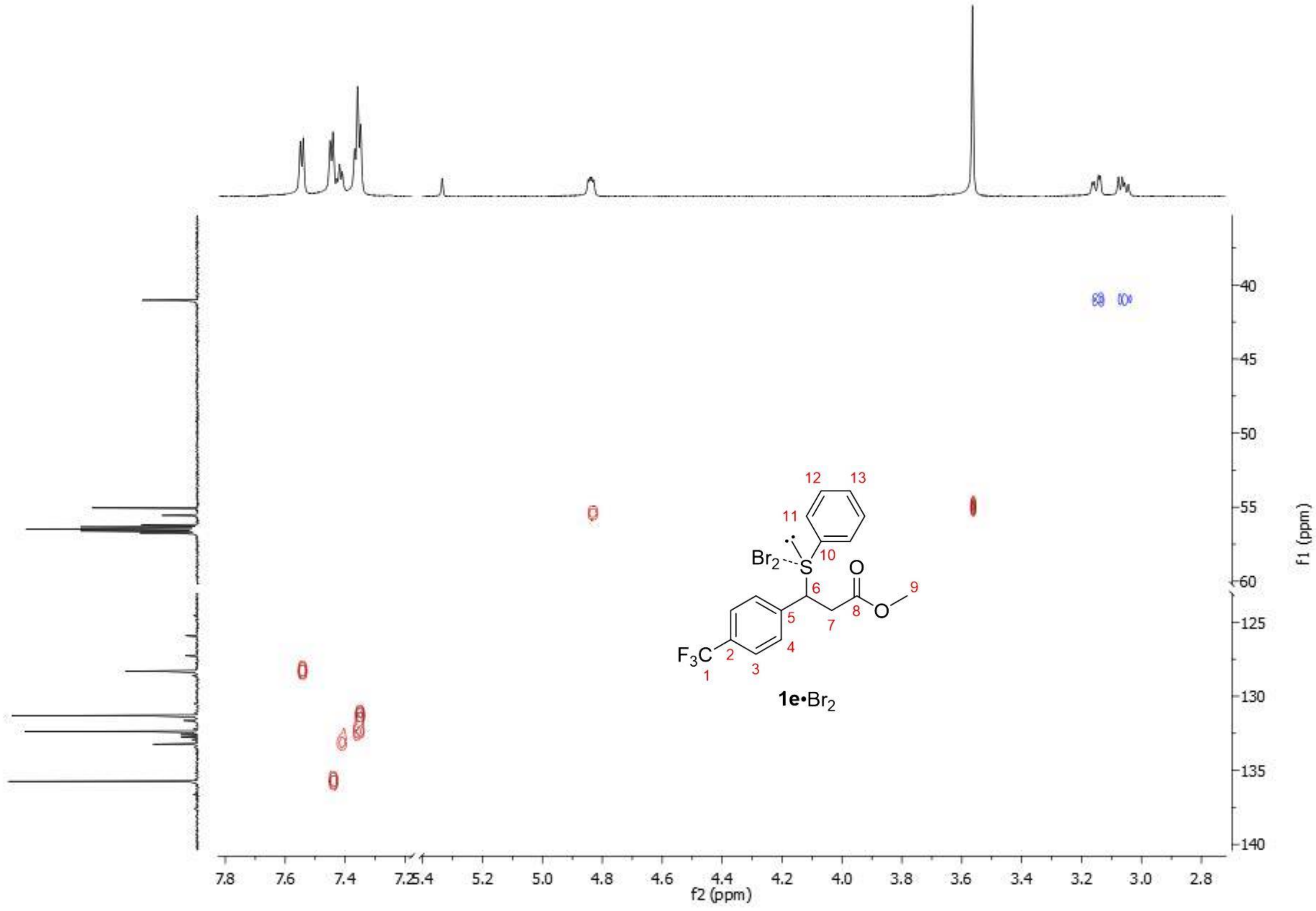

HMBC (800 MHz, CD_2Cl_2, –20 °C)

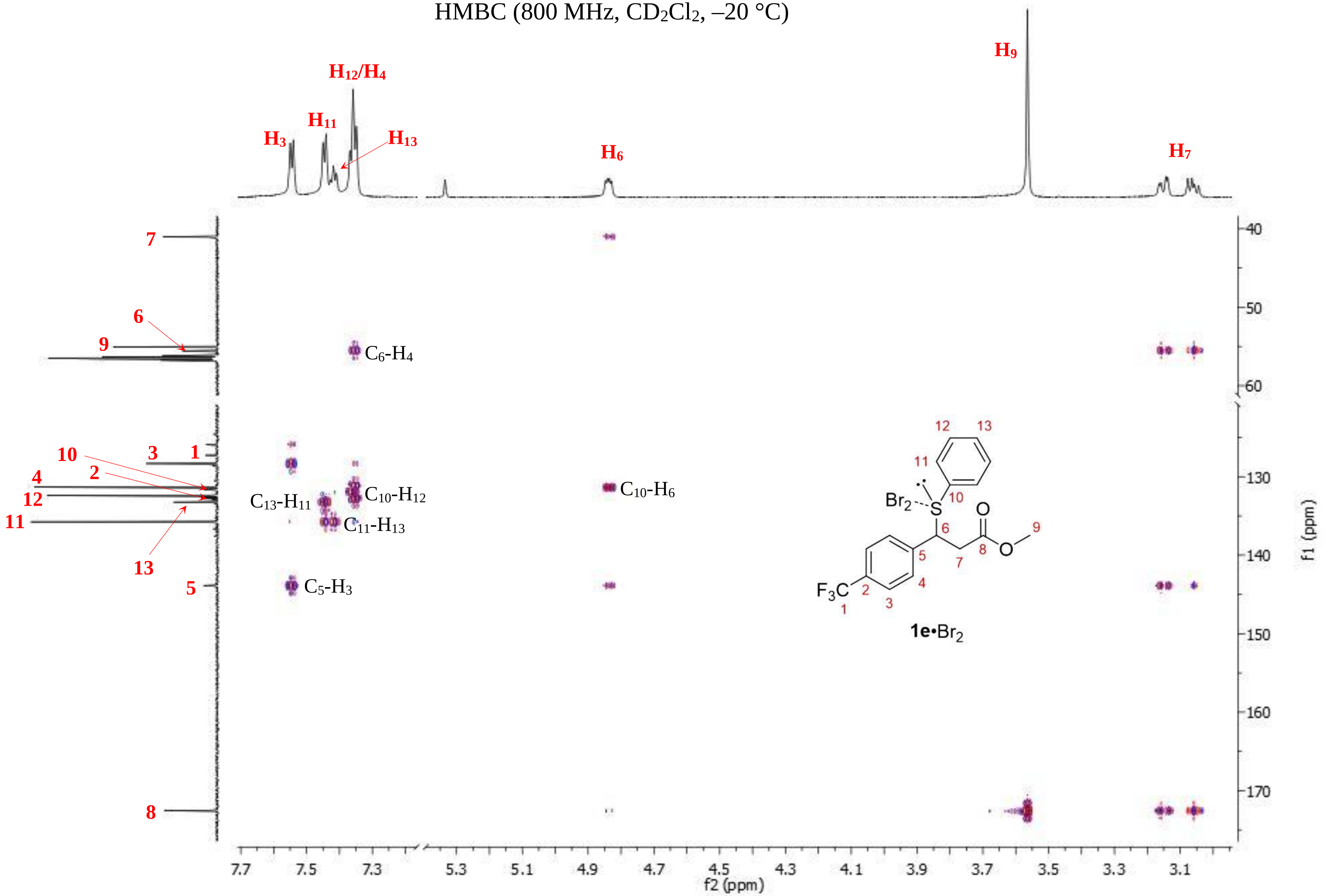

^{1}H NMR monitoring of the conversion of (1e·Br_2) to bromide (4e) after raising the temperature to 20 °C

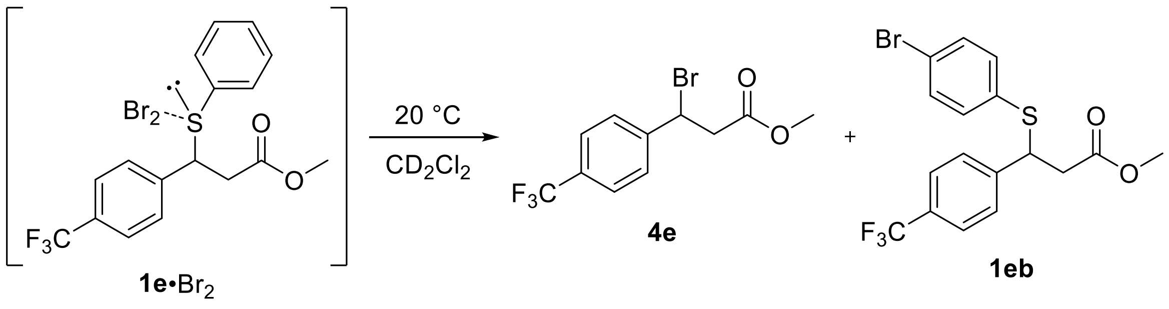

^{1}H NMR spectra (Fig. 4 in the manuscript) were recorded at 15 min intervals over a period of 14.5 h. The first ^{1}H NMR spectrum was recorded after monitoring the reaction at –20 °C for 4 h. The conditioning of the NMR spectrometer to reach 20 °C took around 30 min.

10.1.2. ^{1}H NMR monitoring (400 MHz) of the bromination of (1e) at –20 °C

^{1}H NMR spectra were recorded at 5 min intervals over a period of 650 min. The reaction was performed in an NMR tube as described in section 10.1.1.

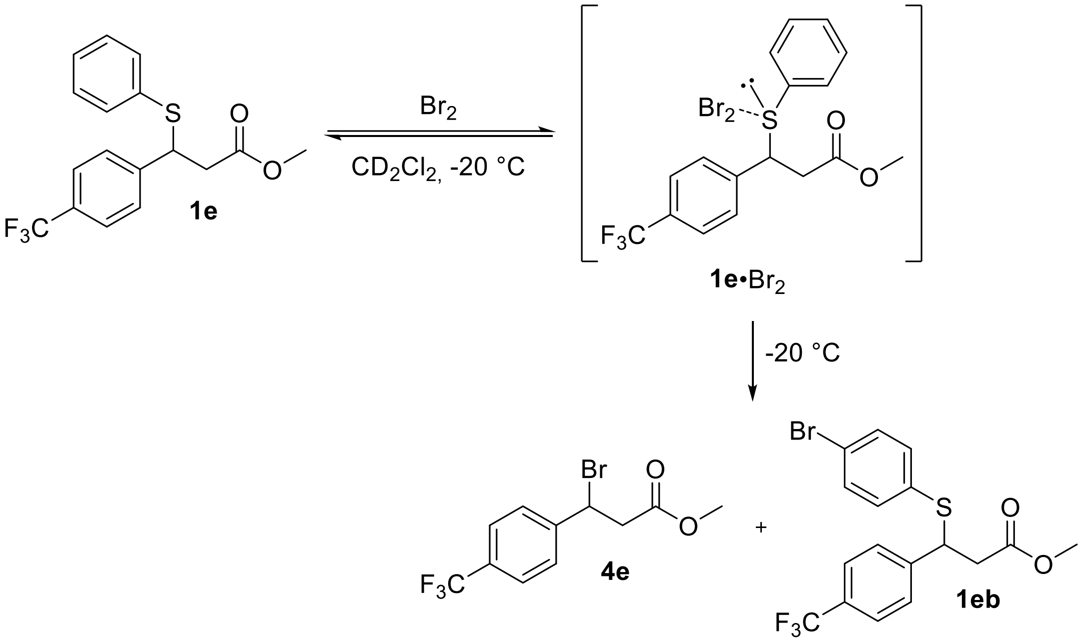

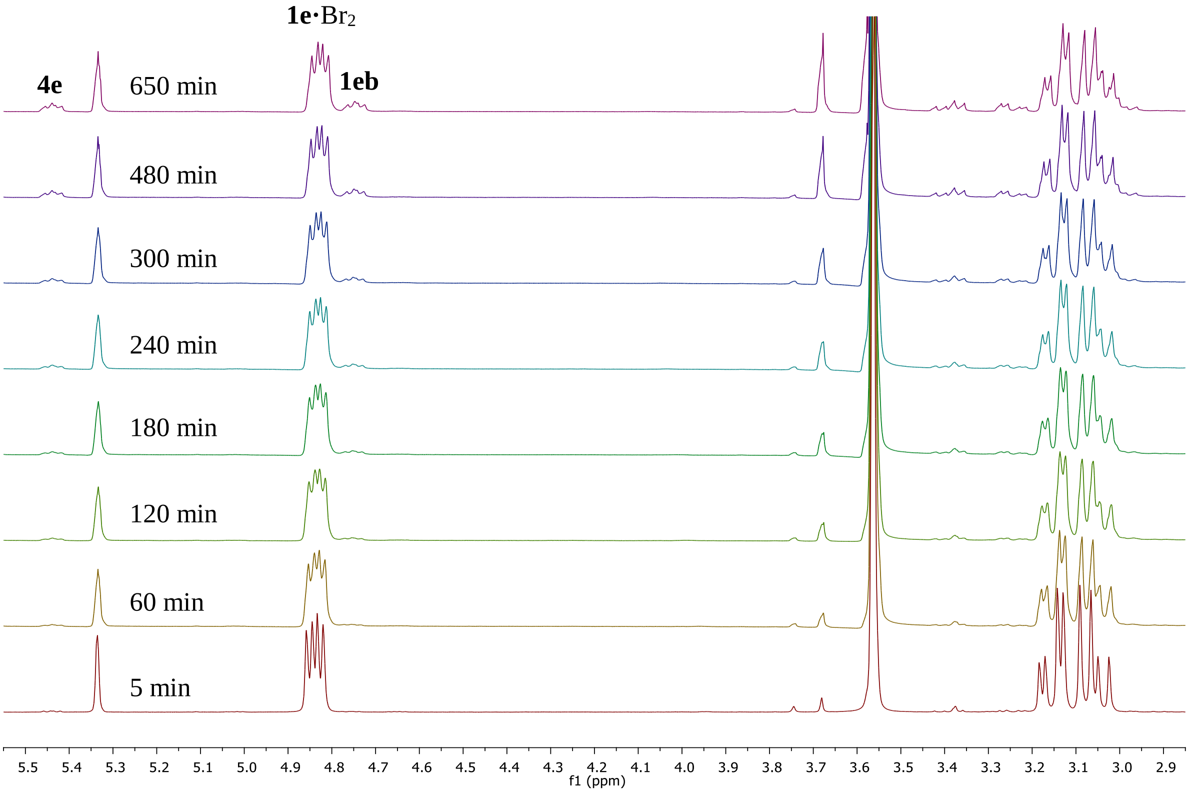

10.1.3. Monitoring the bromination of (1e) by quantitative ^{1}H NMR at 20 °C

Dibromomethane (17 mg, 0.1 mmol, 1.0 equiv) as internal standard was added as a stock solution (0.6 mL, 0.17 M in CD_2Cl_2) to sulphide **1e** (34 mg, 0.1 mmol) under N_2 atmophere. The solution was transferred *via* syringe to a 5 mm NMR tube placed in a suba-seal capped Schlenk tube. The NMR tube was sealed with a precision seal rubber septum, which was covered with parafilm. A Br_2 solution (100 μL, 1.0 M in CD_2Cl_2, 0.1 mmol, 1.0 equiv) was added *via* syringe and a series of 1D NMR spectra (400 MHz, 20 °C) were recorded over a period of 60 hours.

Acquisition parameters for the NMR experiment were as follows: pulse sequence = zg, D1 = 60 sec, AQ = 8.17 sec, TD = 131072, SW = 20 ppm, DS = 0, NS = 16, RG = 64.

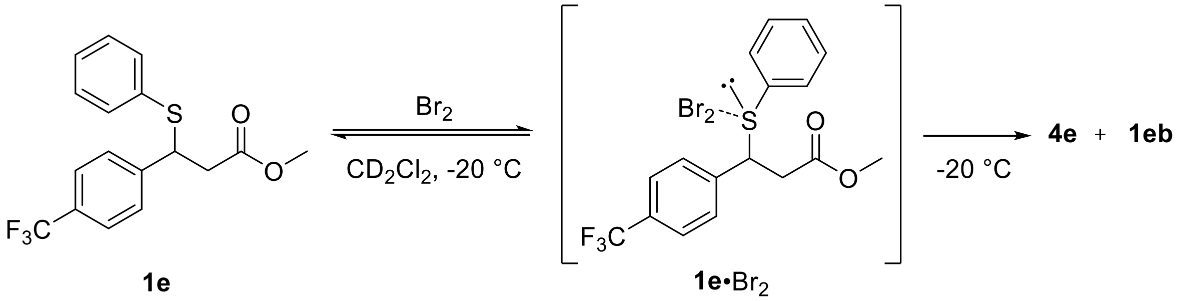

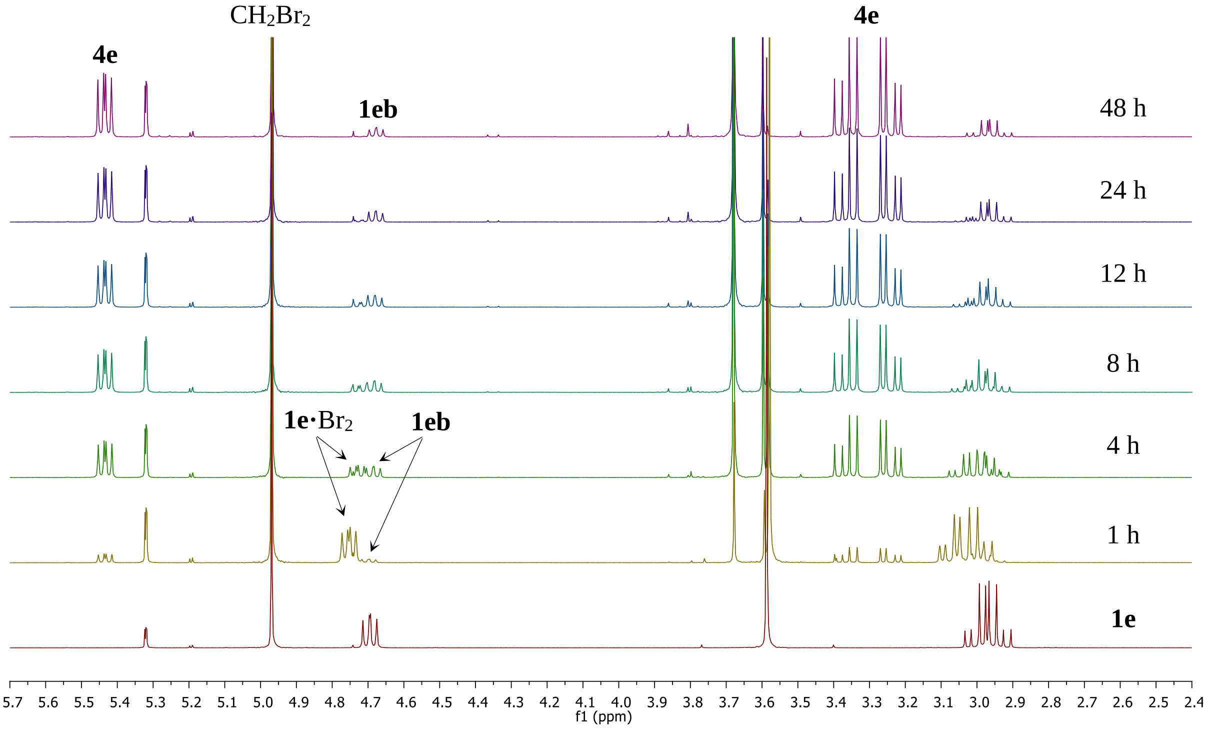

10.2. NMR monitoring of the bromination of sulphide (1eb)

10.2.1. Preparation of sulphide (1eb)

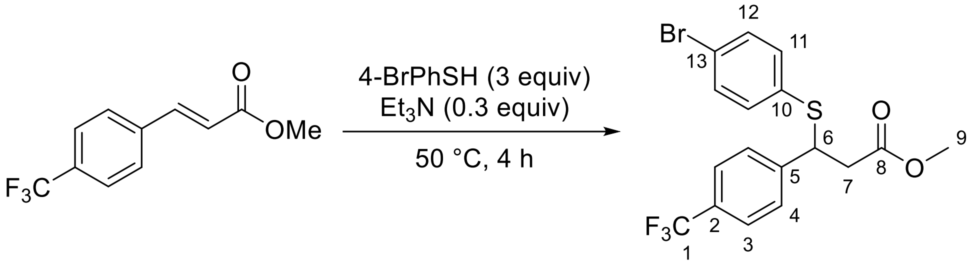

4-Bromothiophenol (1.70 g, 9.0 mmol, 3.0 equiv) and triethylamine (125 µL, 91 mg, 0.9 mmol, 0.3 equiv) were added to methyl 3-(4-(trifluoromethyl)phenyl)cinnamate (690 mg, 3.0 mmol) and the reaction mixture was stirred at 50 °C for 4 h. The reaction mixture was directly purified by flash column chromatography (silica gel; petroleum ether/CH_2Cl_2, 70:30 → 0:100) to afford the title compound as a yellow solid (1.19 g, 95% yield). Mp: 99–100 °C. IR (neat, cm^{-1}): *ν* 3129, 3084, 2761, 1623, 1572, 1251, 1289, 1020, 764. Full assignments were made based on HMBC and HSQC experiments. ^{1}H NMR (400 MHz, CD_2Cl_2): δ 2.83 (dd, *J* = 16.2, 8.7 Hz, 1H, H7), 2.89 (dd, *J* = 16.2, 7.3 Hz, 1H, H7), 3.50 (s, 3H, H9), 4.57 (virt t, *J* = 8.0 Hz, 1H, H6), 7.06 (d, *J* = 8.4 Hz, 2H, H11), 7.27–7.31 (m, 4H, H_4, H12), 7.45 (d, *J* = 8.2 Hz, 2H, H3). ^{19}F NMR (376 MHz, CD_2Cl_2) δ -62.8. ^{13}C NMR (101 MHz, CD_2Cl_2): δ 40.4 (C7), 49.1 (C6), 52.2 (C9), 122.8 (C13), 124.5 (q, *J* = 272.3 Hz, C1), 125.7 (q, *J* = 3.8, C3), 128.4 (C4), 129.8 (q, *J*= 32.4 Hz, C2), 132.4 (C12), 132.4 (C10), 135.4 (C11), 145.1 (C5), 170.8 (C8). HRMS (EI): $C_{17}H_{14}F_3O_2S$ $[M – Br]^+$ calculated: 339.0667, found: 339.0666.

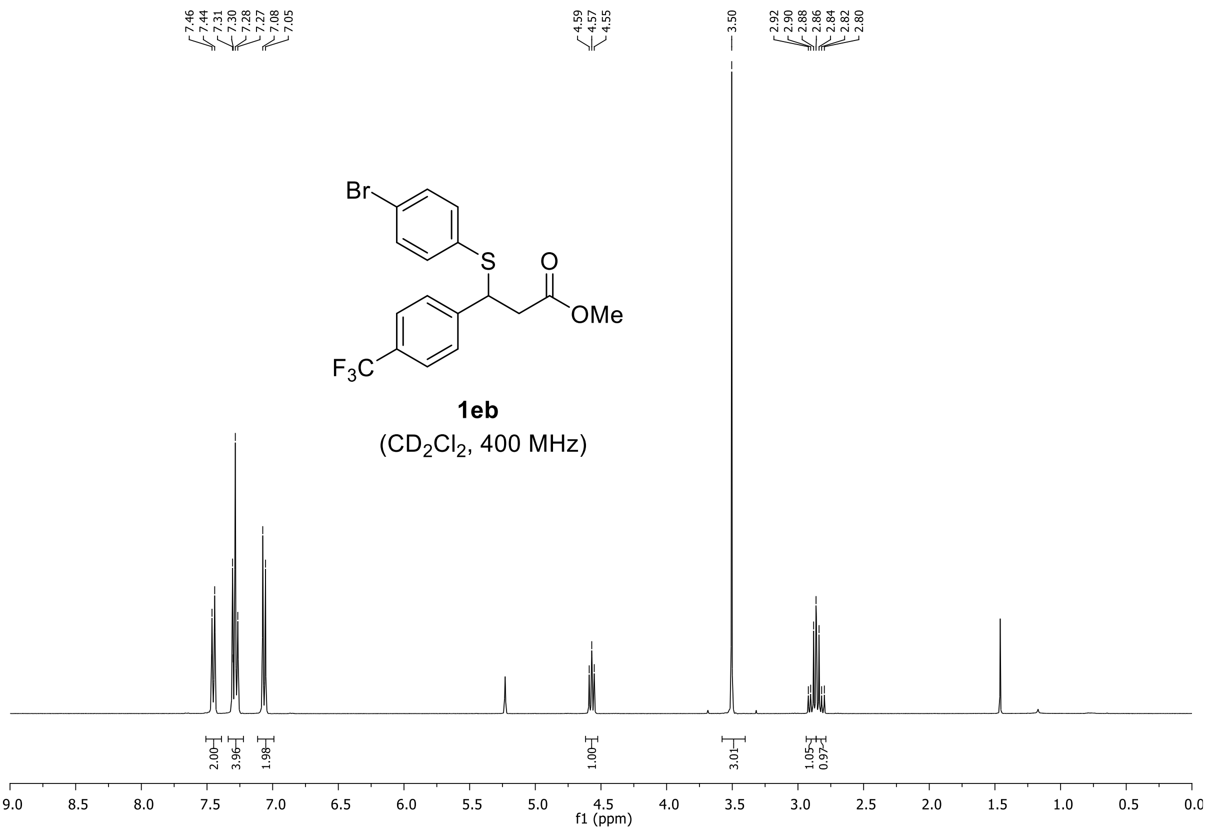
7.46
7.44
7.31
7.30
7.28
7.27
7.08
7.05
4.59
4.57
4.55
3.50
2.92
2.90
2.88
2.86
2.84
2.82
2.80
Br
S
O
OMe
F3C
1eb
(CD2Cl2, 400 MHz)
2.00
3.96
1.98
1.00
3.01
1.05
0.97
f1 (ppm)

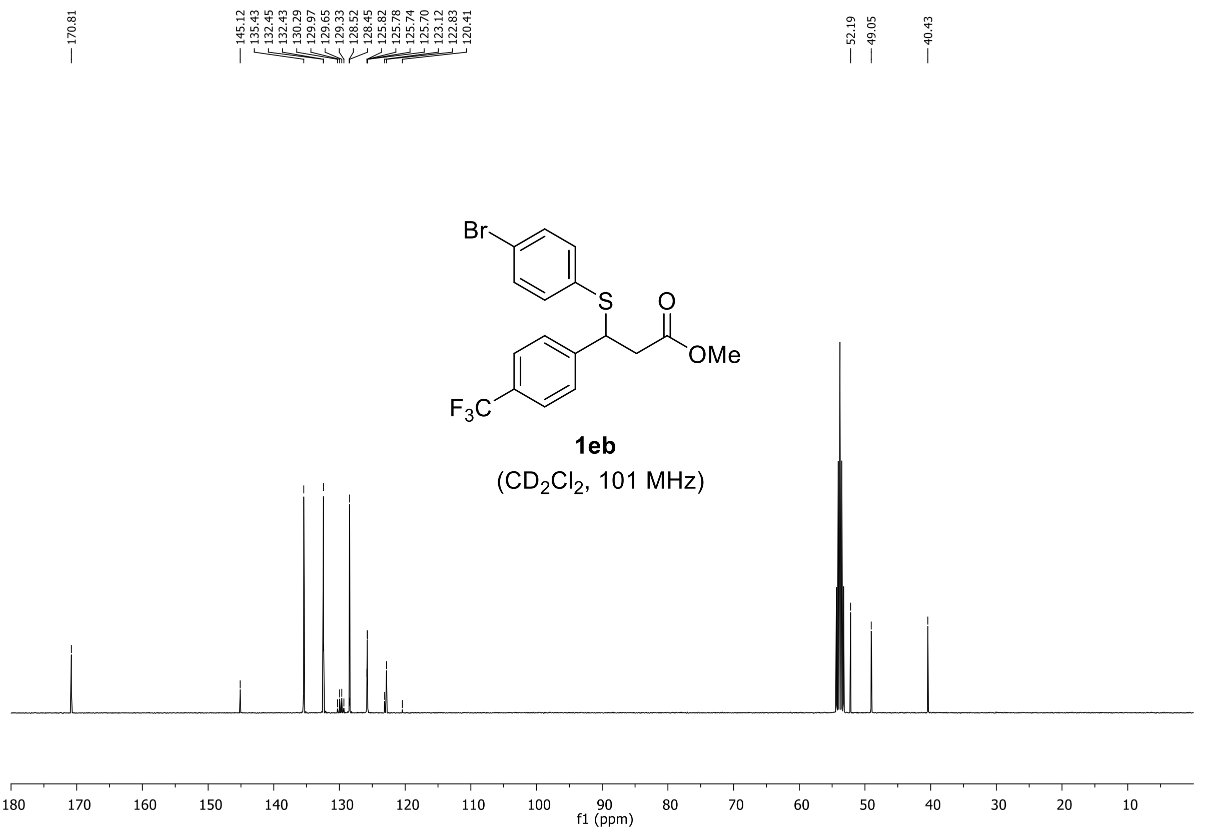
170.81
145.12
135.43
132.45
132.43
130.29
129.97
129.65
129.33
128.52
128.45
125.82
125.78
125.74
125.70
123.12
122.83
120.41
52.19
49.05
40.43
Br
S
O
OMe
F3C
1eb
(CD2Cl2, 101 MHz)
f1 (ppm)

10.2.2. Monitoring the bromination of sulphide (1eb) by NMR and characterisation of adduct (1eb·Br_2)

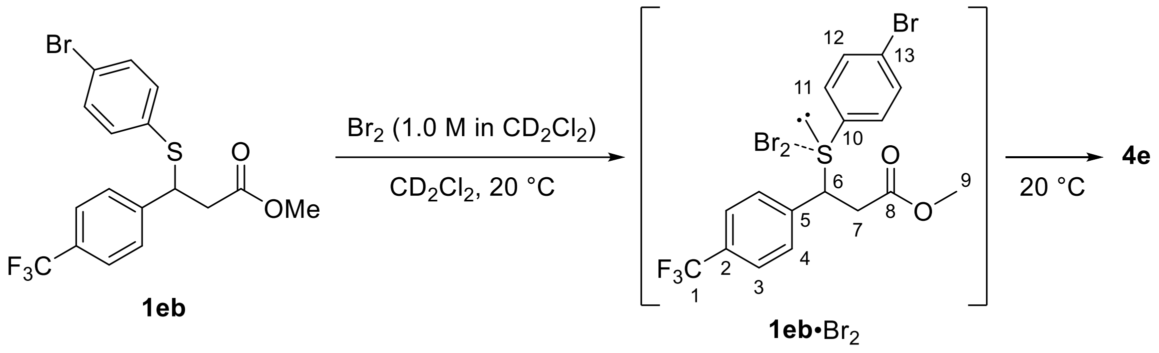

Dibromomethane solution (0.6 mL, 0.17 M in CD_2Cl_2, 0.1 mmol, 1.0 equiv), as internal standard, was added to a sulphide **1eb** (42 mg, 0.1 mmol) under N_2 atmophere. The solution was transferred *via* syringe in a 5 mm NMR tube placed in a suba-seal capped Schlenk tube. The NMR tube was sealed with a precision seal rubber septum and covered with parafilm. A Br_2 solution (100 μL, 1.0 M in CD_2Cl_2, 0.1 mmol, 1.0 equiv) was added *via* syringe and a series of 1D NMR (400 MHz, 20 °C) spectra were recorded over a period of 60 hours. Full NMR spectroscopic characterization of adduct **1eb**·Br_2 was achieved within the first hour.

^{1}H NMR (400 MHz, CD_2Cl_2): δ 2.96 (dd, J = 16.2, 8.7 Hz, 1H, H7), 3.03 (dd, J = 16.2, 6.7 Hz, 1H, H7), 3.59 (s, 3H, H9), 4.70 (virt t, J = 7.6 Hz, 1H, H6), 7.20 (d, J = 8.3 Hz, 2H, H11), 7.36 (d, J = 8.1 Hz, 2H, H4), 7.42 (d, J = 8.3 Hz, 2H, H12), 7.55 (d, J = 8.1 Hz, 2H, H3).

^{19}F NMR (376 MHz, CD_2Cl_2) δ –62.9.

^{13}C NMR (101 MHz, CD_2Cl_2): δ 39.9 (C7), 50.2 (C6), 52.3 (C9), 123.6 (br s, C13), 124.1 (q, J = 271.6 Hz, C1), 125.8 (q, J = 3.8, C3), 128.6 (C4), 130.4 (q, J= 32.4 Hz, C2), 131.3 (br s, C10), 132.6 (C12), 135.3 (C11), 143.3 (br s, C5), 170.5 (C8).

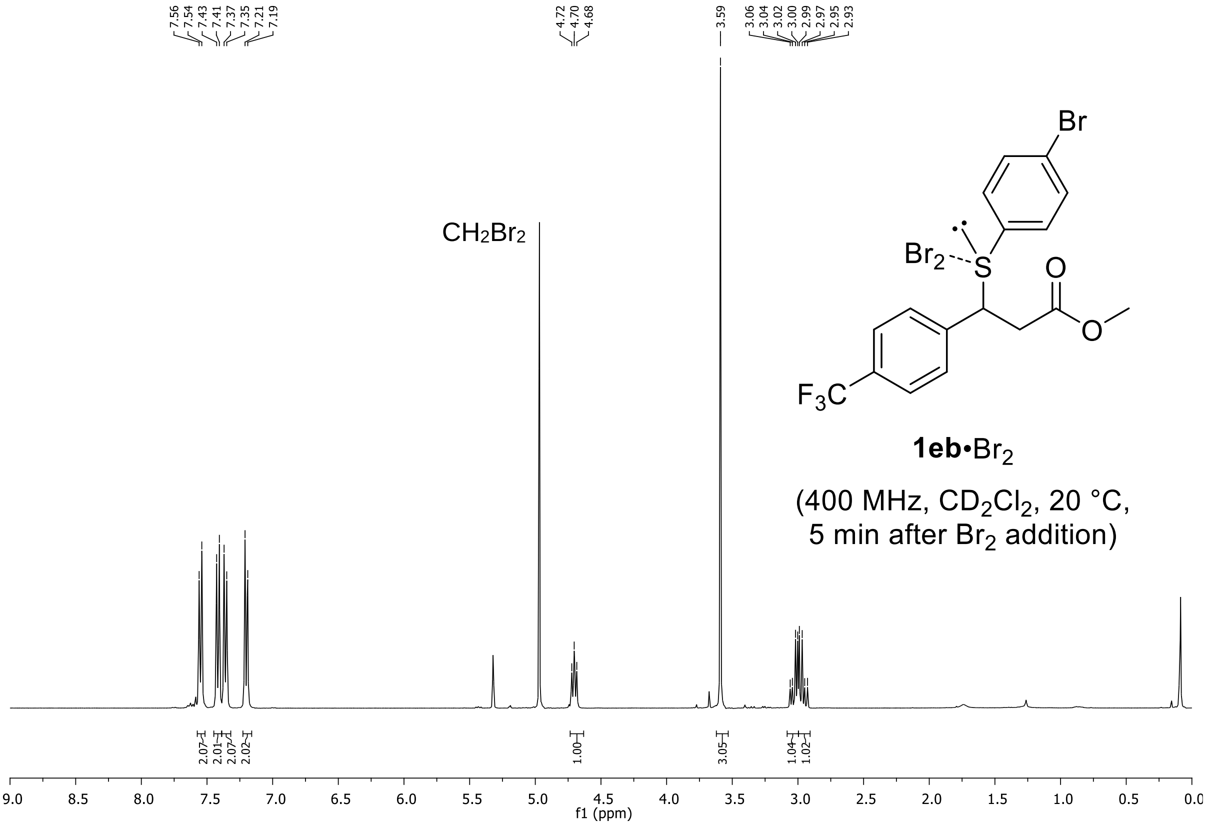
7.56
7.54
7.43
7.41
7.37
7.35
7.21
7.19
4.72
4.70
4.68
3.59
3.06
3.04
3.02
3.00
2.99
2.97
2.95
2.93
CH2Br2
Br
Br2
S
O
O
F3C
1eb•Br2
(400 MHz, CD2Cl2, 20 °C,
5 min after Br2 addition)
2.07
2.01
2.07
2.02
1.00
3.05
1.04
1.02
f1 (ppm)

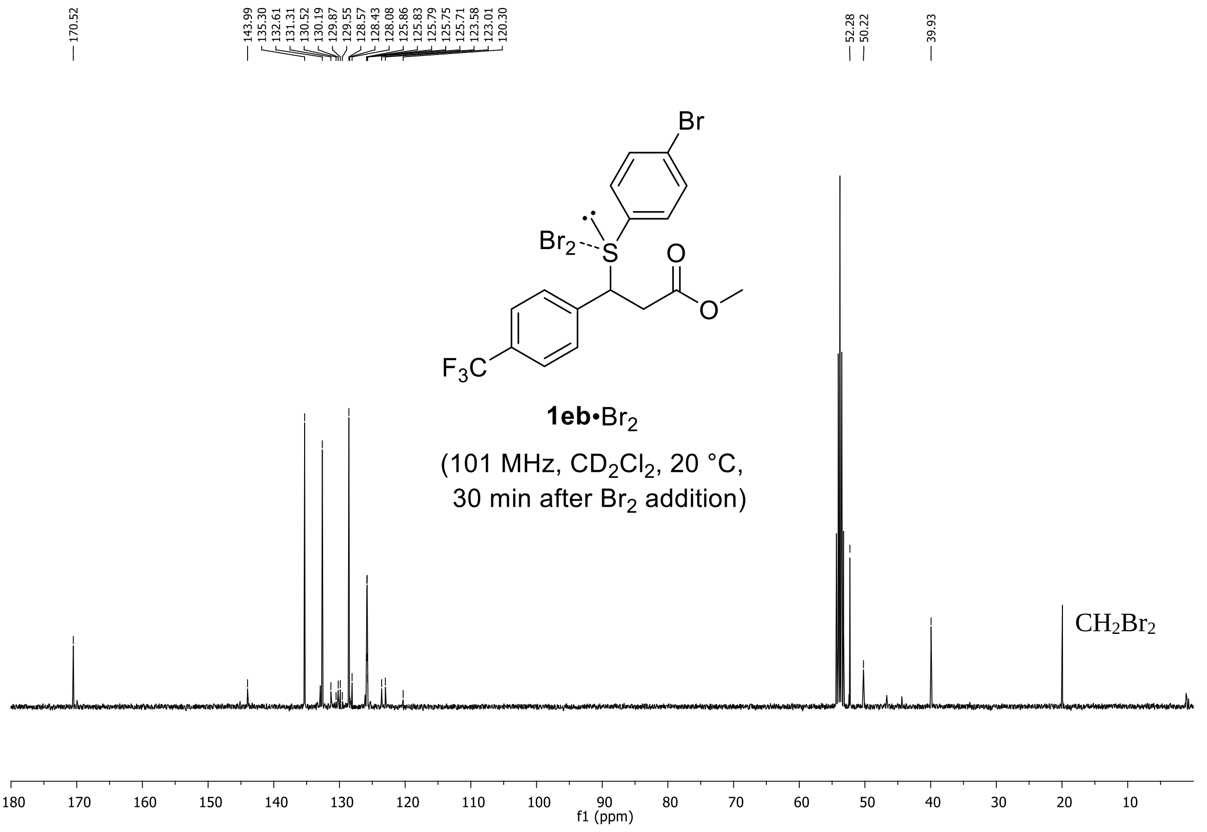
170.52
143.99
135.30
132.61
131.31
130.52
130.19
129.87
129.55
128.57
128.43
128.08
125.86
125.83
125.79
125.75
125.71
123.58
123.01
120.30
52.28
50.22
39.93
Br
Br2
S
O
O
F3C
1eb•Br2
(101 MHz, CD2Cl2, 20 °C,
30 min after Br2 addition)
CH2Br2
f1 (ppm)

10.2.3. NMR monitoring of the bromination of (1eb) at 20 °C

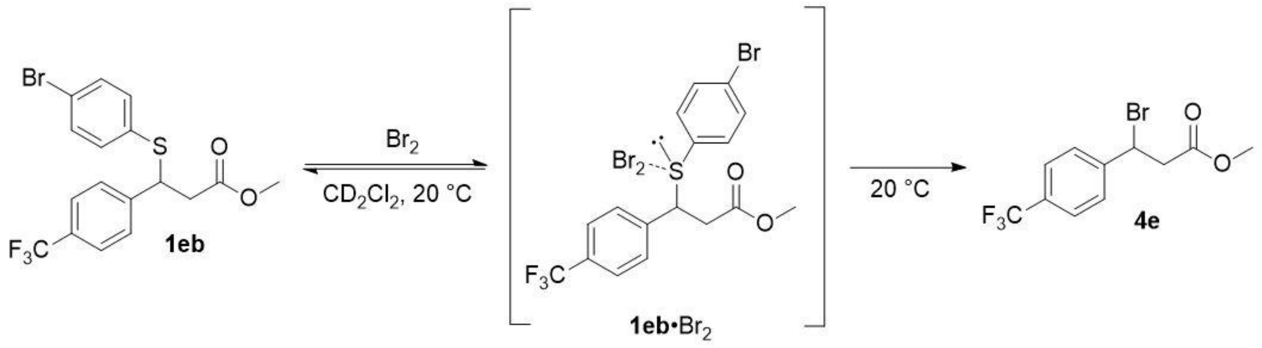

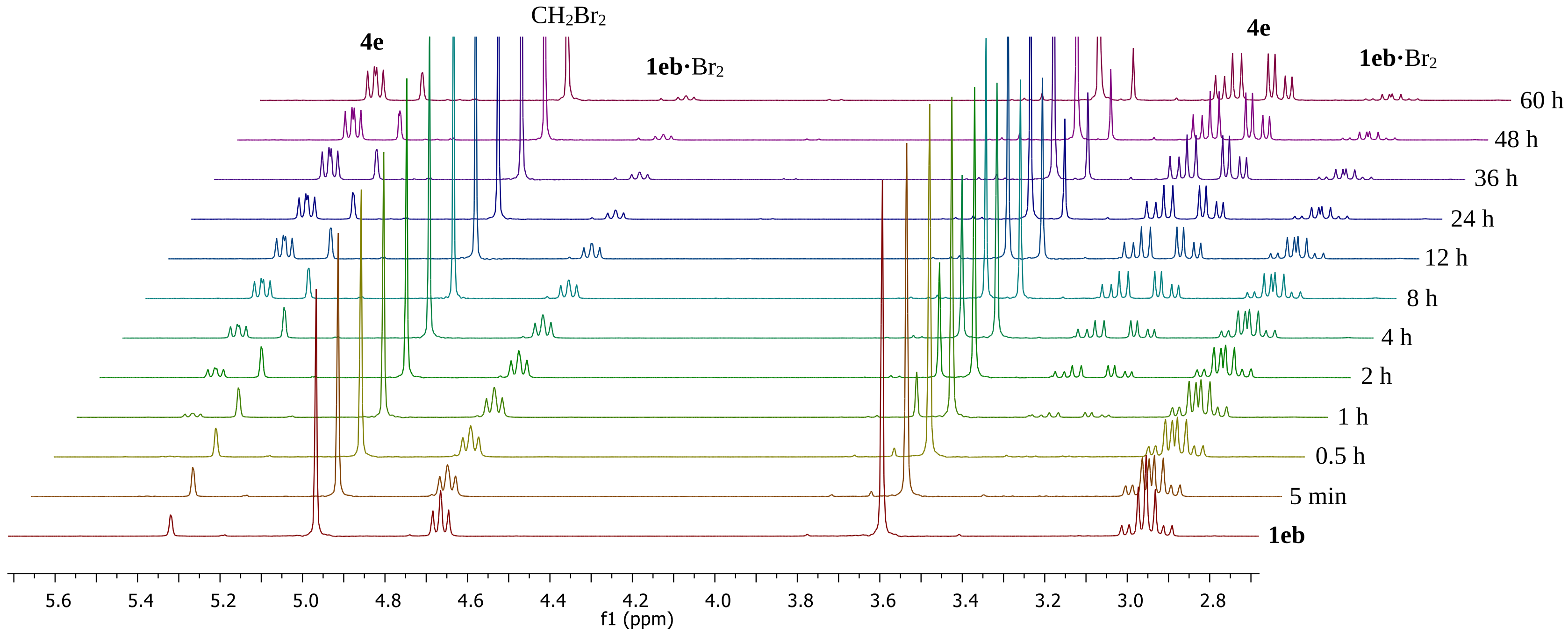

11. References

1. B. List, A. Doehring, M. T. H. Fonseca, K. Wobser, H. van Thienen, R. R. Torres and P. L. Galilea, *Adv. Synth. Catal.*, 2005, **347**, 1558–1560.
2. X. Fang, J. Li and C.-J.Wang, *Org. Lett.*, 2013, **15**, 3448–3451.
3. K. Tanaka, S. Kishigami and F. Toda, *J.Org. Chem.*, 1991, **56**, 4333–4334.
4. D. Canestrari, S. Lancianesi, E. Badiola, C. Strinna, H. Ibrahim and M.F.A. Adamo, *Org. Lett.*, 2017, **19**, 918–921.
5. J. Mirek, E. Chojnacka-Wojcik, E. Tatarzynska and B. Wiczynska, *Farmaco, Ed. Sci*, 1985, **9**, 701–708.
6. S. Gao, T. Tzeng, M. N. V. Sastry, C.-M. Chu, Ju-T. Liu, C. Lin and C.-Fa Yao, *Tetrahedron Lett*., 2006, **47**, 1889–1893.
7. M. Hans, L. Delaude, J. Rodriguez and Y. Coquerel, *J. Org. Chem.*, 2014, **79**, 2758–2764.
8. F. Fleming and J. Pak, *J. Org. Chem.*, 1995, **60**, 4299–4301.
9. B. Movassagh and M.Soleiman-Beigi, *Monatsh Chem*, 2009, **140**, 409–411.
10. B. Janza and A. Studer, *Org. Lett*., 2006, **8**, 1875–1878.
11. V. V. Thakur, M. D. Nikallje and A. Sudalai, *Tetrahedron: Asymmetry*, 2003, **14**, 581–586.
12. Ch. H. Dusseau, S. E. Schaafsma and Th. J. de Boer, *Recl. Trav. Chim. Pays-Bas*, 1970, **89**, 535–544.
13. V. L. Vitalij, A. A. Zemtsov, I. S. Marina and A. D. Dilman, *Org. Lett.*, 2013, **15**, 917–919.
14. O. Brücher, U. Bergsträßer, H. Kelm, J. Hartung, M. Greb, I. Svoboda and H Fuess, *Tetrahedron*, 2012, **68**, 6968–6980.
15. T. Imai and S. Nishida, *J. Org. Chem*, 1980, **45**, 2354-2359.
16. R. F. Klima, A. V. Jadhav, P. N. D. Singh, M. Chang, C. Vanos, J. Sankaranarayanan, M. Vu, N. Ibrahim, E. Ross, S. McCloskey, R. S. Murthy, J. A. Krause, B. S. Ault and A. D. Gudmundsdóttir, *J. Org Chem.*, 2007, **72**, 6372–6381.
17. J. Andersen, N. Stuhr-Hansen, L. Gr. Zachariassen, H. Koldsø, B. Schiott, K. Strømgaard and A. S. Kristensen, *Molecular Pharmacology*, 2014, **85**, 703–714.
18. A. Isleyen, C. Tanyeli and O. Dogan, *Tetrahedron: asymmetry*, 2006, **17**, 1561–1567.
19. X. Tan, T. Song, Z. Wang, H. Chen, L. Cui and C. Li, *Org. Lett*., 2017, **19**, 1634–1637.
20. J. Chen, J.-H. Lin and J.-C. Xiao, *Org. Lett.*, 2018, **20**, 3061-3064.

21. X. Lu, Y. Wang, B. Zhang, J.-J. Pi, X.-X. Wang, T.-J. Gong, B. Xiao and Y. Fu, *J. Am. Chem. Soc.*, 2017, **139**, 12632–12637.
22. A. Isleyen, C. Tanyeli and O. Dogan, *Tetrahedron: asymmetry*, 2006, **17**, 1561–1567.
23. K. Sahasrabudhe, V. Gracias, K. Fumess, B. T. Smith, C. E. Katz, D. S. Reddy and J. Aubé, *J. Am. Chem. Soc.*, 2003, **125**, 7914–7922.

12. HPLC traces of optically active compounds

12.1 HPLC traces of (±)-1a and (–)-(*S*)-1a

CHIRALPAK IB-H, *n*-hexane/*i*-PrOH, 99:1, 1 mL/min, 210 nm

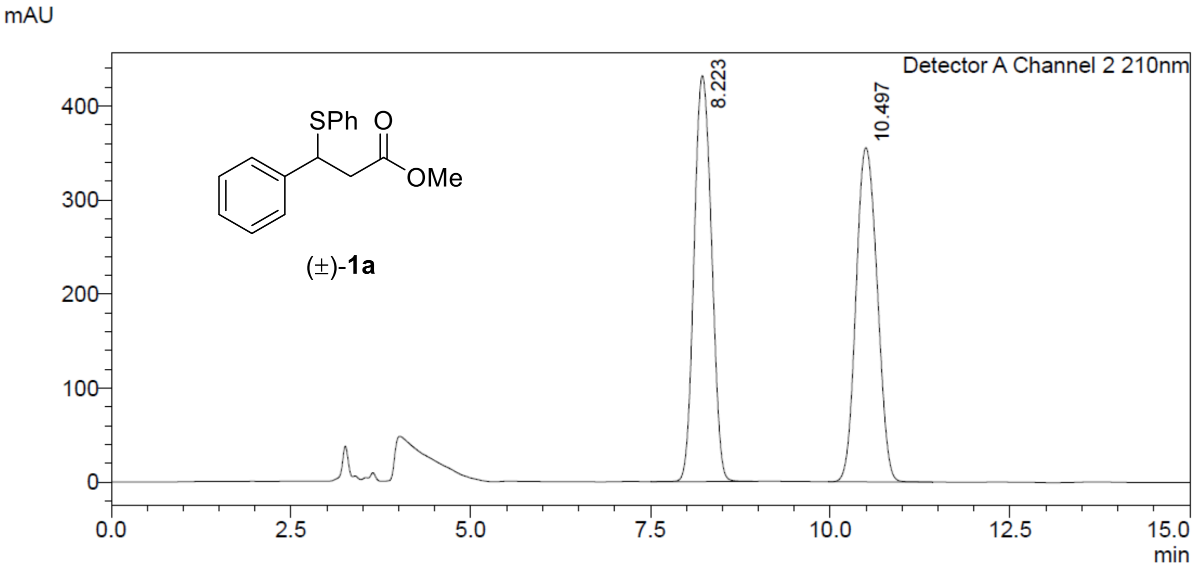

<Peak Table>

Detector A Channel 2 210nm

Peak#	Ret. Time	Area	Height	Conc.
1	8.223	7291578	431520	50.042
2	10.497	7279464	355532	49.958
Total		14571042	787052	

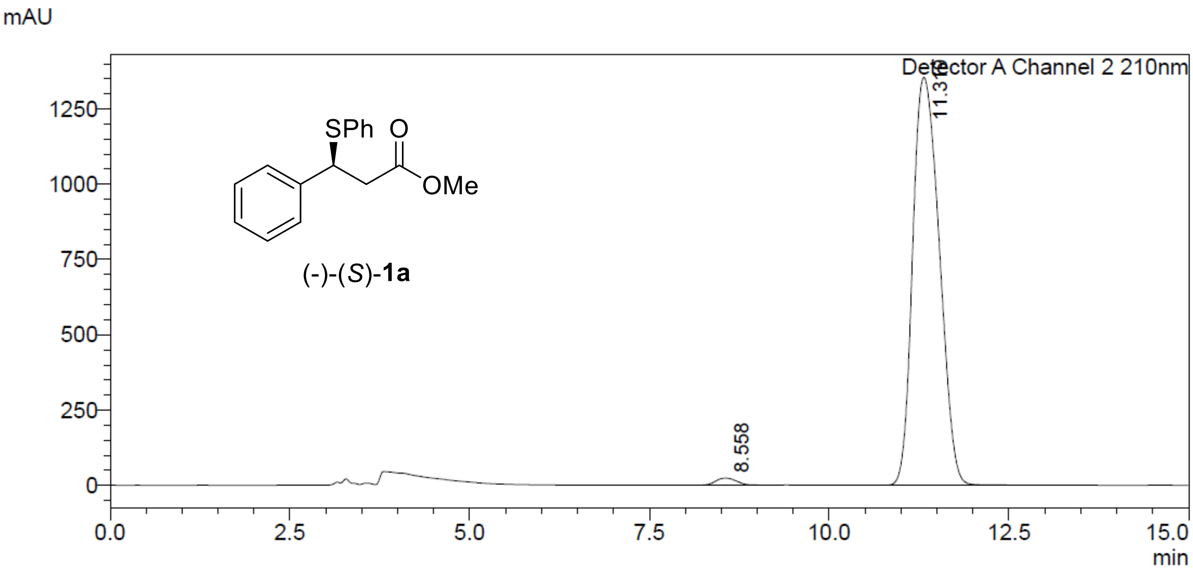

<Peak Table>

Detector A Channel 2 210nm

Peak#	Ret. Time	Area	Height	Conc.
1	8.558	469447	23451	1.328
2	11.319	34878064	1354444	98.672
Total		35347510	1377894	

12.2 HPLC traces of (±)-1b and (–)-(*S*)-1b

CHIRALPAK OD-H, *n*-hexane/*i*-PrOH, 95:5, 1.0 mL/min, 210 nm

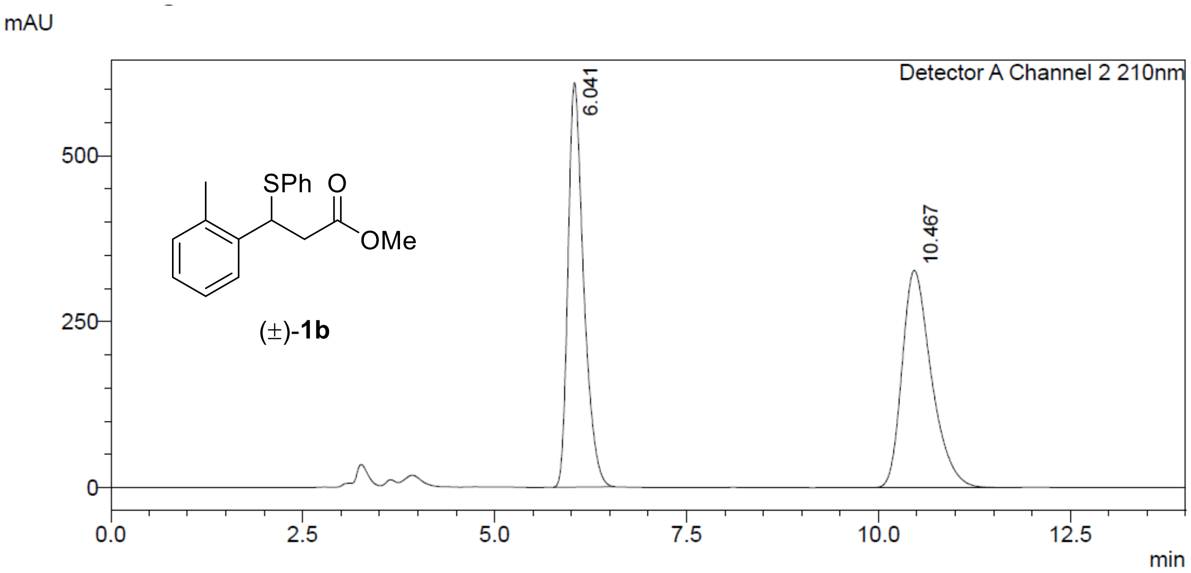

<Peak Table>

Detector A Channel 2 210nm

Peak#	Ret. Time	Area	Height	Conc.
1	6.041	8639711	608650	0.000
2	10.467	8719593	327290	0.000
Total		17359305	935940	

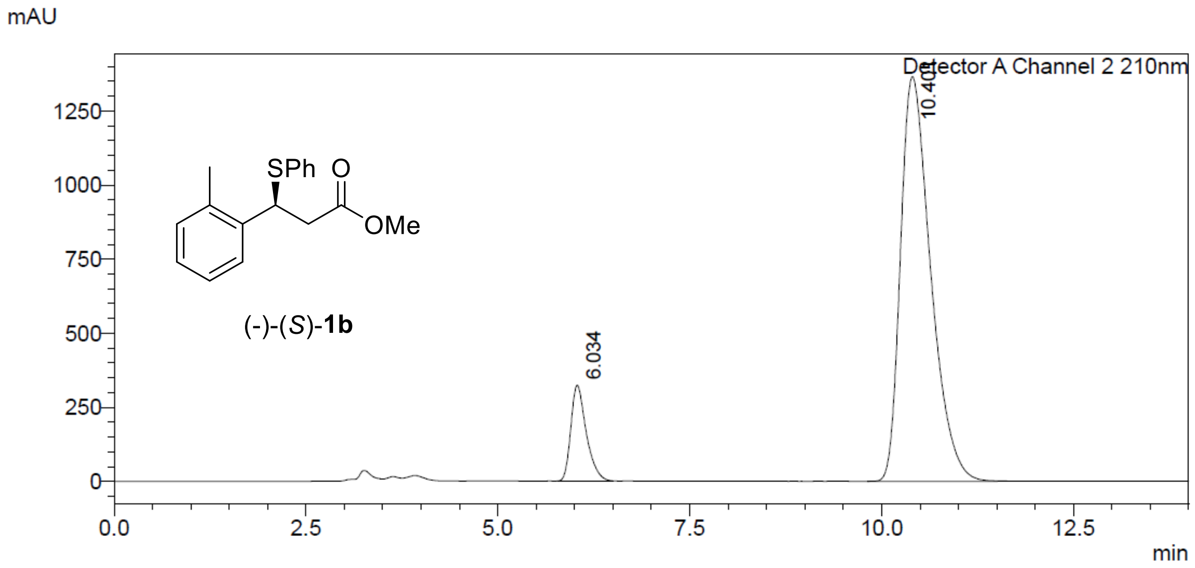

<Peak Table>

Detector A Channel 2 210nm

Peak#	Ret. Time	Area	Height	Conc.
1	6.034	4526863	323139	0.000
2	10.401	37284492	1365360	0.000
Total		41811355	1688500	

12.3 HPLC traces of (±)-1d and (–)-(*S*)-1d

CHIRALPAK OD-H, *n*-hexane/*i*-PrOH, 95:5, 0.5 mL/min, 210 nm

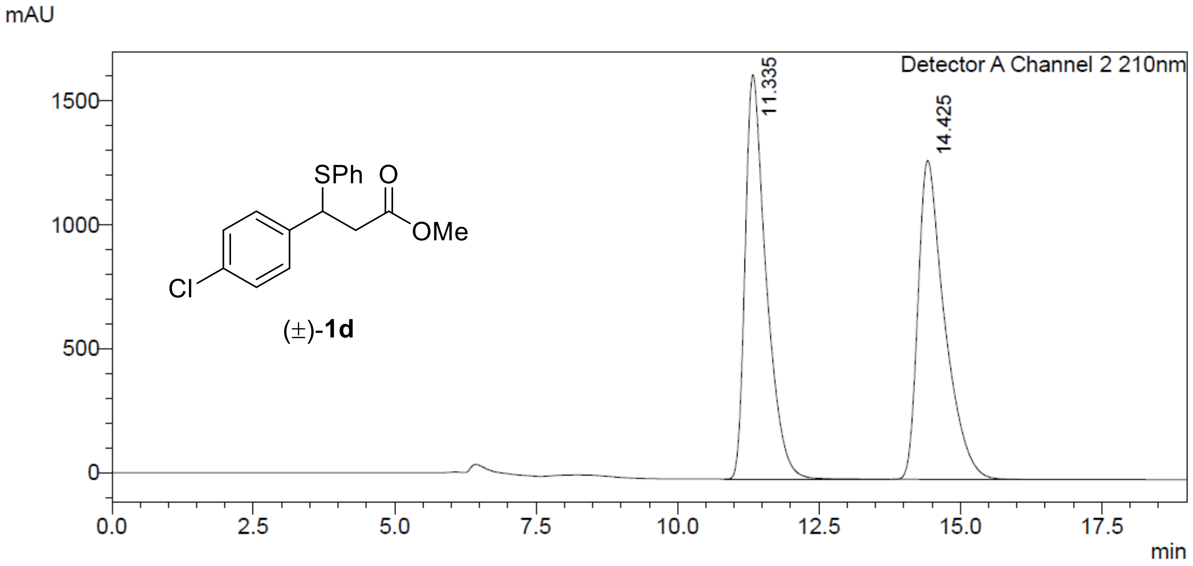

<Peak Table>

Detector A Channel 2 210nm

Peak#	Ret. Time	Area	Height	Conc.
1	11.335	42177480	1629115	0.000
2	14.425	42089734	1285541	0.000
Total		84267214	2914656	

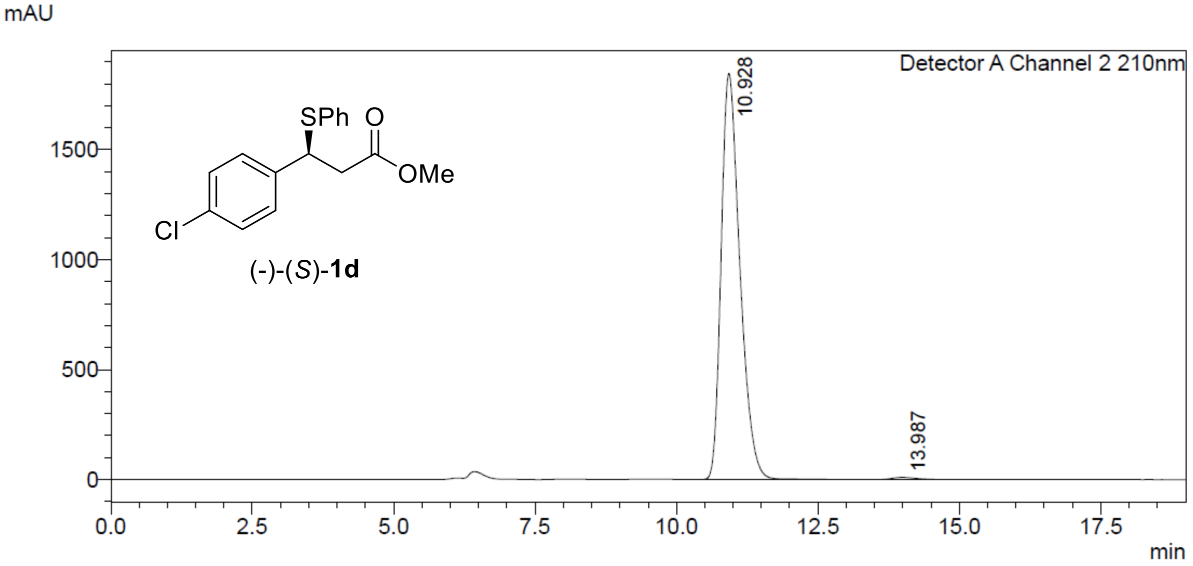

<Peak Table>

Detector A Channel 2 210nm

Peak#	Ret. Time	Area	Height	Conc.
1	10.928	42627957	1846094	0.000
2	13.987	309380	10727	0.000
Total		42937337	1856821	

12.4 HPLC traces of (±)-1e and (–)-(*S*)-1e

CHIRALPAK OD-H, *n*-hexane/*i*-PrOH, 95:5, 0.5 mL/min, 210 nm

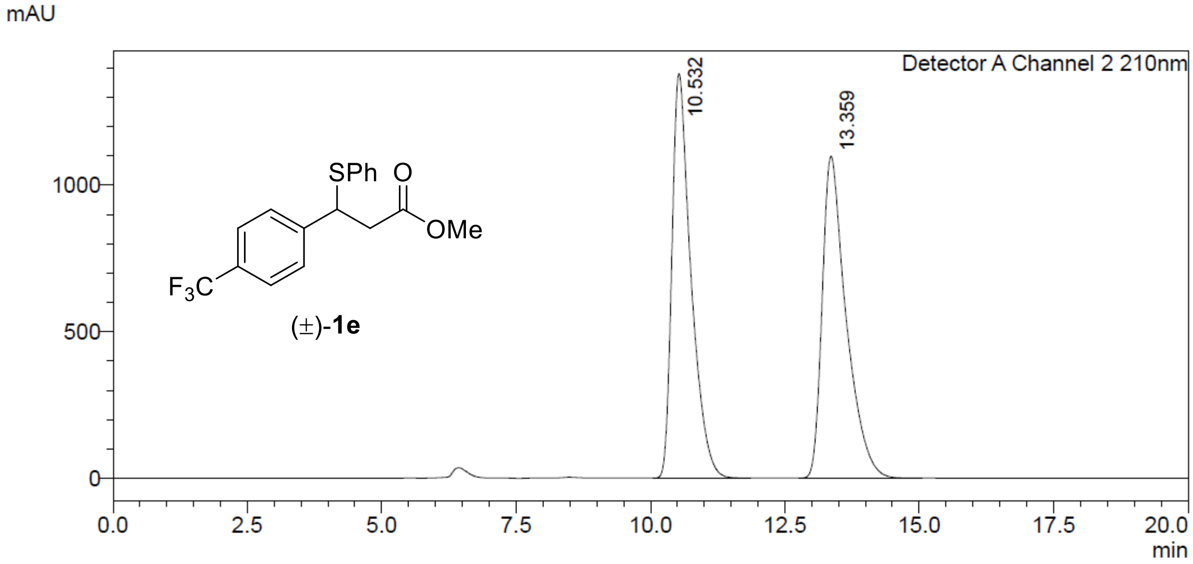

<Peak Table>

Detector A Channel 2 210nm

Peak#	Ret. Time	Area	Height	Conc.
1	10.532	34026823	1380212	0.000
2	13.359	34095662	1098885	0.000
Total		68122485	2479097	

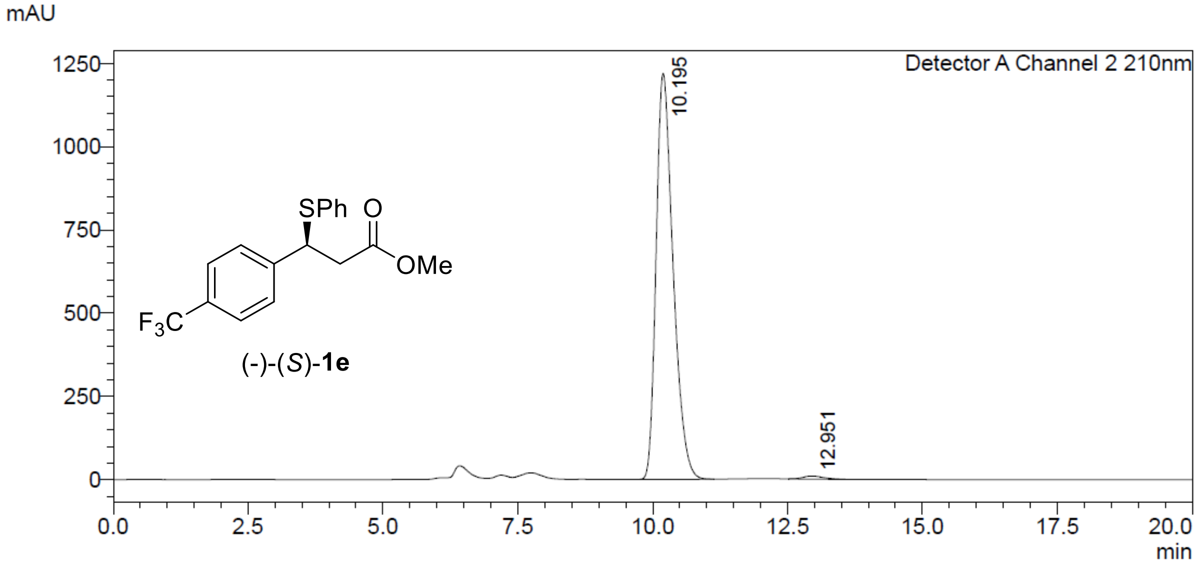

<Peak Table>

Detector A Channel 2 210nm

Peak#	Ret. Time	Area	Height	Conc.
1	10.195	26687256	1218413	0.000
2	12.951	244744	8939	0.000
Total		26931999	1227352	

12.5 HPLC traces of (±)-4a and (+)-(*R*)-4a

CHIRALPAK IB-H, *n*-hexane/*i*-PrOH, 95:5, 0.75 mL/min, 210 nm

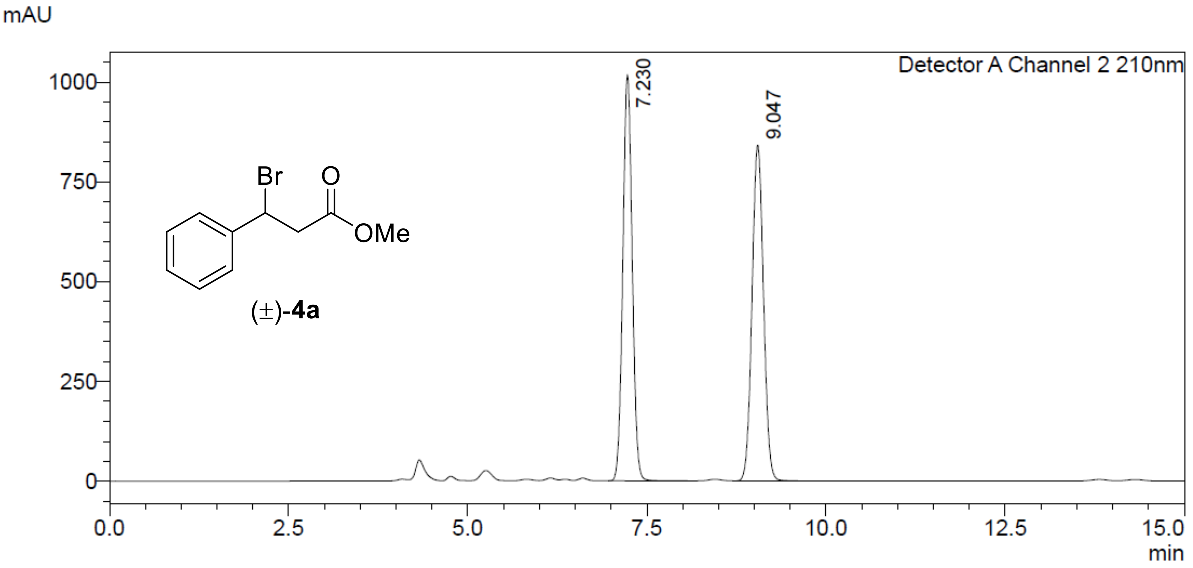

<Peak Table>

Detector A Channel 2 210nm

Peak#	Ret. Time	Area	Height	Conc.
1	7.230	9306586	1015608	49.794
2	9.047	9383654	840673	50.206
Total		18690240	1856280	

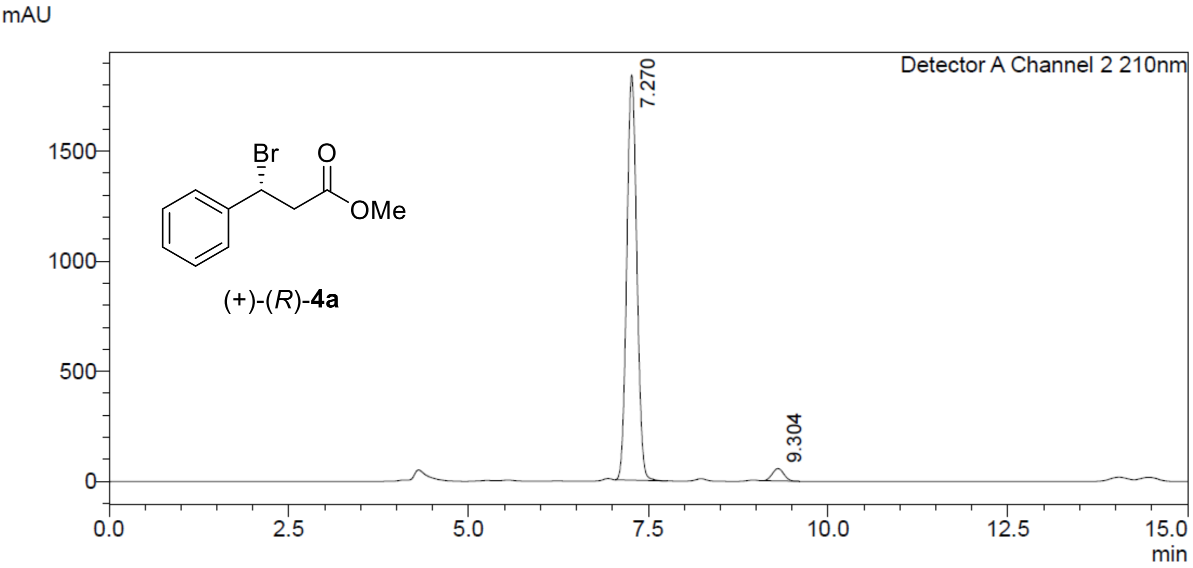

<Peak Table>

Detector A Channel 2 210nm

Peak#	Ret. Time	Area	Height	Conc.
1	7.270	17884055	1838325	96.529
2	9.304	643017	56761	3.471
Total		18527072	1895086	

12.6 HPLC traces of (±)-4b and (–)-(*R*)-4b

CHIRALPAK IB-H, *n*-hexane/*i*-PrOH, 95:5, 0.75 mL/min, 210 nm

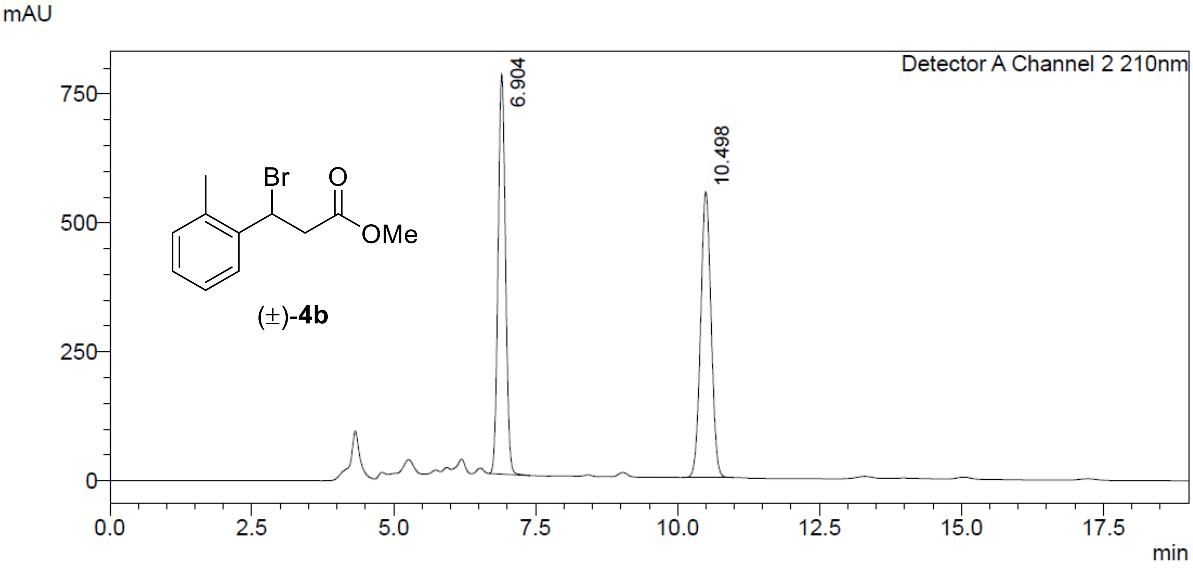

<Peak Table>

Detector A Channel 2 210nm

Peak#	Ret. Time	Area	Height	Conc.
1	6.904	7030763	775636	49.657
2	10.498	7127861	554224	50.343
Total		14158625	1329860	

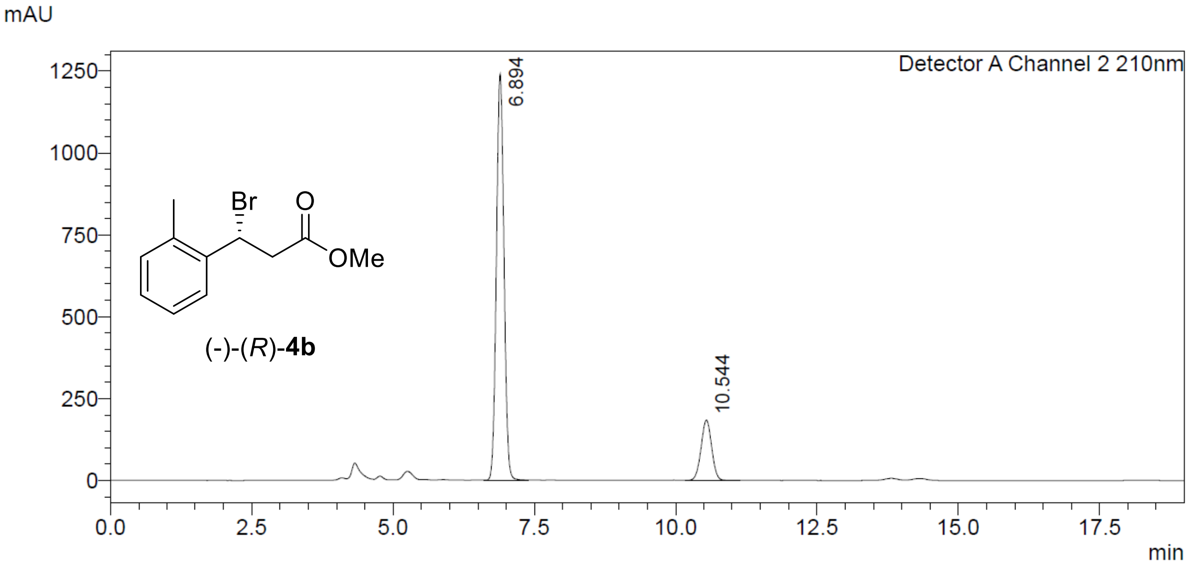

<Peak Table>

Detector A Channel 2 210nm

Peak#	Ret. Time	Area	Height	Conc.
1	6.894	11550607	1239283	82.770
2	10.544	2404512	184018	17.230
Total		13955118	1423301	

12.7 HPLC traces of (±)-4d and (+)-(*R*)-4d

CHIRALPAK IB-H, *n*-hexane/*i*-PrOH, 98:2, 0.5 mL/min, 210 nm

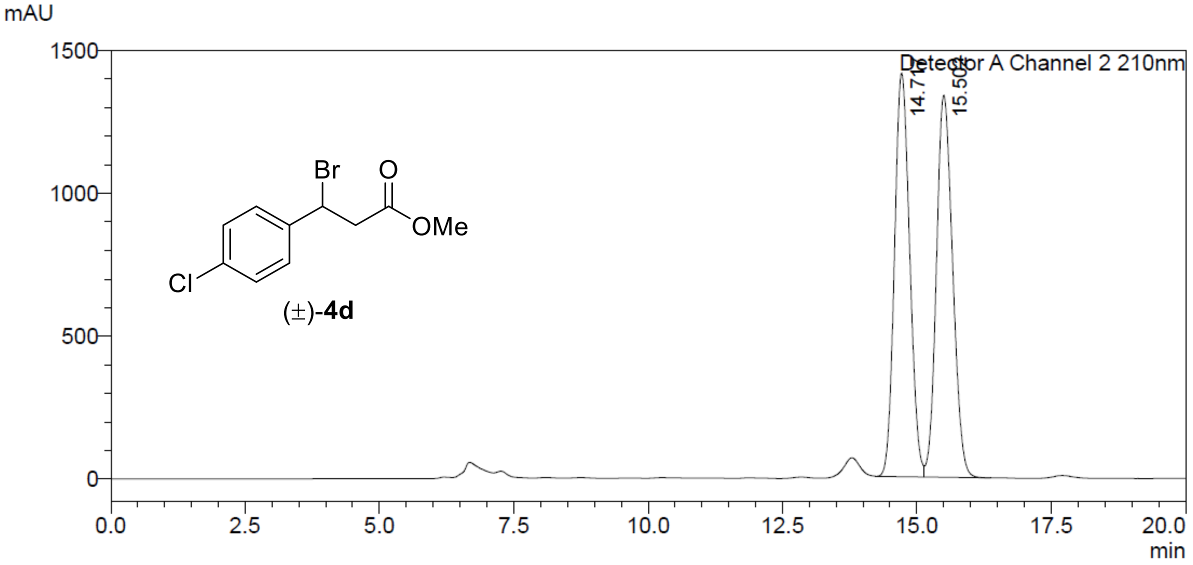

<Peak Table>

Detector A Channel 2 210nm

Peak#	Ret. Time	Area	Height	Conc.
1	14.717	27839185	1412659	49.706
2	15.502	28168814	1338193	50.294
Total		56007999	2750852	

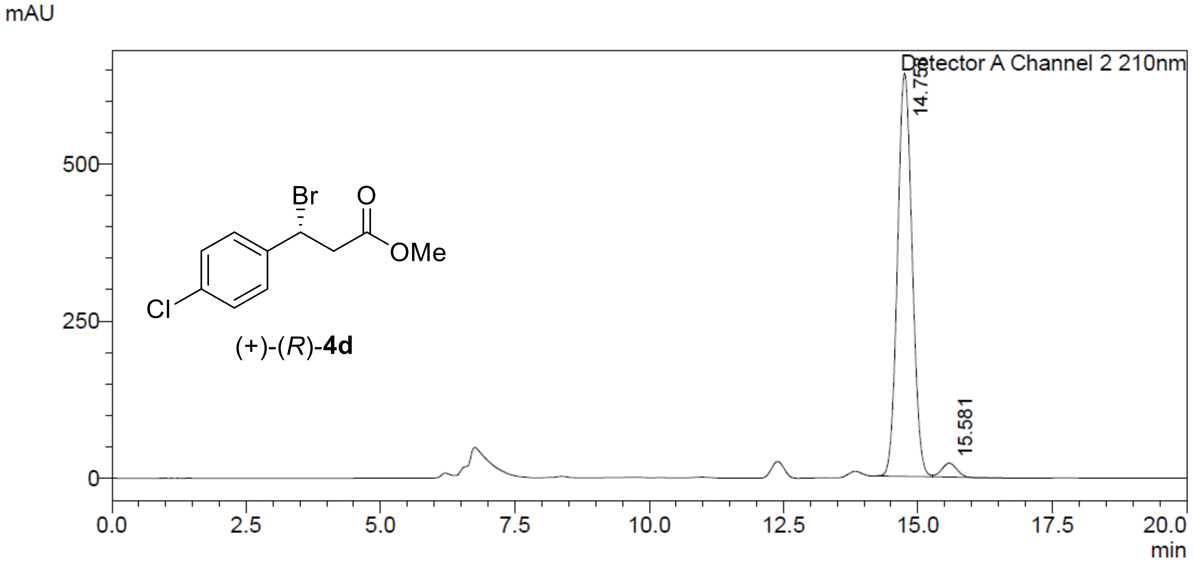

<Peak Table>

Detector A Channel 2 210nm

Peak#	Ret. Time	Area	Height	Conc.
1	14.753	12145607	640981	96.720
2	15.581	411886	22092	3.280
Total		12557493	663074	

12.8 HPLC traces of (±)-4e and (+)-(*R*)-4e

CHIRALPAK IB-H, *n*-hexane/*i*-PrOH, 98:2, 0.5 mL/min, 210 nm

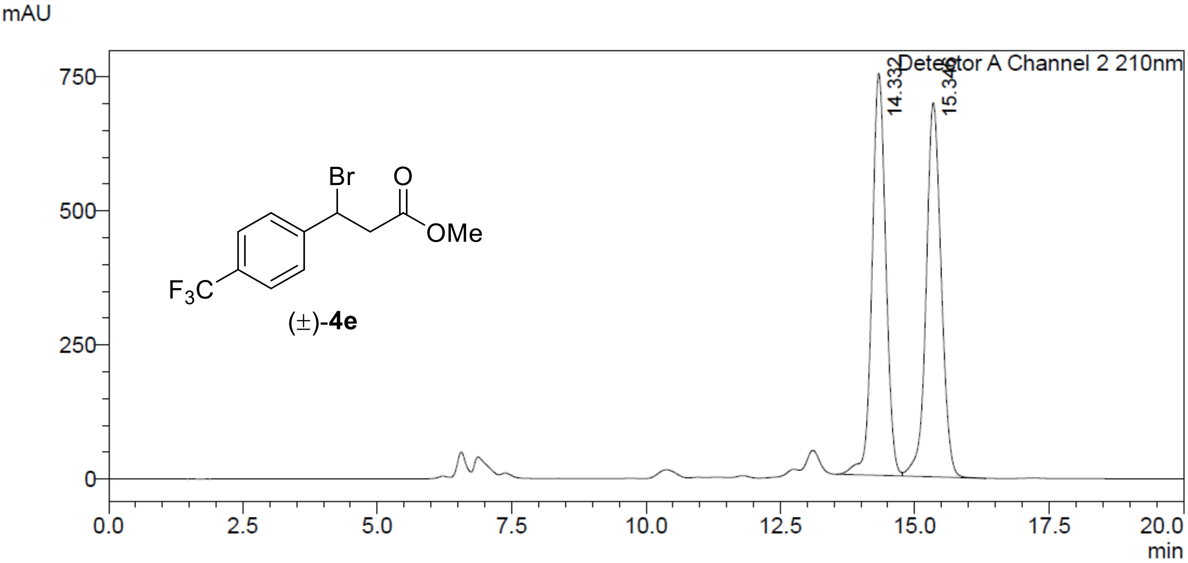

<Peak Table>

Detector A Channel 2 210nm

Peak#	Ret. Time	Area	Height	Conc.
1	14.332	13384115	749288	49.602
2	15.346	13599052	697604	50.398
Total		26983167	1446891	

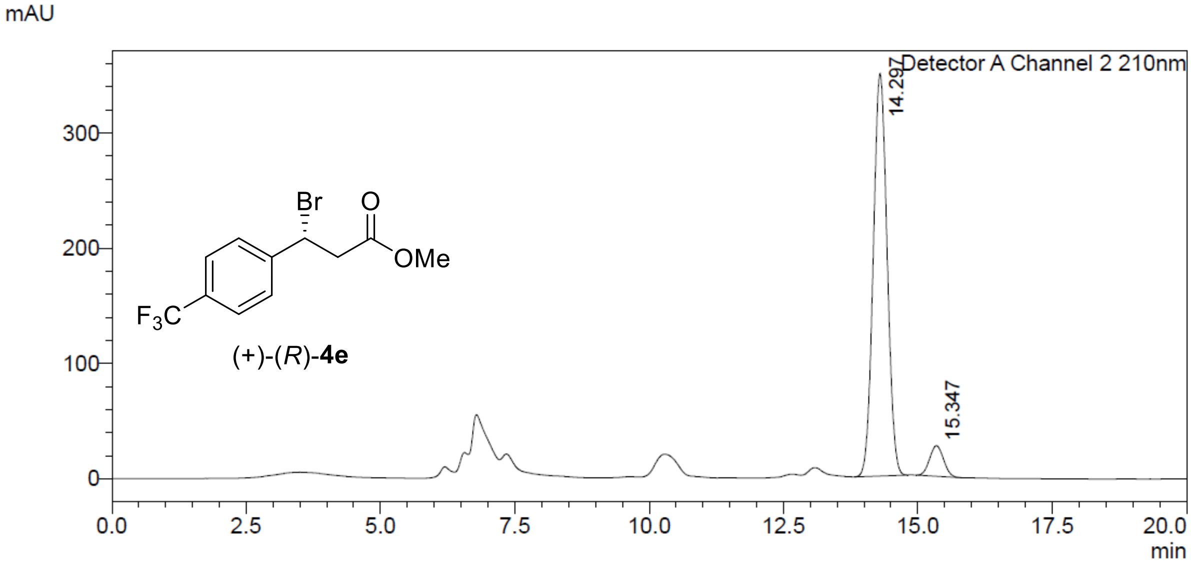

<Peak Table>

Detector A Channel 2 210nm

Peak#	Ret. Time	Area	Height	Conc.
1	14.297	6260040	349040	92.918
2	15.347	477103	26438	7.082
Total		6737143	375478	

12.9 HPLC traces of (±)-5 and (–)-(*S*)-5

CHIRALPAK IB-H, *n*-hexane/*i*-PrOH, 90:10, 0.75 mL/min, 210 nm

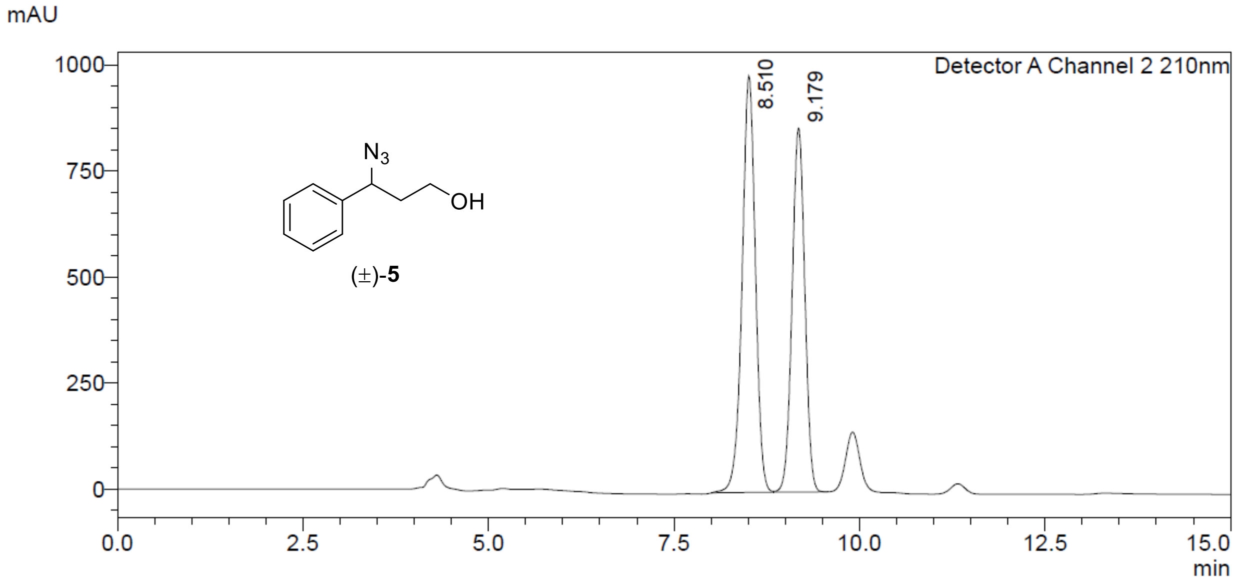

<Peak Table>

Detector A Channel 2 210nm

Peak#	Ret. Time	Area	Height	Conc.
1	8.510	12246093	981130	0.000
2	9.179	10237590	857978	0.000
Total		22483682	1839108	

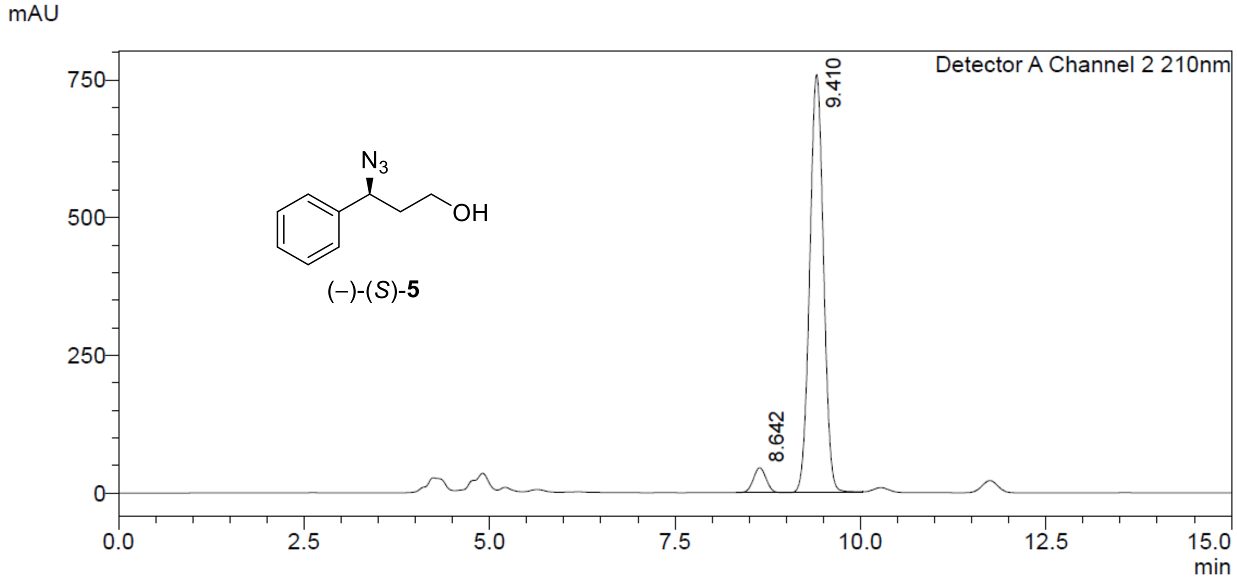

<Peak Table>

Detector A Channel 2 210nm

Peak#	Ret. Time	Area	Height	Conc.
1	8.642	546143	45350	0.000
2	9.410	9861414	758303	0.000
Total		10407557	803653	

12.10 HPLC traces of (±)-4t and (*R*)-4t

CHIRALPAK IB-H, *n*-hexane/*i*-PrOH, 90:10, 0.75 mL/min, 210 nm

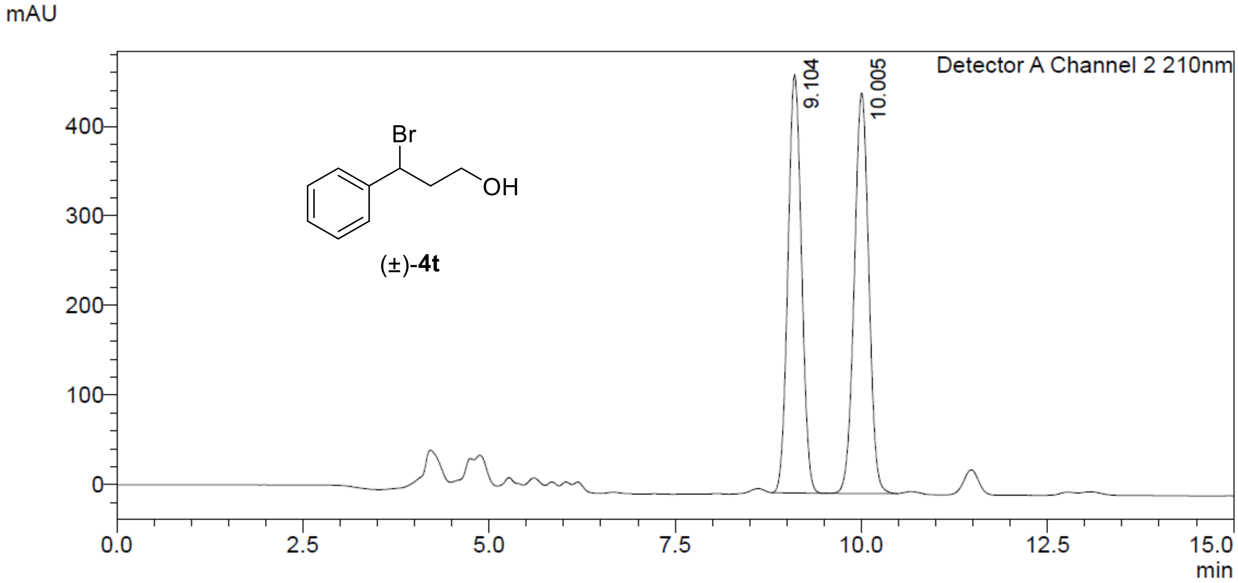

<Peak Table>

Detector A Channel 2 210nm

Peak#	Ret. Time	Area	Height	Conc.
1	9.104	5939848	466259	0.000
2	10.005	6077480	446977	0.000
Total		12017328	913236	

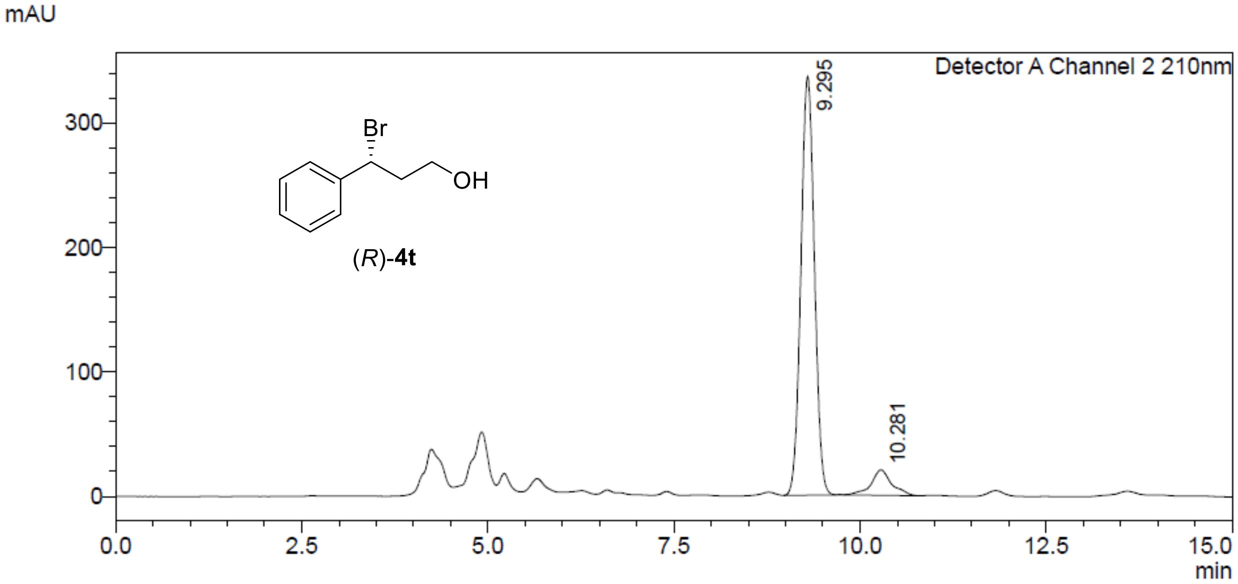

<Peak Table>

Detector A Channel 2 210nm

Peak#	Ret. Time	Area	Height	Conc.
1	9.295	4249275	336103	91.436
2	10.281	398004	20510	8.564
Total		4647279	356613	

13. Copies of ^{1}H NMR and ^{13}C NMR spectra

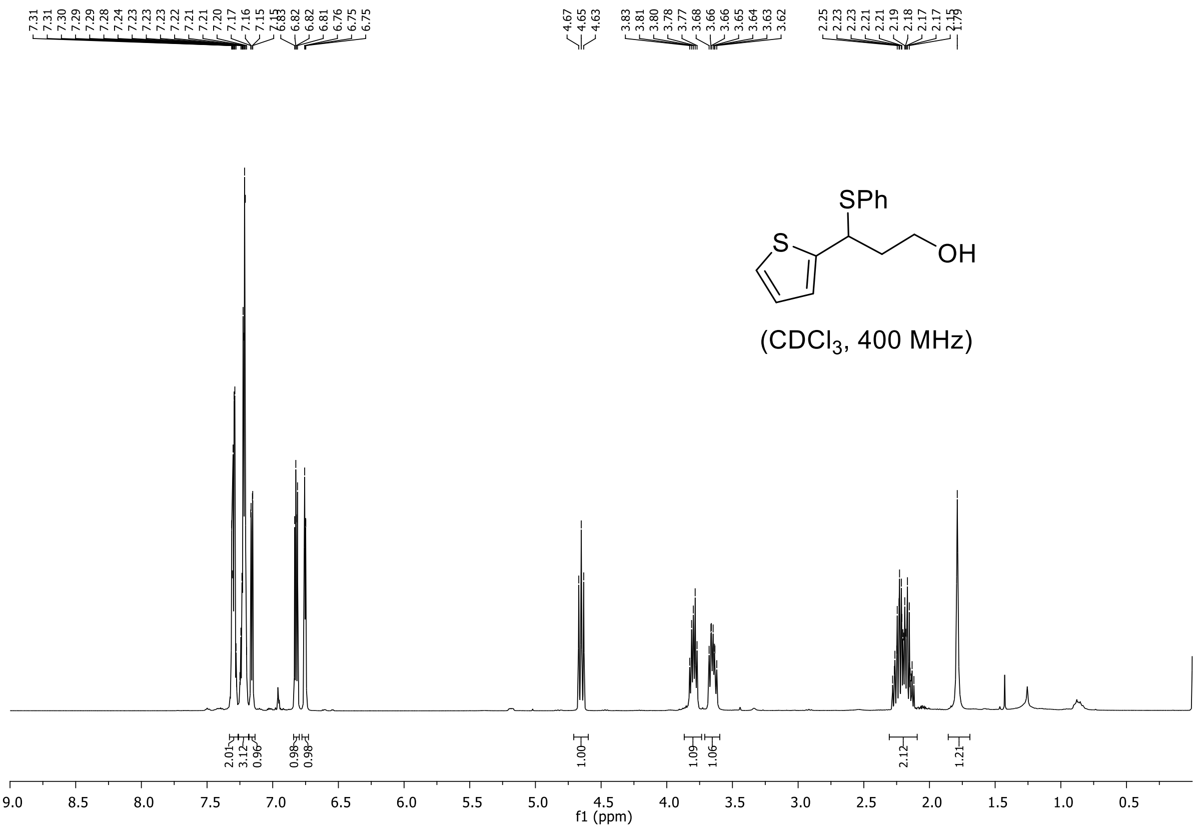

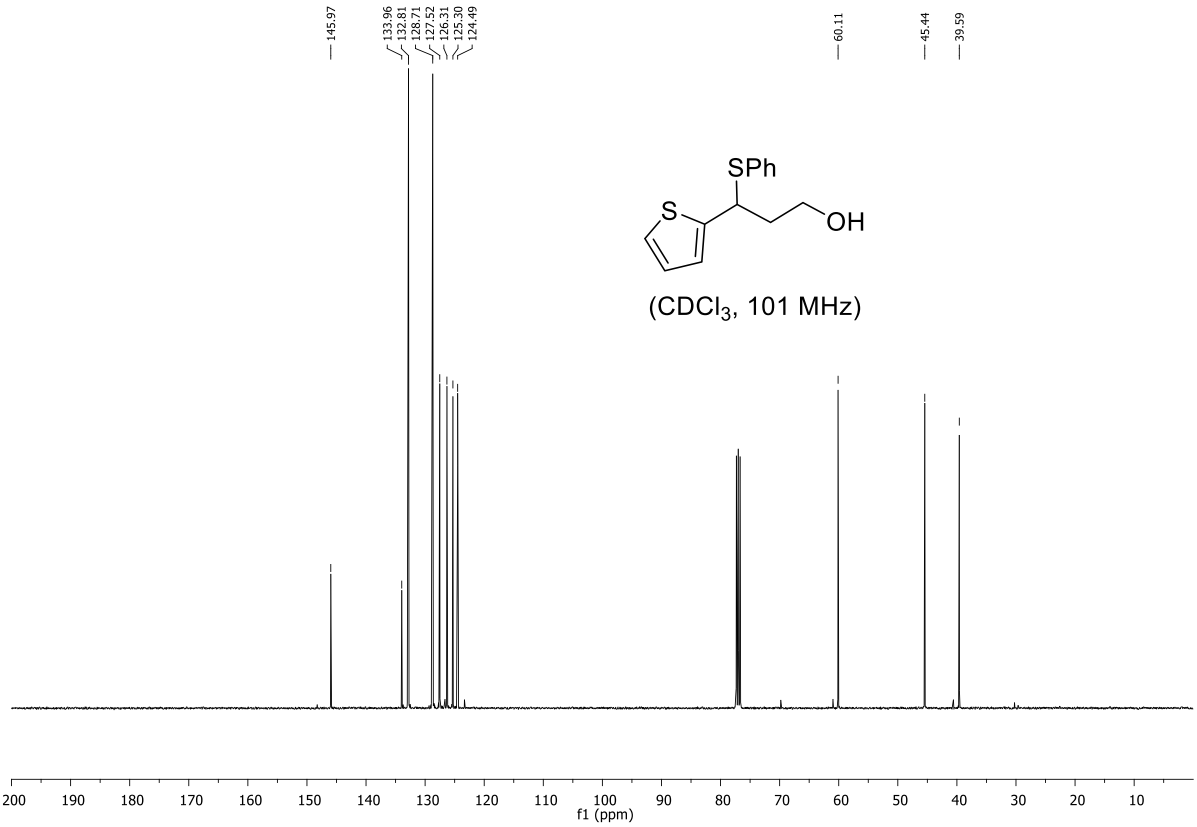

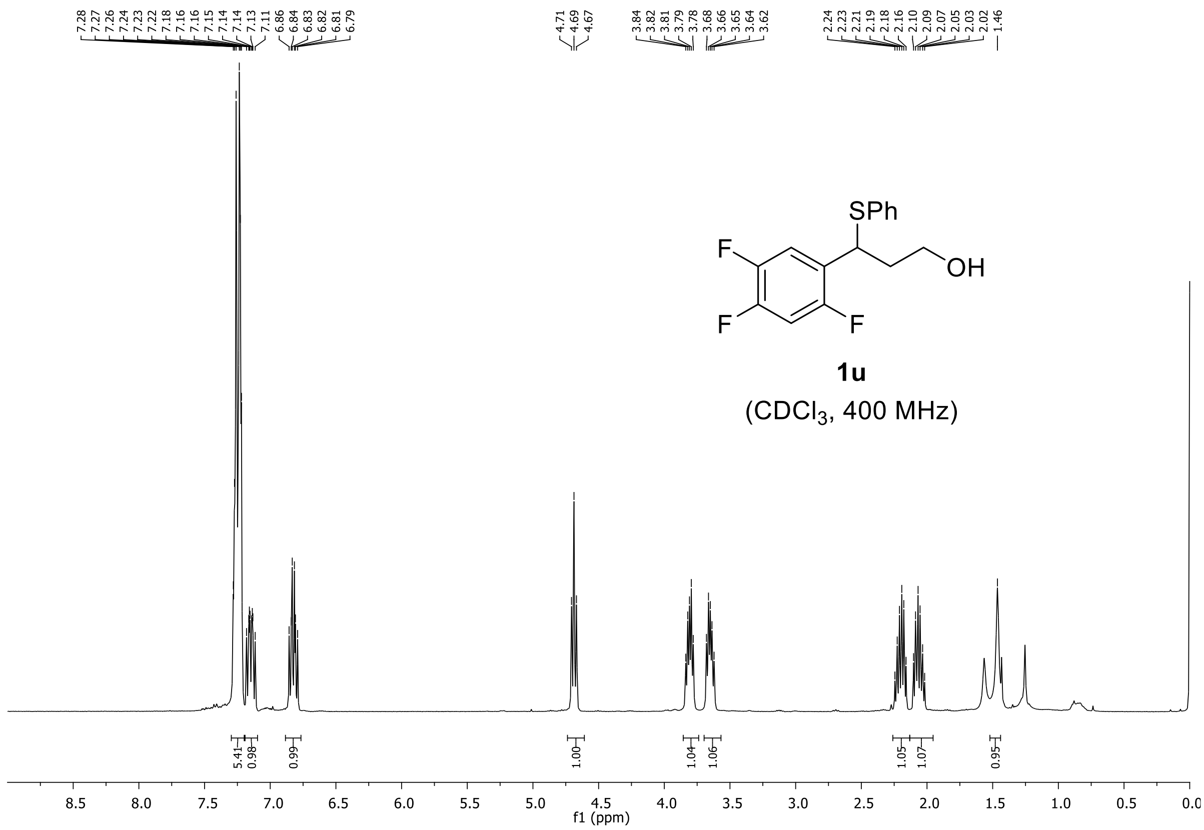
SPh
F
OH
F
F
1u
(CDCl3, 400 MHz)
f1 (ppm)

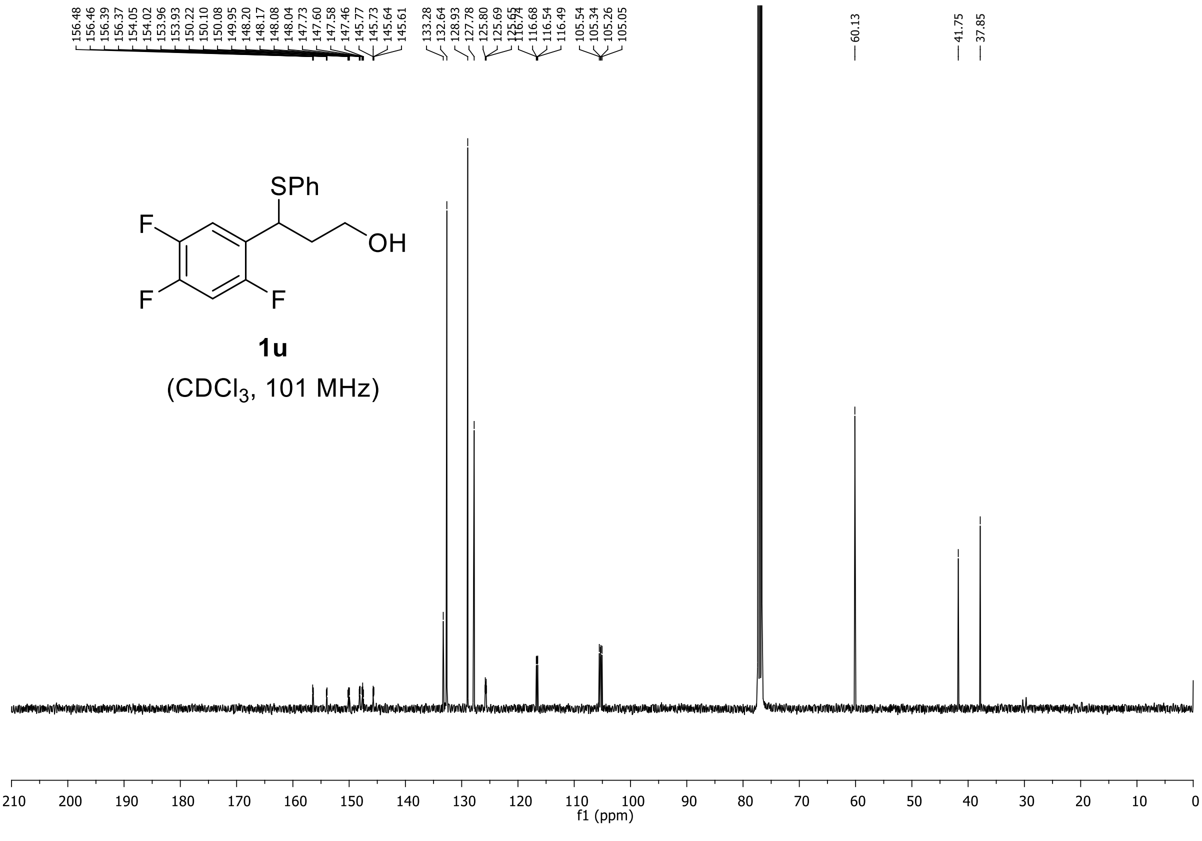
SPh
F
OH
F
F
1u
(CDCl3, 101 MHz)
f1 (ppm)

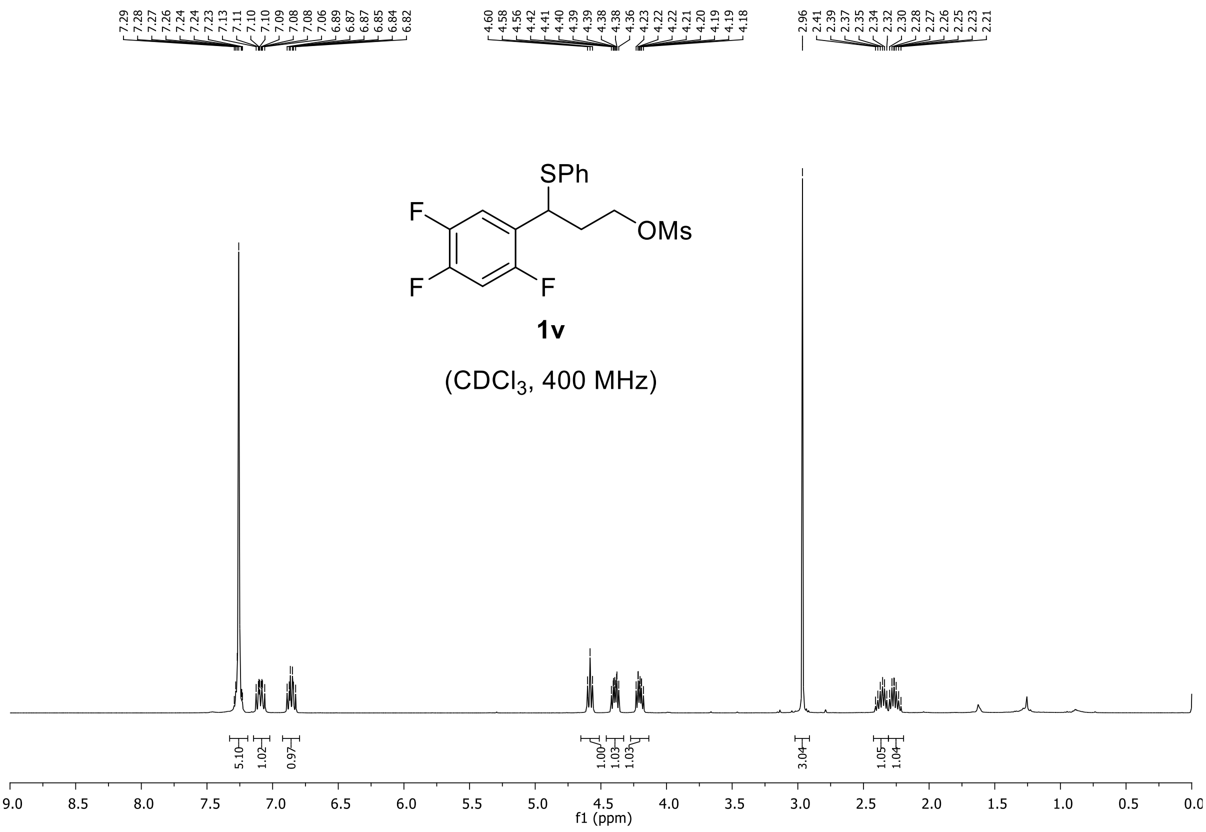
7.29 7.28 7.27 7.26 7.24 7.24 7.23 7.13 7.11 7.10 7.10 7.09 7.08 7.08 7.06 6.89 6.87 6.87 6.85 6.84 6.82
4.60 4.58 4.56 4.42 4.41 4.40 4.39 4.39 4.38 4.38 4.36 4.23 4.22 4.22 4.21 4.20 4.19 4.19 4.18
2.96 2.41 2.39 2.37 2.35 2.34 2.32 2.30 2.28 2.27 2.26 2.25 2.23 2.21
SPh
F
OMs
F
F
1v
(CDCl3, 400 MHz)
5.10 1.02 0.97 1.00 1.03 1.03 3.04 1.05 1.04
9.0 8.5 8.0 7.5 7.0 6.5 6.0 5.5 5.0 4.5 4.0 3.5 3.0 2.5 2.0 1.5 1.0 0.5 0.0
f1 (ppm)

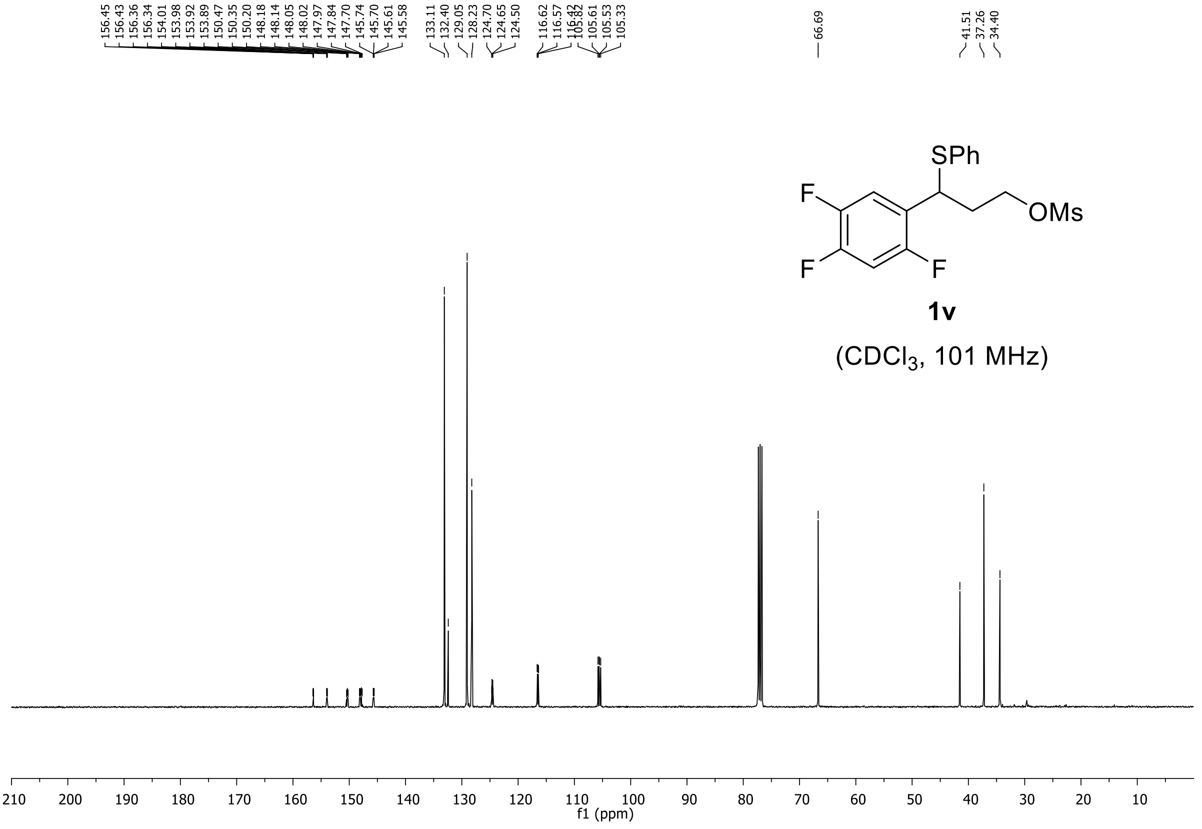
156.45 156.43 156.36 156.34 154.01 153.98 153.92 153.89 150.47 150.35 150.20 148.18 148.14 148.05 148.02 147.97 147.84 147.70 145.74 145.70 145.61 145.58
133.11 132.40 129.05 128.23 124.70 124.65 124.50
116.62 116.57 116.42 105.82 105.61 105.53 105.33
66.69
41.51 37.26 34.40
SPh
F
OMs
F
F
1v
(CDCl3, 101 MHz)
210 200 190 180 170 160 150 140 130 120 110 100 90 80 70 60 50 40 30 20 10
f1 (ppm)

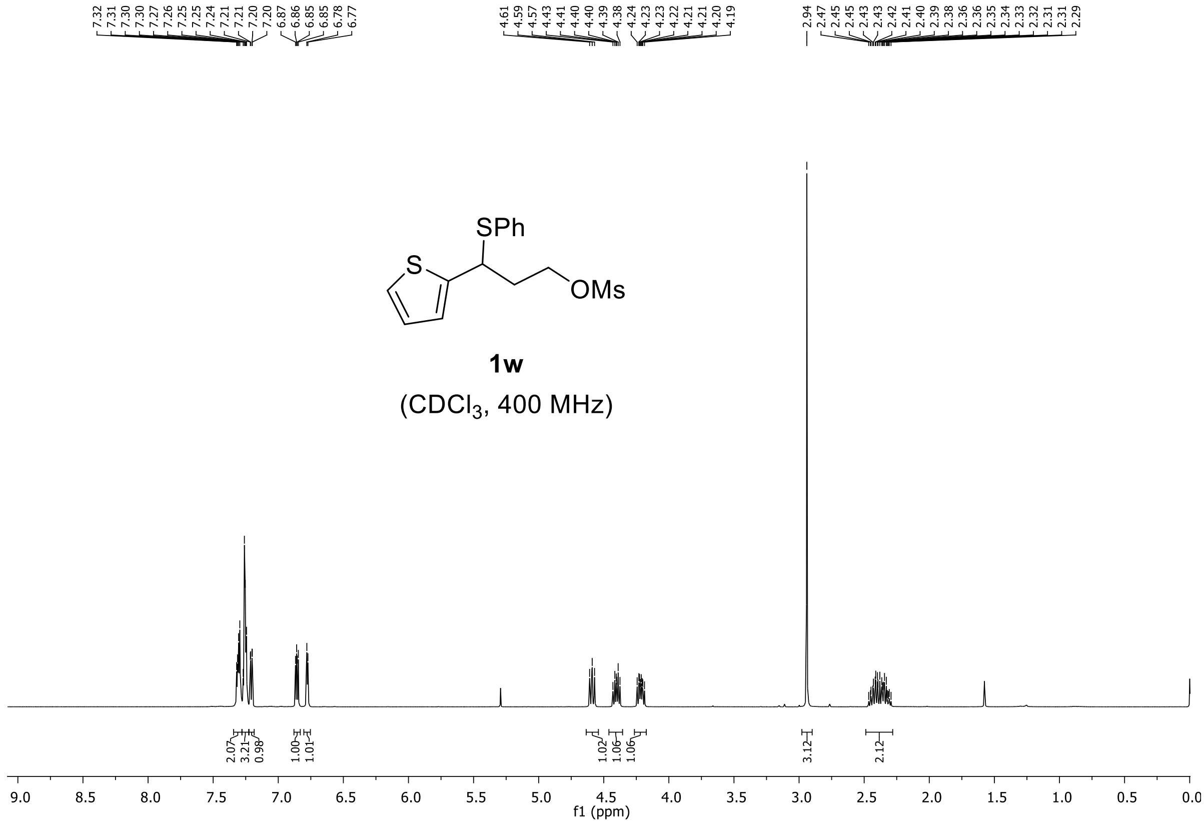
7.32 7.31 7.30 7.30 7.27 7.26 7.25 7.25 7.24 7.21 7.21 7.20 7.20 6.87 6.86 6.85 6.85 6.78 6.77
4.61 4.59 4.57 4.43 4.41 4.40 4.40 4.39 4.38 4.24 4.23 4.23 4.22 4.21 4.21 4.20 4.19
2.94 2.47 2.45 2.45 2.43 2.43 2.42 2.41 2.40 2.39 2.38 2.36 2.36 2.35 2.34 2.33 2.32 2.31 2.29
SPh
S
OMs
1w
(CDCl3, 400 MHz)
2.07 3.21 0.98 1.00 1.01 1.02 1.06 1.06 3.12 2.12
9.0 8.5 8.0 7.5 7.0 6.5 6.0 5.5 5.0 4.5 4.0 3.5 3.0 2.5 2.0 1.5 1.0 0.5 0.0
f1 (ppm)

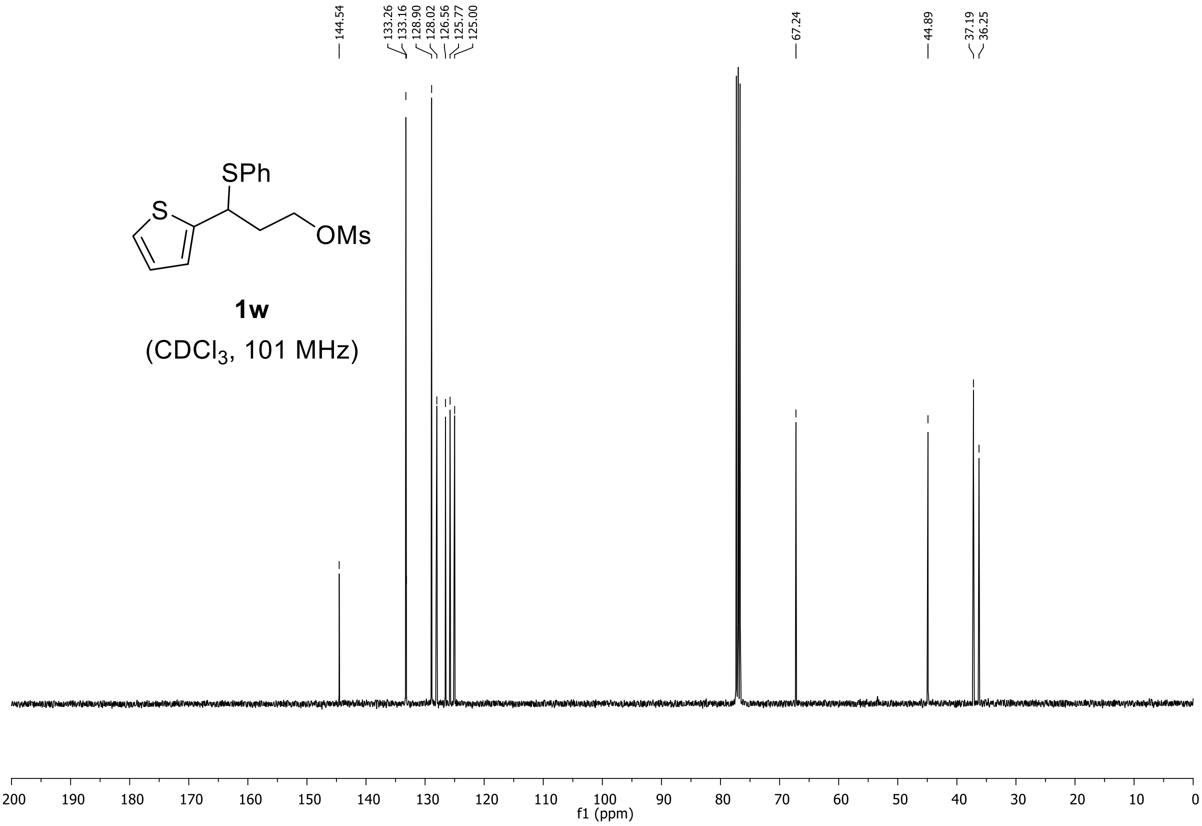
144.54
133.26 133.16 128.90 128.02 126.56 125.77 125.00
67.24
44.89
37.19 36.25
SPh
S
OMs
1w
(CDCl3, 101 MHz)
200 190 180 170 160 150 140 130 120 110 100 90 80 70 60 50 40 30 20 10 0
f1 (ppm)

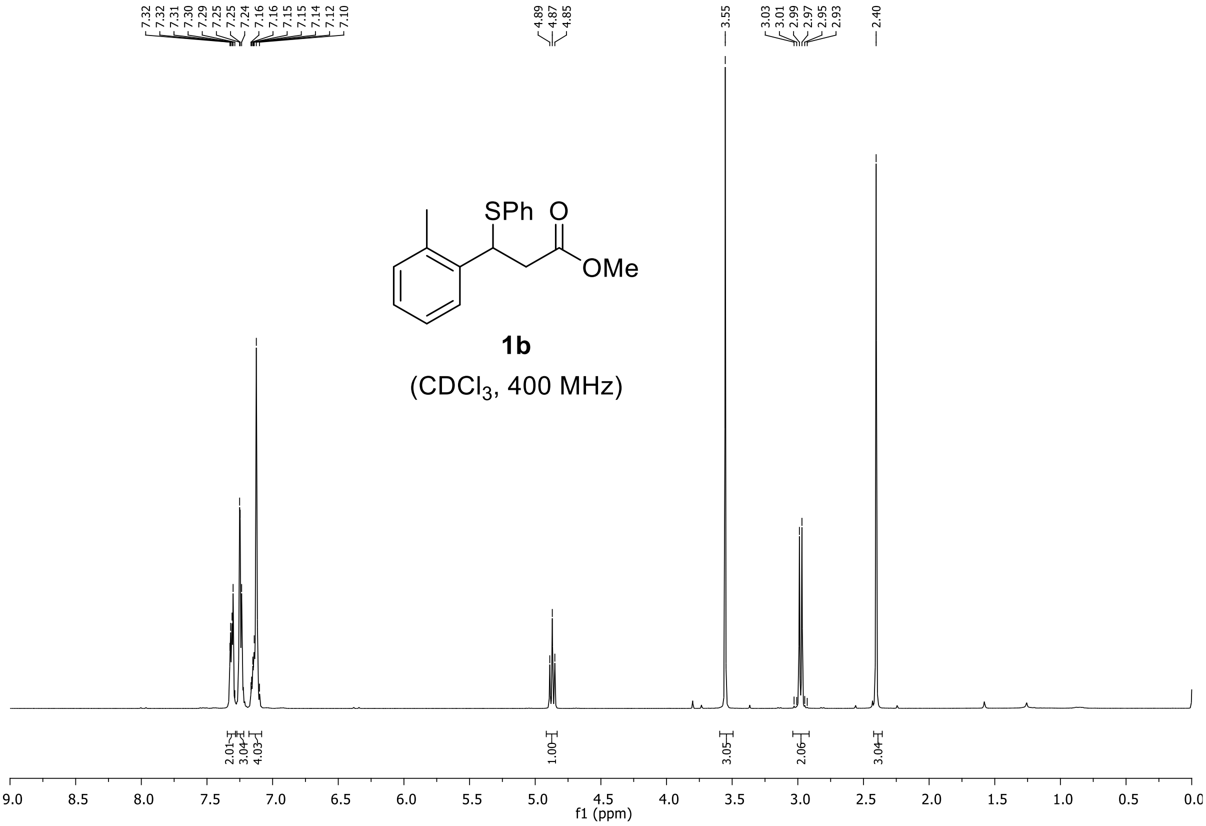
7.32 7.32 7.31 7.30 7.29 7.25 7.25 7.24 7.16 7.16 7.15 7.15 7.14 7.12 7.10
4.89 4.87 4.85
3.55
3.03 3.01 2.99 2.97 2.95 2.93
2.40
SPh
O
OMe
1b
(CDCl3, 400 MHz)
2.01 3.04 4.03 1.00 3.05 2.06 3.04
f1 (ppm)

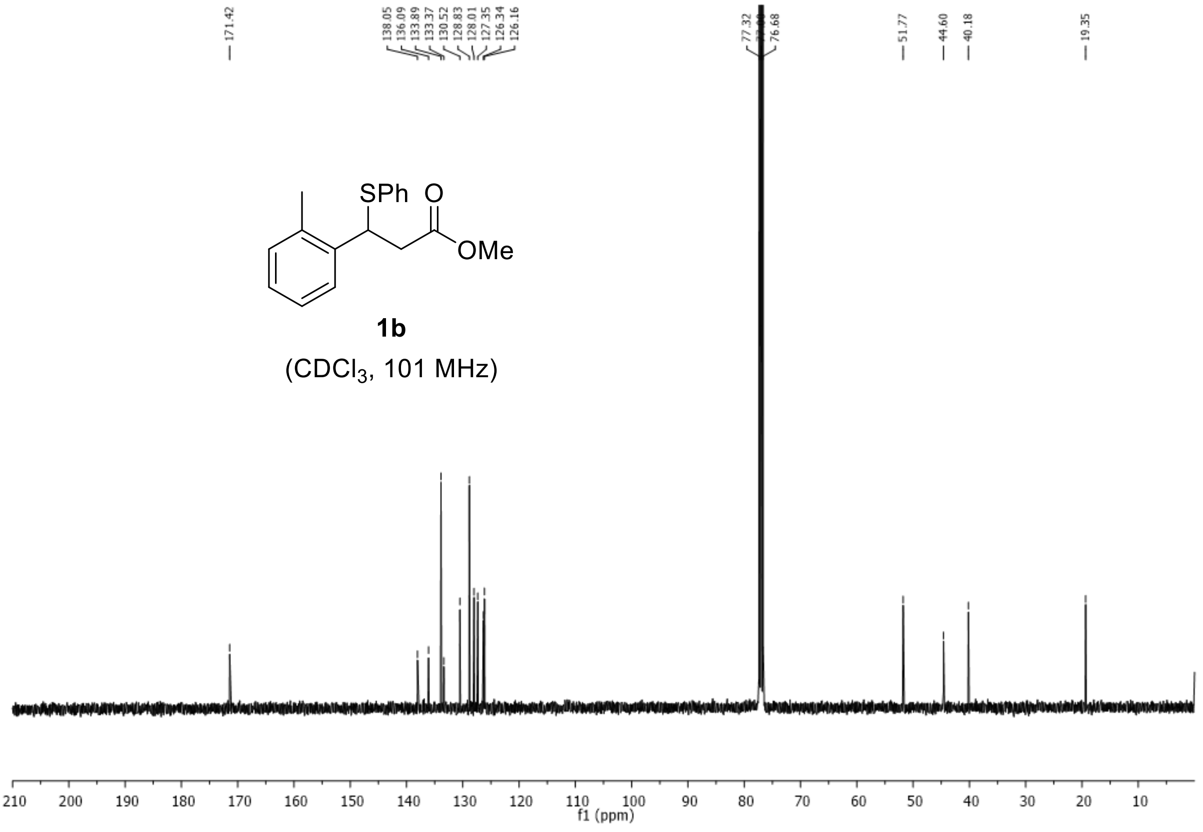
171.42
138.05 136.09 133.89 133.37 130.52 128.83 128.01 127.35 126.34 126.16
77.32 76.68
51.77
44.60
40.18
19.35
SPh
O
OMe
1b
(CDCl3, 101 MHz)
f1 (ppm)

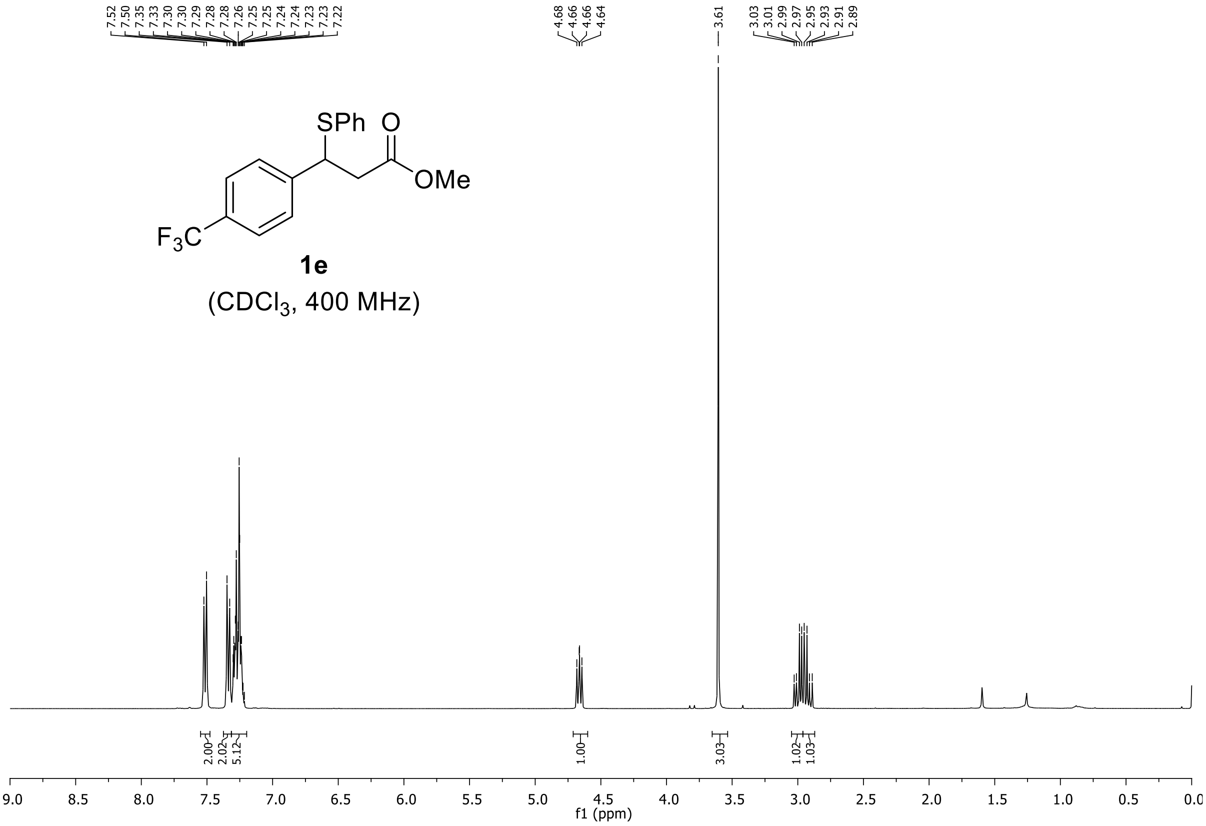
7.52 7.50 7.35 7.33 7.30 7.30 7.29 7.28 7.28 7.26 7.25 7.25 7.24 7.24 7.23 7.23 7.22
4.68 4.66 4.66 4.64
3.61
3.03 3.01 2.99 2.97 2.95 2.93 2.91 2.89
SPh
O
OMe
F3C
1e
(CDCl3, 400 MHz)
2.00 2.02 5.12
1.00
3.03
1.02 1.03
f1 (ppm)

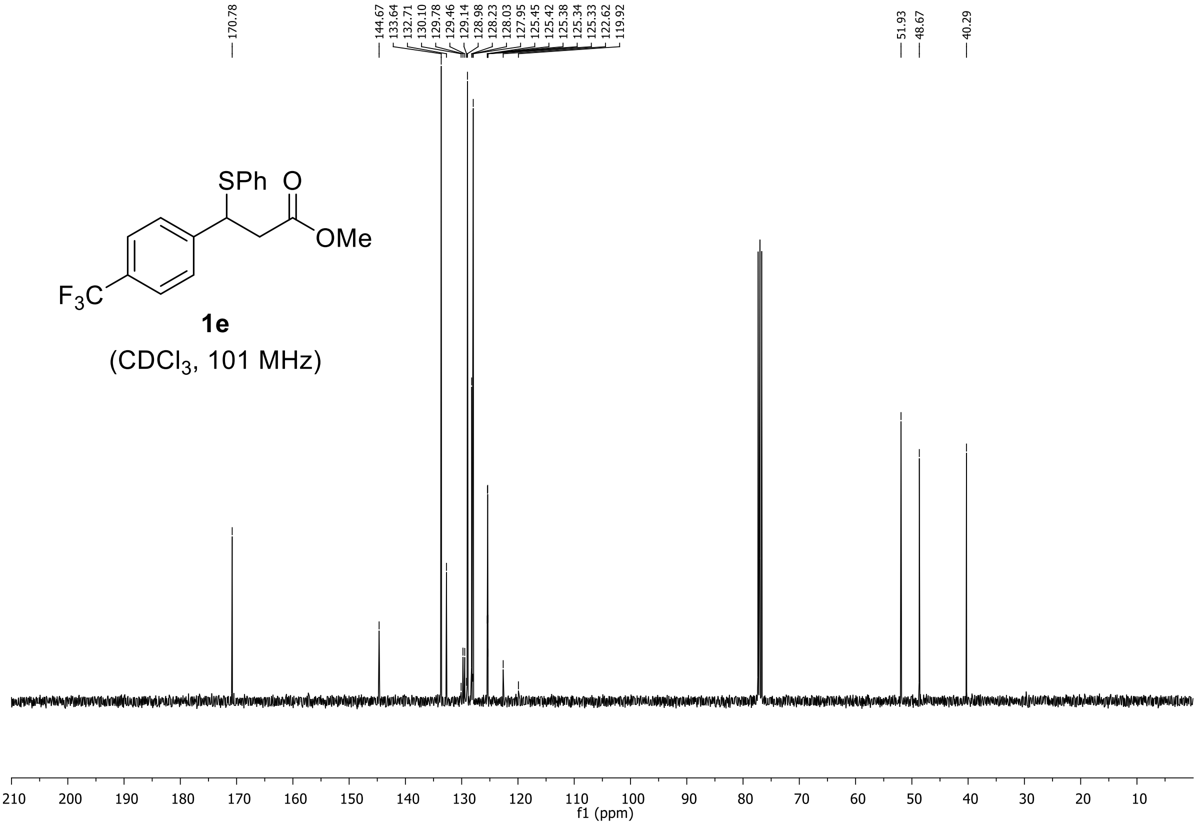
170.78
144.67 133.64 132.71 130.10 129.78 129.46 129.14 128.98 128.23 128.03 127.95 125.45 125.42 125.38 125.34 125.33 122.62 119.92
51.93 48.67
40.29
SPh
O
OMe
F3C
1e
(CDCl3, 101 MHz)
f1 (ppm)

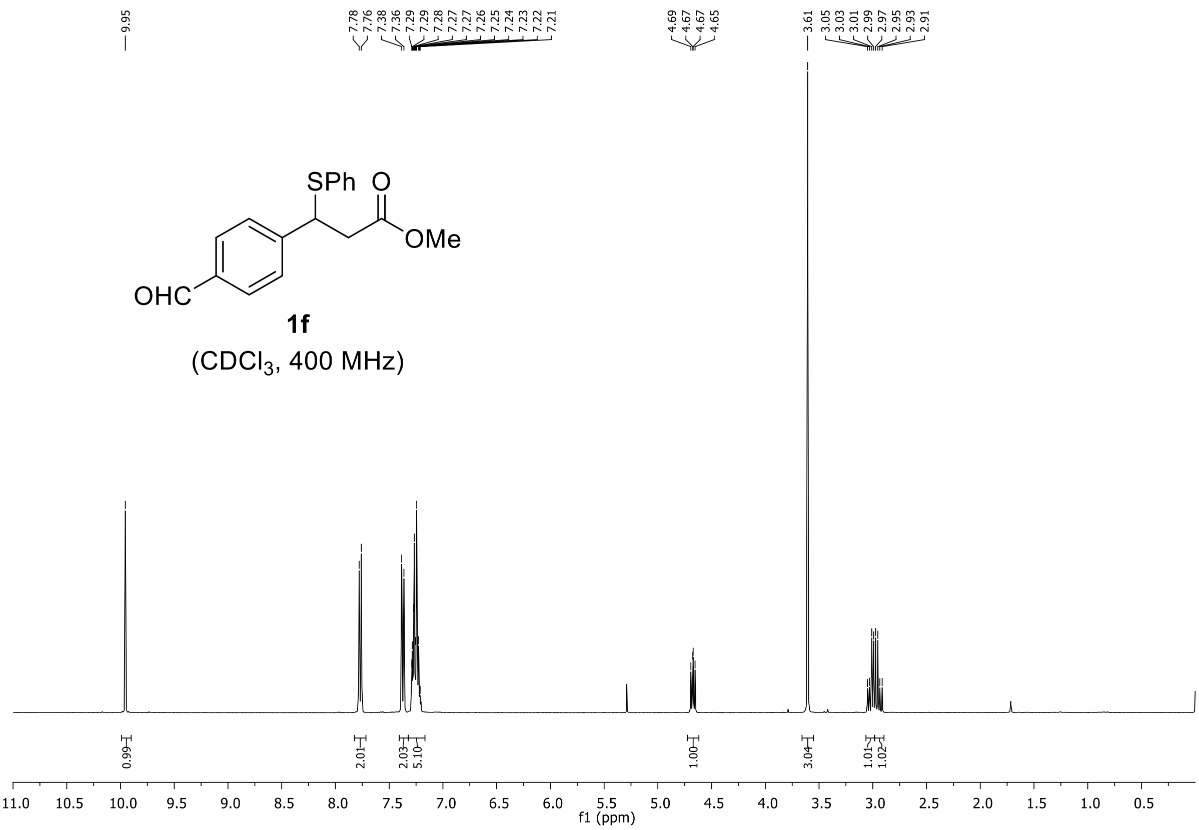
9.95
7.78
7.76
7.38
7.36
7.29
7.29
7.28
7.27
7.27
7.26
7.25
7.24
7.23
7.22
7.21
4.69
4.67
4.67
4.65
3.61
3.05
3.03
3.01
2.99
2.97
2.95
2.93
2.91
SPh
O
OMe
OHC
1f
(CDCl3, 400 MHz)
0.99
2.01
2.03
5.10
1.00
3.04
1.01
1.02
f1 (ppm)

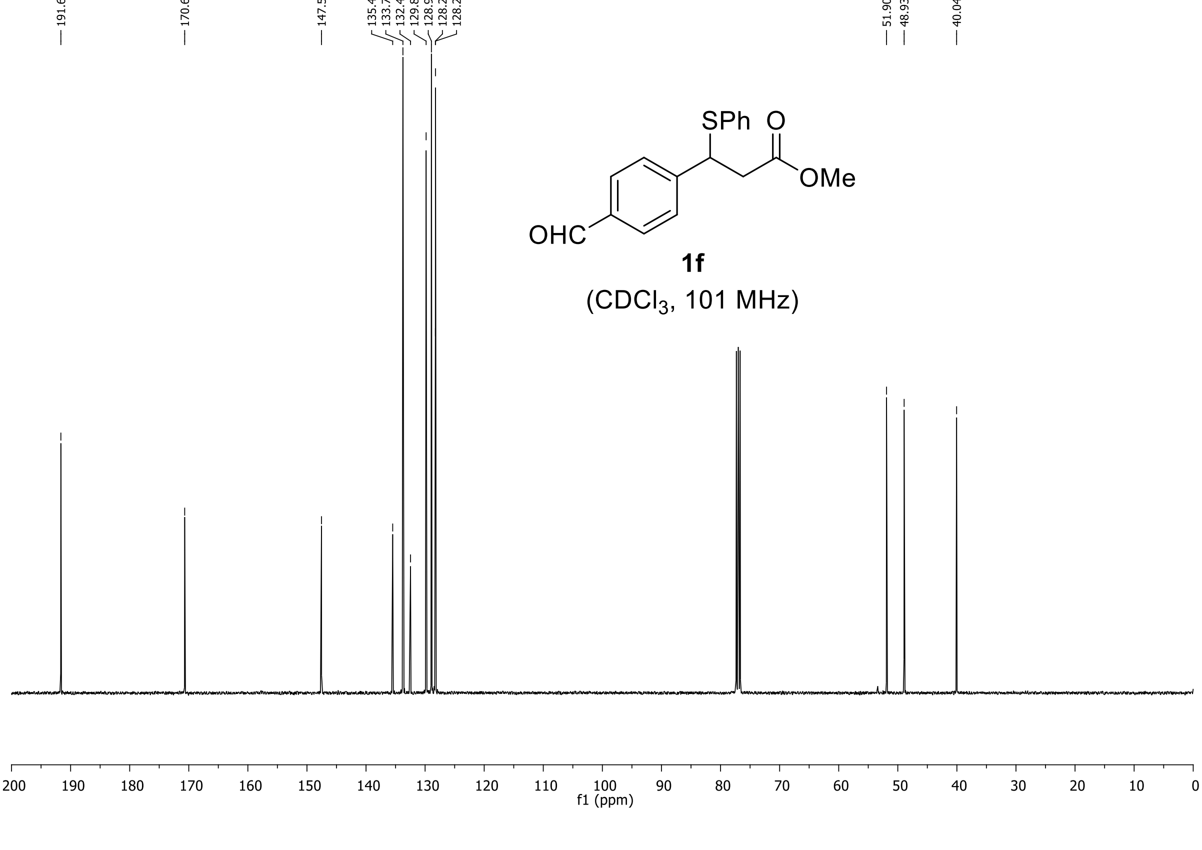
191.65
170.69
147.54
135.49
133.76
132.47
129.83
128.93
128.26
128.24
51.90
48.93
40.04
SPh
O
OMe
OHC
1f
(CDCl3, 101 MHz)
f1 (ppm)

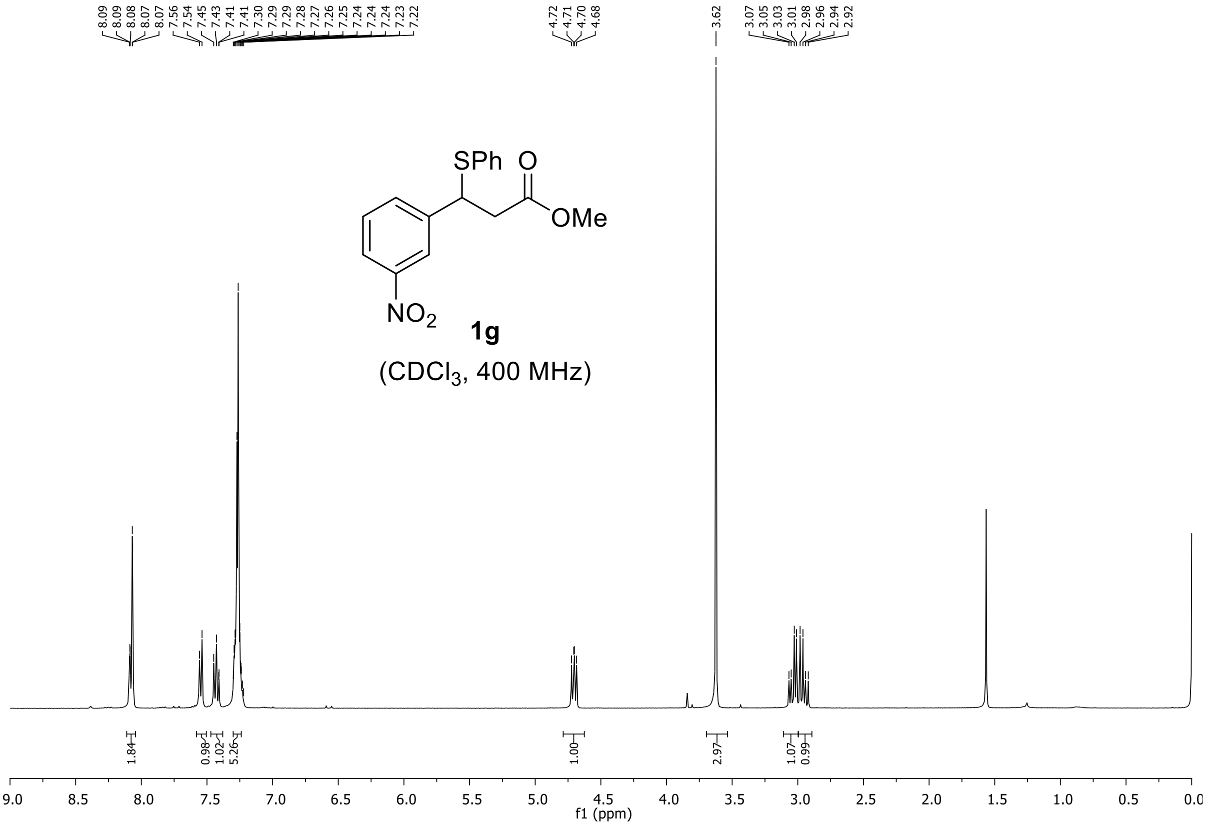
SPh
O
OMe
NO2
1g
(CDCl3, 400 MHz)
f1 (ppm)

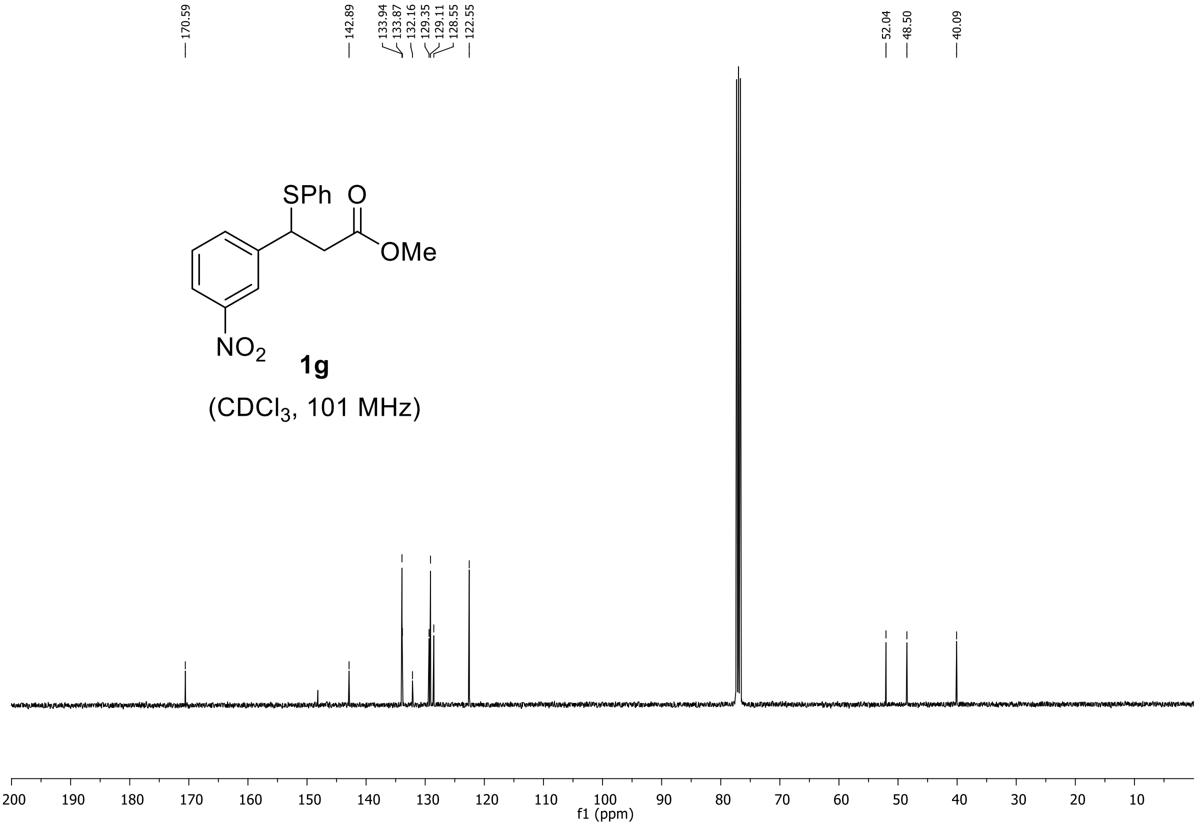
SPh
O
OMe
NO2
1g
(CDCl3, 101 MHz)
f1 (ppm)

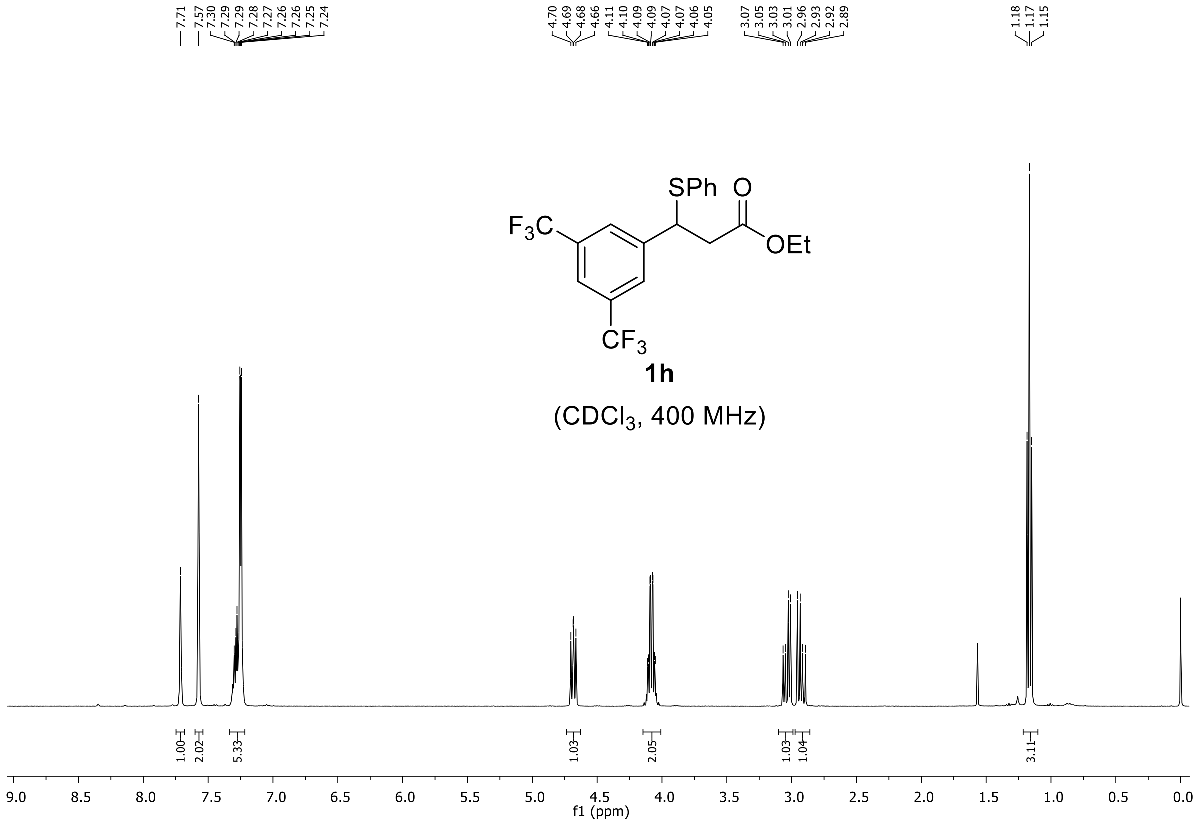
SPh O
F3C
OEt
CF3
1h
(CDCl3, 400 MHz)
f1 (ppm)

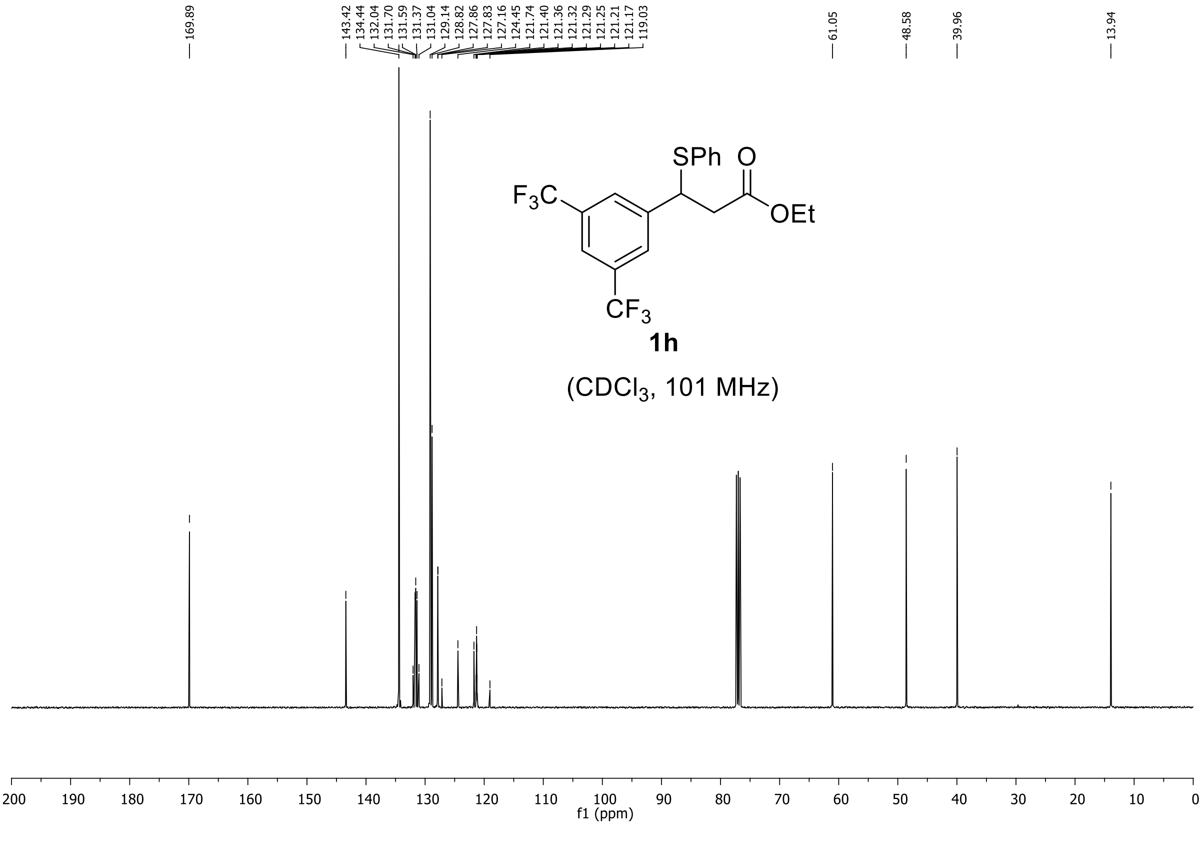
SPh O
F3C
OEt
CF3
1h
(CDCl3, 101 MHz)
f1 (ppm)

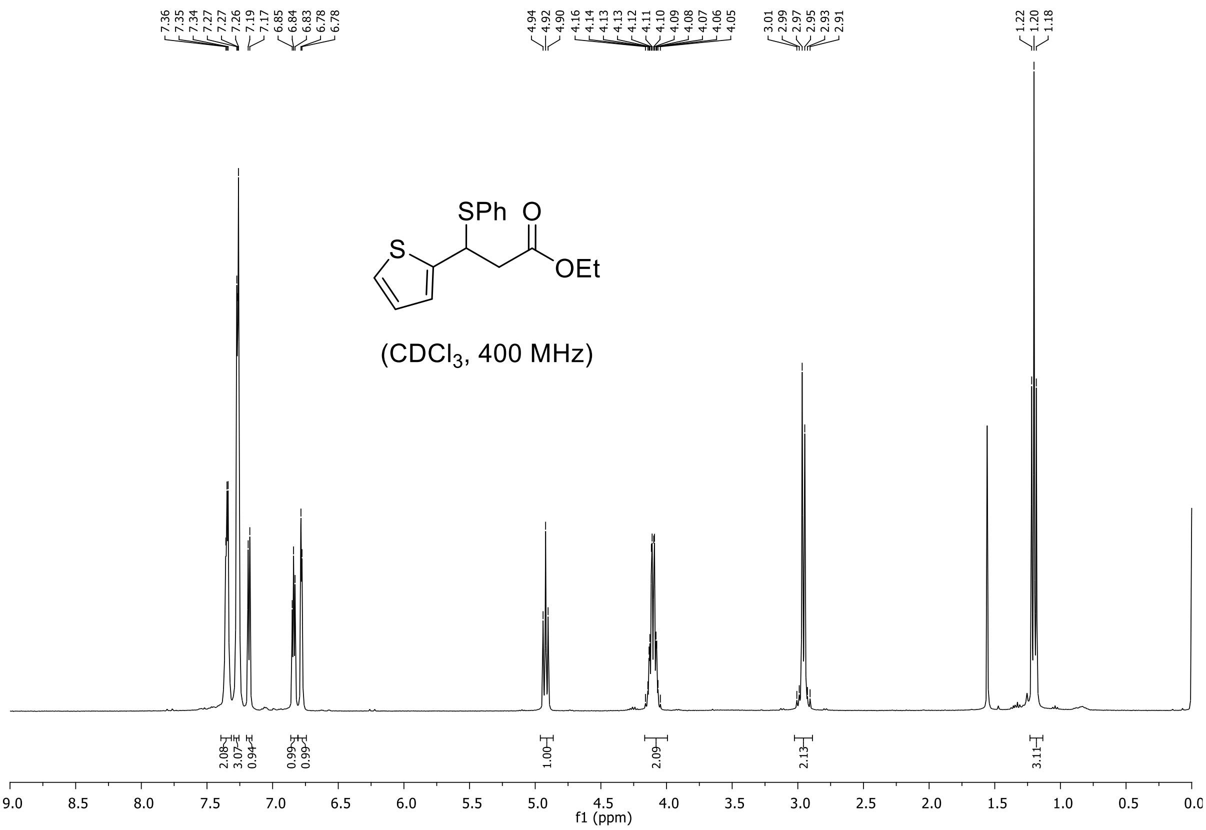
SPh O
S
OEt
(CDCl3, 400 MHz)
f1 (ppm)

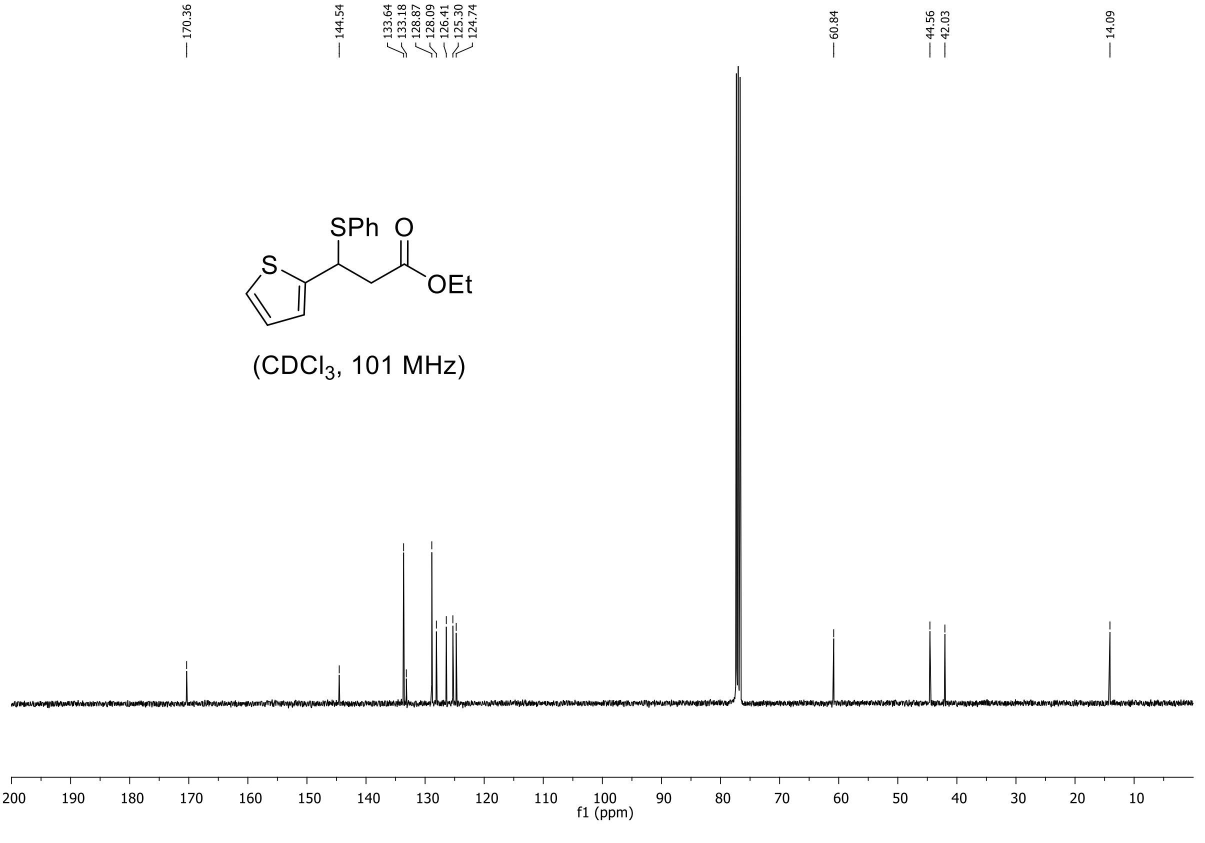
170.36
144.54
133.64
133.18
128.87
128.09
126.41
125.30
124.74
60.84
44.56
42.03
14.09
SPh O
S
OEt
(CDCl3, 101 MHz)
f1 (ppm)

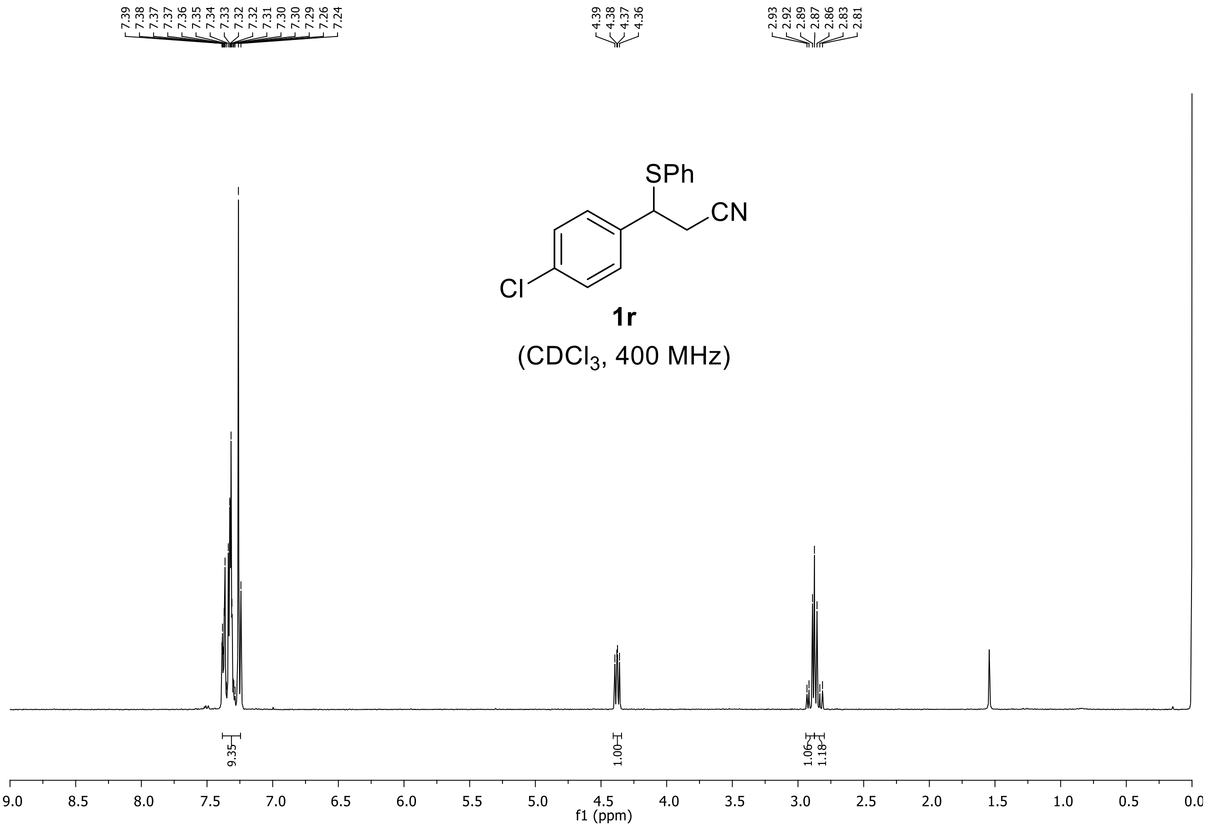
SPh
CN
Cl
1r
(CDCl3, 400 MHz)
f1 (ppm)

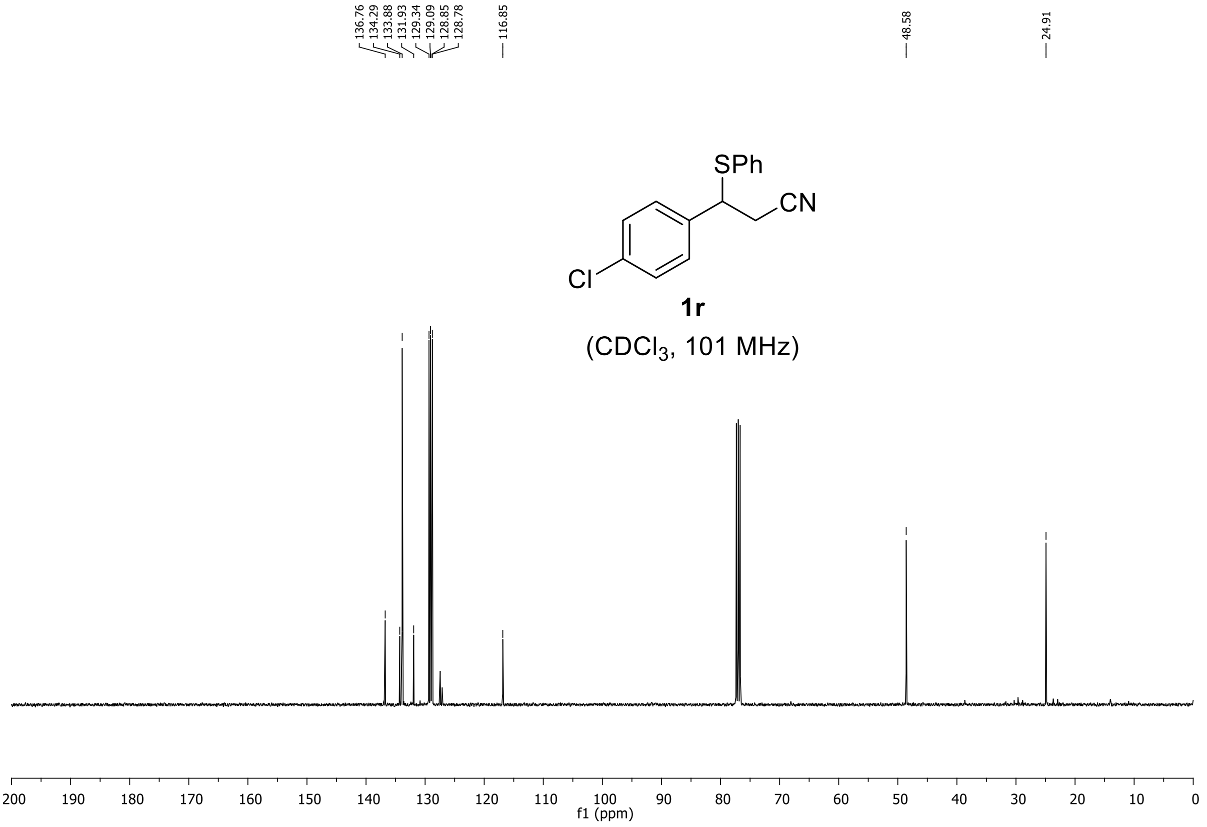
SPh
CN
Cl
1r
(CDCl3, 101 MHz)
f1 (ppm)

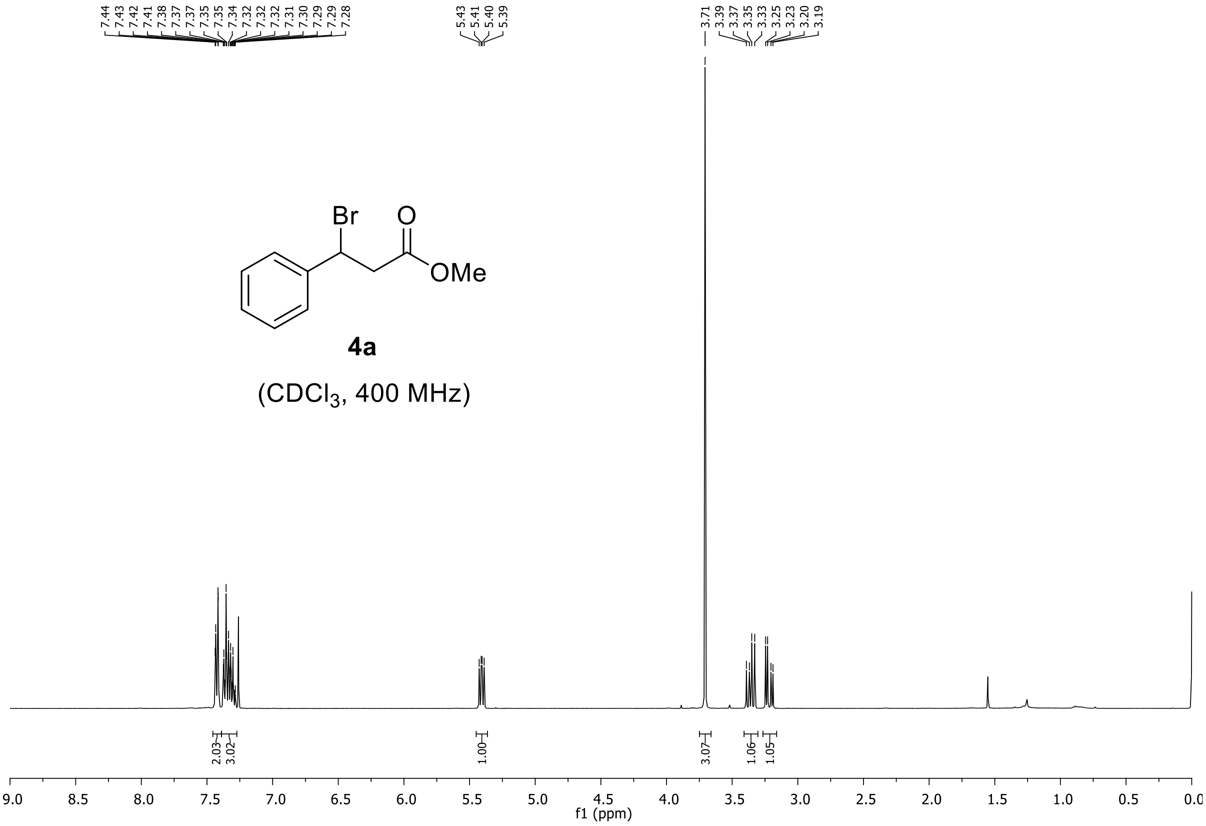
7.44 7.43 7.42 7.41 7.38 7.37 7.37 7.35 7.35 7.34 7.32 7.32 7.32 7.31 7.30 7.29 7.29 7.28
5.43 5.41 5.40 5.39
3.71 3.39 3.37 3.35 3.33 3.25 3.23 3.20 3.19
Br
O
OMe
4a
(CDCl3, 400 MHz)
2.03 3.02 1.00 3.07 1.06 1.05
9.0 8.5 8.0 7.5 7.0 6.5 6.0 5.5 5.0 4.5 4.0 3.5 3.0 2.5 2.0 1.5 1.0 0.5 0.0
f1 (ppm)

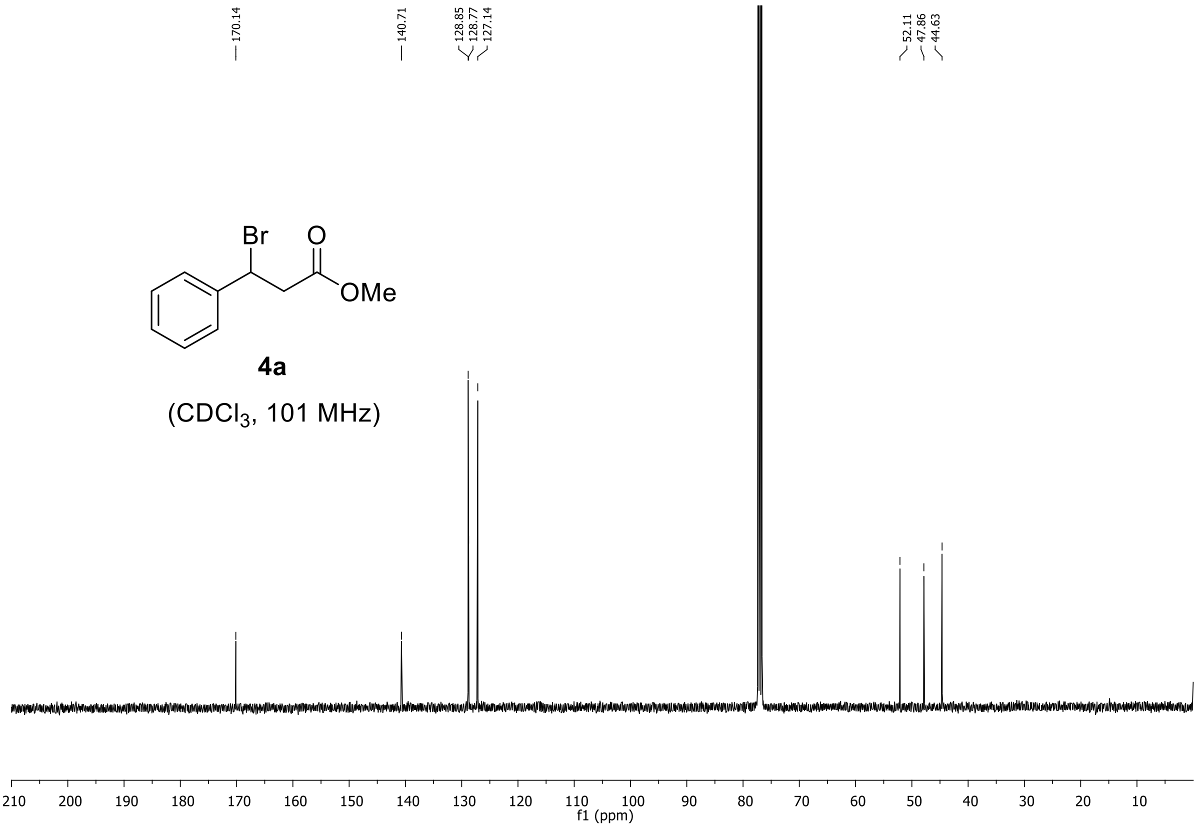
170.14
140.71
128.85 128.77 127.14
52.11 47.86 44.63
Br
O
OMe
4a
(CDCl3, 101 MHz)
210 200 190 180 170 160 150 140 130 120 110 100 90 80 70 60 50 40 30 20 10
f1 (ppm)

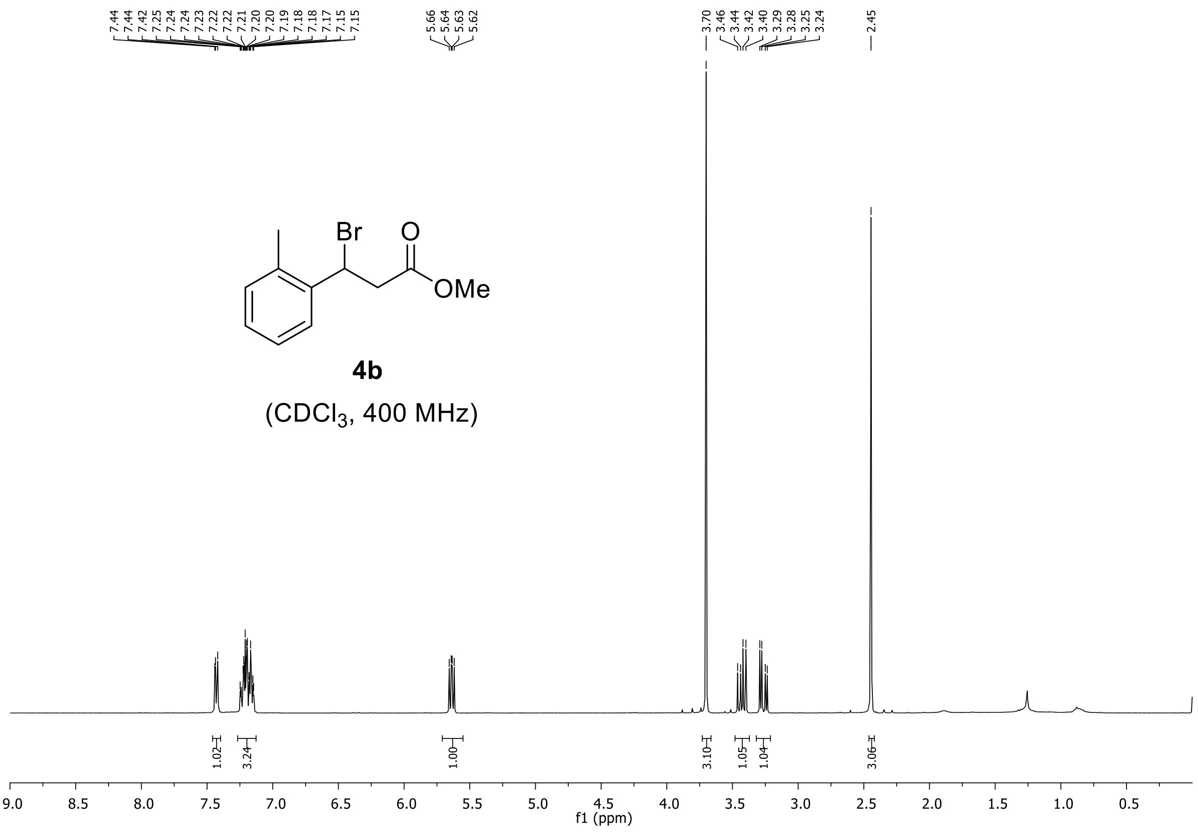
7.44 7.44 7.42 7.25 7.24 7.24 7.23 7.22 7.22 7.21 7.20 7.20 7.19 7.18 7.18 7.17 7.15 7.15
5.66 5.64 5.63 5.62
3.70 3.46 3.44 3.42 3.40 3.29 3.28 3.25 3.24
2.45
Br
O
OMe
4b
(CDCl3, 400 MHz)
1.02
3.24
1.00
3.10
1.05
1.04
3.06
9.0 8.5 8.0 7.5 7.0 6.5 6.0 5.5 5.0 4.5 4.0 3.5 3.0 2.5 2.0 1.5 1.0 0.5
f1 (ppm)

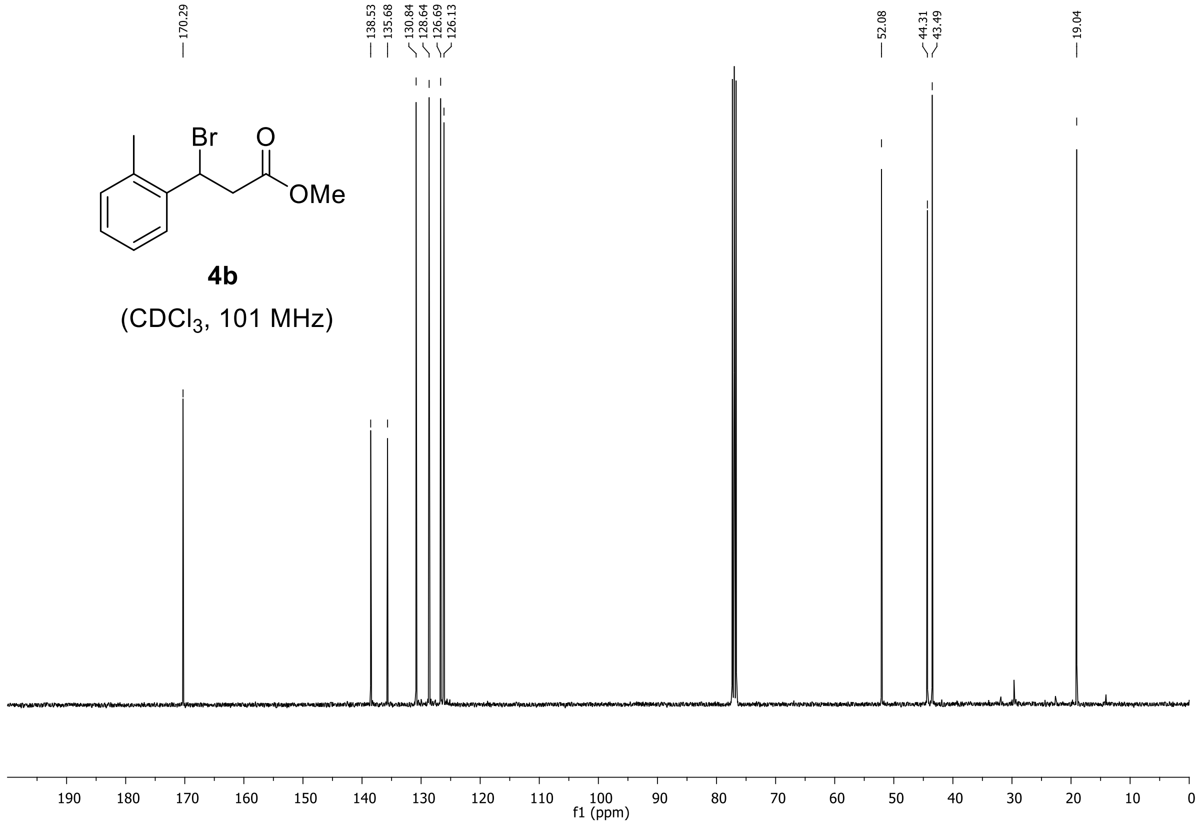
170.29
138.53 135.68 130.84 128.64 126.69 126.13
52.08
44.31 43.49
19.04
Br
O
OMe
4b
(CDCl3, 101 MHz)
190 180 170 160 150 140 130 120 110 100 90 80 70 60 50 40 30 20 10 0
f1 (ppm)

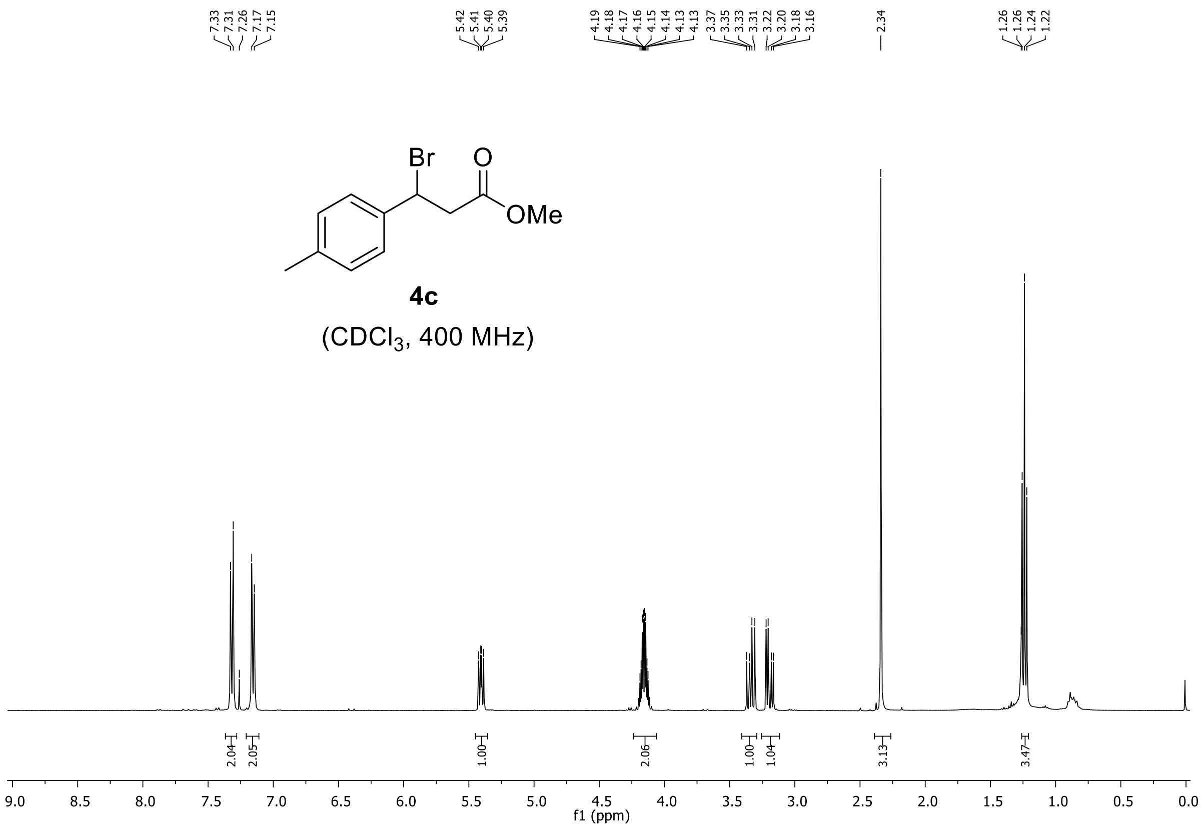
7.33
7.31
7.26
7.17
7.15
5.42
5.41
5.40
5.39
4.19
4.18
4.17
4.16
4.15
4.14
4.13
4.13
3.37
3.35
3.33
3.31
3.22
3.20
3.18
3.16
2.34
1.26
1.26
1.24
1.22
Br
O
OMe
4c
(CDCl3, 400 MHz)
2.04
2.05
1.00
2.06
1.00
1.04
3.13
3.47
f1 (ppm)

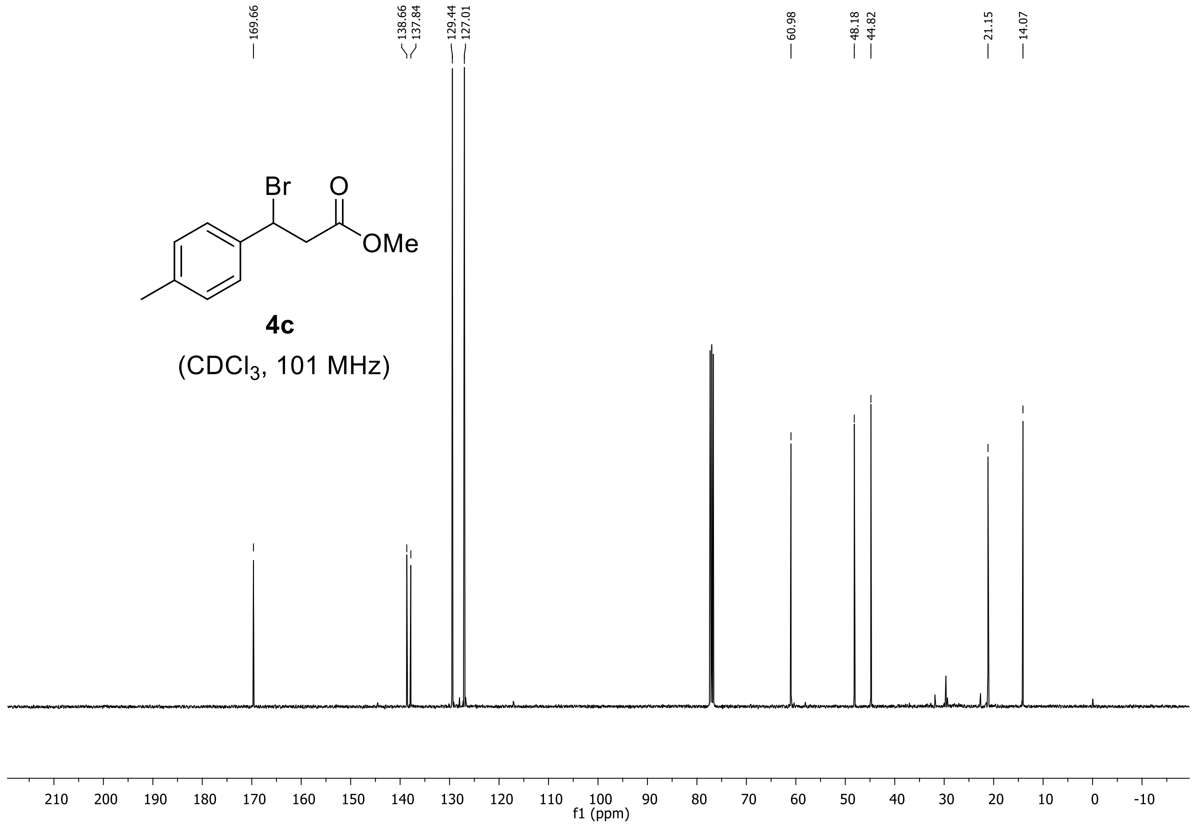
169.66
138.66
137.84
129.44
127.01
60.98
48.18
44.82
21.15
14.07
Br
O
OMe
4c
(CDCl3, 101 MHz)
f1 (ppm)

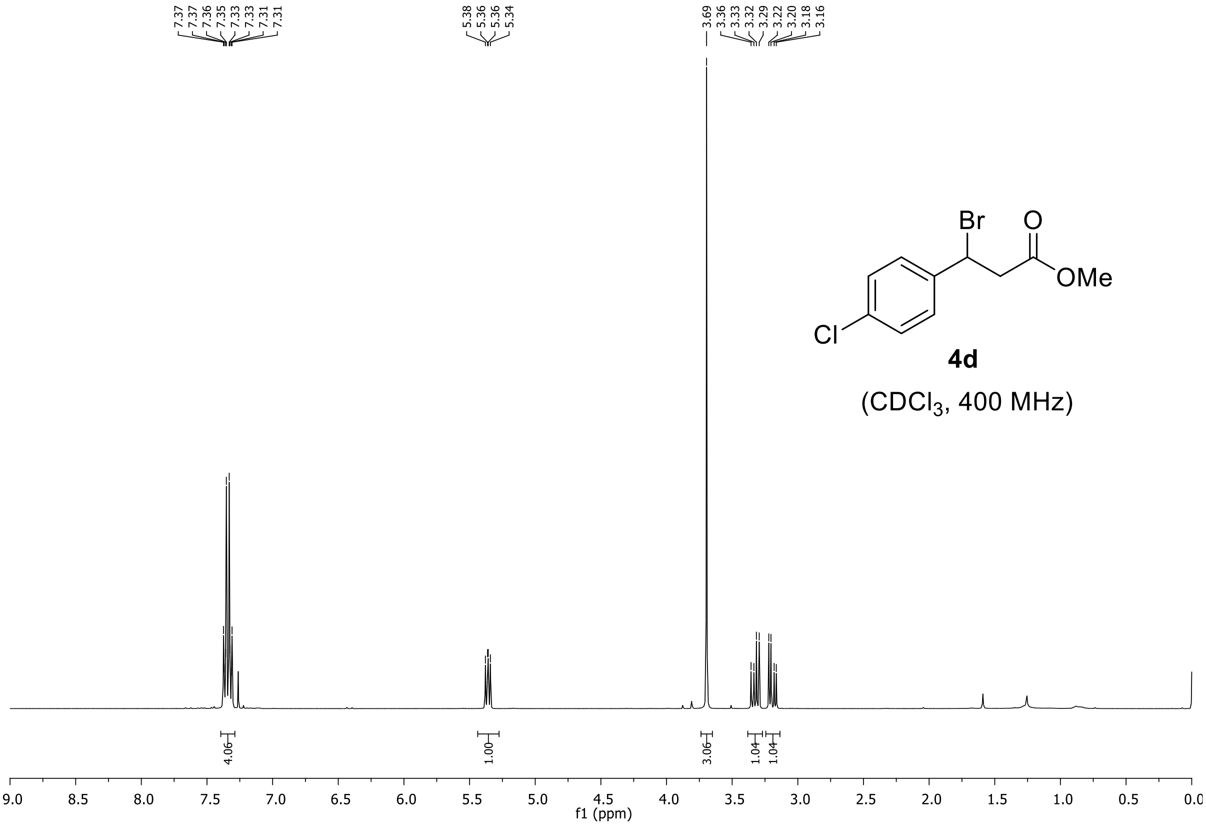
7.37 7.37 7.36 7.35 7.33 7.33 7.31 7.31
5.38 5.36 5.36 5.34
3.69 3.36 3.33 3.32 3.29 3.22 3.20 3.18 3.16
Br
O
OMe
Cl
4d
(CDCl3, 400 MHz)
4.06
1.00
3.06
1.04
1.04
f1 (ppm)

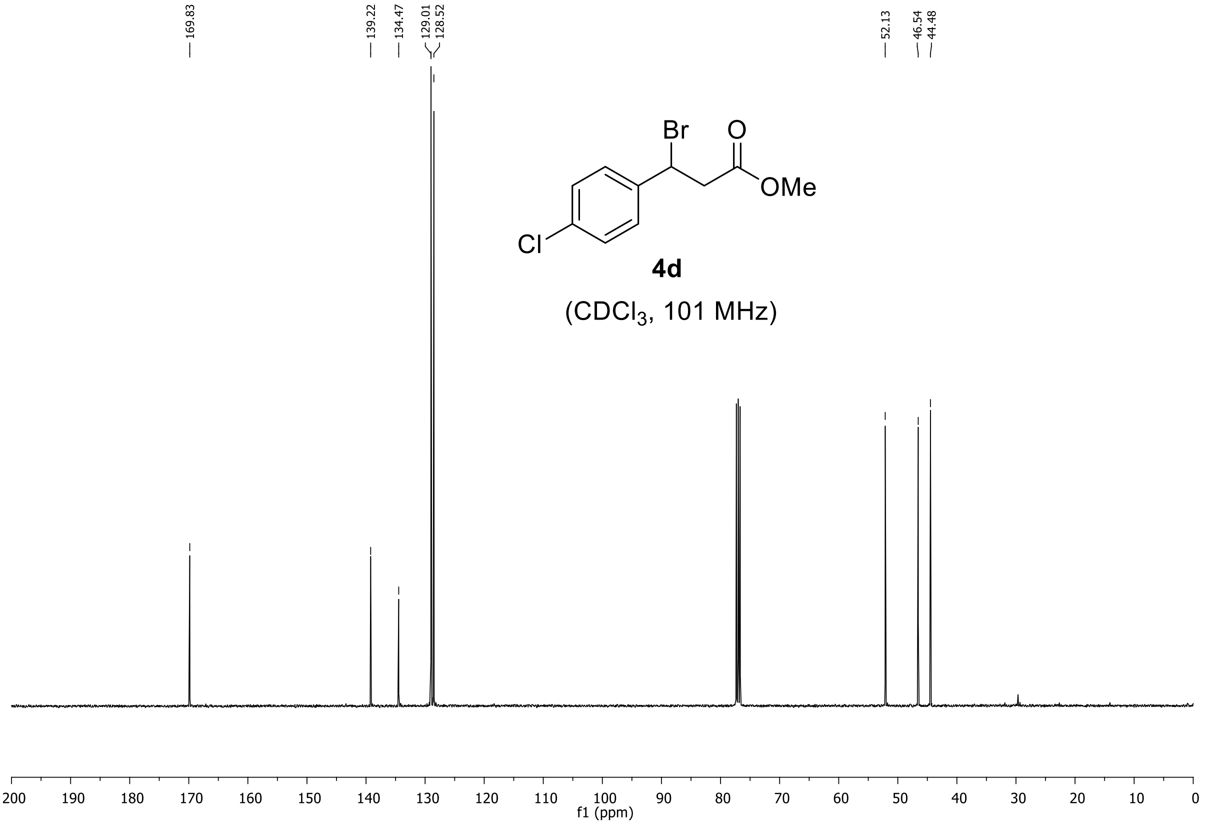
169.83
139.22
134.47
129.01
128.52
52.13
46.54
44.48
Br
O
OMe
Cl
4d
(CDCl3, 101 MHz)
f1 (ppm)

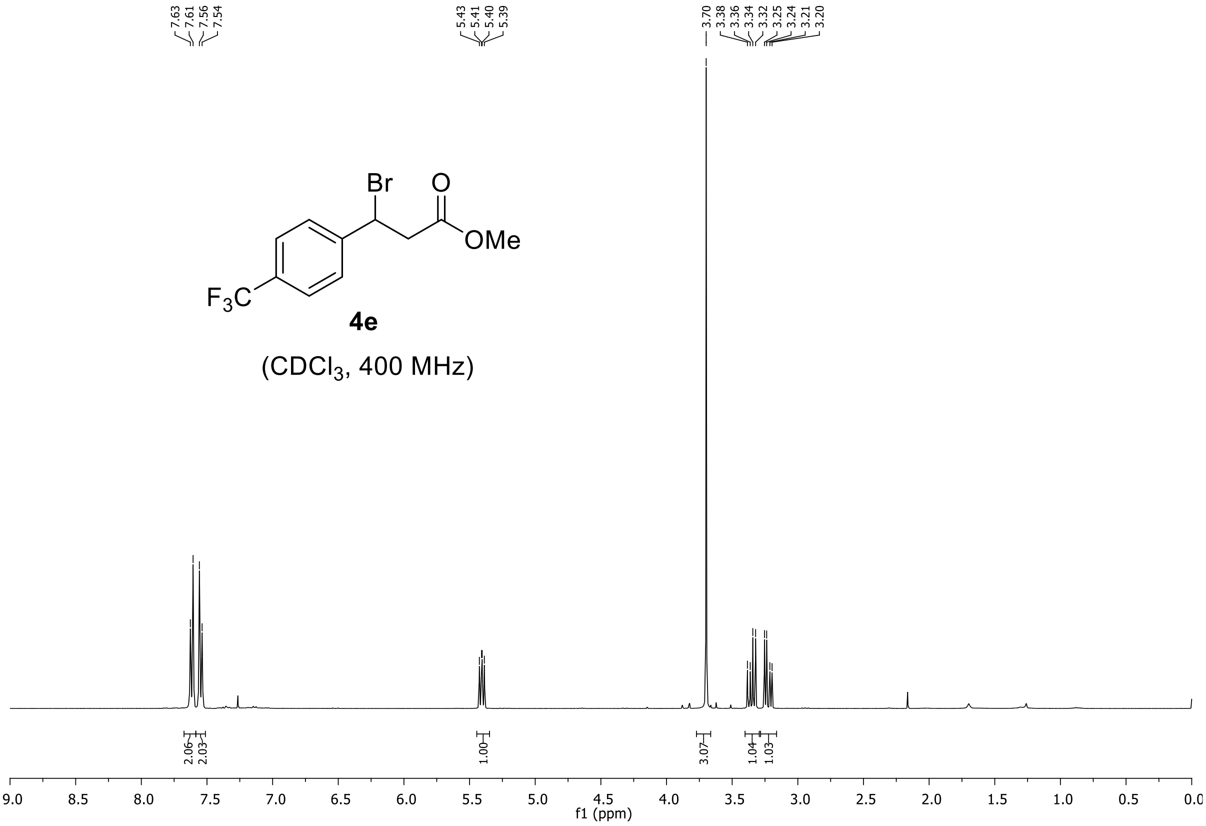
Br
O
OMe
F3C
4e
(CDCl3, 400 MHz)
f1 (ppm)

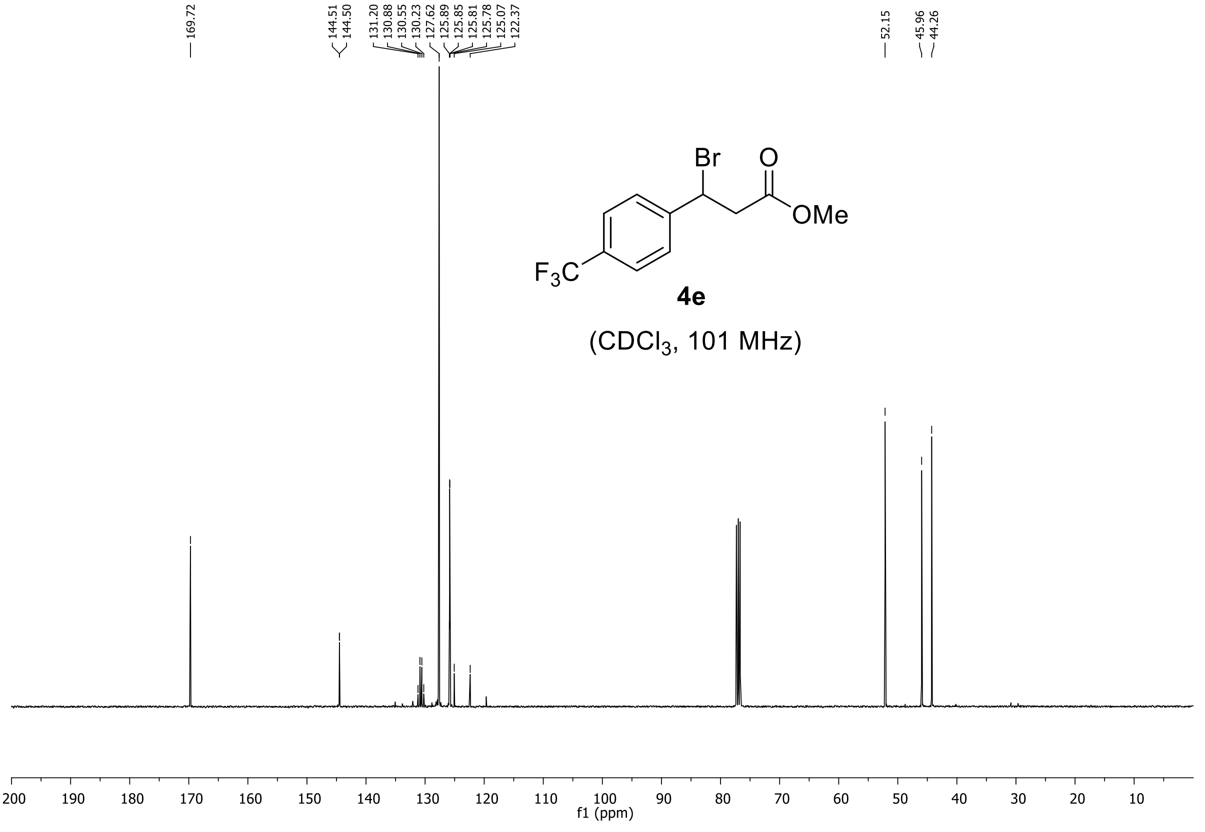
Br
O
OMe
F3C
4e
(CDCl3, 101 MHz)
f1 (ppm)

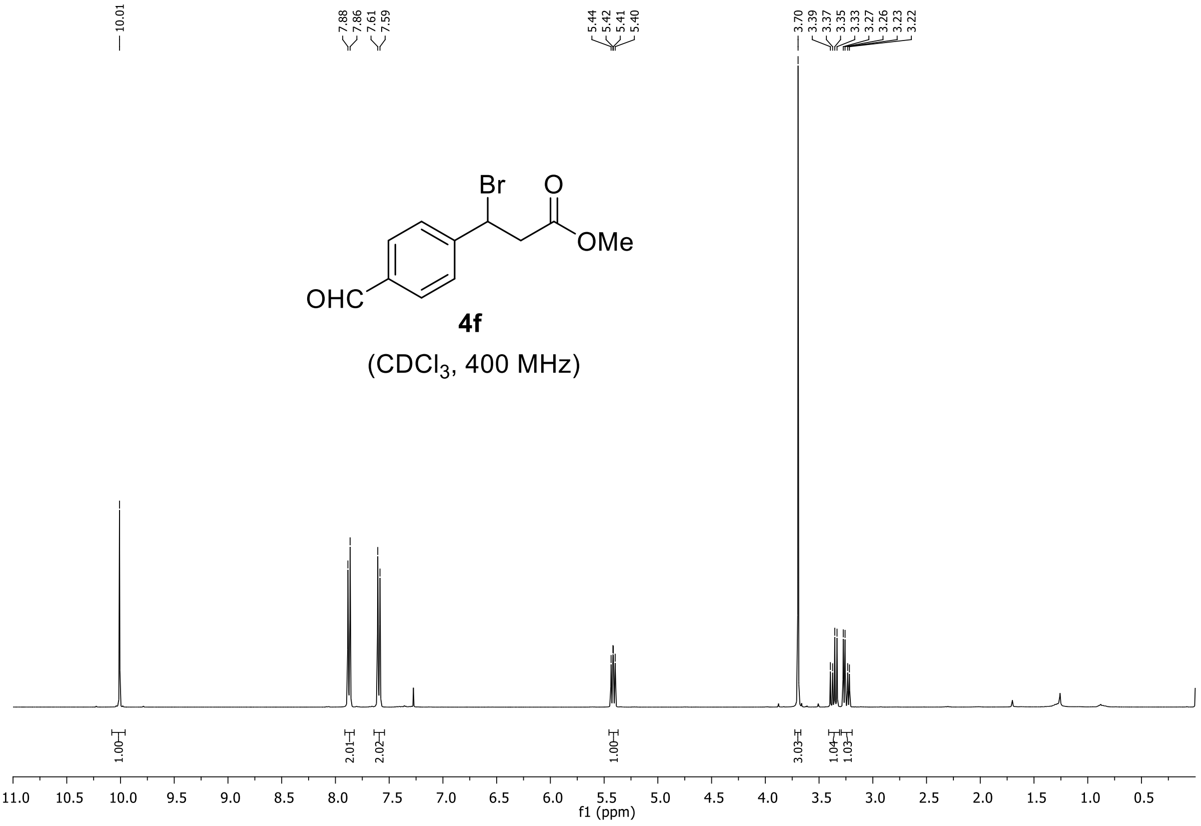
10.01
7.88
7.86
7.61
7.59
5.44
5.42
5.41
5.40
3.70
3.39
3.37
3.35
3.33
3.27
3.26
3.23
3.22
Br
O
OMe
OHC
4f
(CDCl3, 400 MHz)
1.00
2.01
2.02
1.00
3.03
1.04
1.03
f1 (ppm)

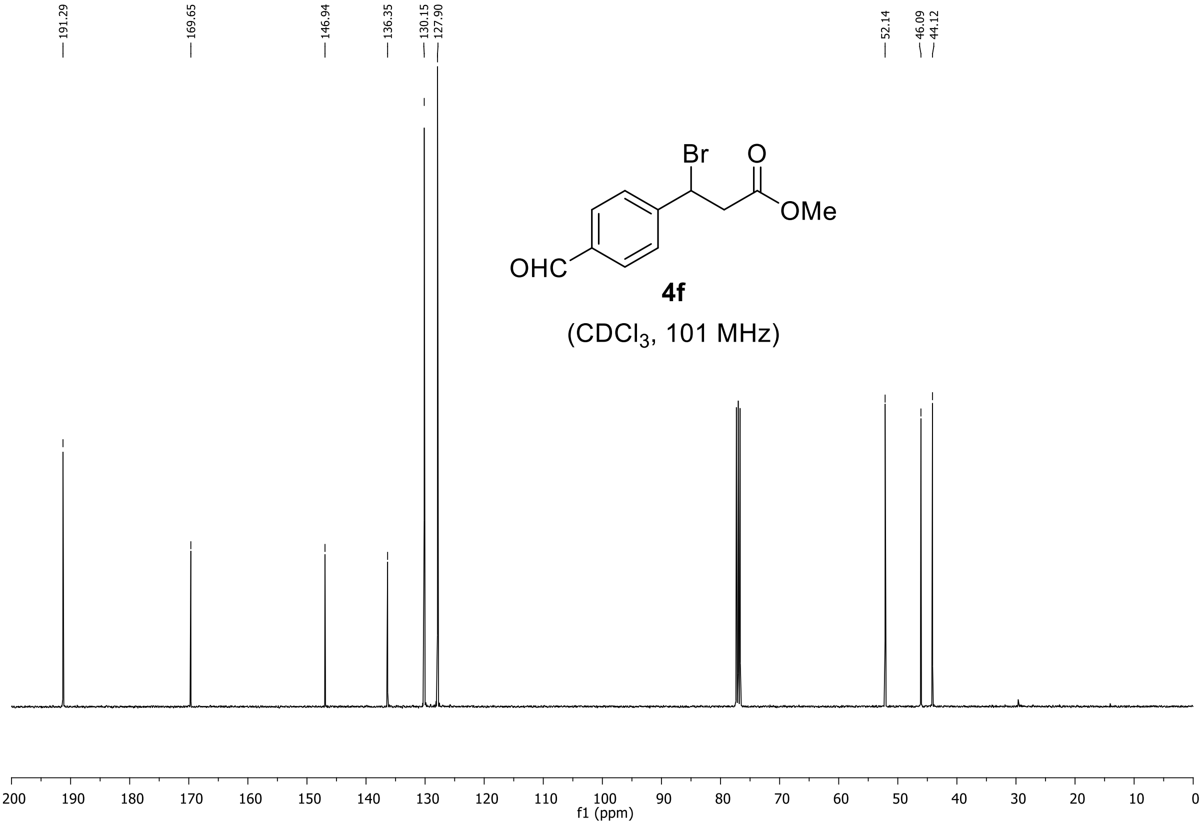
191.29
169.65
146.94
136.35
130.15
127.90
52.14
46.09
44.12
Br
O
OMe
OHC
4f
(CDCl3, 101 MHz)
f1 (ppm)

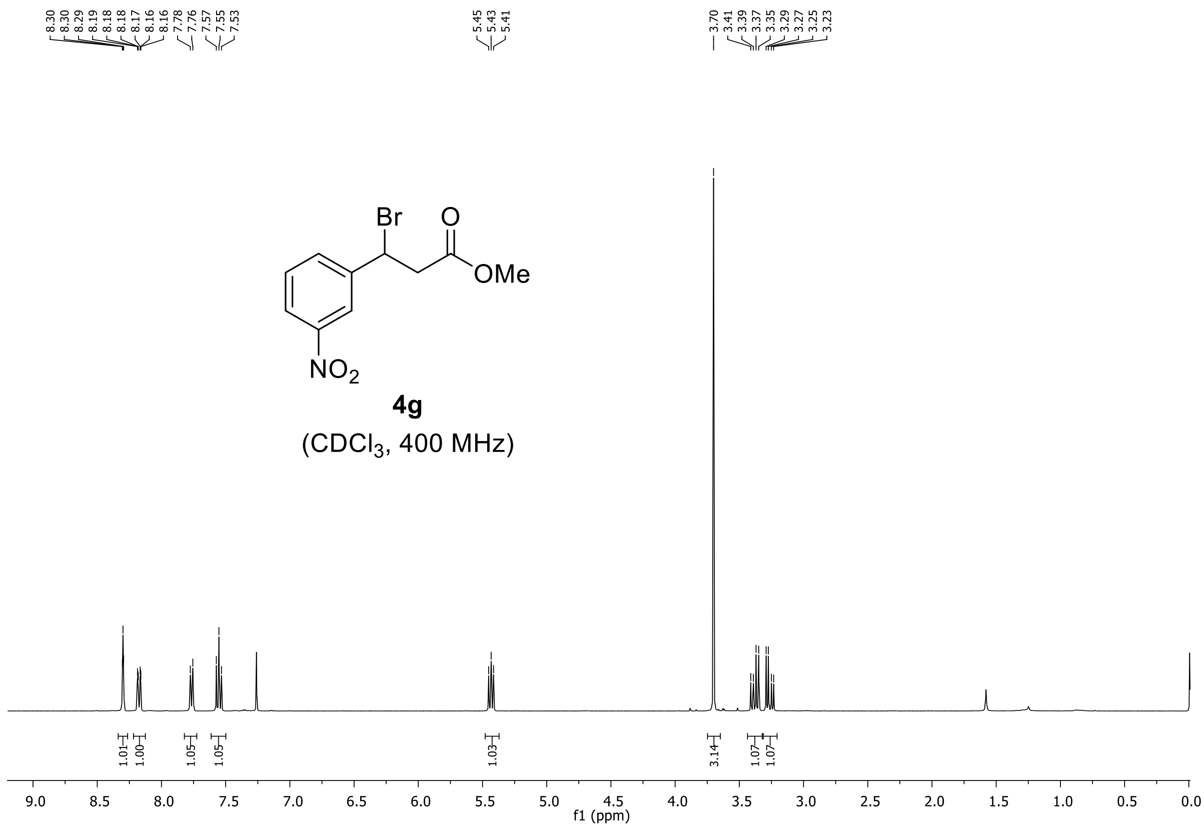
8.30 8.30 8.29 8.19 8.18 8.18 8.17 8.16 8.16 7.78 7.76 7.57 7.55 7.53
5.45 5.43 5.41
3.70 3.41 3.39 3.37 3.35 3.29 3.27 3.25 3.23
Br
O
OMe
NO2
4g
(CDCl3, 400 MHz)
1.01 1.00 1.05 1.05 1.03 3.14 1.07 1.07
f1 (ppm)

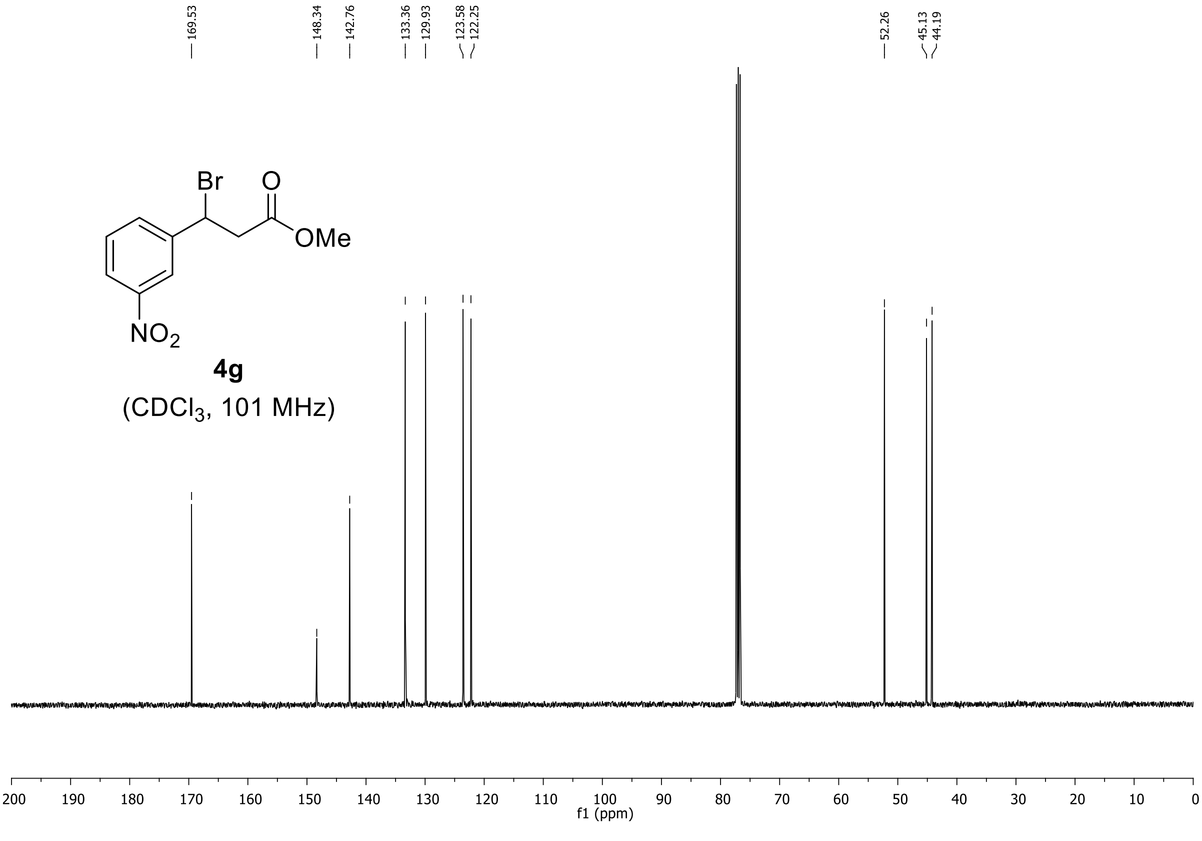
169.53
148.34
142.76
133.36
129.93
123.58
122.25
52.26
45.13
44.19
Br
O
OMe
NO2
4g
(CDCl3, 101 MHz)
f1 (ppm)

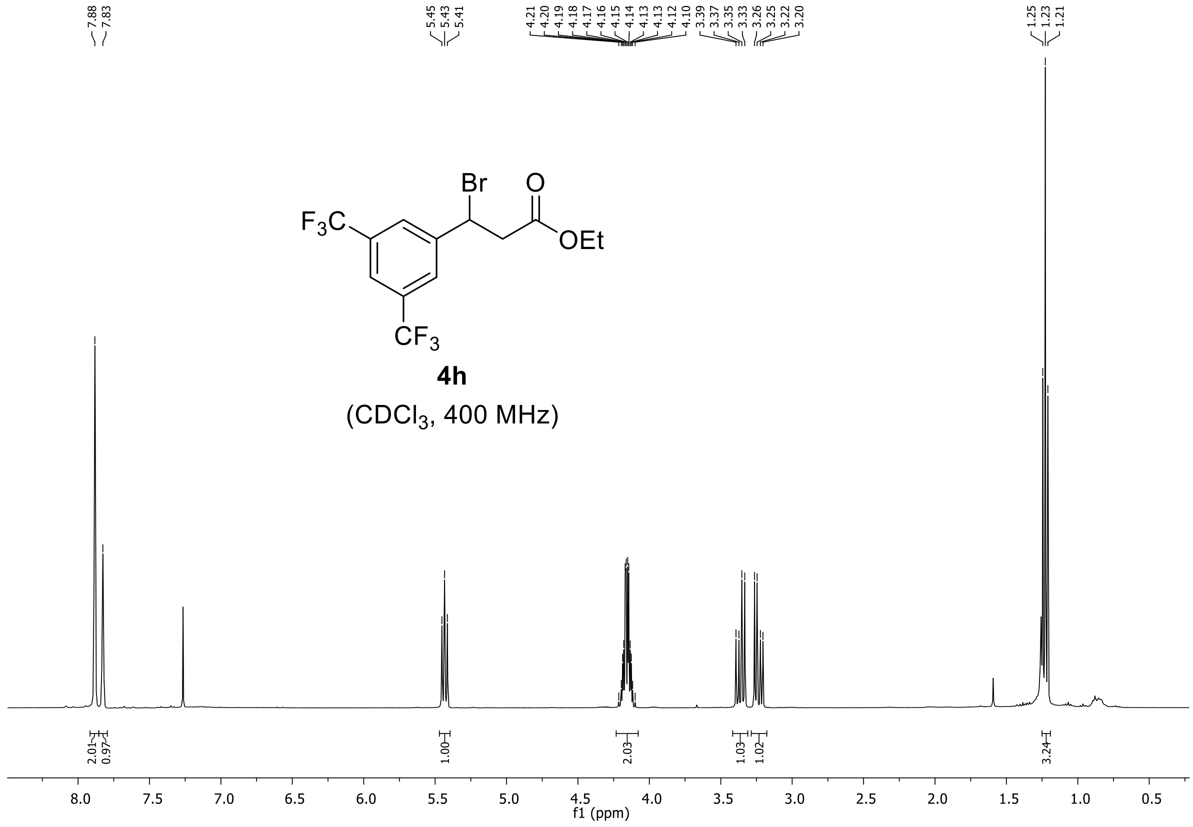

Br
O
F_3C
OEt
CF_3
4h
($CDCl_3$, 400 MHz)
f1 (ppm)

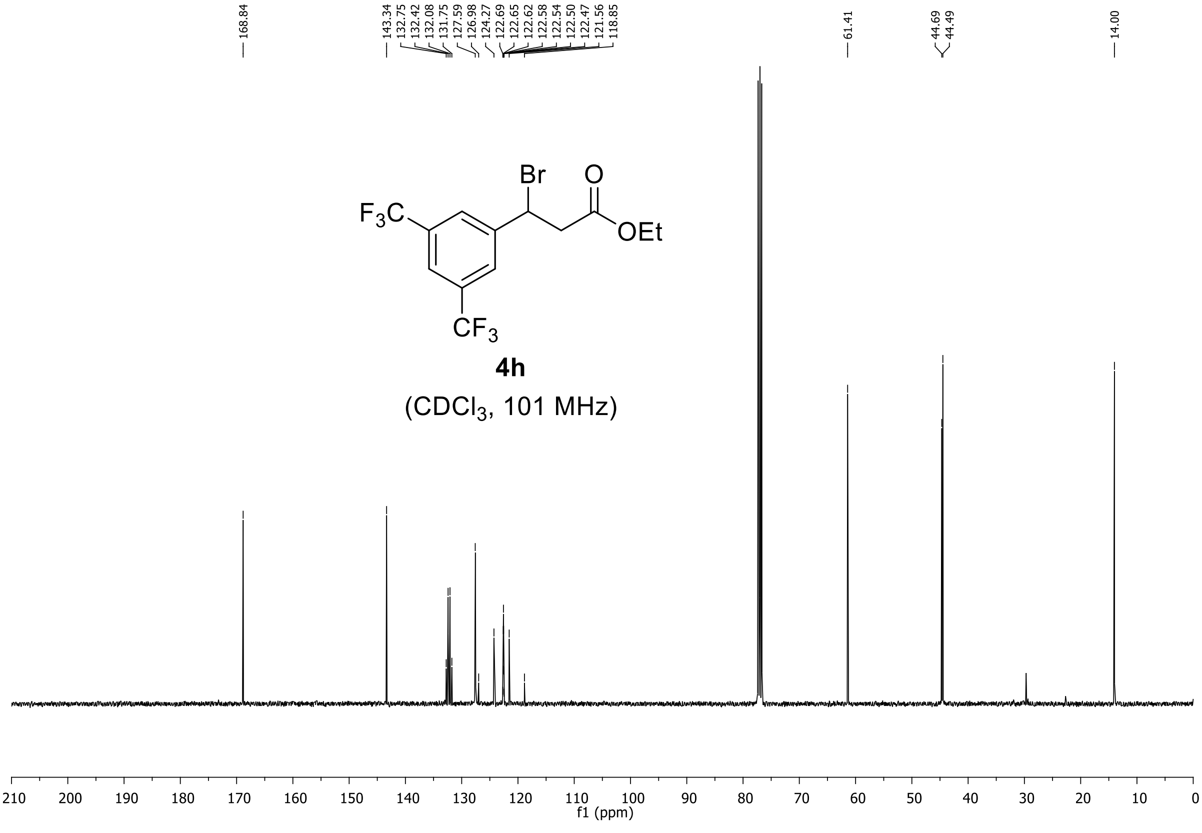

Br
O
F_3C
OEt
CF_3
4h
($CDCl_3$, 101 MHz)
f1 (ppm)

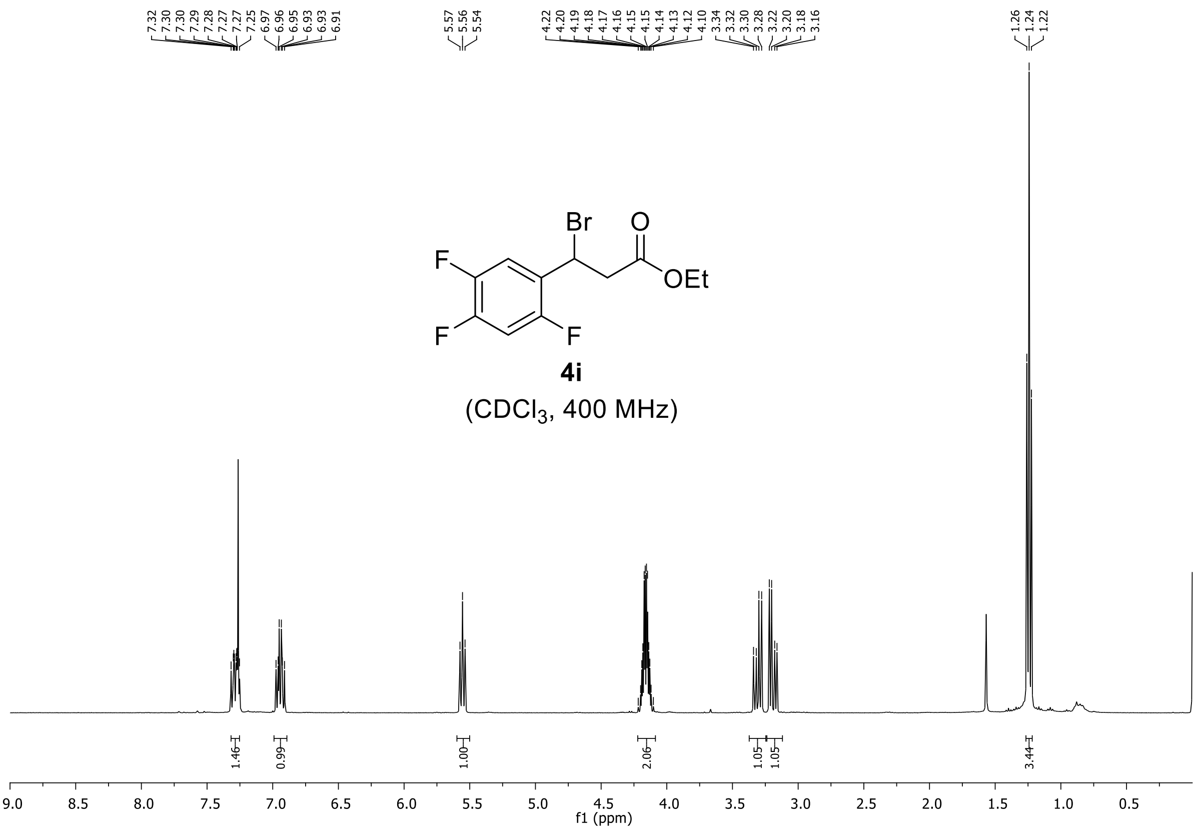
Br
O
F
OEt
F
F
4i
(CDCl$_3$, 400 MHz)
f1 (ppm)

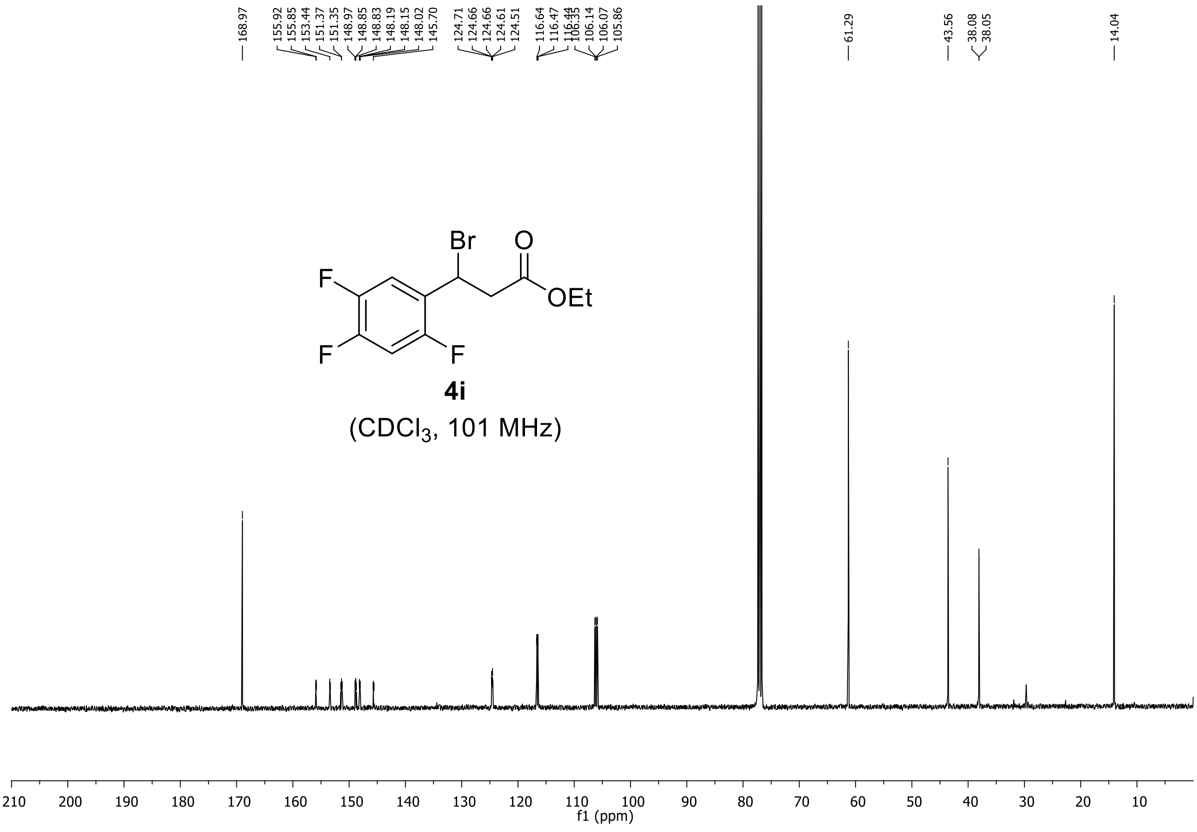
Br
O
F
OEt
F
F
4i
(CDCl$_3$, 101 MHz)
f1 (ppm)

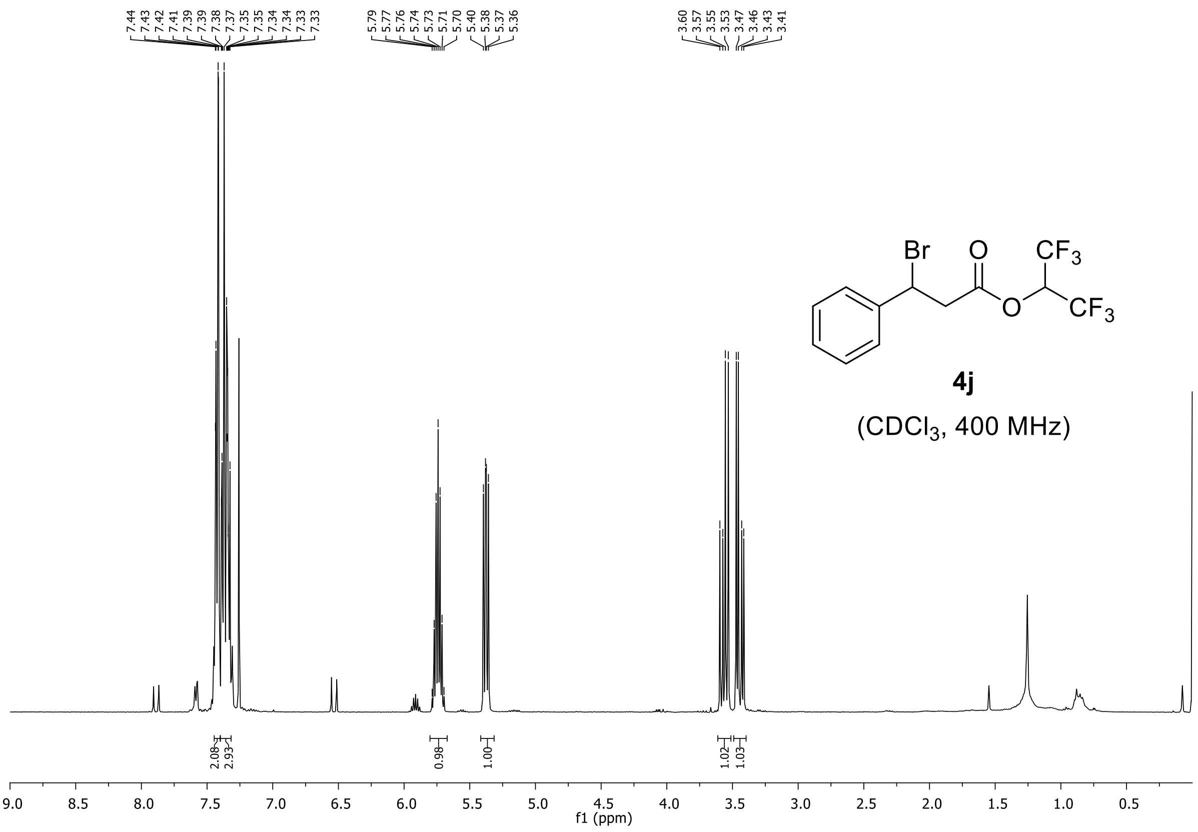
7.44 7.43 7.42 7.41 7.39 7.39 7.38 7.37 7.35 7.35 7.34 7.34 7.33 7.33
5.79 5.77 5.76 5.74 5.73 5.71 5.70 5.40 5.38 5.37 5.36
3.60 3.57 3.55 3.53 3.47 3.46 3.43 3.41
Br O CF3 O CF3
4j
(CDCl3, 400 MHz)
2.08 2.93 0.98 1.00 1.02 1.03
9.0 8.5 8.0 7.5 7.0 6.5 6.0 5.5 5.0 4.5 4.0 3.5 3.0 2.5 2.0 1.5 1.0 0.5
f1 (ppm)

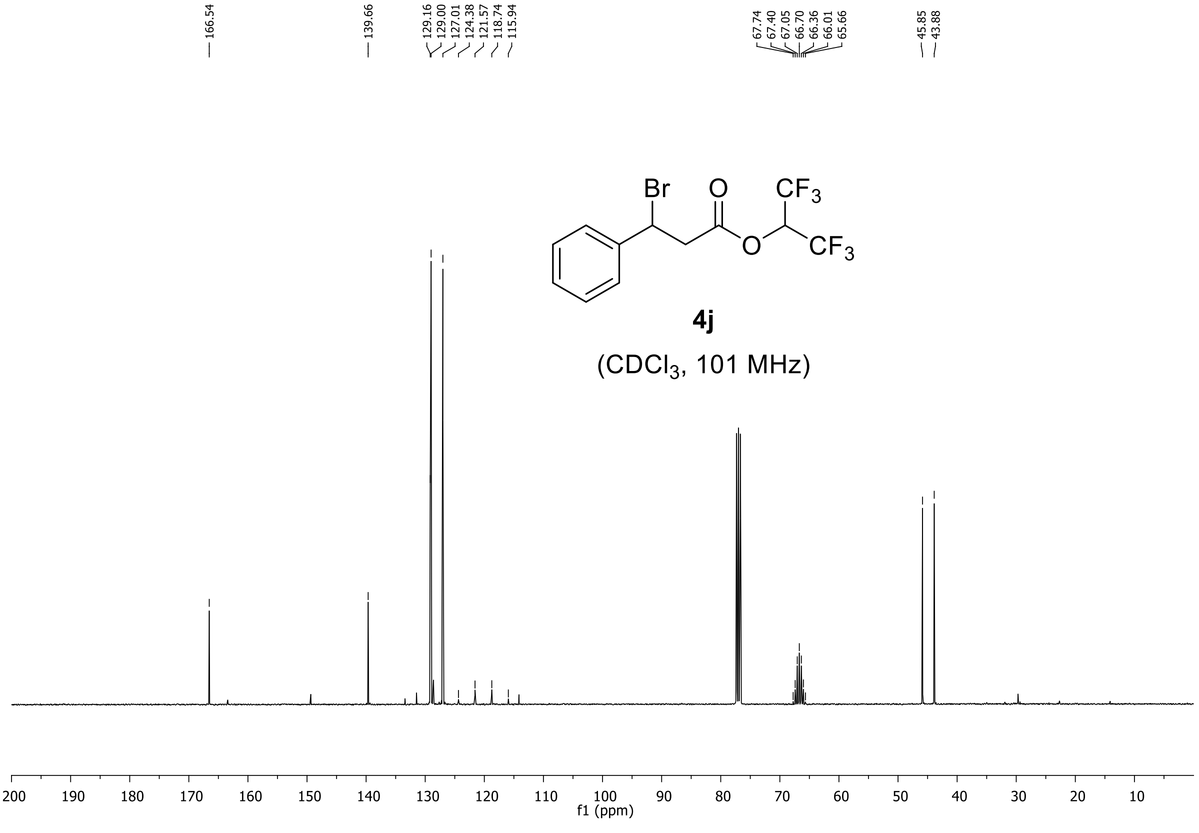
166.54
139.66
129.16 129.00 127.01 124.38 121.57 118.74 115.94
67.74 67.40 67.05 66.70 66.36 66.01 65.66
45.85 43.88
Br O CF3 O CF3
4j
(CDCl3, 101 MHz)
200 190 180 170 160 150 140 130 120 110 100 90 80 70 60 50 40 30 20 10
f1 (ppm)

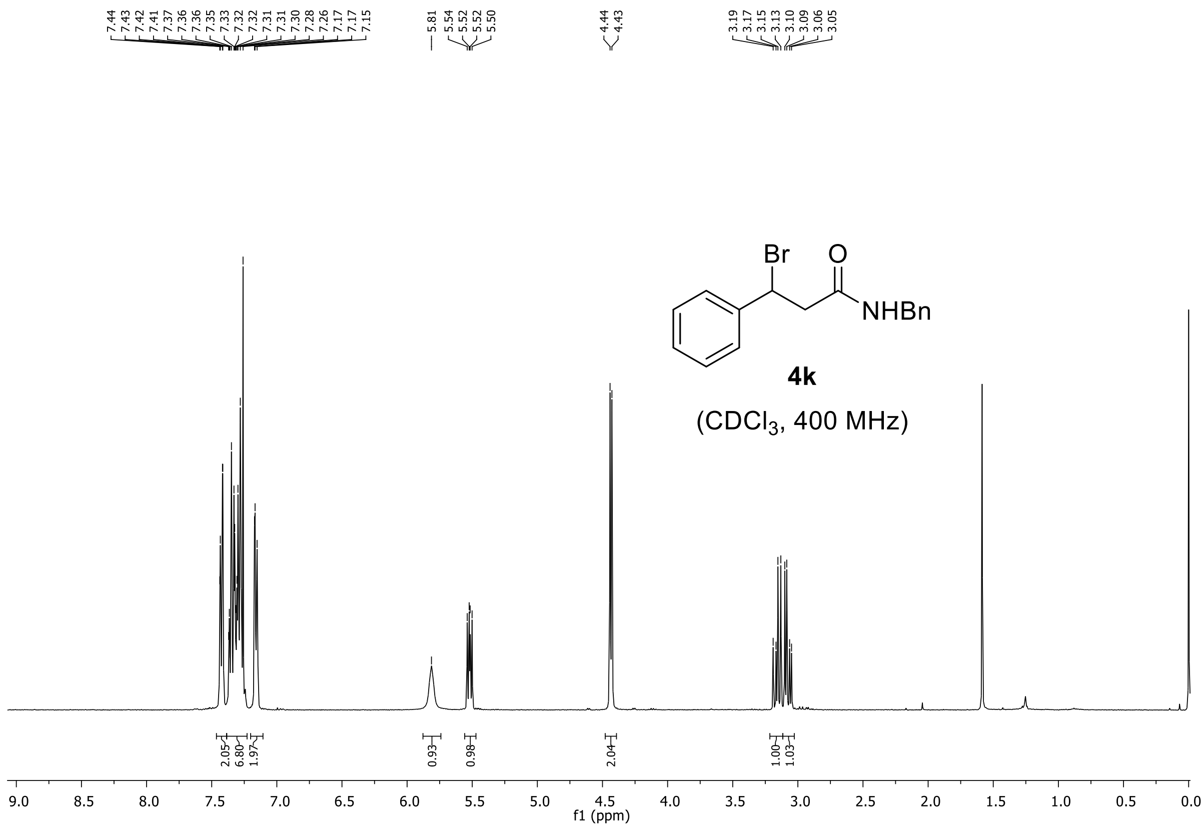
7.44 7.43 7.42 7.41 7.37 7.36 7.36 7.35 7.33 7.32 7.32 7.31 7.31 7.30 7.28 7.26 7.17 7.15
5.81 5.54 5.52 5.52 5.50
4.44 4.43
3.19 3.17 3.15 3.13 3.10 3.09 3.06 3.05
Br
O
NHBn
4k
(CDCl$_3$, 400 MHz)
2.05 6.80 1.97 0.93 0.98 2.04 1.00 1.03
f1 (ppm)

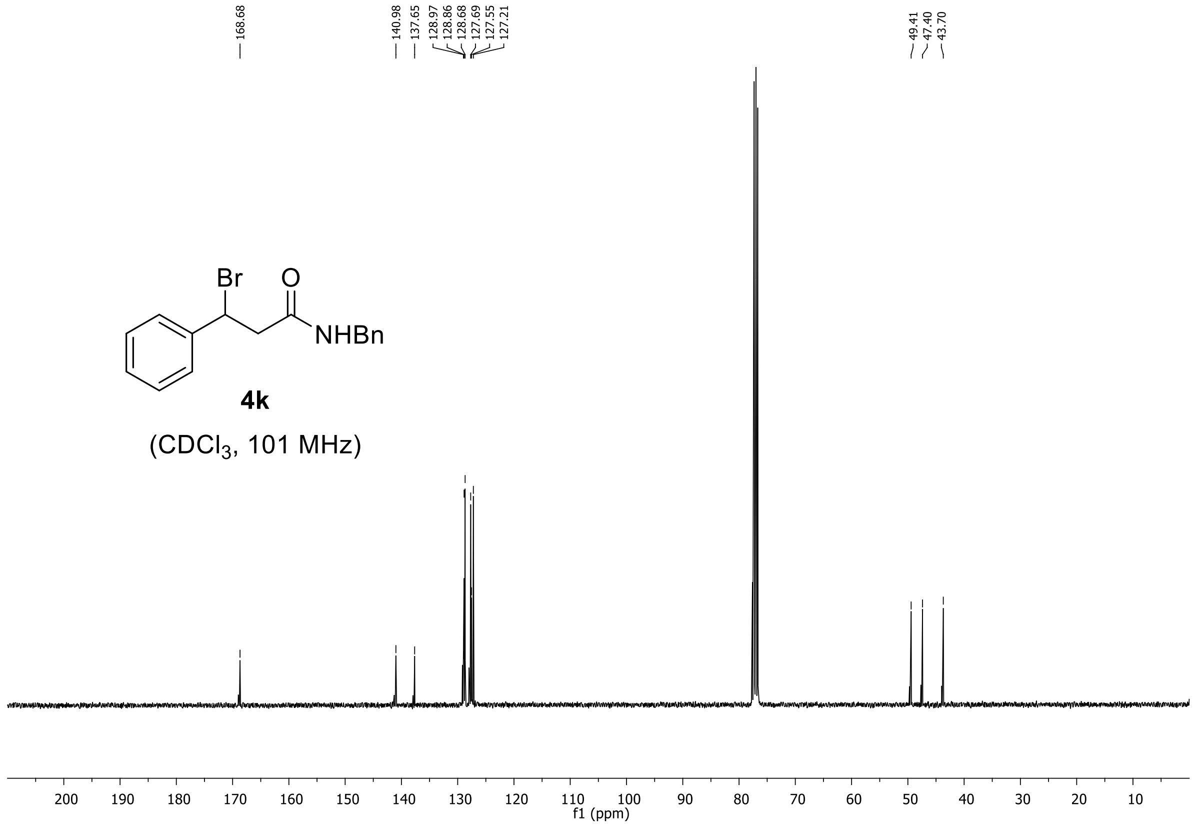
168.68
140.98 137.65 128.97 128.86 128.68 127.69 127.55 127.21
49.41 47.40 43.70
Br
O
NHBn
4k
(CDCl$_3$, 101 MHz)
f1 (ppm)

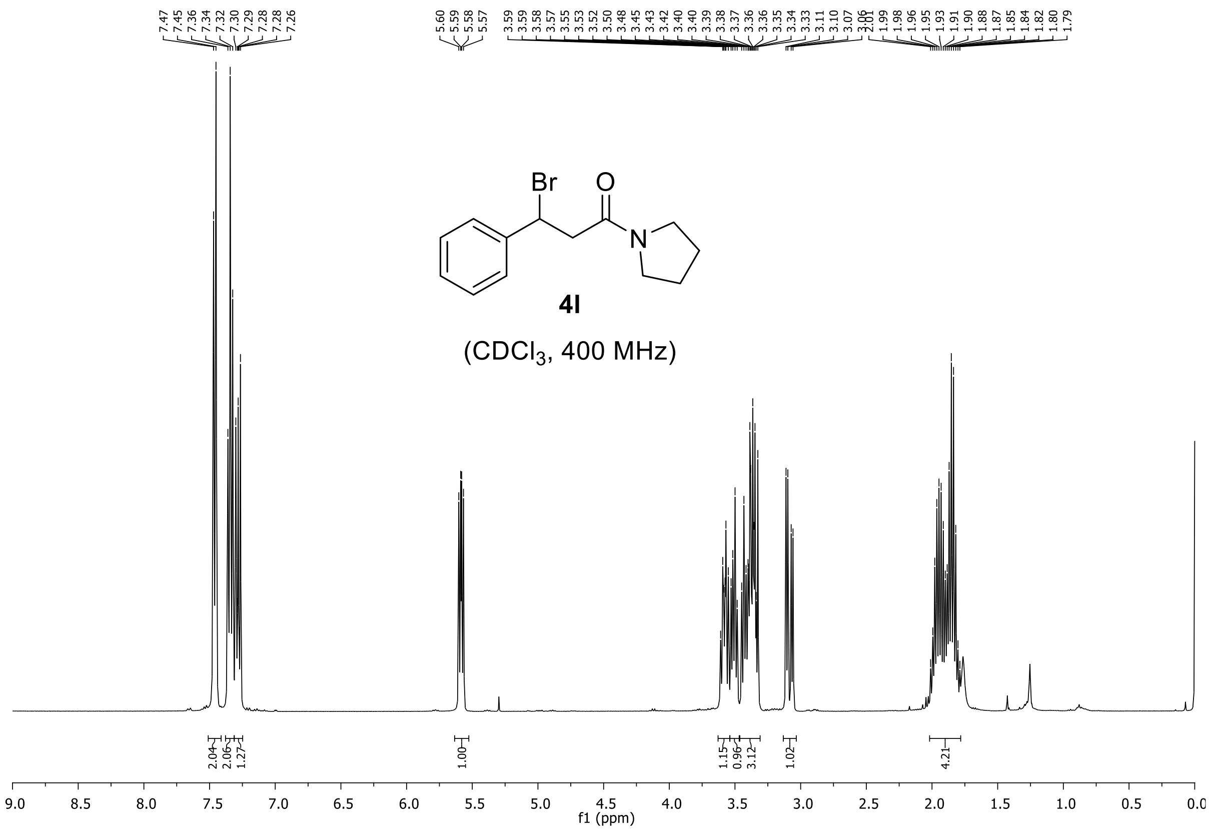
Br
O
N
4l
(CDCl3, 400 MHz)
2.04
2.06
1.27
1.00
1.15
0.96
3.12
1.02
4.21
f1 (ppm)

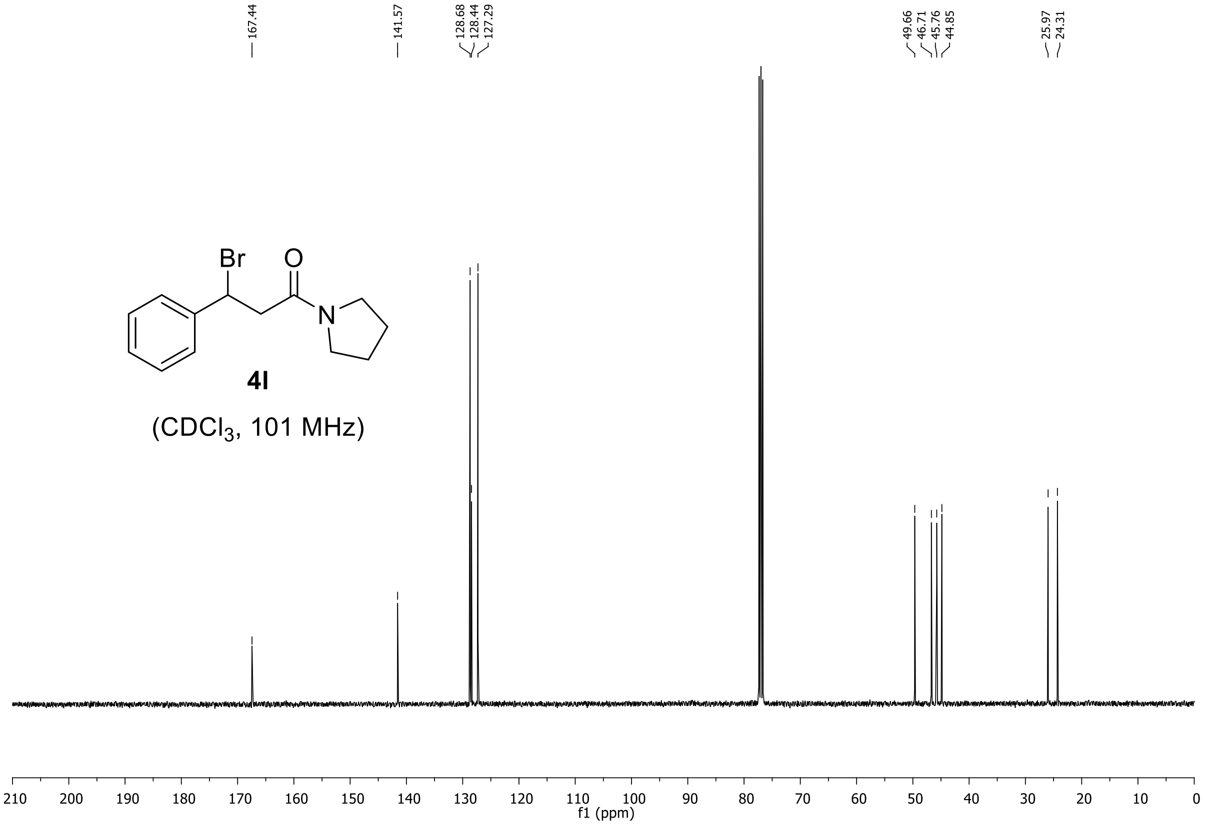
167.44
141.57
128.68
128.44
127.29
49.66
46.71
45.76
44.85
25.97
24.31
Br
O
N
4l
(CDCl3, 101 MHz)
f1 (ppm)

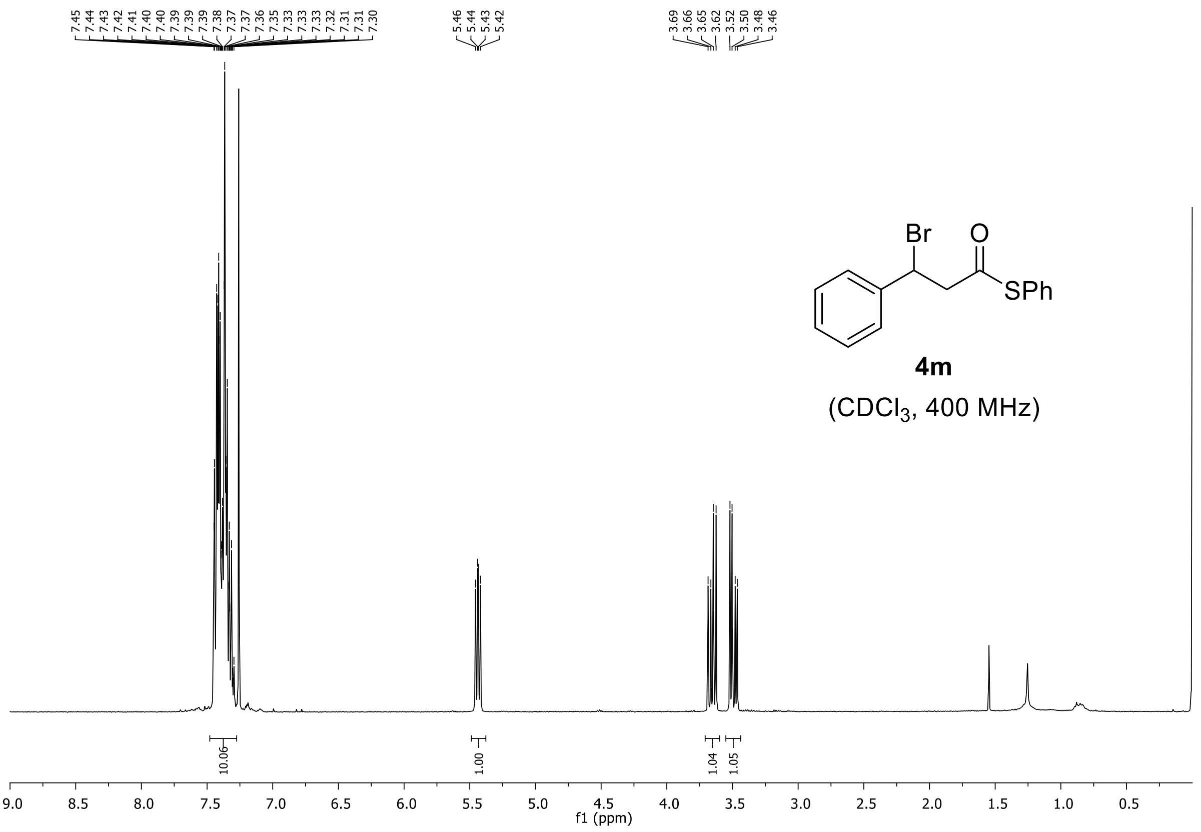
7.45
7.44
7.43
7.42
7.41
7.40
7.39
7.39
7.39
7.38
7.37
7.37
7.36
7.35
7.33
7.33
7.33
7.32
7.31
7.31
7.30
5.46
5.44
5.43
5.42
3.69
3.66
3.65
3.62
3.52
3.50
3.48
3.46
Br
O
SPh
4m
(CDCl3, 400 MHz)
10.06
1.00
1.04
1.05
9.0
8.5
8.0
7.5
7.0
6.5
6.0
5.5
5.0
4.5
4.0
3.5
3.0
2.5
2.0
1.5
1.0
0.5
f1 (ppm)

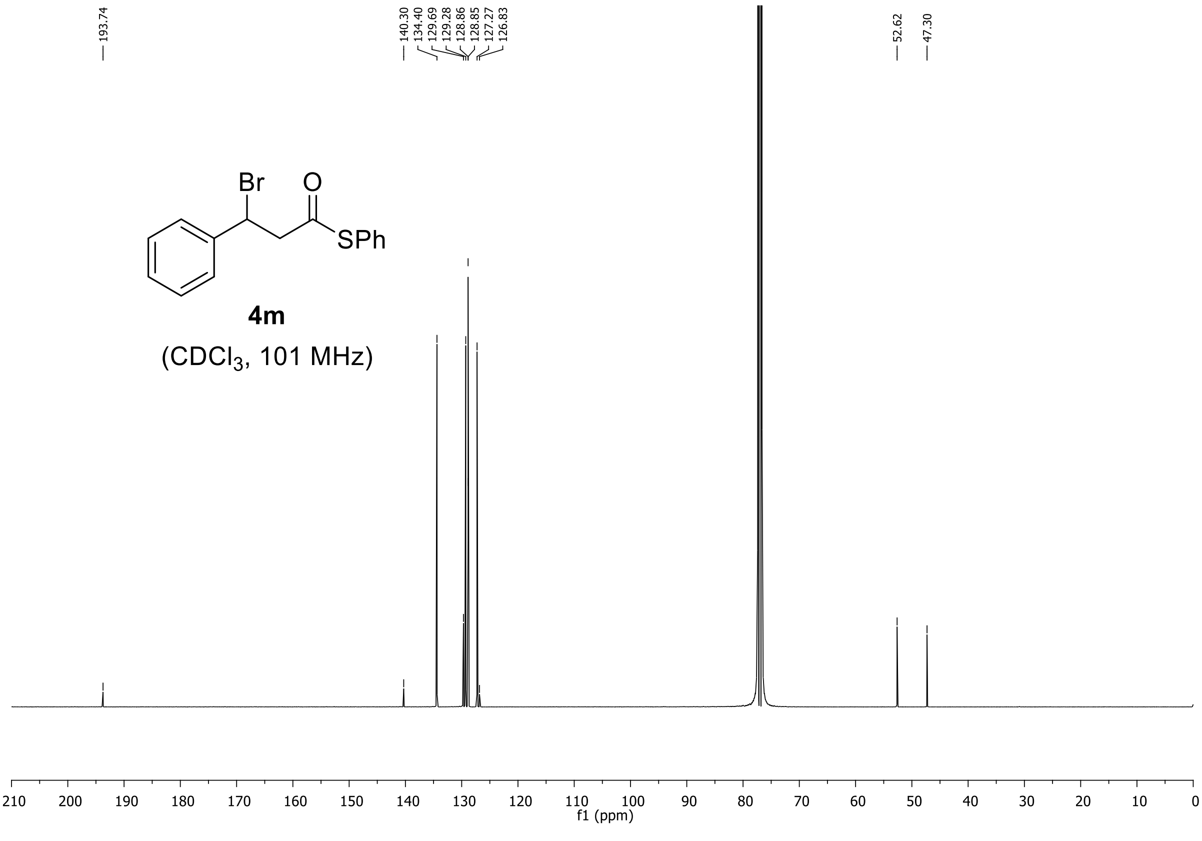
193.74
140.30
134.40
129.69
129.28
128.86
128.85
127.27
126.83
52.62
47.30
Br
O
SPh
4m
(CDCl3, 101 MHz)
210
200
190
180
170
160
150
140
130
120
110
100
90
80
70
60
50
40
30
20
10
0
f1 (ppm)

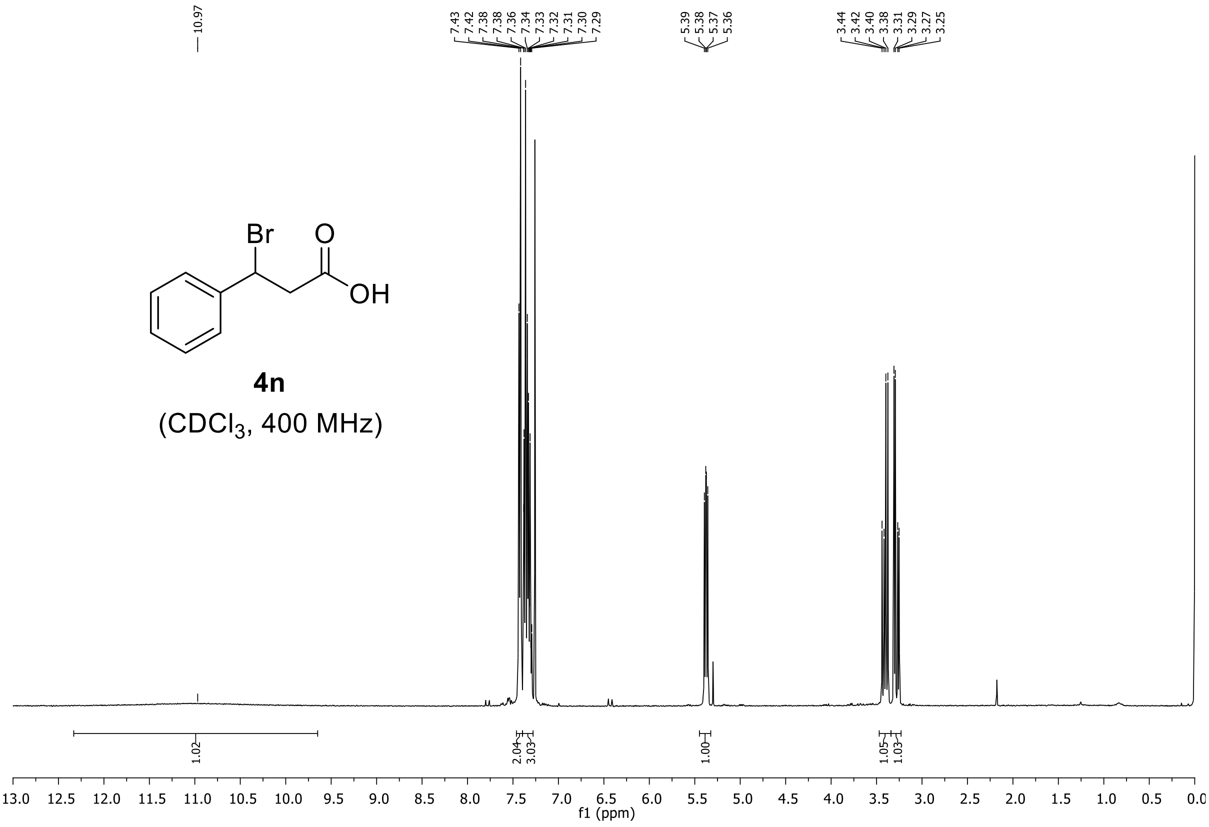
10.97
7.43
7.42
7.38
7.38
7.36
7.34
7.33
7.32
7.31
7.30
7.29
5.39
5.38
5.37
5.36
3.44
3.42
3.40
3.38
3.31
3.29
3.27
3.25
Br
O
OH
4n
(CDCl3, 400 MHz)
1.02
2.04
3.03
1.00
1.05
1.03
f1 (ppm)

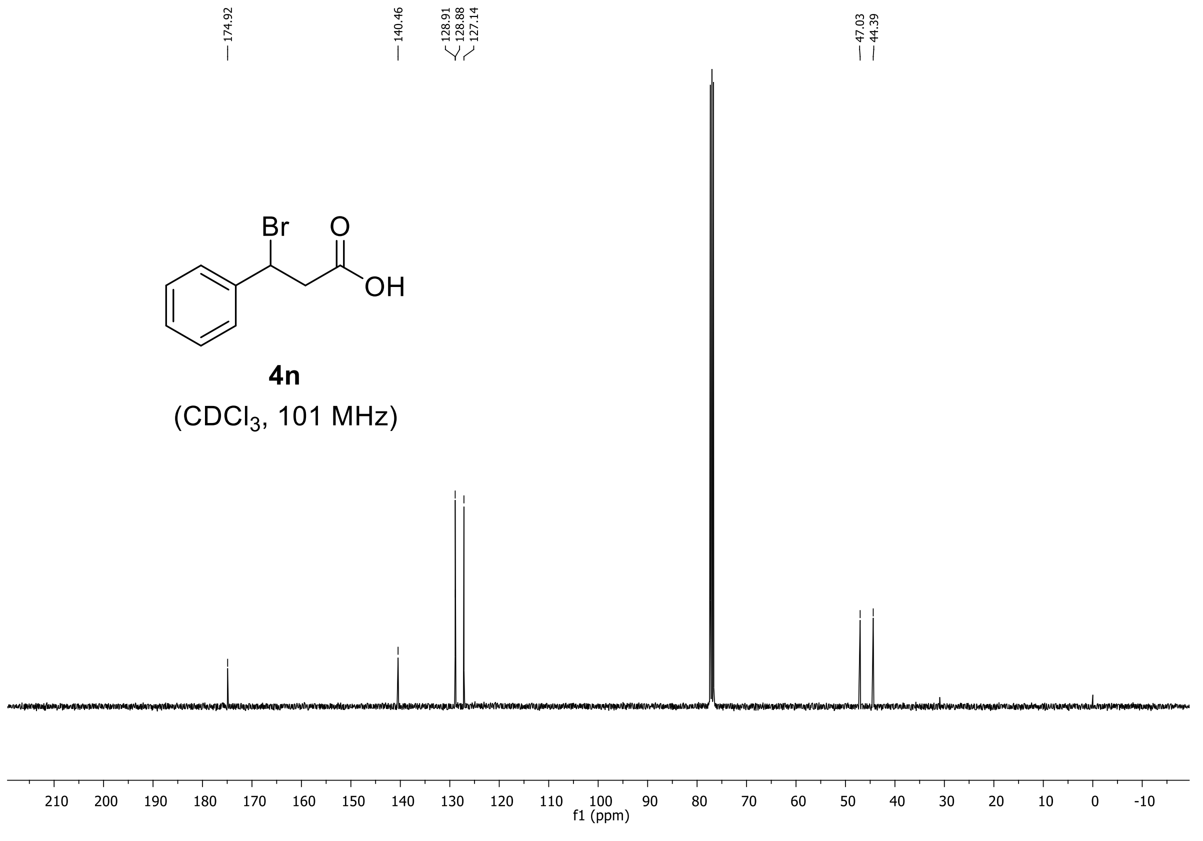
174.92
140.46
128.91
128.88
127.14
47.03
44.39
Br
O
OH
4n
(CDCl3, 101 MHz)
f1 (ppm)

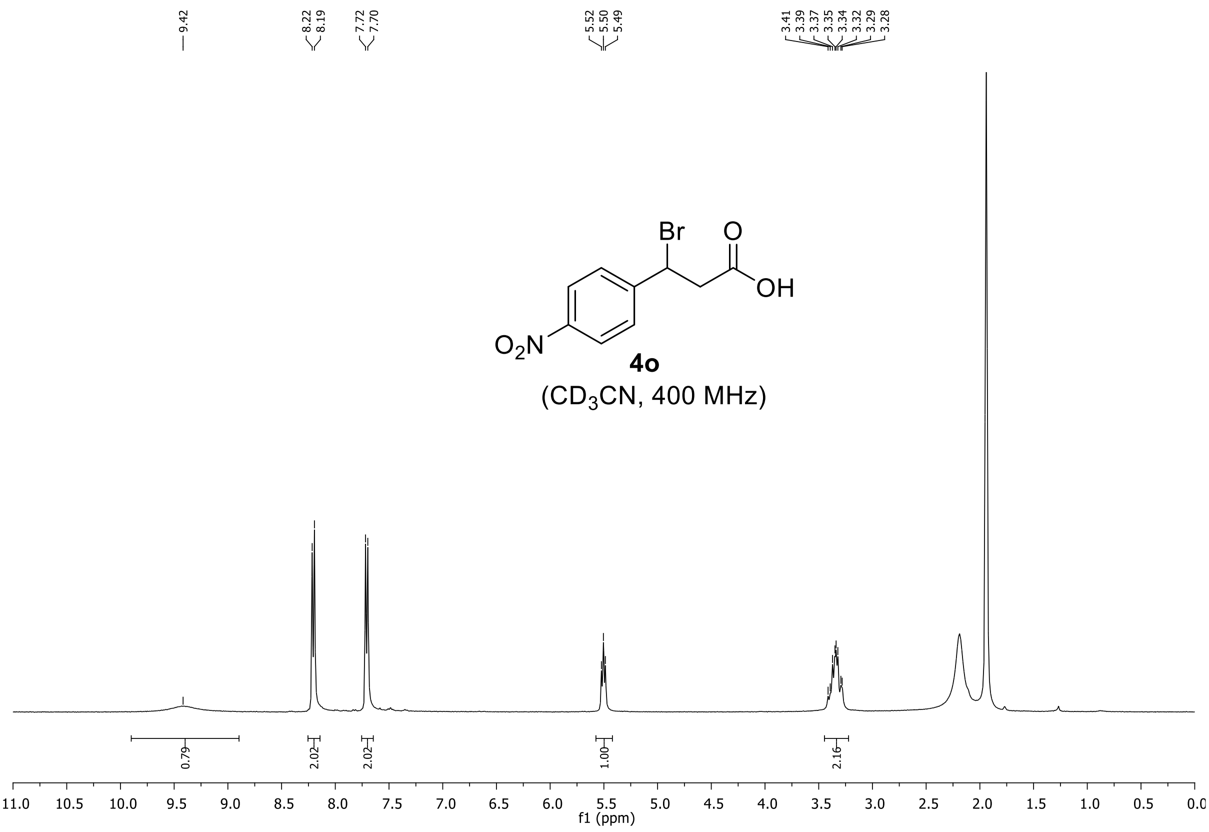
9.42
8.22
8.19
7.72
7.70
5.52
5.50
5.49
3.41
3.39
3.37
3.35
3.34
3.32
3.29
3.28
Br
O
OH
O2N
4o
(CD3CN, 400 MHz)
0.79
2.02
2.02
1.00
2.16
11.0
10.5
10.0
9.5
9.0
8.5
8.0
7.5
7.0
6.5
6.0
5.5
5.0
4.5
4.0
3.5
3.0
2.5
2.0
1.5
1.0
0.5
f1 (ppm)

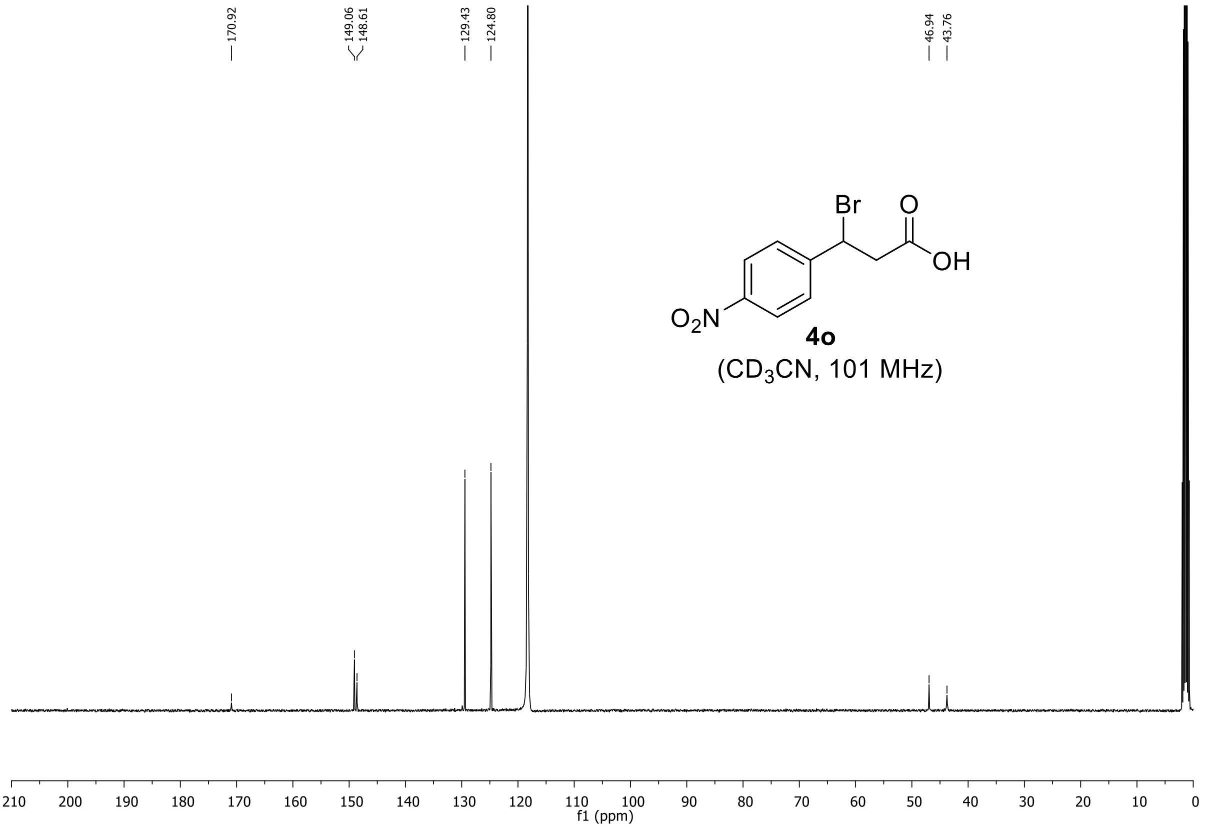
170.92
149.06
148.61
129.43
124.80
46.94
43.76
Br
O
OH
O2N
4o
(CD3CN, 101 MHz)
210
200
190
180
170
160
150
140
130
120
110
100
90
80
70
60
50
40
30
20
10
0
f1 (ppm)

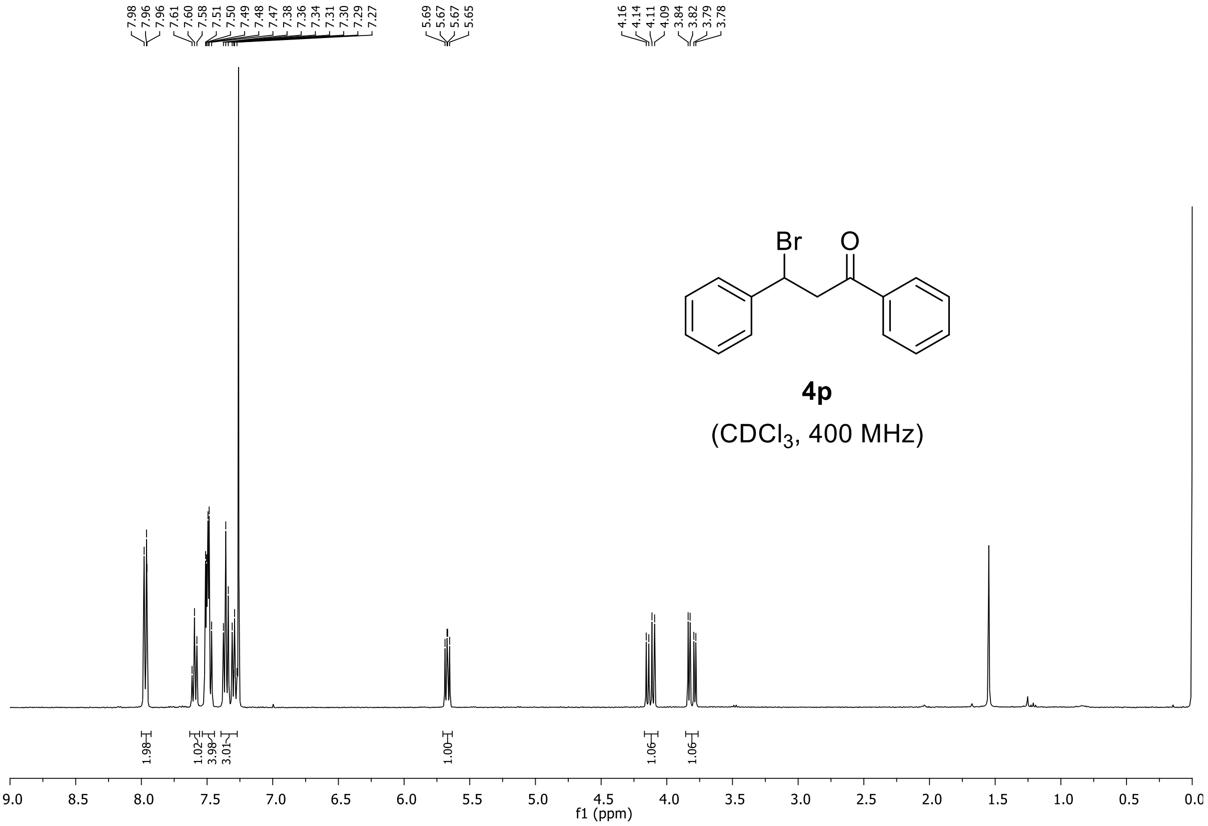
7.98
7.96
7.96
7.61
7.60
7.58
7.51
7.50
7.49
7.48
7.47
7.38
7.36
7.34
7.31
7.30
7.29
7.27
5.69
5.67
5.67
5.65
4.16
4.14
4.11
4.09
3.84
3.82
3.79
3.78
Br
O
4p
(CDCl3, 400 MHz)
1.98
1.02
3.98
3.01
1.00
1.06
1.06
f1 (ppm)

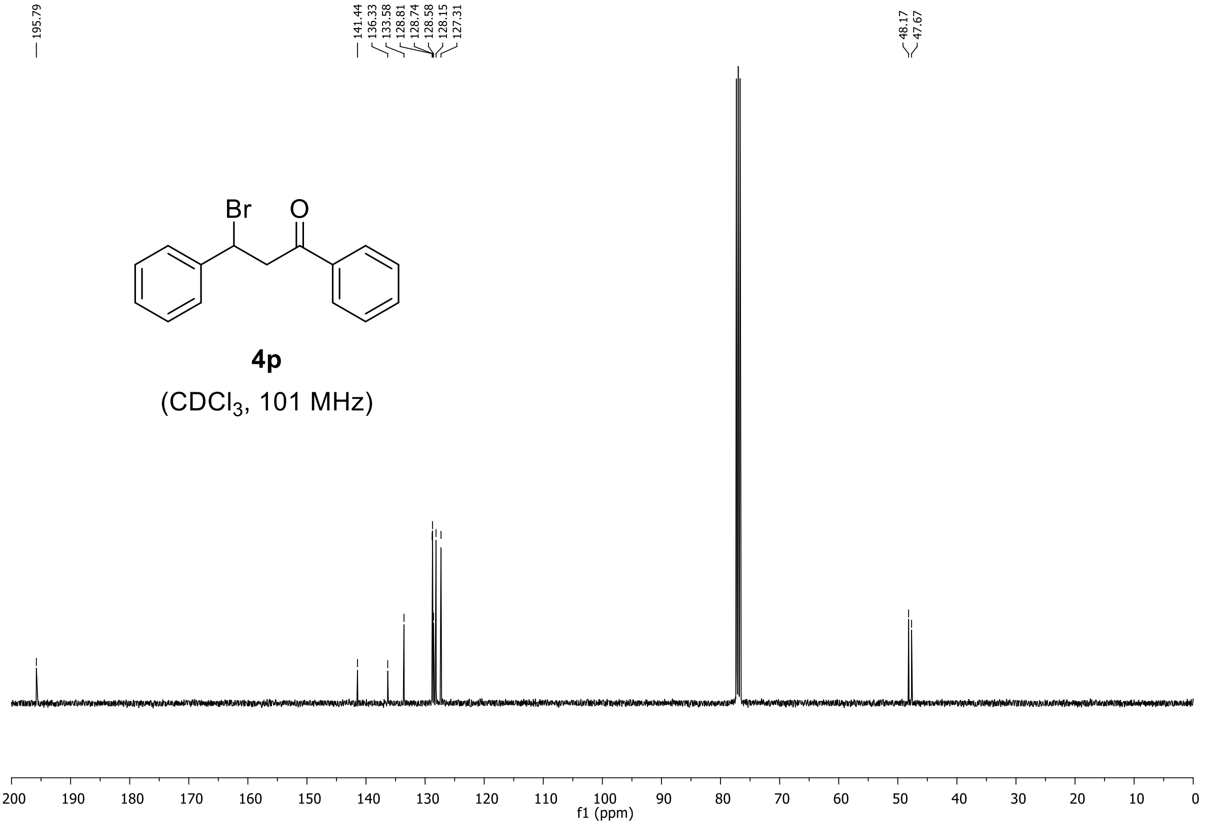
195.79
141.44
136.33
133.58
128.81
128.74
128.58
128.15
127.31
48.17
47.67
Br
O
4p
(CDCl3, 101 MHz)
f1 (ppm)

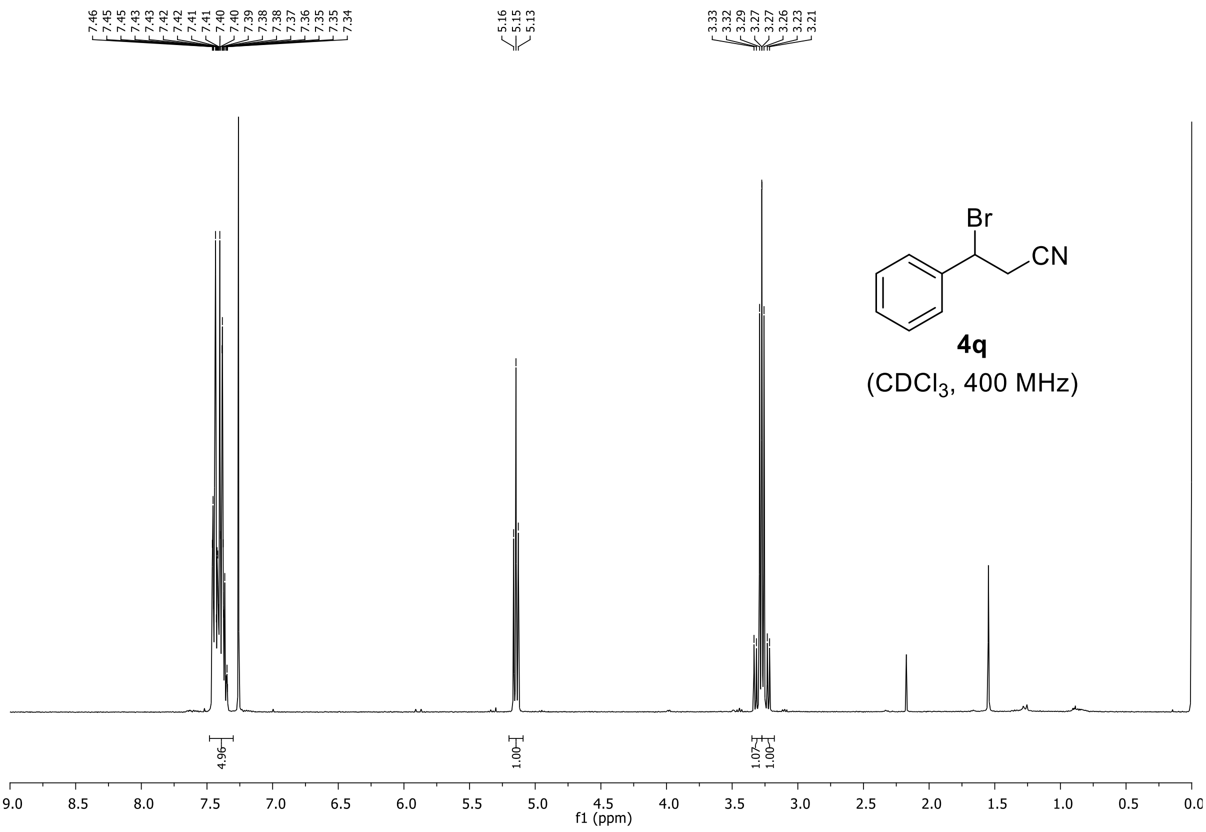

7.46
7.45
7.45
7.43
7.43
7.42
7.41
7.41
7.40
7.40
7.39
7.38
7.38
7.37
7.36
7.35
7.35
7.34
5.16
5.15
5.13
3.33
3.32
3.29
3.27
3.27
3.26
3.23
3.21
Br
CN
4q
(CDCl3, 400 MHz)
4.96
1.00
1.07
1.00
9.0
8.5
8.0
7.5
7.0
6.5
6.0
5.5
5.0
4.5
4.0
3.5
3.0
2.5
2.0
1.5
1.0
0.5
0.0
f1 (ppm)

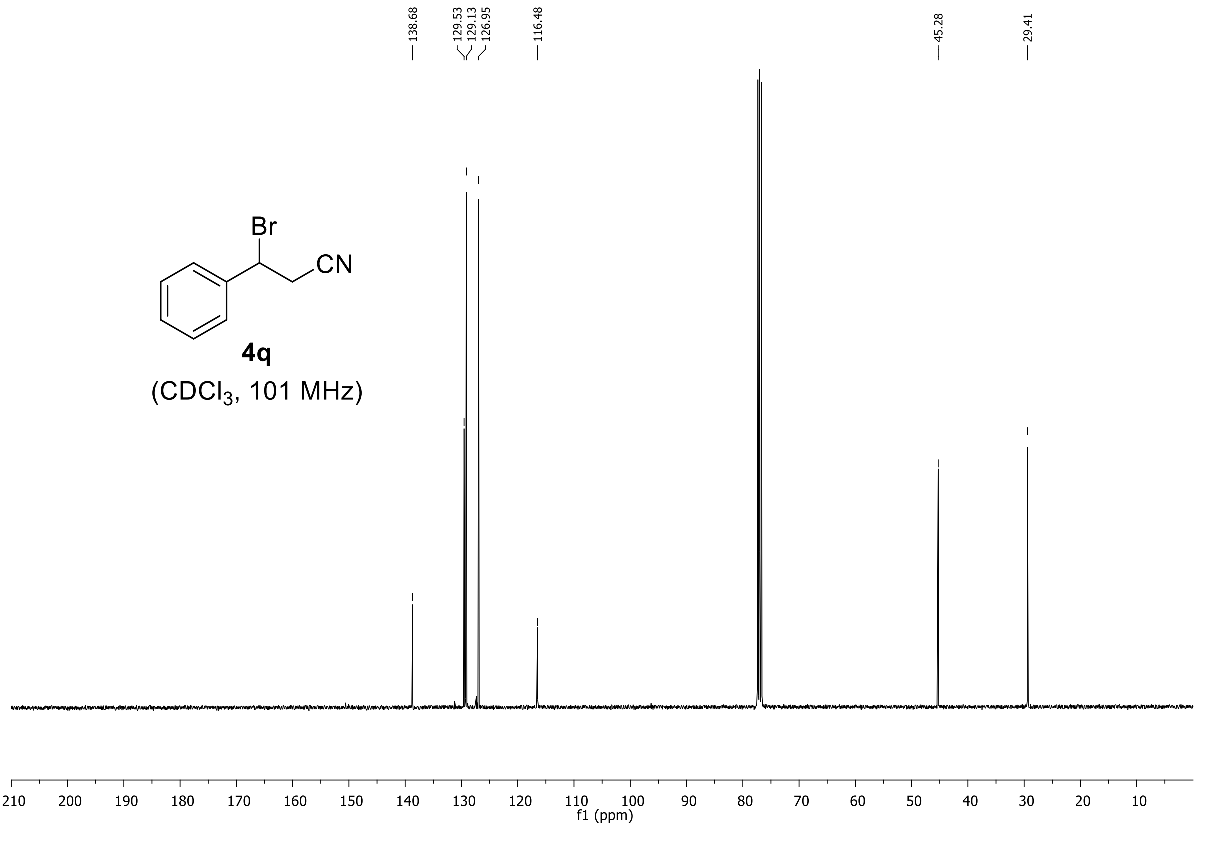

138.68
129.53
129.13
126.95
116.48
45.28
29.41
Br
CN
4q
(CDCl3, 101 MHz)
210
200
190
180
170
160
150
140
130
120
110
100
90
80
70
60
50
40
30
20
10
f1 (ppm)

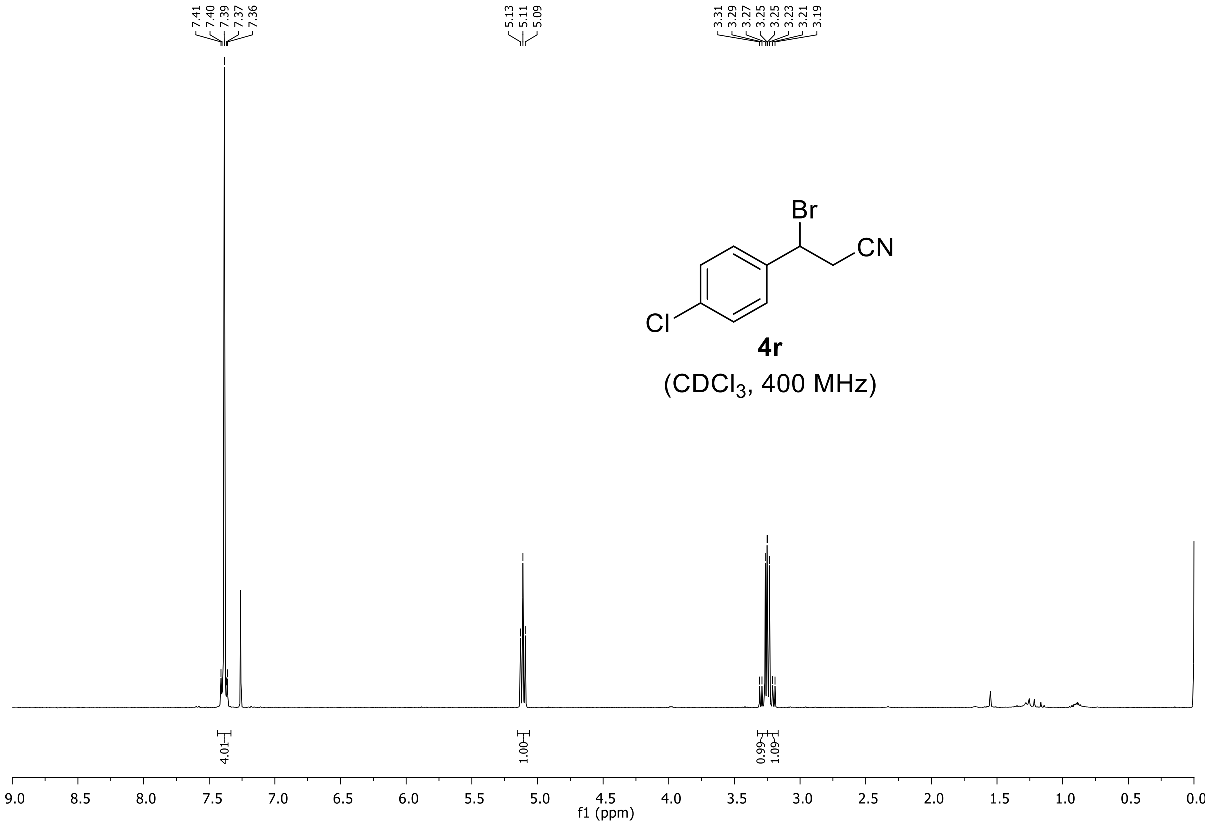
7.41
7.40
7.39
7.37
7.36
5.13
5.11
5.09
3.31
3.29
3.27
3.25
3.25
3.23
3.21
3.19
Br
CN
Cl
4r
(CDCl3, 400 MHz)
4.01
1.00
0.99
1.09
9.0 8.5 8.0 7.5 7.0 6.5 6.0 5.5 5.0 4.5 4.0 3.5 3.0 2.5 2.0 1.5 1.0 0.5 0.0
f1 (ppm)

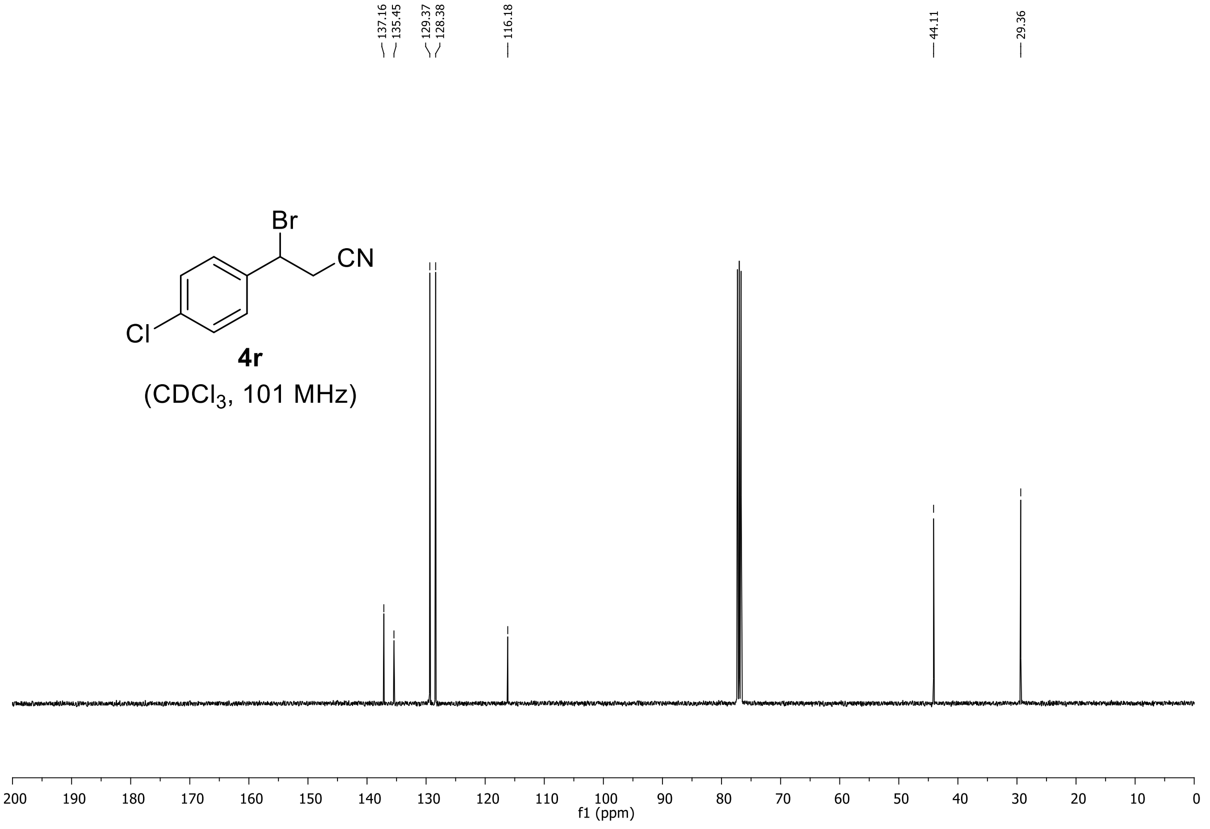
137.16
135.45
129.37
128.38
116.18
44.11
29.36
Br
CN
Cl
4r
(CDCl3, 101 MHz)
200 190 180 170 160 150 140 130 120 110 100 90 80 70 60 50 40 30 20 10 0
f1 (ppm)

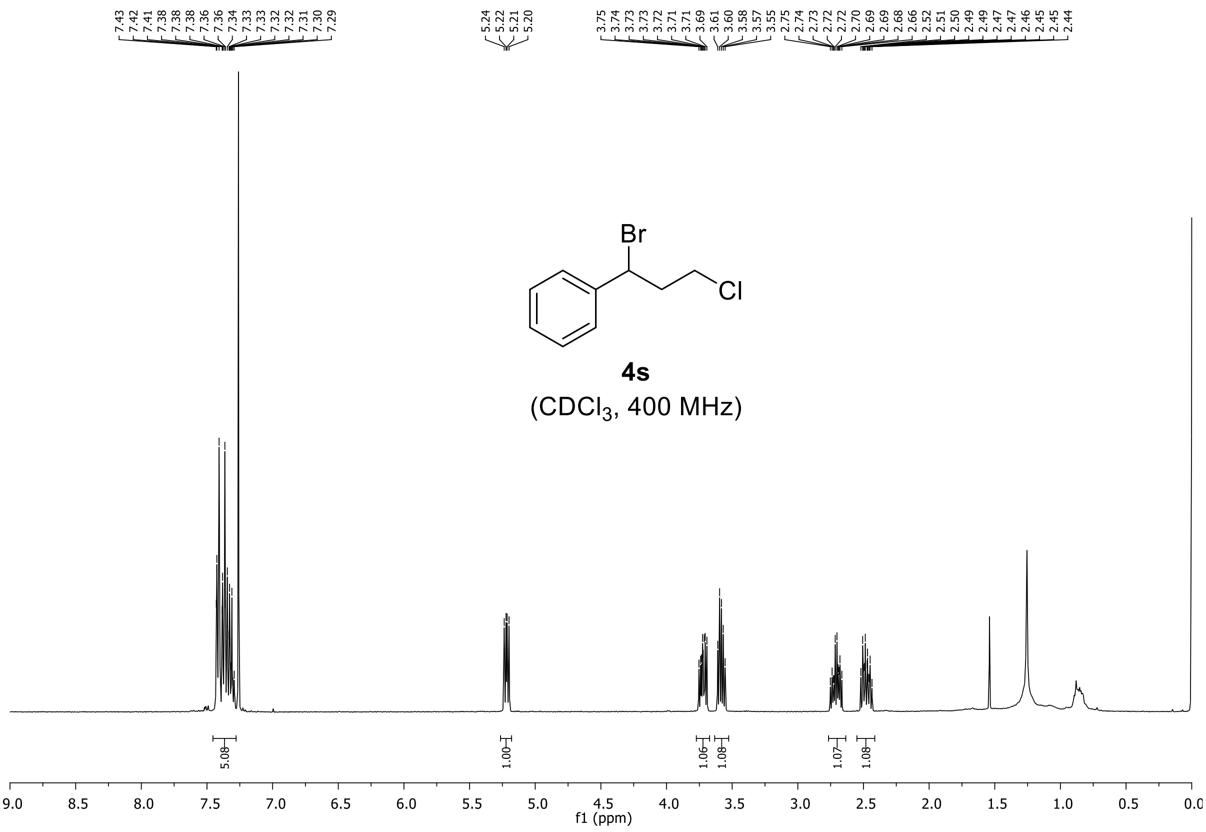
7.43 7.42 7.41 7.38 7.38 7.38 7.36 7.34 7.33 7.33 7.32 7.32 7.31 7.30 7.29
5.24 5.22 5.21 5.20
3.75 3.74 3.73 3.73 3.72 3.71 3.71 3.69 3.61 3.60 3.58 3.57 3.55 2.75 2.74 2.73 2.72 2.72 2.70 2.69 2.69 2.68 2.66 2.52 2.51 2.50 2.49 2.49 2.47 2.47 2.46 2.45 2.45 2.44
Br
Cl
4s
(CDCl3, 400 MHz)
5.08
1.00
1.06
1.08
1.07
1.08
9.0 8.5 8.0 7.5 7.0 6.5 6.0 5.5 5.0 4.5 4.0 3.5 3.0 2.5 2.0 1.5 1.0 0.5 0.0
f1 (ppm)

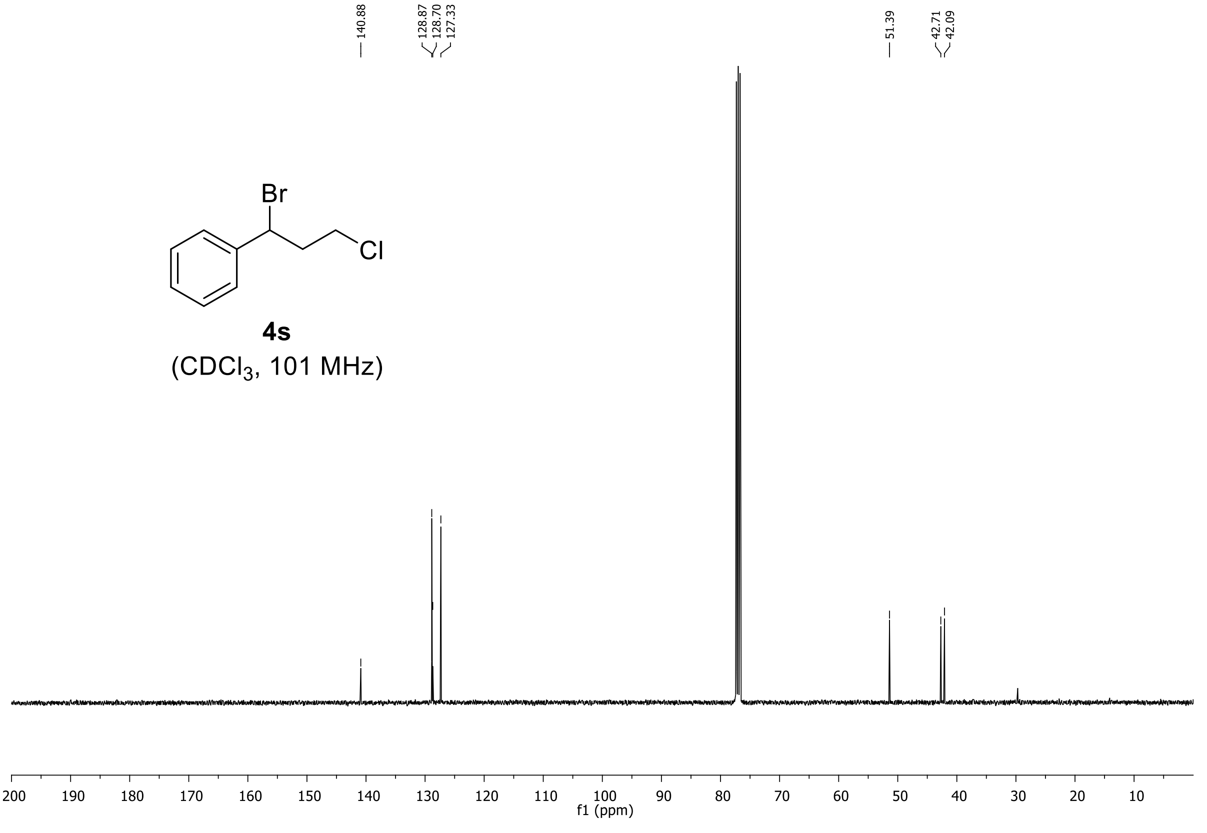
140.88
128.87 128.70 127.33
51.39
42.71 42.09
Br
Cl
4s
(CDCl3, 101 MHz)
200 190 180 170 160 150 140 130 120 110 100 90 80 70 60 50 40 30 20 10
f1 (ppm)

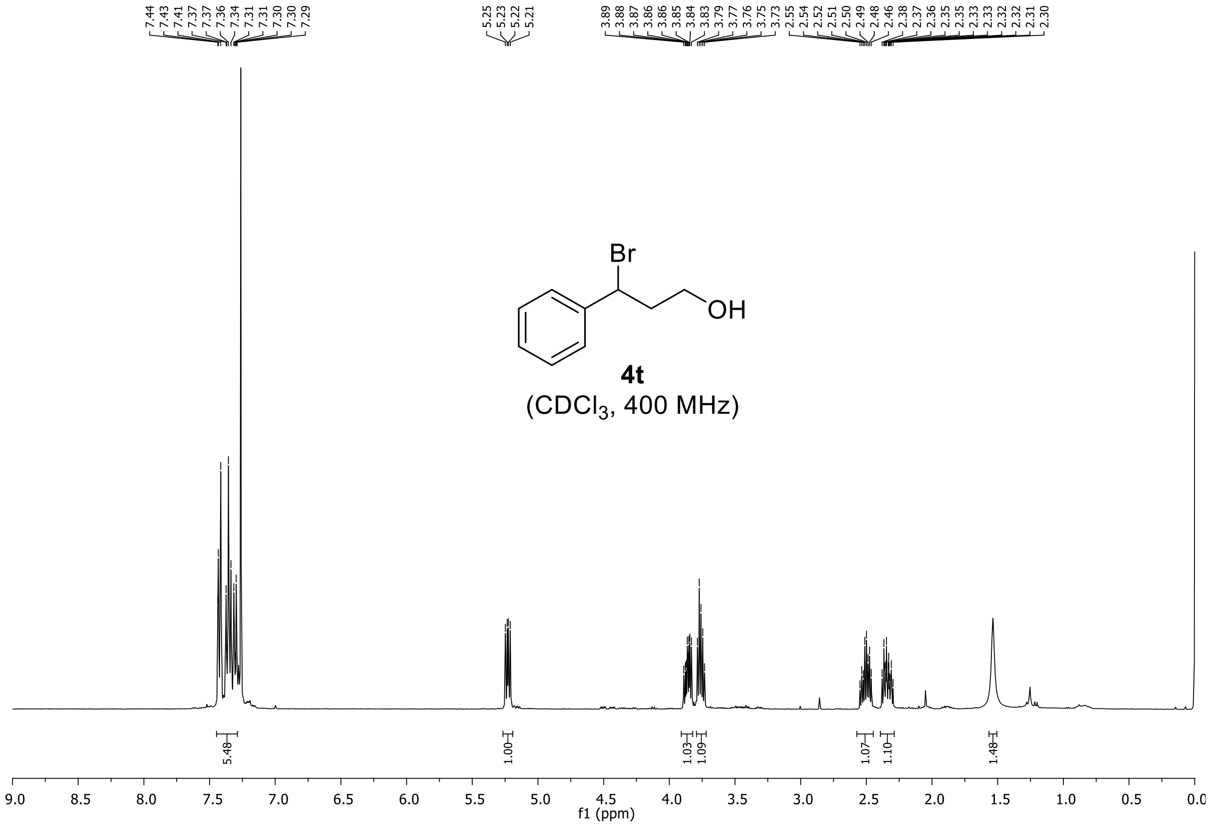
7.44 7.43 7.41 7.37 7.37 7.36 7.34 7.31 7.31 7.30 7.30 7.29
5.25 5.23 5.22 5.21
3.89 3.88 3.87 3.86 3.86 3.85 3.84 3.83 3.79 3.77 3.76 3.75 3.73
2.55 2.54 2.52 2.51 2.50 2.49 2.48 2.46 2.38 2.37 2.36 2.35 2.35 2.33 2.33 2.32 2.32 2.31 2.30
Br
OH
4t
(CDCl$_3$, 400 MHz)
5.48
1.00
1.03
1.09
1.07
1.10
1.48
9.0 8.5 8.0 7.5 7.0 6.5 6.0 5.5 5.0 4.5 4.0 3.5 3.0 2.5 2.0 1.5 1.0 0.5 0.0
f1 (ppm)

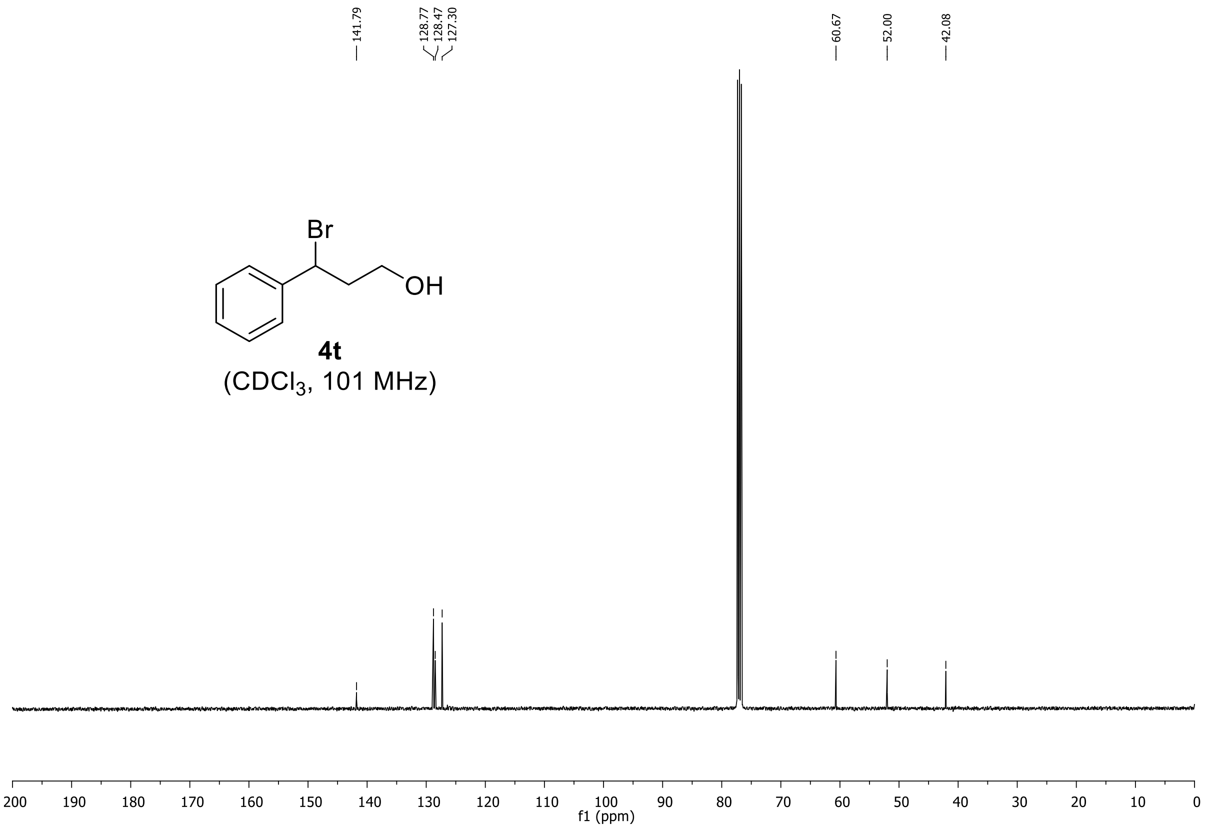
141.79
128.77 128.47 127.30
60.67
52.00
42.08
Br
OH
4t
(CDCl$_3$, 101 MHz)
200 190 180 170 160 150 140 130 120 110 100 90 80 70 60 50 40 30 20 10 0
f1 (ppm)

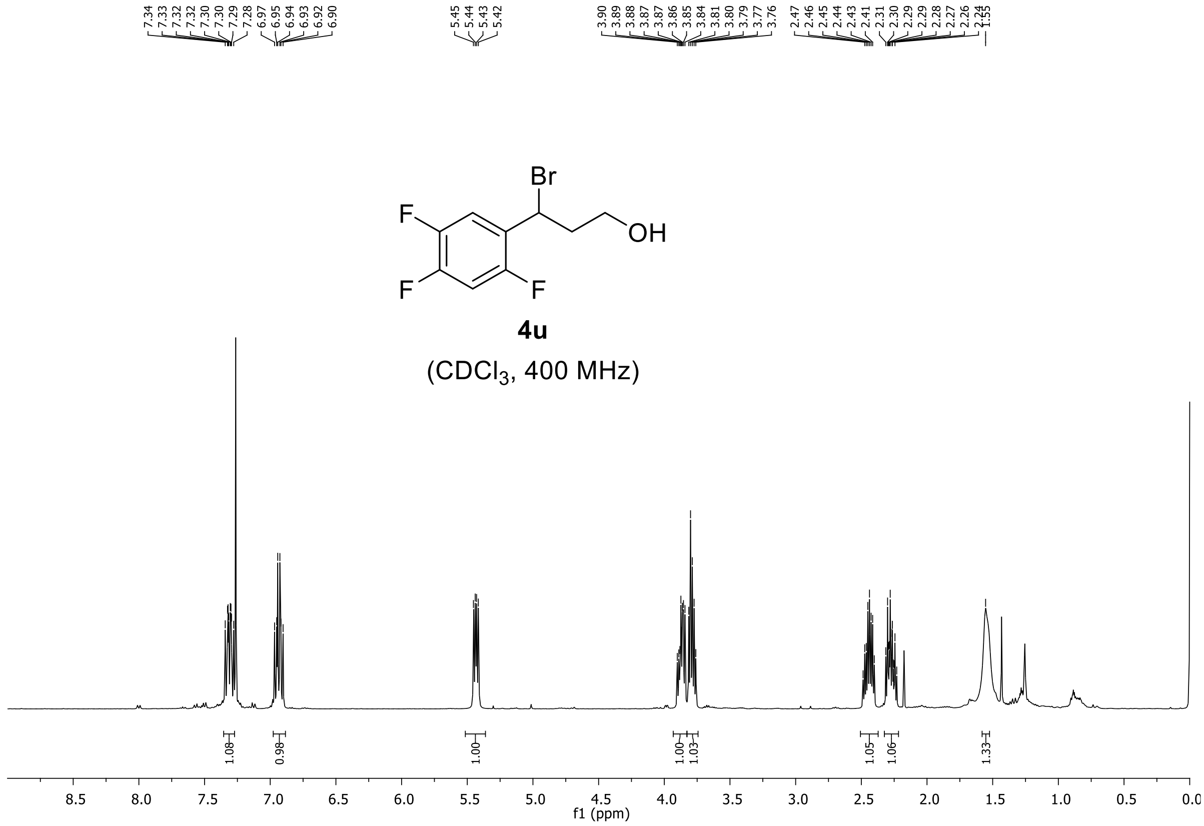
Br
OH
F
F
F
4u
(CDCl3, 400 MHz)
f1 (ppm)

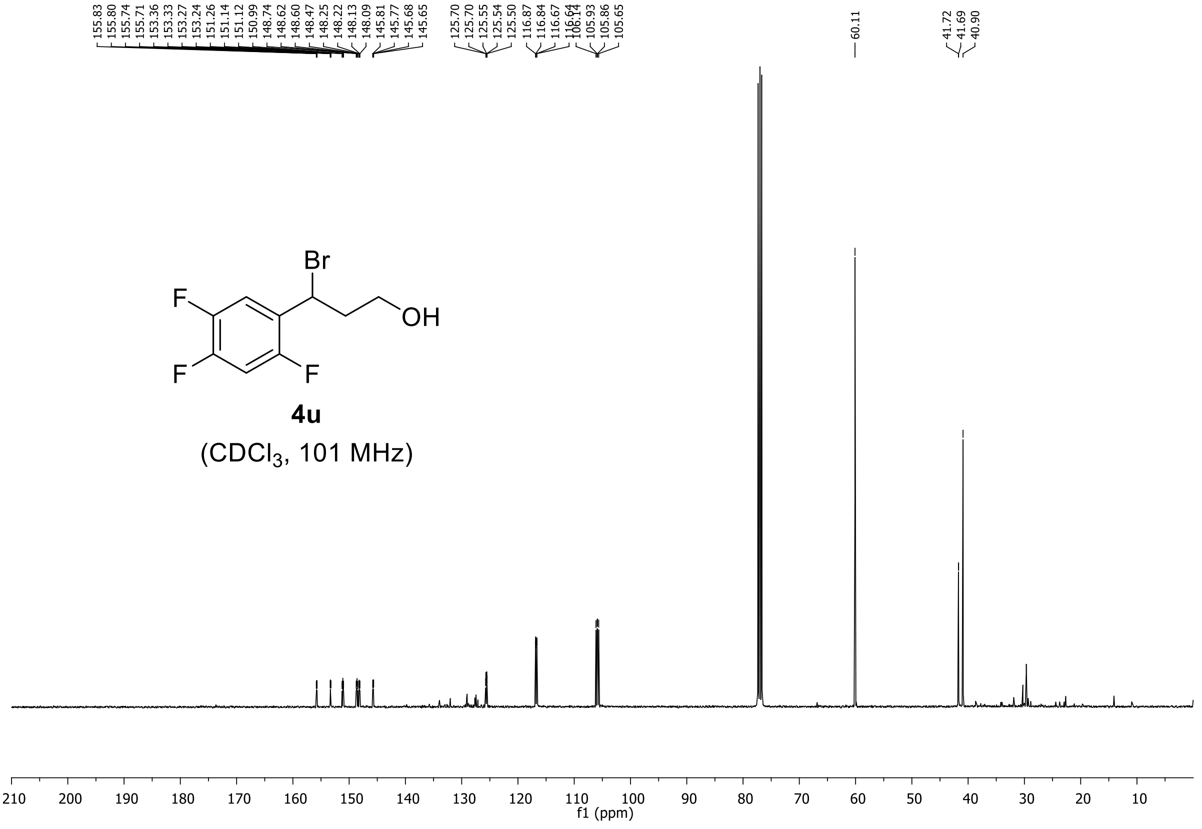
Br
OH
F
F
F
4u
(CDCl3, 101 MHz)
f1 (ppm)

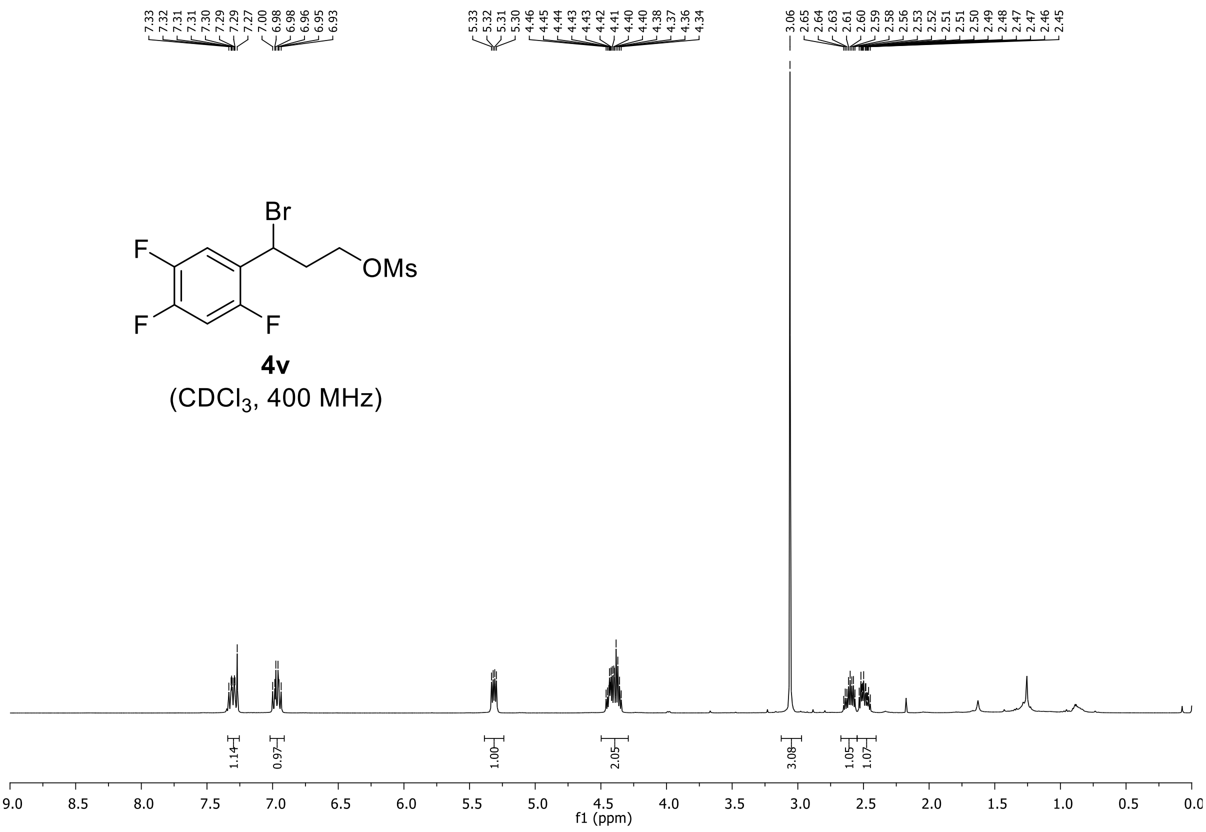
Br
F
OMs
F
F
4v
(CDCl3, 400 MHz)
f1 (ppm)

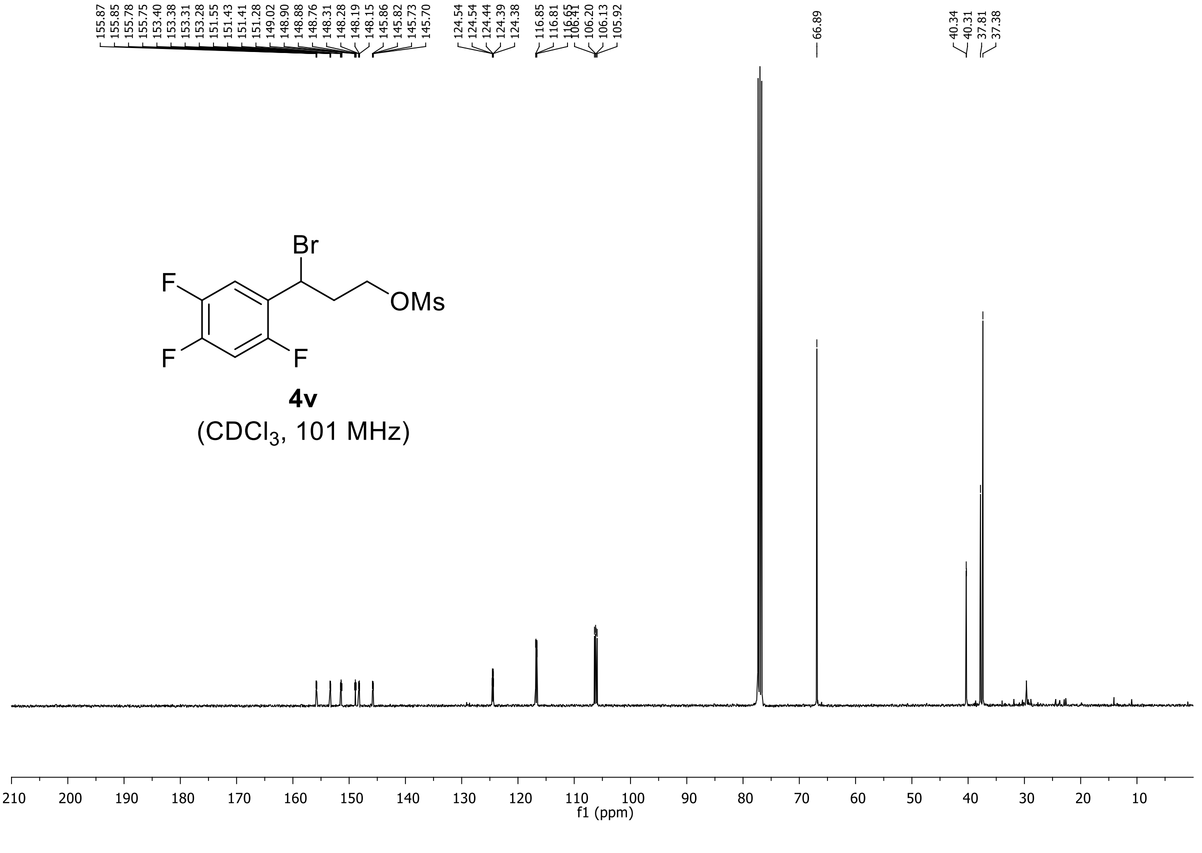
Br
F
OMs
F
F
4v
(CDCl3, 101 MHz)
f1 (ppm)

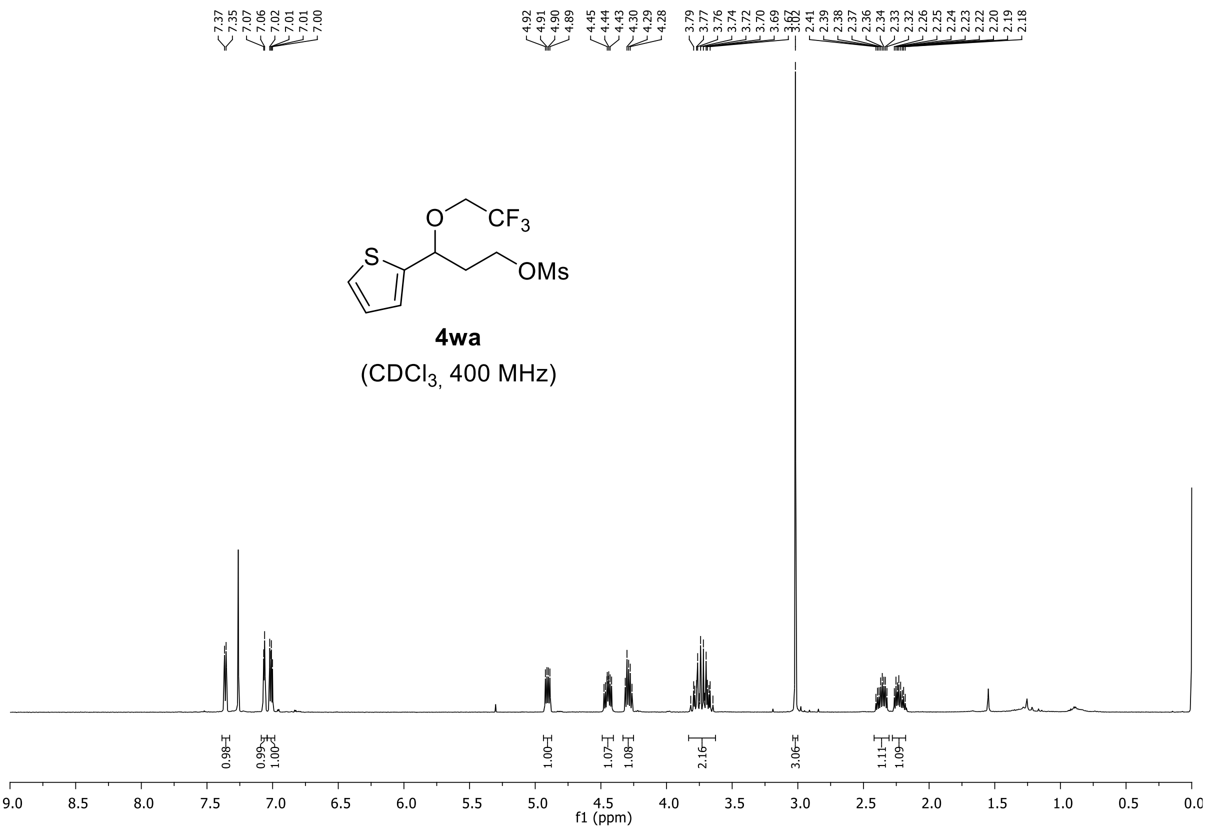
7.37
7.35
7.07
7.06
7.02
7.01
7.01
7.00
4.92
4.91
4.90
4.89
4.45
4.44
4.43
4.30
4.29
4.28
3.79
3.77
3.76
3.74
3.72
3.70
3.69
3.67
3.02
2.41
2.39
2.38
2.37
2.36
2.34
2.33
2.32
2.26
2.25
2.24
2.23
2.22
2.20
2.19
2.18
O
CF3
S
OMs
4wa
(CDCl3, 400 MHz)
0.98
0.99
1.00
1.00
1.07
1.08
2.16
3.06
1.11
1.09
9.0 8.5 8.0 7.5 7.0 6.5 6.0 5.5 5.0 4.5 4.0 3.5 3.0 2.5 2.0 1.5 1.0 0.5 0.0
f1 (ppm)

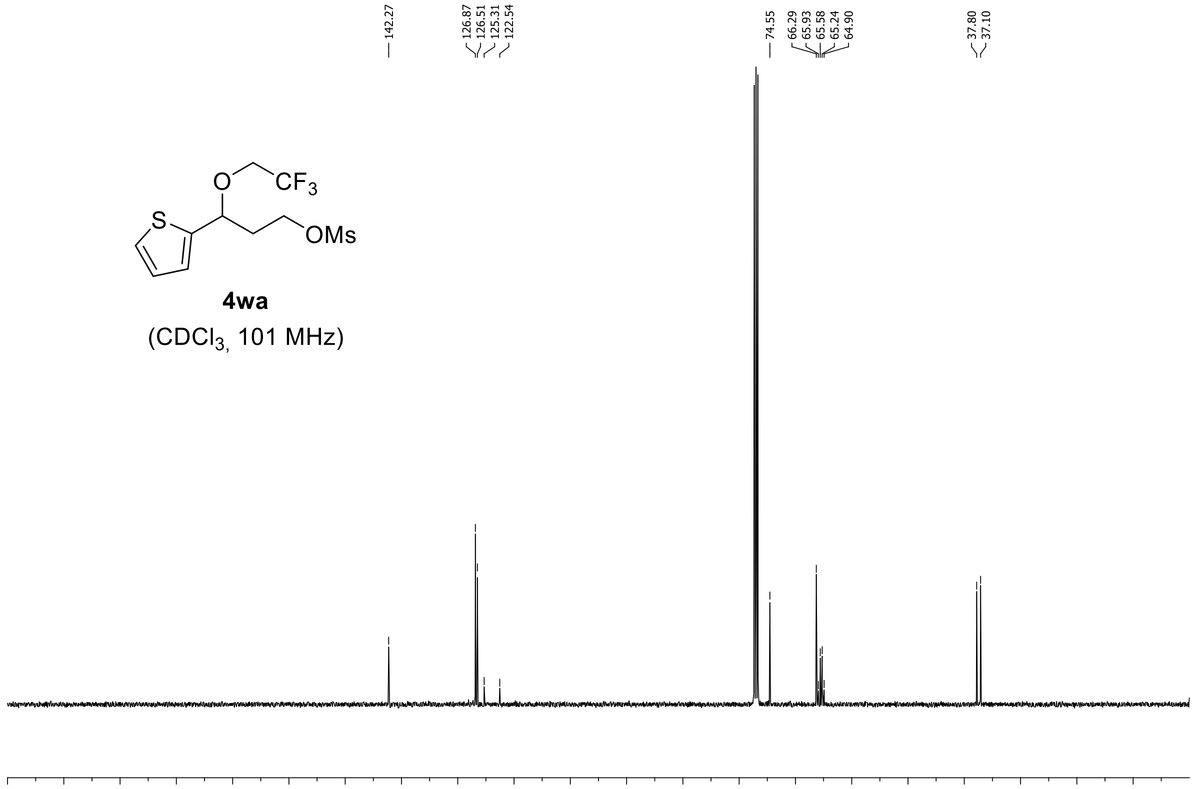
142.27
126.87
126.51
125.31
122.54
74.55
66.29
65.93
65.58
65.24
64.90
37.80
37.10
O
CF3
S
OMs
4wa
(CDCl3, 101 MHz)
210 200 190 180 170 160 150 140 130 120 110 100 90 80 70 60 50 40 30 20 10
f1 (ppm)

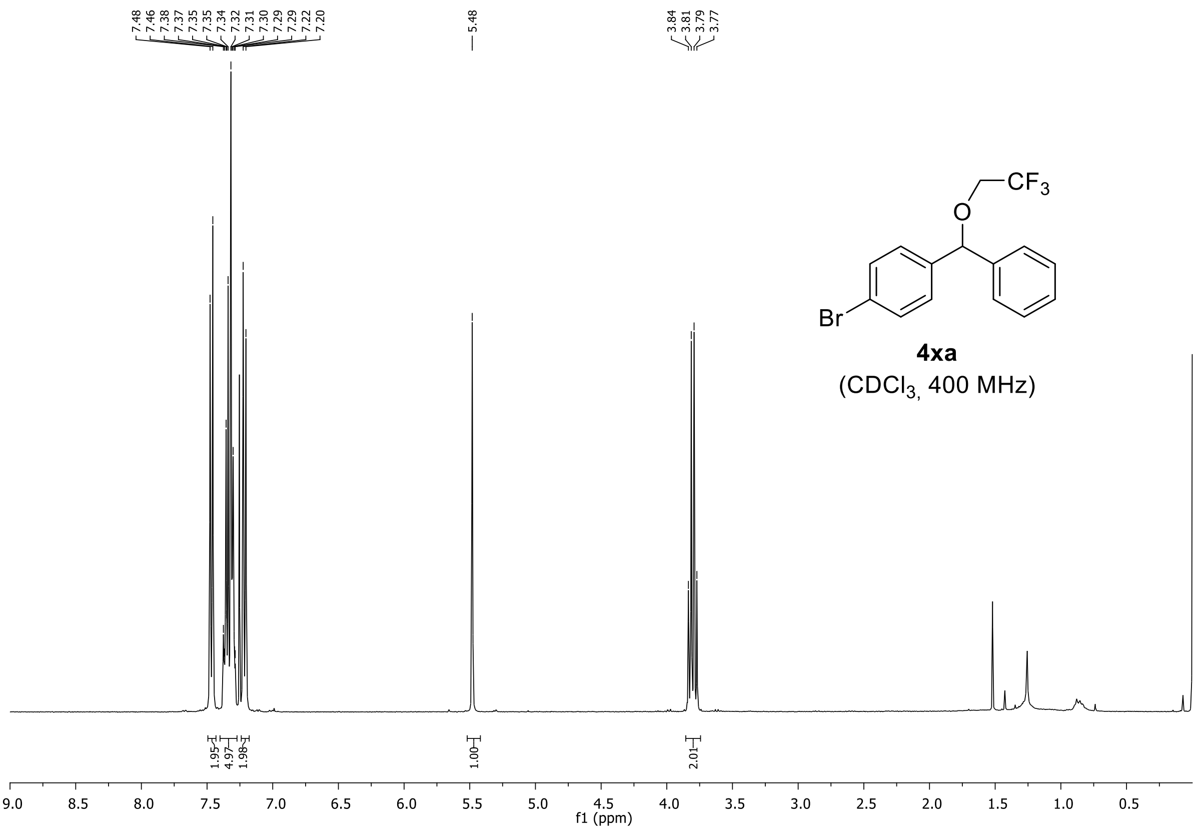
7.48 7.46 7.38 7.37 7.35 7.35 7.34 7.32 7.31 7.30 7.29 7.29 7.22 7.20
5.48
3.84 3.81 3.79 3.77
CF3
O
Br
4xa
(CDCl3, 400 MHz)
1.95 4.97 1.98
1.00
2.01
9.0 8.5 8.0 7.5 7.0 6.5 6.0 5.5 5.0 4.5 4.0 3.5 3.0 2.5 2.0 1.5 1.0 0.5
f1 (ppm)

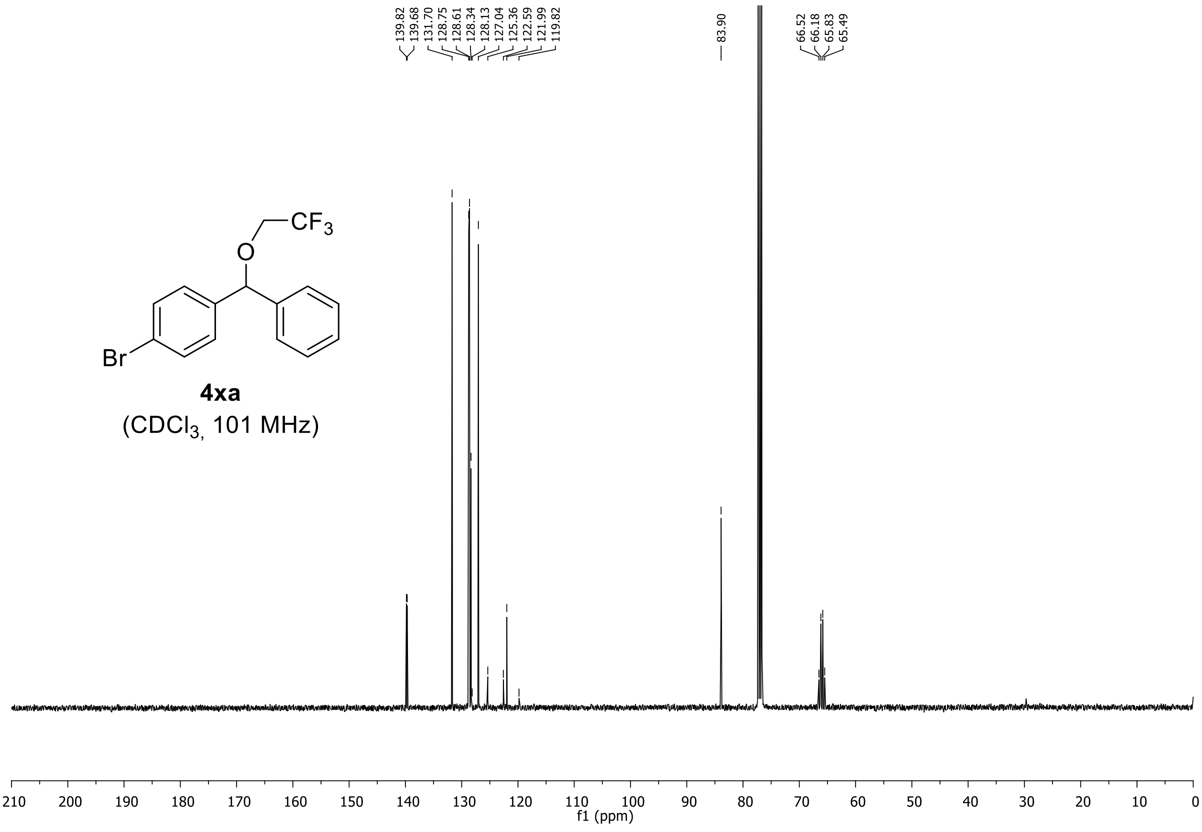
139.82 139.68 131.70 128.75 128.61 128.34 128.13 127.04 125.36 122.59 121.99 119.82
83.90
66.52 66.18 65.83 65.49
CF3
O
Br
4xa
(CDCl3, 101 MHz)
210 200 190 180 170 160 150 140 130 120 110 100 90 80 70 60 50 40 30 20 10 0
f1 (ppm)

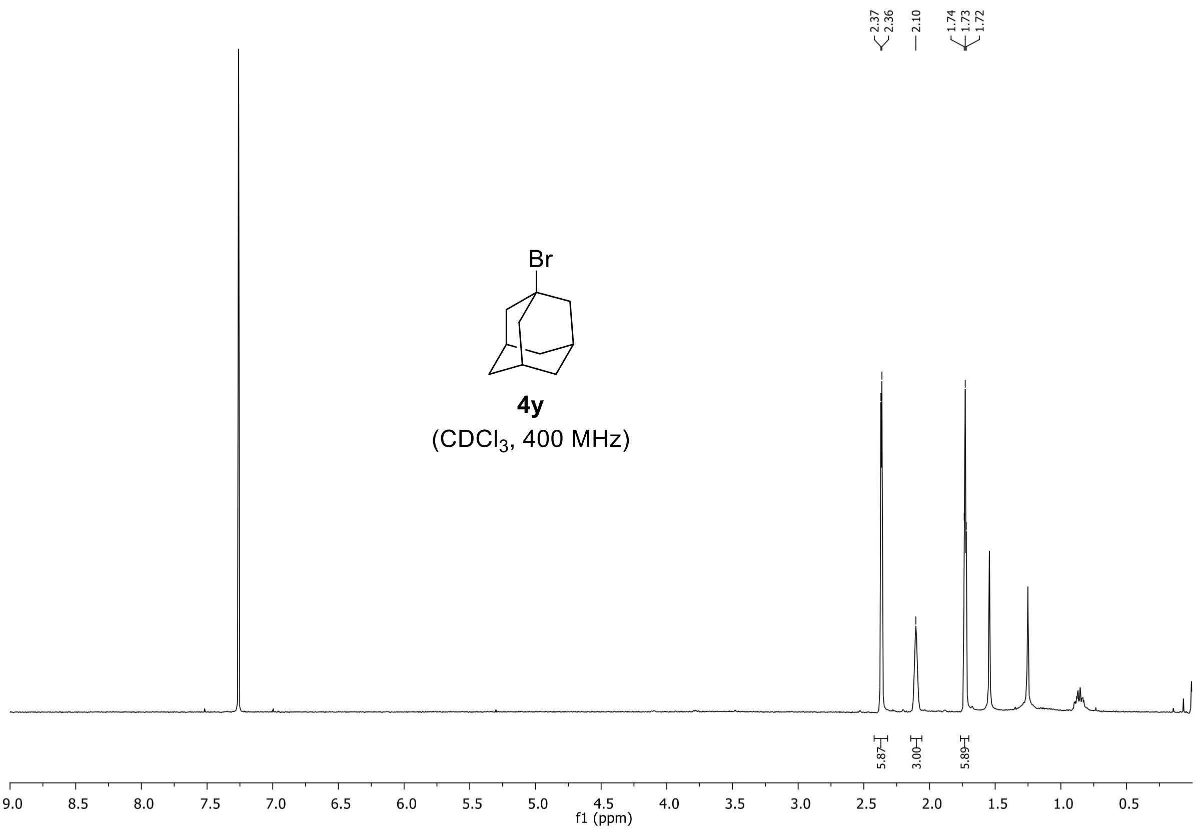
2.37
2.36
2.10
1.74
1.73
1.72
Br
4y
(CDCl3, 400 MHz)
5.87
3.00
5.89
9.0 8.5 8.0 7.5 7.0 6.5 6.0 5.5 5.0 4.5 4.0 3.5 3.0 2.5 2.0 1.5 1.0 0.5
f1 (ppm)

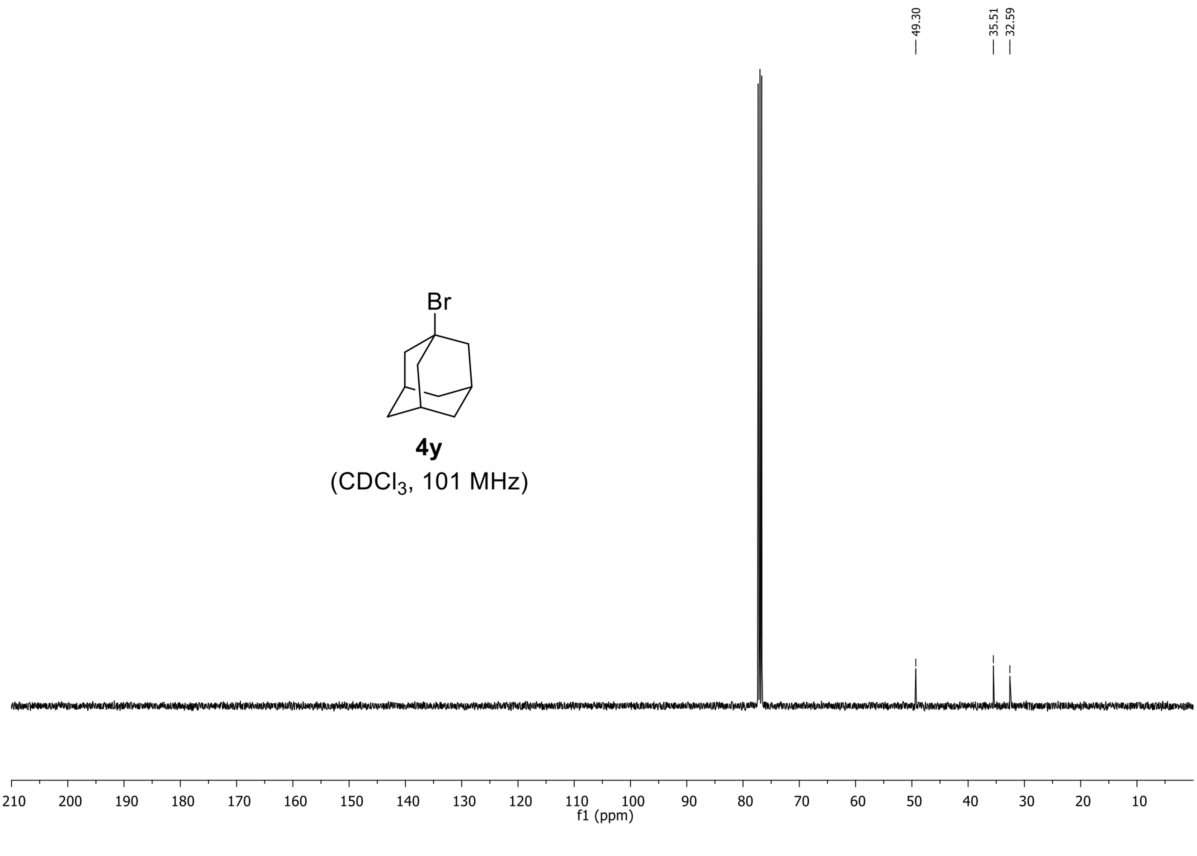
49.30
35.51
32.59
Br
4y
(CDCl3, 101 MHz)
210 200 190 180 170 160 150 140 130 120 110 100 90 80 70 60 50 40 30 20 10
f1 (ppm)

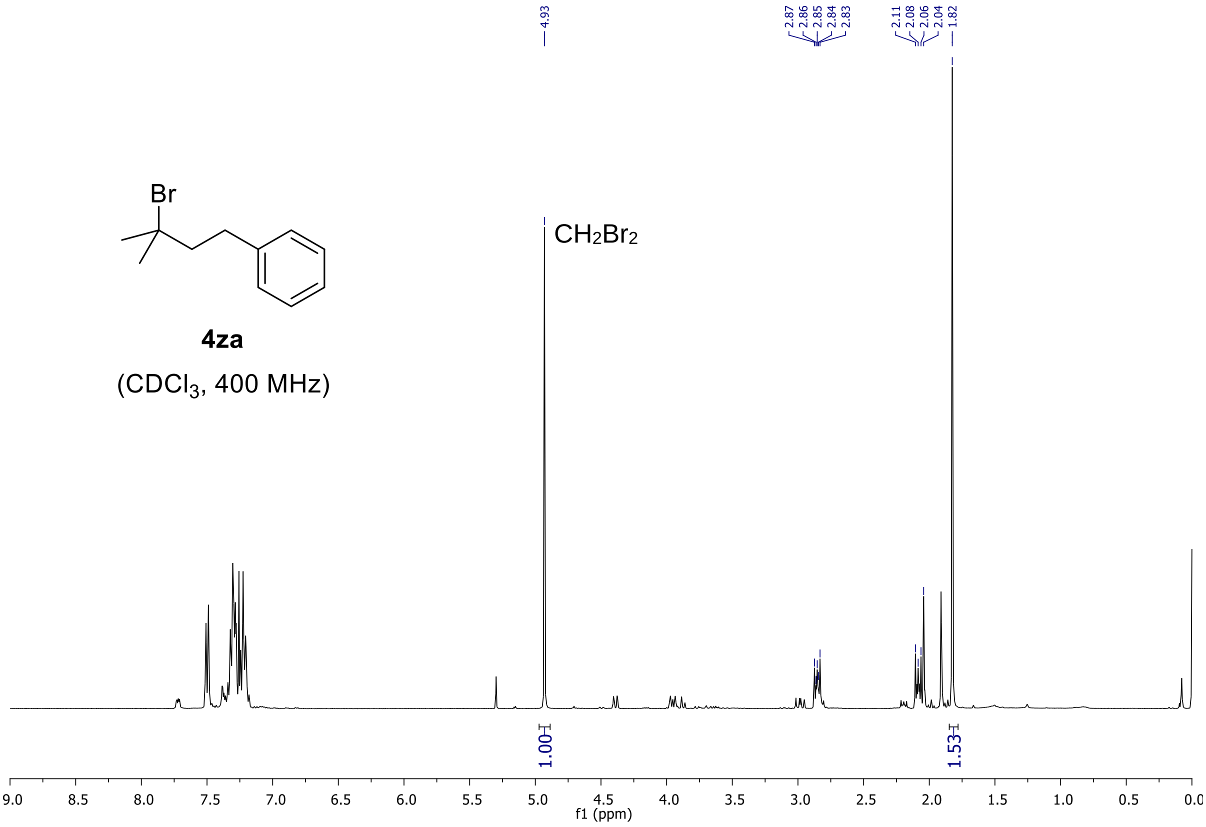

Br
4za
(CDCl3, 400 MHz)
4.93
2.87
2.86
2.85
2.84
2.83
2.11
2.08
2.06
2.04
1.82
CH2Br2
1.00
1.53
9.0
8.5
8.0
7.5
7.0
6.5
6.0
5.5
5.0
4.5
4.0
3.5
3.0
2.5
2.0
1.5
1.0
0.5
0.0
f1 (ppm)

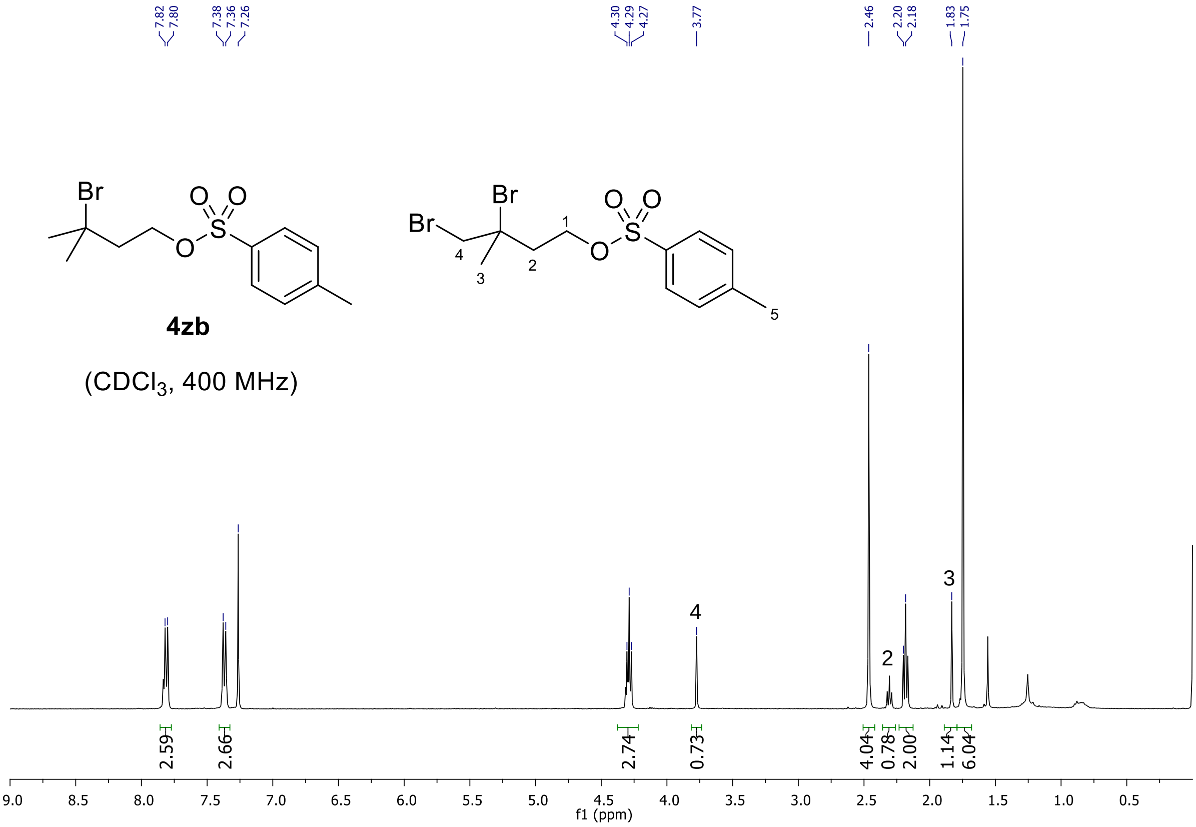
7.82
7.80
7.38
7.36
7.26
4.30
4.29
4.27
3.77
2.46
2.20
2.18
1.83
1.75
Br
O O
S
O
4zb
(CDCl3, 400 MHz)
Br
Br
1
2
3
4
5
4
2
3
2.59
2.66
2.74
0.73
4.04
0.78
2.00
1.14
6.04
9.0 8.5 8.0 7.5 7.0 6.5 6.0 5.5 5.0 4.5 4.0 3.5 3.0 2.5 2.0 1.5 1.0 0.5
f1 (ppm)

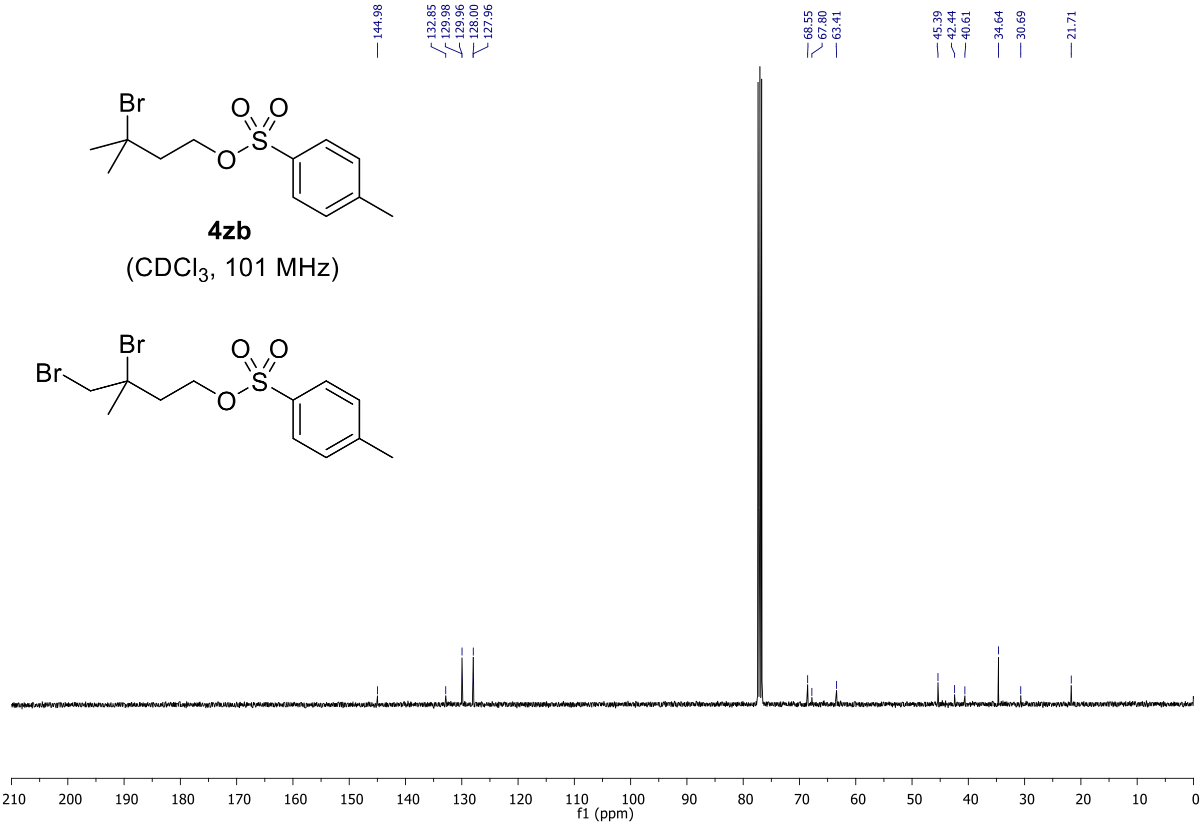
144.98
132.85
129.98
129.96
128.00
127.96
68.55
67.80
63.41
45.39
42.44
40.61
34.64
30.69
21.71
Br
O O
S
O
4zb
(CDCl3, 101 MHz)
Br
Br
210 200 190 180 170 160 150 140 130 120 110 100 90 80 70 60 50 40 30 20 10 0
f1 (ppm)

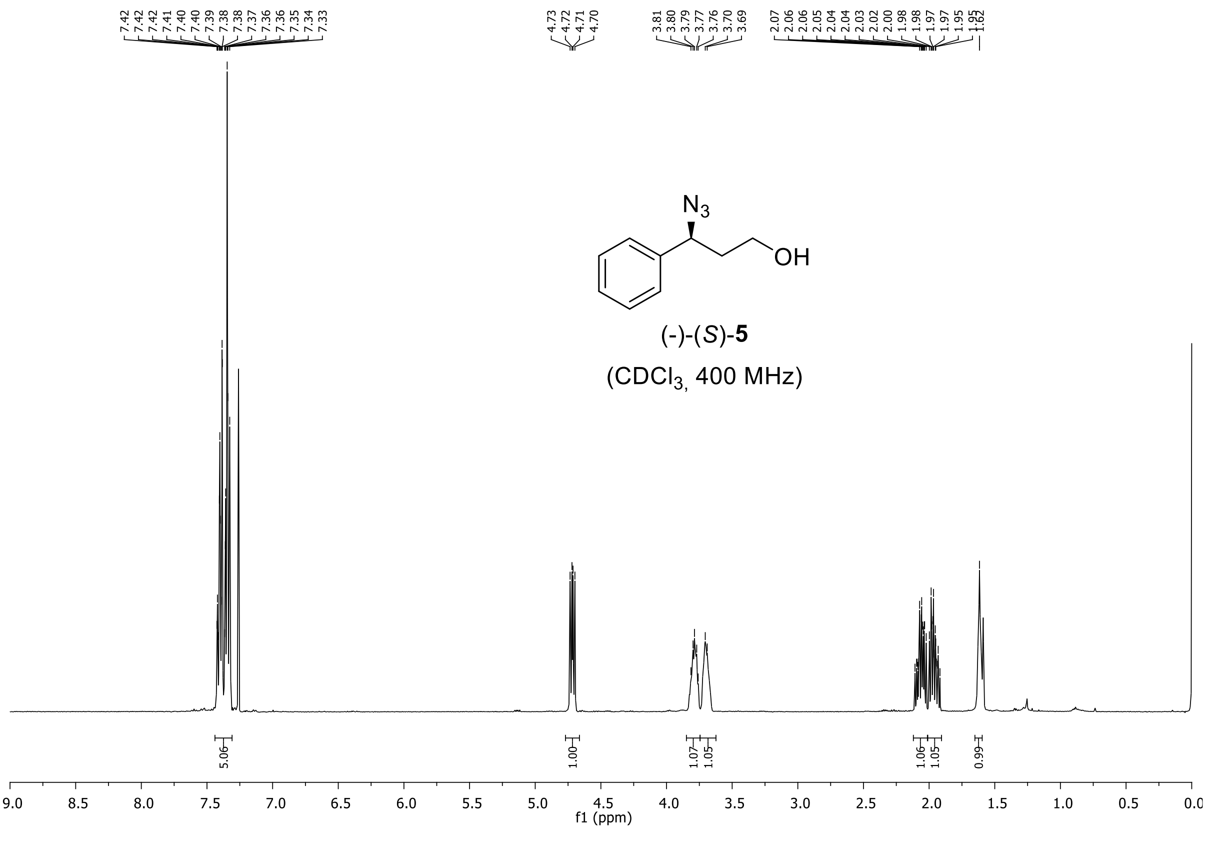
N3
OH
(-)-(S)-5
(CDCl3, 400 MHz)
f1 (ppm)

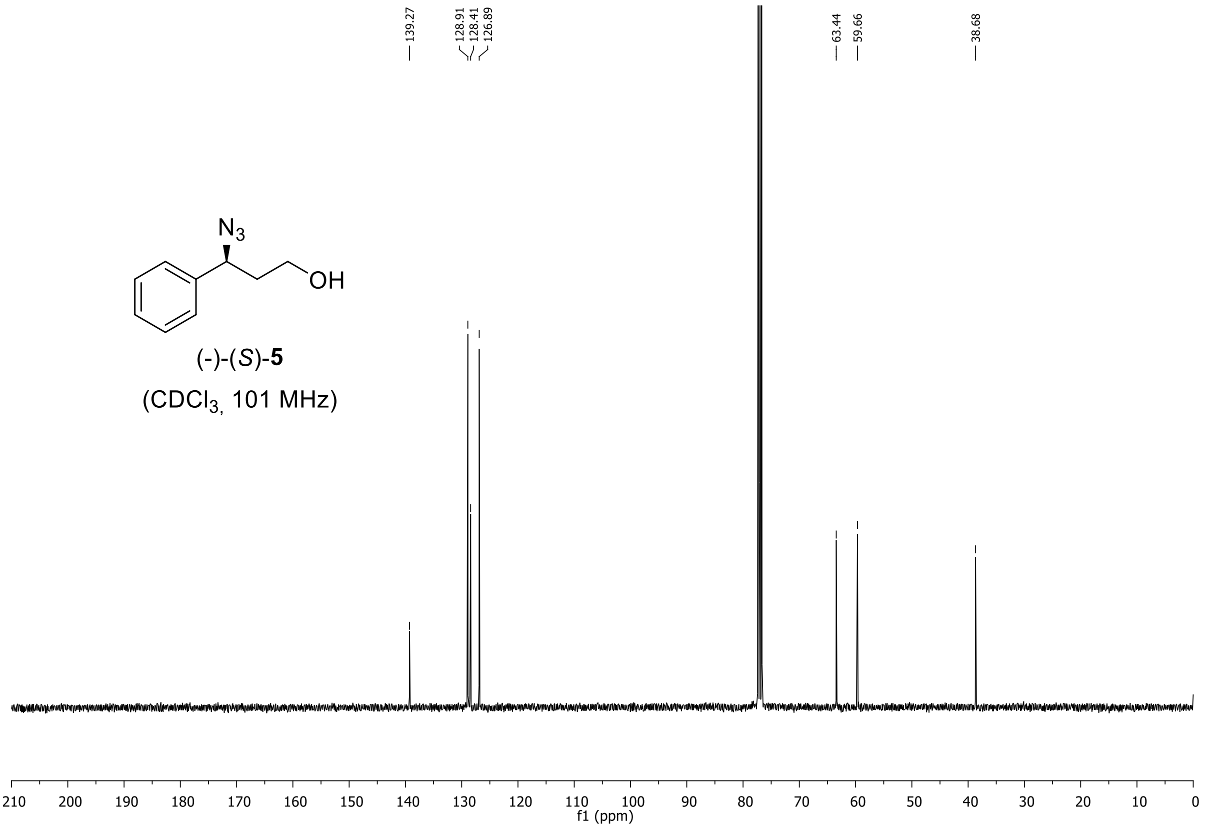
N3
OH
(-)-(S)-5
(CDCl3, 101 MHz)
f1 (ppm)
